# Consecutive One-Pot versus Domino Multicomponent Approaches to 3-(Diarylmethylene)oxindoles

**DOI:** 10.3390/molecules22030503

**Published:** 2017-03-22

**Authors:** Sunhwa Park, Jiyun Lee, Kye Jung Shin, Euichaul Oh, Jae Hong Seo

**Affiliations:** Integrated Research Institute of Pharmaceutical Sciences, College of Pharmacy, The Catholic University of Korea, Bucheon-si, Gyeonggi-do 420-743, Korea; sunalovegs@catholic.ac.kr (S.P.); ljiy727@catholic.ac.kr (J.L.); kyejung@catholic.ac.kr (K.J.S.); eoh@catholic.ac.kr (E.O.)

**Keywords:** consecutive one-pot reaction, domino reaction, 3-(diarylmethylene)oxindole, palladium-catalyzed reaction, microwave irradiation

## Abstract

Based on consecutive one-pot conditions combining three palladium-catalyzed reactions (Sonogashira, Heck and Suzuki-Miyaura reactions), a more efficient domino multicomponent method has been successfully developed to access a wide variety of 3-(diarylmethylene)oxindoles. Microwave irradiation and use of a silver salt were the most important factors to achieve high yields and stereoselectivity.

## 1. Introduction

**Scheme 1:**
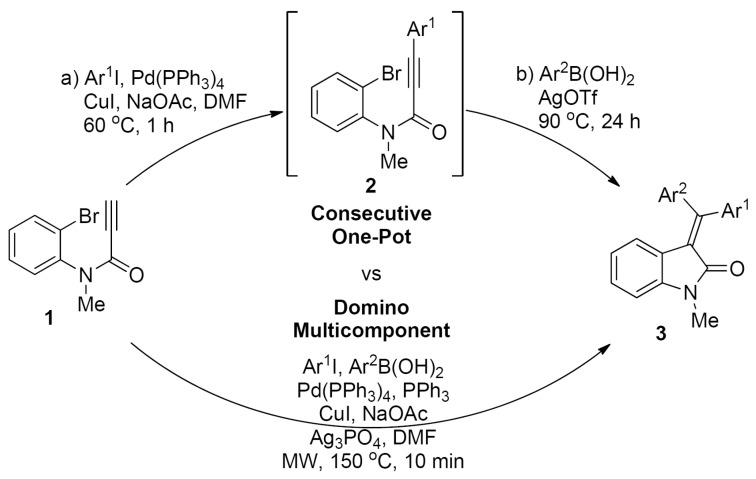
Consecutive one-pot versus domino MCR approach to 3-(diarylmethylene)oxindoles.

Multicomponent reactions (MCRs) are generally understood as reactions in which three or more substrates are added at the beginning of the reaction to afford the products via two or more chemical transformations [1]. Inherently, well-designed MCRs can provide an efficient synthetic method for complex skeletons from simple substrates, securing diversity of products in a less time-consuming and more economical manner compared with the corresponding stepwise approaches [2]. These features of MCRs have attracted much interest among synthetic chemists in the pharmaceutical industry aiming to apply MCRs to create a wide variety of small molecule libraries [3,4,5]. Various heterocycles have also been synthesized via MCRs [6], especially utilizing transition metal catalysts [7]. Recently, as part of our ongoing efforts to find efficient synthetic methods for biologically active skeletons, we reported two such methods [8,9] for the preparation of 3-(diarylmethylene)oxindoles (Scheme 1), which are attracting increased interest due to their recently identified biological activities, such as AMPK activation [10] and anti-breast-cancer activity [11]. Both of our methods involve a simple propiolamide **1**, and commercially available aryl iodides and arylboronic acids as starting materials to produce 3-(diarylmethylene)oxindoles via three successive palladium-catalyzed reactions: the Sonogashira, Heck, and Suzuki-Miyaura reactions. However, there is a big difference between the two methods in terms of the reaction process. The first method, under thermal conditions, begins with the Sonogashira reaction of propiolamide **1** and aryl iodide at 60 °C to give Sonogashira adduct **2**, which then proceeds through Heck and Suzuki-Miyaura reactions upon the addition of arylboronic acid and elevation of the reaction temperature to 90 °C [8]. It is worth noting that the addition of a silver salt (AgOTf) with the arylboronic acid enhances the *E*/*Z* stereoselectivity of unsymmetrically substituted 3-(diarylmethylene)oxindoles. In contrast, the second method, under microwave-assisted conditions, requires neither a second addition, nor a temperature change [9]. Although both methods are one-pot reactions, the first method is a consecutive one-pot reaction requiring more than two separate operations, whereas the second method is a domino MCR, which offers a more efficient chemical transformation but makes it harder to achieve optimal conditions. Herein, we report our efforts to develop efficient domino MCRs from consecutive one-pot conditions and wide screening of the substrate scope of the resulting domino MCRs, which contributes to our understanding of the mechanism of the reaction and could inform future studies on MCRs.

## 2. Results and Discussion

To verify the possibility of domino MCRs, we started with the addition of all three substrates (propiolamide **1**, aryl iodide and arylboronic acid) at the beginning of the reaction, which were then exposed to the same reaction conditions for the consecutive one-pot reaction (Pd(PPh_3_)_4_, CuI, NaOAc, DMF; 60 °C for 1.5 h, then 90 °C for 24 h) (Table 1). A silver salt (AgOTf) was not added in this preliminary study because it does not affect reaction progress, but mainly enhances the stereoselectivity of unsymmetrically substituted products. The main concern of this study was possibility of the direct Suzuki-Miyaura reaction between aryl iodide and arylboronic acid, to produce biphenyl byproducts **4**. However, the formation of biphenyl compounds **4** was less than 15% for both symmetric (entries 1–3) and unsymmetric products (entries 4–10). The yield and *E*/*Z* stereoselectivity were also similar to those of consecutive one-pot conditions. These results imply that aryl iodide reacted first with propiolamide **1** rather than arylboronic acid at the initial temperature (60 °C), to produce the corresponding Sonogashira adduct **2** that was then transformed into the desired **3** at a higher temperature (90 °C).

In spite of the promising preliminary results, the *E*/*Z* stereoselectivity for unsymmetrically substituted products was still poor without silver salt (Table 1; entries 5–10). Thus, we investigated the ability of silver salt to improve the stereoselectivity of the products (Table 2). In the initial screening of silver salts, AgOTf showed the best enhancement of stereoselectivity without any compensatory loss of product yield [8]. Subsequently, more intensive screenings for silver salts revealed that Ag_3_PO_4_ is a better additive for this purpose, as shown in Table 2. When Ag_3_PO_4_ was added at the beginning of the reaction with all substrates, stereoselectivity was enhanced as expected, but yields decreased with the formation of a substantial amount of the biphenyl product **4** (20%–40% yield). Silver salt might hamper the Sonogashira reaction between **1** and aryl iodide, allowing more aryl iodide to undergo the direct Suzuki-Miyaura reaction with arylboronic acid.

To solve the problem of low yield with the addition of silver salt, we screened various phosphine ligands, expecting improvement of yield while maintaining good stereoselectivity (Table 3, entries 1–5). Bidentate phosphine ligands are known as effective ligands for the cationic pathway of palladium-catalyzed reactions [12]. Unfortunately, the addition of bidentate ligands (dppp, dppb and dppf) did not afford the desired product **3f** (entries 1–3). When P(*o*-tol)_3_ was added, **3f** was obtained in poor yield (13%) (entry 4). Addition of PPh_3_ showed a promising result (entry 5; 50% yield, 5:1 *E*/*Z* ratio) but was still less effective than the consecutive one-pot conditions with Ag_3_PO_4_ (Table 2, entry 2; 86% yield, 9:1 *E*/*Z* ratio). Interestingly, elevation of the initial reaction temperature to 90 °C, which removed the additional temperature change operation, provided **3f** in 67% yield with a 7.5:1 *E*/*Z* ratio (entry 6). When the reaction temperature was further increased (150 °C), the reaction rate increased but yield and stereoselectivity decreased slightly (entries 6 and 7). These results showed that high reaction temperature reduced the hampering effect of silver salt on the initial Sonogashira reaction.

Considering the above results, we decided to test microwave irradiation conditions, which are known for accelerating sluggish thermal reactions [13,14,15] (Table 4). Reactions with microwave irradiation at 100 °C, for 10, 30 and 60 min, increased yields from 46% to 80% with increasing reaction time, and the stereoselectivity in all three cases was very high (9:1~18:1 *E*/*Z* ratio, entries 1–3). Higher reaction temperatures (110 and 130 °C) made it possible to complete the reaction in a shortest time (10 min) with good yield and stereoselectivity (entries 4 and 5). The best result was obtained at 150 °C, presenting 99% yield and a 13:1 *E*/*Z* ratio (entry 6). At 160 °C, the reaction was completed in 5 min to give **3f** in 98% yield with slightly lower stereoselectivity (7.5:1 *E*/*Z* ratio) (entry 7).

Based on these favorable results under microwave conditions, we decided to investigate the effects of additives on microwave-assisted reactions by performing a blank test. Without phosphine ligand (PPh_3_), the reaction still proceeded smoothly, to provide **3f** in slightly lower yield and stereoselectivity (entry 8; 92% yield, 8:1 *E*/*Z* ratio). However, the removal of silver salt (Ag_3_PO_4_) dramatically decreased the yield (36%) and stereoselectivity (1:1.2 *E*/*Z* ratio) (entry 9). Without both PPh_3_ and Ag_3_PO_4_, the lowest yield (35%) and stereo-inverted selectivity (1:1.5 *E*/*Z* ratio) were obtained (entry 10). Interestingly, the addition of two equivalents of Ag_3_PO_4_ without base (NaOAc) still provided product **3f** in moderate yield and stereoselectivity (entry 11). Thus, under microwave irradiation conditions, silver salt seems to be a crucial additive not only for good stereoselectivity, but also for high yield.

With optimized microwave-assisted conditions in hand, we investigated the substrate scope for the synthesis of symmetrically substituted 3-(diarylmethylene)oxindoles (Table 5). Regardless of the substituents on the aryl group, all reactions gave the desired products **3a**–**d** in good yield (entries 1–4). To verify the effect of silver salt, control experiments without Ag_3_PO_4_ were also performed. As expected, the absence of silver salt decreased the yield of all reactions. However, the degree of yield reduction appears to be related to the electronic effect of the substituent on the aryl group. The formation of **3b** bearing the electron-donating methoxy group at the 4-position was hardly affected by the absence of Ag_3_PO_4_ (75% vs. 55%); whereas the yields for **3d** with the electron-withdrawing nitro group at the same position changed dramatically, from 90% to 9%, without addition of Ag_3_PO_4_. This suggests that the positive effect of Ag_3_PO_4_ on the domino MCRs is due in part to enhancing the last Suzuki-Miyaura reaction step [16].

Next, we tested the effect of our reaction conditions on the synthesis of unsymmetrically substituted 3-(diarylmethylene)oxindoles having electronically different substituents at the 4-position of the phenyl group (Table 6). All reactions provided products **3e**–**g** in good yield (80%–99%). Stereoselectivity for each reaction was more than 8:1, giving the expected stereoisomer as the major product, except in the reaction with 4-methoxyphenyl iodide, in which the *E*/*Z* ratio of 1.2:1 resulted in a little more of the stereo-inverted *E*-isomer than the *Z*-isomer. This exceptional case will be revisited later through extensive screening of the reaction conditions. To confirm the ligand effect on our domino MCRs, all reactions were tested again without PPh_3_, which resulted in similar but slightly lower yield and stereoselectivity. The addition of PPh_3_ did not enhance the reaction as much as silver salt, but nevertheless surely plays a meaningful role in producing good yield and stereoselectivity of the domino MCRs.

To widen the application of the reaction, a more expanded substrate scope was investigated (Table 7). The reaction of 4-acetoxyphenylboronic acid showed relatively low yield (45%) but high stereoselectivity (10:1 *E*/*Z* ratio) (entry 1). Unlike 4-methoxyphenyl iodide, the reaction of 4-acetoxyphenyl iodide presented good yield and stereoselectivity (entry 2), representing an alternative way to synthesize the *Z*-isomer with an oxygen atom at the 4-position of the phenyl group. Moreover, 3-(diarylmethylene)oxindoles with substituents at the 3-position of the phenyl group were easily prepared by our domino MCRs, regardless of the electronic effect of the substituent (entries 3–10). When the yield was moderate, addition of two equivalents of arylboronic acid increased the yield up to 94%, accompanied by slightly lower stereoselectivity (entries 5 and 10). It is worth pointing out that the reaction of 3-methoxyphenyl iodide showed good stereoselectivity (1:8 *E*/*Z* ratio, entry 7). This result suggests that the position of the methoxy group on the aryl iodide is important for determining the stereoselectivity of the reaction. 2-Chloro and 2-methoxyphenylboronic acids were good substrates for the reaction, providing **3l** and **3m** in good yield (entries 11 and 12); however, no product was formed in the reaction of 2-nitrophenylboronic acid (entry 13). 2-Methoxyphenyl iodide reiterated the problem of low stereoselectivity, as well as 4-methoxyphenyl iodide (entry 14). The reaction of 2-chlorophenyl iodide resulted in good yield and stereoselectivity (entry 15), while the 2-nitrophenyl iodide was a poor substrate for the reaction, giving product **3n** in very low yield (entry 16). Taken together, the above results suggest that our domino MCRs have broad substituent tolerance for both aryl iodide and arylboronic acid, producing good yield and stereoselectivity except for 2- or 4-methoxyphenyl iodide and the 2-nitro substituent.

Next, we tried to solve the low stereoselectivity problem for the reaction with 4-methoxyphenyl iodide (Table 8). We focused on the amount of Ag_3_PO_4_, which seems to play a crucial role in achieving good stereoselectivity in the domino MCRs. Increasing the amount of Ag_3_PO_4_ showed little improvement of stereoselectivity, but rather reduced the yield (entries 2–4). Other silver salts were not as effective as Ag_3_PO_4_ (entries 5–8). Then, we lowered the reaction temperature. At 130 °C, stereoselectivity increased slightly (1:1.5 *E/Z* ratio, entry 9). At 100 °C, the reaction rate was much slower and a longer reaction time was needed to achieve reasonable yield. The reaction for 1 h at 100 °C provided the best stereoselectivity (1:4 *E*/*Z* ratio) with good yield (70% entry 10). A longer reaction time (3 h) at 100 °C increased the yield to 79% but decreased stereoselectivity (1:3 *E*/*Z* ratio, entry 11). Thus, in the case of poor stereoselectivity, modification of the reaction temperature and time of the reaction can offer a solution.

Our next challenge was to expand the substrate scope to heteroaryl groups (Table 9). The 3- or 4-pyridinyl group was introduced by our domino MCRs in good yield and high stereoselectivity (entries 1 and 2). The reaction with 3-benzothiophenylboronic acid provided the desired product in 52% yield and 16:1 *E*/*Z* ratio (entry 3). Interestingly, in this reaction, unexpected byproduct **5** was isolated; the structure of **5** was elucidated by intensive one-dimensional (1D) and two-dimensional (2D) nuclear magnetic resonance (NMR) experiments. The formation of tricyclic compound **5** would be explained by insertion of an acetyl group originating from the base (NaOAc). The formation of **5** was easily removed by the use of the more heteroarylboronic acid (2 eq, entry 4); 2-benzothiophenylboronic acid and 3-furanylboronic acid showed similar activity in domino MCRs (entries 5–8), although the stereoselectivity of the reaction with 2-benzothiophenylboronic acid was much lower than for the others (entries 5 and 6).

Next, we investigated the possibility of derivatization of propiolamide **1** (Table 10). When the *N*-substituent of propiolamide **1** was changed to a H or a Bn group, the reactions showed good yield and moderate stereoselectivity (entries 1 and 2). Under standard conditions, one carbon-extended substrate **6c** showed low yield (35%, entry 3). The yield increased to 45% at higher reaction temperature (180 °C) but almost no stereoselectivity was observed (1.1:1 *E*/*Z* ratio, entry 4). Propiolate ester **6d** turned out to be a poor substrate for our domino MCRs, giving both a low yield and poor stereoselectivity (entry 5). All results implied that applying our microwave-assisted domino MCRs to the synthesis of other heterocycles is possible, but needs further study for optimization of each reaction.

In all of the above results, the stereoselectivity of the domino MCRs varied depending on the substrate. One of the reasons for this could be the isomerization of product **3** under the reaction conditions. Thus, we exposed pure (*Z*)- and (*E*)-isomers of **3e** to the standard reaction conditions (Table 11). Both **(*Z*)-** and **(*E*)-3e** showed very low isomerization rates (<15%), regardless of the addition of Ag_3_PO_4_. This suggests that isomerization of the product would not be a main reason for low stereoselectivity, and the enhancing effect of the silver salt on stereoselectivity is not caused by suppressing the isomerization of the initial products.

**Scheme 2:**
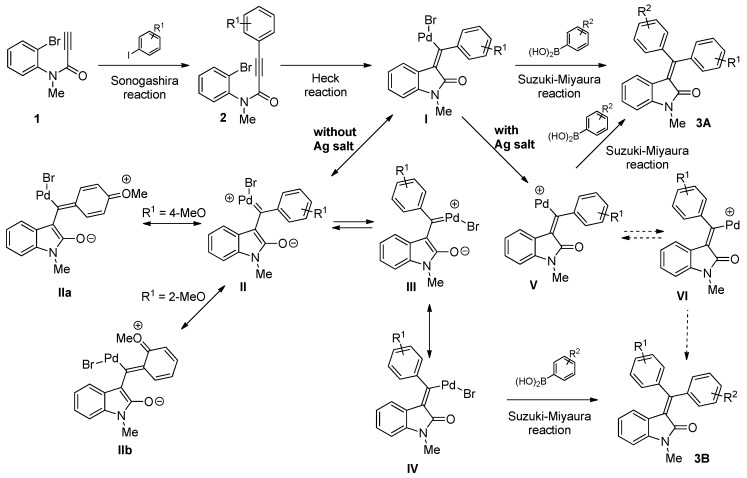
Proposed mechanisms of domino multicomponent reactions (MCRs) and isomerization.

Based on all of the above results, we propose a mechanism for our domino MCRs and isomerization (Scheme 2). The domino reaction begins with the Sonogashira reaction of propiolamide **1** with aryl iodide to afford adduct **2**. The fact that the biphenyl byproduct **4** was not detected under microwave-assisted conditions demonstrates that the Sonogashira reaction is more favorable than the Suzuki-Miyaura reaction between aryl iodide and arylboronic acid. In the second step, the bromo substituent of **2** reacts with Pd catalyst to produce vinyl palladium intermediate **I**, the stereochemistry of which would be the *E*-configuration due to the *syn*-addition mechanism of the migratory insertion step [17]. Without the silver salt, isomerization of **I** to **IV** probably occurred via a zwitterionic palladium carbenoid intermediate **II**/**III** [18,19]. This isomerization mechanism is supported by low stereoselectivity of the reaction with 2-methoxy- and 4-methoxyphenyl iodide. Both substrates could have additional resonance structures (**IIa** or **IIb**), which increase the stability of **II** to facilitate the isomerization rate between **I** and **IV**. The last step of the reaction is a Suzuki-Miyaura reaction of **I** or **IV** with arylboronic acid to give product **3A** or **3B,** respectively. Addition of the silver salt changes the catalytic pathway of the palladium-catalyzed reaction from neutral to cationic to give the cationic vinyl palladium intermediate **V** [16,20]. The cationic character on the palladium of **V** decreases the possibility of isomerization from **V** to **VI**, probably due to the instability of the corresponding zwitterionic palladium carbenoid species. Thus, **3A** would mainly be obtained by Suzuki-Miyaura reaction of **V**. Another benefit achieved by the addition of silver salt is the enhancing effect on reaction rate. We already discussed that the silver salt seems to facilitate the last Suzuki-Miyaura step (Table 5). In addition, our previous work proved that the silver salt was an effective additive to also increase the rate of Sonogashira reaction [9]. Thus, microwave irradiation and the silver salt are key factors for the current domino MCRs.

## 3. Experimental Section

### 3.1. General Information

Microwave reactions were conducted in a Biotage Initiator^+^ microwave reactor (Biotage AB, Uppsala, Sweden). The wattage was automatically adjusted to maintain the desired temperature for the desired period of time. All reactions were performed under an argon atmosphere with dry solvents, unless otherwise stated. Dry methylene chloride (CH_2_Cl_2_) tetrahydrofuran (THF) and acetonitrile (CH_3_CN) were obtained from an Ultimate Solvent Purification System (JC Meyer Solvent System, Laguna Beach, CA, USA). All commercially available reagents were purchased and used without further purification. Reactions were monitored by thin-layer chromatography (TLC) on silica gel plates (Merck TLC Silica Gel 60 F254, Darmstadt, Germany) using UV light, PMA (an ethanolic solution of phosphomolybdic acid) or ANIS (an ethanolic solution of *p*-anisaldehyde) as visualizing agent. Purification of products was conducted by column chromatography through silica gel 60 (0.060–0.200 mm). NMR spectra were obtained on Bruker AVANCE III 500 MHz (Bruker Corporation, Billerica, MA, USA) using residual undeuterated solvent or tetramethylsilane (TMS) as an internal reference. Stereochemistry of products was assigned by careful 1D- and 2D-NMR studies (see supporting information). High-resolution mass spectra (HR-MS) were recorded on an Agilent 6530 Q-TOF (Agilent, Santa Clara, CA, USA) using ESI (electrospray ionization) or a JEOL JMS-700 (JEOL, Tokyo, Japan) using EI (electron impact) or FAB (fast atom bombardment).

### 3.2. Preparation of Substrates ***1*** and ***6***

*N-(2-Bromophenyl)-N-methylpropiolamide* (**1**) [21]: To a stirred suspension of NaH (44 mg, 60% in mineral oil, 1.1 mmol, 1.1 equiv.) in THF (5.0 mL) was added a solution of *N*-(2-bromophenyl)propiolamide [8] (**6a**, 224 mg, 1.0 mmol, 1.0 equiv.) in THF (5 mL) at 0 °C. After 30 min stirring, MeI (0.08 mL, 1.3 mmol, 1.3 equiv.) was added dropwise at the same temperature. Then, the temperature was gradually raised to 25 °C. The mixture was stirred for 6 h at 25 °C and diluted with sat. aq. NH_4_Cl (50 mL). The mixture was extracted with EtOAc (50 mL × 2). The combined organic layer was dried (Na_2_SO_4_), filtered, and concentrated under reduced pressure. The crude residue was purified by column chromatography (silica gel, hexanes−EtOAc 5:1) to yield *N*-methylpropiolamide **1** (212 mg, 89% yield) as an off-white solid; m.p. = 73.2 °C (lit. [21] 88–89 °C); *R*_f_ = 0.32 (silica gel, hexanes−EtOAc 4:1); IR (film) 3221, 2107, 1646, 1372, 763 cm^−1^; ^1^H-NMR (CDCl_3_, 7:1 atropisomeric mixture, major peaks): δ 7.68 (dd, *J* = 8.0, 1.0 Hz, 1H), 7.41–7.21 (m, 3H), 3.25 (s, 3H), 2.73 (s, 1H) ppm; ^13^C-NMR (CDCl_3_): δ 153.3, 141.7, 133.9, 133.8, 130.5, 130.4, 129.9, 129.3, 128.9, 128.8, 123.8, 80.0, 78.9, 76.1, 39.0, 35.4 ppm; HRMS (ESI-TOF): calcd. for C_10_H_8_^79^BrNO [M + H^+^]: 237.9868, found 237.9872.

*N-Benzyl-N-(2-bromophenyl)propiolamide* (**6b**): To a stirred suspension of NaH (22 mg, 60% in mineral oil, 0.54 mmol, 1.2 equiv.) and tetrabutylammonium iodide (TBAI, 33 mg, 0.89 mmol, 2.0 equiv.) in DMF (2.0 mL) was added a solution of *N*-(2-bromophenyl)propiolamide [8] (**6a**, 100 mg, 0.45 mmol, 1.0 equiv.) in DMF (5 mL) at 0 °C. After 30 min stirring, BnBr (0.11 mL, 1.3 mmol, 2.0 equiv.) was added dropwise at the same temperature. The mixture was stirred for additional 30 min at 0 °C and diluted with Et_2_O (50 mL) and sat. aq. NH_4_Cl (1 mL). The organic layer was separated and washed with water (10 mL × 3). The organic layer was dried (Na_2_SO_4_), filtered, and concentrated under reduced pressure. The crude residue was purified by column chromatography (silica gel, hexanes−EtOAc 6:1) to yield *N*-benzylpropiolamide **6b** (92 mg, 65% yield) as a white solid; m.p. = 52.5 °C; *R*_f_ = 0.3 (silica gel, hexanes−EtOAc 5:1); IR (film) 3214, 3064, 2106, 1640, 722, 697, 697 cm^−1^; ^1^H-NMR (CDCl_3_, 7:1 atropisomeric mixture, major peaks) δ 7.67 (dd, *J* = 7.8, 1.8 Hz, 1H), 7.28–7.26 (m, 3H), 7.24–7.19 (m, 4H), 6.84 (dd, *J* = 12.9, 6.9 Hz, 1H), 5.58 (d, *J* = 14.3 Hz, 1H), 4.16 (d, *J* = 14.1 Hz, 1H), 2.73 (s, 1H) ppm; ^13^C-NMR (CDCl_3_) δ 153.3, 139.7, 136.0, 133.7, 132.1, 130.4, 129.6, 128.9, 128.8, 128.7, 128.1, 128.0, 124.2, 79.2, 76.2, 51.2; HRMS (EI): calcd. for C_16_H_12_^79^BrNO [M^+^]: 313.0102, found 313.0099.

*N-(2-Bromobenzyl)-N-methylpropiolamide* (**6c**): To a stirred solution of commercially available 1-(2-bromophenyl)-*N*-methylmethanamine (565 mg, 2.8 mmol, 1.0 equiv.) and propiolic acid (0.21 mL, 3.4 mmol, 1.2 equiv.) in CH_2_Cl_2_ (30 mL) was added DCC (700 mg, 3.4 mmol, 1.2 equiv.) at 0 °C. Then, the reaction temperature was raised to r.t. After 1.5 h, the reaction mixture was diluted with CH_2_Cl_2_ (200 mL), and then washed with aq. 2N-HCl (5 mL) and sat. aq. NaHCO_3_ (20 mL). The organic layer was dried (Na_2_SO_4_), filtered, and concentrated under reduced pressure. The crude residue was purified by column chromatography (silica gel, hexanes−EtOAc 3:1) to yield **6c** (675 mg, 95% yield) as an off-white solid; m.p. = 53.1 °C; *R*_f_ = 0.3 (silica gel, hexanes−EtOAc 2:1); IR (film) 3283, 3215, 3061, 2926, 2107, 1644, 1401 cm^−1^; ^1^H-NMR (CDCl_3_, 1.3:1 atropisomeric mixture): δ = 7.59 (d, *J* = 8.0 Hz, 1H, *major*), 7.56 (d, *J* = 8.0 Hz, 1H, *minor*), 7.35 (t, *J* = 7.5 Hz, 1H, *major*), 7.30 (t, *J* = 7.5 Hz, 1H, *minor*), 7.19 (t, *J* = 7.8 Hz, 2H, *major*), 7.16 (d, *J* = 7.8 Hz, 2H, *minor*), 4.91 (s, 2H, *major*), 4.75 (s, 2H, *minor*), 3.09 (s, 1H, *major*), 3.20 (s, 1H, *minor*), 2.94 (s, 3H, *major*), 3.18 (s, 3H, *minor*), ppm; ^13^C-NMR (CDCl_3_): δ = 154.4 (*major*), 154.2 (*minor*), 135.3 (*major*), 135.3 (*minor*), 133.5 (*major*), 133.3 (*minor*), 129.8 (*major*), 129.6 (*minor*), 129.5 (*minor*), 128.4 (*major*), 128.3 (*major*), 128.2 (*minor*), 80.0 (*minor*), 79.4 (*major*), 76.0 (*minor*), 75.9 (*major*), 54.9 (*major*), 50.0 (*minor*), 36.5 (*minor*), 32.7 (*major*) ppm; HRMS (EI): calcd. for C_23_H_18_ClNO [M^+^]: 250.9946, found 250.9944.

*2-Bromophenyl propiolate* (**6d**): To a stirred solution of 2-bromophenol (124 mg, 0.717 mmol), propiolic acid (0.665 mL, 1.08 mmol, 1.5 equiv.) and 4-(dimethylamino)pyridine (8.8 mg, 0.072 mmol, 0.1 equiv.) in CH_2_Cl_2_ (3.5 mL) was added DCC (222 mg, 1.08 mmol, 1.5 equiv.) at 0 °C. After 30 min the reaction mixture was diluted with CH_2_Cl_2_ (60 mL) and sat. aq. NaHCO_3_ (20 mL). The organic layer was separated, dried (Na_2_SO_4_), filtered, and concentrated under reduced pressure. The crude residue was purified by column chromatography (silica gel, hexanes−EtOAc 20:1) to yield **6d** (115 mg, 71% yield) as a colorless oil; *R*_f_ = 0.25 (silica gel, hexanes−EtOAc 20:1); IR (film) 3280, 1741, 1470, 1206, 752 cm^−1^; ^1^H-NMR (CDCl_3_): δ = 7.64 (dd, *J* = 7.8, 1.4 Hz, 1H), 7.37–7.34 (m, 1H), 7.19–7.16 (m, 2H), 3.14 (s, 1H) ppm; ^13^C-NMR (CDCl_3_): δ = 150.1, 147.3, 133.7, 128.8, 128.3, 123.6, 115.9, 77.7, 73.9 ppm; HRMS (EI): calcd. for C_9_H_5_BrO_2_ [M^+^]: 223.9473, found 223.9472.

### 3.3. General Procedure for Microwave-Assisted Reactions

A microwave reaction vial was charged with **1** or **6** (0.15 mmol, 1.0 equiv.), aryl iodide (0.17 mmol, 1.1 equiv.), aryl boronic acid (0.18 mmol, 1.2 equiv.), CuI (0.0075 mmol, 5 mol %), NaOAc (0.45 mmol, 3.0 equiv.), Pd(PPh_3_)_4_ (0.015 mmol, 10 mol %), Ag_3_PO_4_ (0.17 mmol, 1.1 equiv.) and DMF (3 mL). The reaction vial was sealed and exposed to microwave irradiation conditions with indicated time and temperature (150 °C, 10 min; representative conditions). The mixture was cooled to 25 °C and diluted with EtOAc (100 mL). Organic layer was washed with H_2_O (20 mL × 3) and brine (20 mL), then dried (Na_2_SO_4_), filtered and concentrated under reduced pressure. The crude residue was purified by column chromatography (silica gel, hexane:EtOAc) to yield 3-(diarylmethylene)oxindoles **3** or **7**.

*3-(Diphenylmethylene)-1-methylindolin-2-one* (**3a**) [22]: Yellow solid; m.p. = 165.9 °C (lit. [22] 153.2–154.9 °C); *R*_f_ = 0.33 (silica gel, hexanes−EtOAc 4:1); IR (film) 3054, 2923, 1700, 1606, 1469 cm^−1^; ^1^H-NMR (CDCl_3_): δ = 7.45−7.41 (m, 3H), 7.37–7.32 (m, 7H), 7.17 (td, *J* = 7.5, 1.0 Hz, 1H), 6.76 (d, *J* = 8.0 Hz, 1H), 6.68 (td, *J* = 7.5, 1.0 Hz, 1H), 6.42 (dd, *J* = 7.5, 1.0 Hz, 1H), 3.21 (s, 3H) ppm; ^13^C-NMR (CDCl_3_): δ = 167.0, 154.7, 143.5, 141.5, 140.1, 130.1, 129.5, 129.3, 129.2, 129.1, 128.9, 128.0, 124.4, 123.4, 123.3, 121.5, 107.8, 26.0 ppm; HRMS (ESI-TOF): calcd. for C_22_H_17_NO [M + H^+^]: 312.1388, found 312.1394.

*3-(Bis(4-methoxyphenyl)methylene)-1-methylindolin-2-one* (**3b**): Brown solid; m.p. = 192.2 °C; *R*_f_ = 0.2 (silica gel, hexanes−EtOAc 4:1); IR (film) 3017, 1691, 1603, 1251 cm^−1^; ^1^H-NMR (CDCl_3_): δ = 7.29–7.24 (m, 4H), 7.14 (td, *J* = 8.0, 1.5 Hz, 1H), 6.93 (d, *J* = 9.0 Hz, 2H), 6.88 (d, *J* = 9.0 Hz, 2H), 6.77 (d, *J* = 8.0 Hz, 1H), 6.70 (td, *J* = 8.0, 1.5 Hz, 1H), 6.57 (dd, *J* = 7.5, 0.5 Hz, 1H), 3.88 (s, 3H), 3.84 (s, 3H), 3.22 (s, 3H) ppm; ^13^C-NMR (CDCl_3_): δ = 167.3, 161.0, 160.8, 155.1, 142.8, 133.9, 132.9, 132.4, 132.1, 128.0, 124.3, 122.7, 122.5, 121.3, 114.2, 113.2, 107.7, 55.5, 55.4, 26.0 ppm; HRMS (ESI-TOF): calcd. for C_24_H_21_NO_3_ [M + H^+^]: 372.1600, found 372.1611.

*3-(Bis(4-chlorophenyl)methylene)-1-methylindolin-2-one* (**3c**): Yellow solid; m.p. = 134.4 °C; *R*_f_ = 0.33 (silica gel, hexanes−EtOAc 4:1); IR (film) 3054, 2927, 1699, 1606, 1486, 1089 cm^−1^; ^1^H-NMR (CDCl_3_): δ = 7.41 (dd, *J* = 7.0, 2.0 Hz, 2H), 7.33 (dd, *J* = 7.0, 2.0 Hz, 2H), 7.26–7.20 (m, 5H), 6.78 (d, *J* = 8.0 Hz, 1H), 6.73 (td, *J* = 7.5, 1.0 Hz, 1H), 6.52 (d, *J* = 7.5 Hz, 1H), 3.20 (s, 3H) ppm; ^13^C-NMR (CDCl_3_): δ = 166.7, 151.4, 143.5, 139.3, 138.0, 135.7, 135.6, 131.7, 131.1, 129.5, 129.4, 128.3, 125.1, 123.2, 122.8, 121.7, 108.1, 26.0 ppm; HRMS (ESI-TOF): calcd. for C_22_H_15_Cl_2_NO [M + H^+^]: 380.0609, found 380.0612.

*3-(Bis(4-nitrophenyl)methylene)-1-methylindolin-2-one* (**3d**): Brown solid; m.p. = 244.5 °C; *R*_f_ = 0.21 (silica gel, hexanes−EtOAc 3:1); IR (film) 3073, 1703, 1601, 1517, 1486, 1344, 1098 cm^−1^; ^1^H-NMR (CDCl_3_): δ = 8.34 (dd, *J* = 7.0, 2.0 Hz, 2H), 8.24 (dd, *J* = 7.0, 2.0 Hz, 2H), 7.56 (dd, *J* = 7.0, 2.0 Hz, 2H), 7.48 (dd, *J* = 7.0, 2.0 Hz, 2H), 7.29 (dd, *J* = 7.5, 1.0 Hz, 1H), 6.83 (d, *J* = 8.0 Hz, 1H), 6.76 (td, *J* = 8.0, 1.0 Hz, 1H), 6.45 (d, *J* = 7.5, 1H), 3.20 (s, 3H) ppm; ^13^C-NMR (CDCl_3_): δ = 166.0, 148.4, 148.1, 147.1, 146.3, 145.6, 144.3, 130.9, 130.6, 130.4, 127.5, 124.8, 123.63, 123.61, 122.2, 121.4, 108.7, 26.1; HRMS (ESI-TOF): calcd. for C_22_H_15_ N_3_O_5_ [M + H^+^]: 402.1092, found 402.1097.

*(E)-3-((4-Methoxyphenyl)(phenyl)methylene)-1-methylindolin-2-one* (**(*E*)-3e**) [22]: Yellow solid; m.p. = 162.7 °C (lit. [22] 163.8–164.3 °C); *R*_f_ = 0.26 (silica gel, hexanes−EtOAc 4:1); IR (film) 3053, 2927, 1696, 1603, 1508, 1469, 1250 cm^−1^; ^1^H-NMR (CDCl_3_): δ = 7.39–7.25 (m, 7H), 7.17 (td, *J* = 7.5, 1.5 Hz, 1H), 6.93 (dd, *J* = 7.0, 2.0 Hz, 2H), 6.78–6.67 (m, 3H), 3.87 (s, 3H), 3.20 (s, 3H) ppm; ^13^C-NMR (CDCl_3_): δ = 167.1, 160.8, 154.9, 143.3, 140.6, 133.6, 132.7, 131.7, 130.5, 129.3, 129.0, 128.6, 127.9, 122.9, 121.4, 114.3, 107.8, 55.5, 26.0 ppm; HRMS (ESI-TOF): calcd. for C_23_H_19_NO_2_ [M + H^+^]: 342.1494, found 342.1498.

*(Z)-3-((4-Methoxyphenyl)(phenyl)methylene)-1-methylindolin-2-one* (**(*Z*)-3e**) [23]: Yellow solid; m.p. = 153.0 °C (lit. [23] 163.8–164.3 °C); *R*_f_ = 0.24 (silica gel, hexanes−EtOAc 4:1); IR (film) 2931, 1696, 1604, 1250 cm^−1^; ^1^H-NMR (CDCl_3_): δ = 7.46–7.40 (m, 3H), 7.32–7.29 (m, 4H), 7.15 (t, *J* = 13.0 Hz, 1H), 6.88 (dd, *J* = 7.0, 2.0 Hz, 2H), 6.76 (d, *J* = 8.0 Hz, 1H), 6.66 (t, *J* = 7.5 Hz, 1H), 6.32 (d, *J* = 7.5 Hz, 1H), 3.84 (s, 3H), 3.23 (s, 3H); ^13^C-NMR (CDCl_3_): δ = 167.1, 161.0, 154.9, 143.0, 141.7, 132.7, 131.9, 129.9, 129.3, 129.0, 128.4, 123.9, 123.0, 121.4, 113.2, 107.7, 55.4, 26.0 ppm; HRMS (ESI-TOF): calcd. for C_23_H_19_NO_2_ [M + H^+^]: 342.1494, found 342.1503.

*(E)-3-((4-Chlorophenyl)(phenyl)methylene)-1-methylindolin-2-one* (**(*E*)-3f**): Yellow solid; m.p. = 54.8 °C; *R*_f_ = 0.35 (silica gel, hexanes−EtOAc 4:1); ^1^H-NMR (CDCl_3_): δ = 7.41–7.18 (m, 9H), 7.19 (td, *J* = 7.5, 1.0 Hz, 1H), 6.78 (d, *J* = 7.5 Hz, 1H), 6.73 (td, *J* = 7.5, 1.0 Hz, 1H), 6.53 (dd, *J* = 7.5, 0.5 Hz, 1H), 3.20 (s, 3H) ppm; ^13^C-NMR (CDCl_3_): δ = 166.8, 153.0, 143.6, 139.8, 139.7, 135.5, 131.1, 130.2, 129.42, 129.37, 129.2, 128.1, 124.7, 123.2, 123.0, 121.6, 108.0, 26.0 ppm; HRMS (EI): calcd. for C_22_H_16_ClNO [M^+^]: 345.0920, found 345.0919.

*(Z)-3-((4-Chlorophenyl)(phenyl)methylene)-1-methylindolin-2-one* (**(*Z*)-3f**): Yellow solid; m.p. = 59.0 °C: *R*_f_ = 0.37 (silica gel, hexanes−EtOAc 4:1); IR (film) 3054, 1698, 1606, 1486, 1335, 1090 cm^−1^; ^1^H-NMR (CDCl_3_): δ = 7.48–7.43 (m, 3H), 7.35–7.27 (m, 6H), 7.17 (td, *J* = 7.5, 1.0 Hz, 1H), 6.77 (d, *J* = 8.0 Hz, 1H), 6.68 (td, *J* = 7.5, 1.0 Hz, 1H), 6.42 (dd, *J* = 7.5, 0.5 Hz, 1H), 3.20 (s, 3H) ppm; ^13^C-NMR (CDCl_3_): δ = 166.9, 153.0, 143.5, 141.0, 138.4, 135.3, 131.6, 129.51, 129.50, 129.2, 129.1, 128.2, 124.7, 123.4, 123.2, 121.6, 107.9, 26.0 ppm; HRMS (EI): calcd. for C_22_H_16_ClNO [M^+^]: 345.0920, found 345.0916.

*(E)-1-Methyl-3-((4-nitrophenyl)(phenyl)methylene)indolin-2-one* (**(*E*)-3g**) [22]: Yellow solid; m.p. = 201.2 °C (lit. [22] 198.8–200.8 °C); *R*_f_ = 0.25 (silica gel, hexanes−EtOAc 4:1); IR (film) 3056, 1701, 1604, 1519, 1469, 1345 cm^−1^; ^1^H-NMR (CDCl_3_): δ = 8.30 (t, *J* = 4.25 Hz, 2H), 7.54 (d, *J* = 9.0 Hz, 2H), 7.41–7.21 (m, 6H), 6.80 (d, *J* = 8.0 Hz, 1H), 6.71 (t, *J* = 7.75 Hz, 1H), 6.36 (d, *J* = 7.5 Hz, 1H), 3.21 (s, 3H) ppm; ^13^C-NMR (CDCl_3_): δ = 166.4, 150.9, 148.2, 147.9, 143.9, 138.8, 130.6, 130.0, 129.9, 129.7, 128.3, 125.7, 124.4, 123.3, 122.4, 121.8, 108.3, 26.1 ppm; HRMS (EI): calcd. for C_22_H_16_N_2_O_3_ [M^+^]: 356.1161, found 356.1161.

*(Z)-1-Methyl-3-((4-nitrophenyl)(phenyl)methylene)indolin-2-one* (**(*Z*)-3g**): Yellow solid; m.p. = 186.6 °C; *R*_f_ = 0.28 (silica gel, hexanes−EtOAc 4:1); IR (film) 3057, 1699, 1603, 1510, 1342 cm^−1^; ^1^H-NMR (CDCl_3_): δ = 8.22 (dd, *J* = 7.0, 1.5 Hz, 2H), 7.50–7.44 (m, 5H), 7.32 (dd, *J* = 8.0, 2.0 Hz, 2H), 7.22 (t, *J* = 7.5 Hz, 1H), 6.80 (d, *J* = 8.0 Hz, 1H), 6.73 (t, *J* = 7.75 Hz, 1H), 6.52 (d, *J* = 8.0 Hz, 1H), 3.20 (s, 3H) ppm; ^13^C-NMR (CDCl_3_): δ = 166.6, 150.8, 147.8, 147.0, 143.9, 140.0, 130.7, 129.9, 129.8, 129.4, 129.2, 126.1, 123.7, 123.4, 122.4, 121.9, 108.2, 26.0 ppm; HRMS (ESI-TOF): calcd. for C_22_H_16_N_2_O_3_ [M + H^+^]: 357.1239, found 357.1249.

*(E)-4-((1-Methyl-2-oxoindolin-3-ylidene)(phenyl)methyl)phenyl acetate* (**(*E*)-3h**): Yellow solid; m.p. = 137.0 °C; *R*_f_ = 0.28 (silica gel, hexanes−EtOAc 4:1); IR (film) 3053, 2925, 1700, 1605, 1195 cm^−1^; ^1^H-NMR (CDCl_3_): δ = 7.39–7.36 (m, 3H), 7.35–7.34 (m, 2H), 7.33–7.31 (m, 2H), 7.20–7.19 (m, 1H), 7.18–7.16 (m, 2H), 6.77 (d, *J* = 7.8 Hz, 1H), 6.71 (td, *J* = 7.7, 0.8 Hz, 1H), 6.54 (d, *J* = 7.7 Hz, 1H), 3.20 (s, 3H), 2.34 (s, 3H) ppm; ^13^C-NMR (CDCl_3_): δ = 169.2, 166.9, 153.6, 151.5, 143.5, 139.9, 138.7, 130.9, 130.2, 129.3, 129.0, 128.0, 124.6, 123.22, 123.20, 122.2, 121.6, 107.9, 26.0, 21.4 ppm; HRMS (EI): calcd. for C_24_H_19_NO_3_ [M^+^]: 369.1365, found 369.1366.

*(Z)-4-((1-Methyl-2-oxoindolin-3-ylidene)(phenyl)methyl)phenyl acetate* (**(*Z*)-3h**): Yellow solid; m.p. = 134.8 °C; *R*_f_ = 0.20 (silica gel, hexanes−EtOAc 4:1); IR (film) 3053, 2928, 1768, 1605, 1197, 1094 cm^−1^; ^1^H-NMR (CDCl_3_): δ = 7.46–7.41 (m, 3H), 7.37–7.35 (m, 2H), 7.32–7.30 (m, 2H), 7.16 (td, *J* = 7.7, 1.0 Hz, 1H), 7.11–7.08 (m, 2H), 6.77 (d, *J* = 7.7 Hz, 1H), 6.67 (td, *J* = 7.7, 0.8 Hz, 1H), 6.38 (d, *J* = 7.6 Hz, 1H), 3.21 (s, 3H), 2.30 (s, 3H) ppm; ^13^C-NMR (CDCl_3_): δ = 169.3, 166.9, 153.7, 151.5, 143.4, 141.2, 137.2, 131.7, 129.6, 129.4, 129.1, 128.9, 124.5, 123.4, 123.3, 121.6, 121.0, 107.8, 26.0, 21.4 ppm; HRMS (EI): calcd. for C_24_H_19_NO_3_ [M^+^]: 369.1365, found 369.1366.

*(E)-3-((3-Methoxyphenyl)(phenyl)methylene)-1-methylindolin-2-one* (**(*E*)-3i**): Yellow solid; m.p. = 53.0 °C; *R*_f_ = 0.26 (silica gel, hexanes−EtOAc 4:1); IR (film) 3287, 3055, 2937, 1700, 1604, 1469, 1094, 698 cm^−1^; ^1^H-NMR (CDCl_3_): δ = 7.37–7.33 (m, 6H), 7.16 (td, *J* = 7.7, 1.1 Hz, 1H), 6.98 (ddd, *J* = 8.3, 2.6, 0.9 Hz, 1H), 6.77 (dt, *J* = 9.9, 1.9 Hz, 1H), 6.84 (dd, *J* = 2.5, 1.6 Hz, 1H), 6.76 (d, *J* = 7.8 Hz, 1H), 6.69 (td, *J* = 7.7, 1.0 Hz, 1H), 6.48 (d, *J* = 7.3 Hz, 1H), 3.77 (s, 3H), 3.20 (s, 3H) ppm; ^13^C-NMR (CDCl_3_): δ = 166.9, 160.1, 154.4, 143.5, 142.7, 139.9, 130.2, 130.0, 129.2, 128.9, 128.0, 124.4, 123.5, 123.3, 121.7, 121.6, 115.0, 114.5, 107.8, 55.5, 26.0 ppm; HRMS (EI): calcd. for C_23_H_19_NO_2_ [M^+^]: 341.1416, found 341.1417.

*(Z)-3-((3-Methoxyphenyl)(phenyl)methylene)-1-methylindolin-2-one* (**(*Z*)-3i**): Yellow solid; m.p. = 118.2 °C; *R*_f_ = 0.21 (silica gel, hexanes−EtOAc 4:1); IR (film) 3535, 3055, 2927, 2214, 1700, 1604, 1096, 734 cm^−1^; ^1^H-NMR (CDCl_3_): δ = 7.43–7.41 (m, 3H), 7.33–7.29 (m, 3H), 7.15 (td, *J* = 7.7, 1.0 Hz, 1H), 6.95 (d, *J* = 7.8 Hz, 1H), 6.91 (dd, *J* = 8.3, 1.9 Hz, 1H), 6.83 (t, *J* = 1.9 Hz, 1H), 6.76 (d, *J* = 7.8 Hz, 1H), 6.66 (td, *J* = 7.7, 1.0 Hz, 1H), 6.42 (d, *J* = 7.6 Hz, 1H), 3.76 (s, 3H), 3.20 (s, 3H) ppm; ^13^C-NMR (CDCl_3_): δ = 166.8, 159.3, 154.3, 143.5, 141.5, 141.3, 129.31, 129.27, 129.1, 128.94, 128.93, 124.5, 123.3, 122.5, 121.5, 115.7, 114.4, 107.8, 55.4, 26.0 ppm; HRMS (EI): calcd. for C_23_H_19_NO_2_ [M^+^]: 341.1416, found 341.1422.

*(E)-3-((3-Chlorophenyl)(phenyl)methylene)-1-methylindolin-2-one* (**(*E*)-3j**): Yellow solid; m.p. = 130.2 °C; *R*_f_ = 0.25 (silica gel, hexanes−EtOAc 4:1); IR (film) 3055, 3016, 1699, 1470, 1095 cm^−1^; ^1^H-NMR (CDCl_3_): δ = 7.44–7.41 (m, 1H), 7.39–7.35 (m, 4H), 7.34–7.31 (m, 3H), 7.23 (dt, *J* = 9.8, 1.9 Hz, 1H), 7.18 (td, *J* = 7.7, 1.1 Hz, 1H), 6.78 (d, *J* = 7.7 Hz, 1H), 6.71 (td, *J* = 7.7, 0.7 Hz, 1H), 6.44 (d, *J* = 7.7 Hz, 1H), 3.20 (s, 3H) ppm; ^13^C-NMR (CDCl_3_): δ = 166.7, 152.5, 143.6, 143.1, 139.4, 135.1, 130.4, 130.0, 129.4, 129.34, 129.30, 128.1, 127.6, 125.0, 123.3, 122.9, 121.7, 108.0, 26.0 ppm; HRMS (EI): calcd. for C_22_H_16_ClNO [M^+^]: 345.0920, found 345.0920.

*(Z)-3-((3-Chlorophenyl)(phenyl)methylene)-1-methylindolin-2-one* (**(*Z*)-3j**): Yellow solid; m.p. = 143.2 °C; *R*_f_ = 0.31 (silica gel, hexanes−EtOAc 4:1); IR (film) 3055, 3016, 1699, 1607, 1470, 1098 cm^−1^; ^1^H-NMR (CDCl_3_): δ = 7.47–7.43 (m, 3H), 7.34–7.29 (m, 4H), 7.28–7.26 (m, 2H), 7.18 (td, *J* = 7.7, 1.1 Hz, 1H), 6.78 (d, *J* = 7.7 Hz, 1H), 6.68 (td, *J* = 7.7, 1.1 Hz, 1H), 6.44 (d, *J* = 7.7 Hz, 1H), 3.21 (s, 3H) ppm; ^13^C-NMR (CDCl_3_): δ = 166.7, 152.4, 143.6, 141.9, 140.7, 134.0, 129.8, 129.5, 129.3, 129.28, 129.21, 129.1, 129.0, 128.2, 125.1, 123.4, 122.9, 121.6, 107.9, 26.0 ppm; HRMS (EI): calcd. for C_22_H_16_ClNO [M^+^]: 345.0920, found 345.0918.

*(E)-1-Methyl-3-((3-nitrophenyl)(phenyl)methylene)indolin-2-one* (**(*E*)-3k**): Yellow solid; m.p. = 186.9 °C; *R*_f_ = 0.32 (silica gel, hexanes−EtOAc 2:1); IR (film) 3055, 3017, 1699, 1528, 1350, 1096 cm^−1^; ^1^H-NMR (CDCl_3_): δ = 8.33–8.30 (m, 1H), 8.19 (t, *J* = 1.9 Hz, 1H), 7.71 (dt, *J* = 10.3, 1.9 Hz, 1H), 7.64 (t, *J* = 7.9 Hz, 1H), 7.41–7.38 (m, 3H), 7.32–7.30 (m, 2H), 7.20 (td, *J* = 7.7, 1.1 Hz, 1H), 6.80 (d, *J* = 7.8 Hz, 1H), 6.67 (td, *J* = 7.7, 1.0 Hz, 1H), 6.32 (d, *J* = 7.7 Hz, 1H), 3.21 (s, 3H) ppm; ^13^C-NMR (CDCl_3_): δ = 166.4, 150.7, 148.9, 143.9, 143.0, 138.9, 135.6, 130.3, 130.0, 129.8, 129.7, 128.3, 125.8, 124.5, 124.0, 123.0, 122.4, 121.8, 108.3, 26.1 ppm; HRMS (EI): calcd. for C_22_H_16_N_2_O_3_ [M^+^]: 356.1161, found 356.1159.

*(Z)-1-Methyl-3-((3-nitrophenyl)(phenyl)methylene)indolin-2-one* (**(*Z*)-3k**): Yellow solid; m.p. = 168.1 °C; *R*_f_ = 0.28 (silica gel, hexanes−EtOAc 3:1); IR (film) 3055, 3017, 1698, 1527, 1348, 1094 cm^−1^; ^1^H-NMR (CDCl_3_): δ = 8.23–8.21 (m, 1H), 8.17–8.16 (m, 1H), 7.69 (d, *J* = 7.7 Hz, 1H), 7.53 (t, *J* = 8.0 Hz, 1H), 7.49–7.45 (m, 3H), 7.34–7.32 (m, 2H), 7.20 (td, *J* = 7.7, 0.9 Hz, 1H), 6.79 (d, *J* = 7.8 Hz, 1H), 6.70 (td, *J* = 7.7, 0.7 Hz, 1H), 6.49 (d, *J* = 7.7 Hz, 1H), 3.20 (s, 3H) ppm; ^13^C-NMR (CDCl_3_): δ = 166.6, 150.6, 148.2, 143.8, 141.7, 140.1, 136.1, 129.84, 129.80, 129.5, 129.3, 128.9, 126.0, 125.0, 123.7, 123.6, 122.5, 121.9, 108.1, 26.1 ppm; HRMS (EI): calcd. for C_22_H_16_N_2_O_3_ [M^+^]: 356.1161, found 356.1159.

*(E)-3-((2-Methoxyphenyl)(phenyl)methylene)-1-methylindolin-2-one* (**(*E*)-3l**): Yellow solid; m.p. = 188.5 °C; *R*_f_ = 0.33 (silica gel, hexanes−EtOAc 4:1); IR (film) 2918, 2849, 1701, 1604, 1094 cm^−1^; ^1^H-NMR (CDCl_3_): δ = 7.44–7.40 (m, 3H), 7.36–7.32 (m, 3H), 7.21 (dd, *J* = 7.5, 1.5 Hz, 1H), 7.16 (t, *J* = 7.7 Hz, 1H), 7.05 (t, *J* = 7.5 Hz, 1H), 7.00 (d, *J* = 8.3 Hz, 1H), 6.75 (d, *J* = 7.8 Hz, 1H), 6.69 (t, *J* = 7.7 Hz, 1H), 6.23 (d, *J* = 7.8 Hz, 1H), 3.63 (s, 3H), 3.19 (s, 3H) ppm; ^13^C-NMR (CDCl_3_): δ = 166.7, 156.4, 151.4, 143.4, 139.7, 130.5, 130.3, 129.6, 129.2, 128.7, 128.5, 127.6, 125.0, 123.5, 123.0, 121.7, 121.5, 112.2, 107.6, 55.9, 25.9 ppm; HRMS (EI): calcd. for C_23_H_19_NO_2_ [M^+^]: 341.1416, found 341.1419.

*(Z)-3-((2-Methoxyphenyl)(phenyl)methylene)-1-methylindolin-2-one* (**(*Z*)-3l**): Yellow solid; m.p. = 139.3 °C; *R*_f_ = 0.24 (silica gel, hexanes−EtOAc 4:1); IR (film) 3053, 2925, 2854, 1704, 1606, 1469, 1095 cm^−1^; ^1^H-NMR (CDCl_3_): δ = 7.40 (s, 5H), 7.34–7.31 (m, 1H), 7.19–7.14 (m, 2H), 6.97 (td, *J* = 7.5, 0.8 Hz, 1H), 6.93 (d, *J* = 8.3 Hz, 1H), 6.76 (d, *J* = 7.7 Hz, 1H), 6.68 (td, *J* = 7.7, 0.9 Hz, 1H), 6.52 (d, *J* = 7.7 Hz, 1H), 3.70 (s, 3H), 3.17 (s, 3H) ppm; ^13^C-NMR (CDCl_3_): δ = 166.8, 156.7, 150.6, 143.6, 140.9, 130.4, 129.7, 129.6, 128.9, 128.8, 128.7, 128.6, 125.5, 123.3, 122.8, 121.4, 120.7, 111.4, 107.7, 55.8, 25.9 ppm; HRMS (EI): calcd. for C_23_H_19_NO_2_ [M^+^]: 341.1416, found 341.1419.

*(E)-3-((2-Chlorophenyl)(phenyl)methylene)-1-methylindolin-2-one* (**(*E*)-3m**): Yellow solid; m.p. = 164.6 °C; *R*_f_ = 0.33 (silica gel, hexanes−EtOAc 4:1); IR (film) 3053, 2918, 1703, 1605, 1095 cm^−1^; ^1^H-NMR (CDCl_3_): δ = 7.53–7.51 (m, 1H), 7.49–7.47 (m, 2H), 7.41–7.36 (m, 5H), 7.34–7.32 (m, 1H), 7.18 (td, *J* = 7.7, 1.1 Hz, 1H), 6.77 (d, *J* = 7.7 Hz, 1H), 6.69 (td, *J* = 7.7, 1.0 Hz, 1H), 6.04 (d, *J* = 7.7 Hz, 1H), 3.21 (s, 3H) ppm; ^13^C-NMR (CDCl_3_): δ = 166.4, 150.1, 143.6, 140.0, 137.9, 132.5, 130.7, 130.0, 129.9, 129.7, 129.3, 129.1, 127.8, 125.7, 123.1, 123.0, 122.0, 107.9, 26.0 ppm; HRMS (EI): calcd. for C_22_H_16_ClNO [M^+^]: 345.0920, found 345.0921.

*(Z)-3-((2-Chlorophenyl)(phenyl)methylene)-1-methylindolin-2-one* (**(*Z*)-3m**): Yellow solid; m.p. = 199.5 °C; *R*_f_ = 0.34 (silica gel, hexanes−EtOAc 4:1); IR (film) 3054, 3017, 2931, 1706, 1606, 1099 cm^−1^; ^1^H-NMR (CDCl_3_): δ = 7.49–7.43 (m, 6H), 7.32–7.29 (m, 2H), 7.25–7.21 (m, 2H), 6.79 (d, *J* = 7.8 Hz, 1H), 6.75 (t, *J* = 7.6 Hz, 1H), 6.70 (d, *J* = 7.7 Hz, 1H), 3.19 (s, 3H) ppm; ^13^C-NMR (CDCl_3_): δ = 166.6, 149.3, 144.0, 140.1, 139.4, 132.3, 129.8, 129.6, 129.4, 129.3, 129.2, 129.0, 128.9, 127.1, 126.4, 123.5, 122.1, 121.6, 108.0, 26.0 ppm; HRMS (EI): calcd. for C_22_H_16_ClNO [M^+^]: 345.0920, found 345.0920.

*(Z)-1-Methyl-3-((2-nitrophenyl)(phenyl)methylene)indolin-2-one* (**(*Z*)-3n**): Yellow solid; m.p. = 206.6 °C; *R*_f_ = 0.27 (silica gel, hexanes−EtOAc 2:1); IR (film) 3063, 3021, 2930, 1700, 1522 cm^−1^; ^1^H-NMR (CDCl_3_): δ = 8.15 (dd, *J* = 8.2, 1.0 Hz, 1H), 7.66 (td, *J* = 7.6, 1.1 Hz, 1H), 7.54–7.51 (m, 2H), 7.43–7.40 (m, 5H), 7.24–7.21 (m, 1H), 6.78 (d, *J* = 7.7 Hz, 1H), 6.75–6.74 (m, 2H), 3.15 (s, 3H) ppm; ^13^C-NMR (CDCl_3_): δ = 166.8, 148.4, 147.7, 143.9, 138.1, 136.9, 133.7, 130.7, 129.7, 129.6, 129.0, 128.9, 125.2, 125.0, 123.4, 122.0, 121.8, 108.1, 26.0 ppm; HRMS (EI): calcd. for C_22_H_16_N_2_O_3_ [M^+^]: 356.1161, found 356.1163.

*(E)-1-Methyl-3-(phenyl(pyridin-3-yl)methylene)indolin-2-one* (**(*E*)-3p**): Yellow solid; m.p. = 159.0 °C; *R*_f_ = 0.24 (silica gel, hexanes−EtOAc 2:1); IR (film) 3732, 3052, 2926, 1700, 1470, 1095, 749, 698 cm^−1^; ^1^H-NMR (CDCl_3_): δ = 8.73 (brs, 2H), 7.62 (d, *J* = 7.8 Hz, 1H), 7.42–7.36 (m, 4H), 7.33–7.30 (m, 2H), 7.19 (td, *J* = 7.7, 1.1 Hz, 1H), 6.79 (d, *J* = 7.7 Hz, 1H), 6.70 (td, *J* = 7.7, 1.0 Hz, 1H), 6.42 (d, *J* = 7.5 Hz, 1H), 3.21 (s, 3H) ppm; ^13^C-NMR (CDCl_3_): δ = 166.5, 150.3, 150.0, 143.7, 139.4, 137.2, 130.1, 129.5, 129.5, 128.2, 125.7, 123.0, 122.8, 121.8, 108.1, 26.0 ppm; HRMS (EI): calcd. for C_21_H_16_N_2_O [M^+^]: 312.1263, found 312.1262.

*(Z)-1-Methyl-3-(phenyl(pyridin-3-yl)methylene)indolin-2-one* (**(*Z*)-3p**): Yellow oil; *R*_f_ = 0.12 (silica gel, hexanes−EtOAc 2:1); ^1^H-NMR (CDCl_3_): δ = 8.58 (d, *J* = 17.9 Hz, 2H), 7.65 (d, *J* = 7.8 Hz, 1H), 7.48–7.44 (m, 3H), 7.33–7.31 (m, 3H), 7.19 (td, *J* = 7.7, 1.1 Hz, 1H), 6.79 (d, *J* = 7.7 Hz, 1H), 6.69 (td, *J* = 7.7, 1.0 Hz, 1H), 6.47 (d, *J* = 7.7 Hz, 1H), 3.21 (s, 3H) ppm; ^13^C-NMR (CDCl_3_): δ = 166.9, 149.9, 143.7, 140.5, 137.4, 130.1, 129.7, 129.5, 129.4, 129.3, 125.6, 123.5, 122.8, 121.7, 108.0, 26.1 ppm: HRMS (FAB): calcd. for C_21_H_17_N_2_O [M + H^+^]: 313.1341, found 313.1342.

*(E)-3-(Benzo[b]thiophen-3-yl(phenyl)methylene)-1-methylindolin-2-one* (**(*E*)-3q**): Yellow solid; m.p. = 82.3 °C; *R*_f_ = 0.24 (silica gel, hexanes−EtOAc 4:1); IR (film) 3055, 3010, 2929, 1698, 1606, 1094 cm^−1^; ^1^H-NMR (CDCl_3_): δ = 7.93 (d, *J* = 8.1 Hz, 1H), 7.46–7.43 (m, 4H), 7.38–7.35 (m, 4H), 7.24 (t, *J* = 7.7 Hz, 1H), 7.14 (t, *J* = 7.7 Hz, 1H), 6.77 (d, *J* = 7.7 Hz, 1H), 6.60 (t, *J* = 7.7 Hz, 1H), 6.21 (d, *J* = 7.7 Hz, 1H), 3.23 (s, 3H) ppm; ^13^C-NMR (CDCl_3_): δ = 166.7, 147.4, 143.4, 140.5, 139.2, 137.1, 130.0, 129.5, 129.4, 129.0, 128.0, 127.7, 125.9, 125.0, 124.9, 123.6, 123.1, 123.0, 121.8, 107.8, 26.0 ppm; HRMS (EI): calcd. for C_24_H_17_NOS [M^+^]: 367.1031, found 367.1033.

*(Z)-3-(Benzo[b]thiophen-3-yl(phenyl)methylene)-1-methylindolin-2-one* (**(*Z*)-3q**): Yellow solid; m.p. = 74.5 °C; *R*_f_ = 0.29 (silica gel, hexanes−EtOAc 4:1); IR (film) 3026, 2920, 1701, 1089, 750 cm^−1^; ^1^H-NMR (CDCl_3_): δ = 7.86 (d, *J* = 8.1 Hz, 1H), 7.48–7.41 (m, 7H), 7.33–7.30 (m, 1H), 7.25–7.20 (m, 2H), 6.80 (d, *J* = 7.8 Hz, 1H), 6.72 (td, *J* = 7.7, 1.0 Hz, 1H), 6.66 (d, *J* = 7.1 Hz, 1H), 3.19 (s, 3H) ppm; ^13^C-NMR (CDCl_3_): δ = 166.6, 146.8, 143.6, 140.5, 140.0, 138.1, 136.4, 129.6, 129.4, 129.2, 129.1, 128.3, 126.1, 124.5, 124.4, 123.4, 123.0, 122.9, 122.8, 121.5, 107.9, 26.1 ppm; HRMS (EI): calcd. for C_24_H_17_NOS [M^+^]: 367.1031, found 367.1027.

*(E)-3-(Benzo[b]thiophen-2-yl(phenyl)methylene)-1-methylindolin-2-one* (**(*E*)-3r**): Yellow solid; m.p. = 173.7 °C; *R*_f_ = 0.27 (silica gel, hexanes:−EtOAc 4:1); IR (film) 3732, 3627, 2925, 2854, 1699, 1094, 747, 695 cm^−1^; ^1^H-NMR (CDCl_3_): δ = 7.84–7.79 (m, 2H), 7.50 (d, *J* = 0.4 Hz, 1H), 7.44–7.37 (m, 7H), 7.20 (td, *J* = 7.7, 1.2 Hz, 1H), 7.08 (dd, *J* = 7.8, 0.5 Hz, 1H), 6.79 (d, *J* = 7.8 Hz, 1H), 6.73 (td, *J* = 7.7, 1.0 Hz, 1H), 3.20 (s, 3H) ppm; ^13^C-NMR (CDCl_3_): δ = 166.6, 146.3, 143.7, 143.2, 141.8, 139.7, 139.6, 130.0, 129.7, 129.6, 128.1, 126.4, 126.1, 125.5, 124.9, 124.6, 123.5, 122.9, 122.7, 121.8, 108.0, 26.1 ppm; HRMS (EI): calcd. for C_24_H_17_NOS [M^+^]: 367.1031, found 367.1035.

*(Z)-3-(Benzo[b]thiophen-2-yl(phenyl)methylene)-1-methylindolin-2-one* (**(*Z*)-3r**): Yellow solid; m.p. = 160.7 °C; *R*_f_ = 0.39 (silica gel, hexanes−EtOAc 4:1); IR (film) 3733, 3054, 2926, 1694, 1470 1419, 1373, 1092, 745, 698 cm^−1^; ^1^H-NMR (CDCl_3_): δ = 7.77–7.75 (m, 3H), 7.55–7.48 (m, 3H), 7.41–7.38 (m, 2H), 7.33–7.31 (m, 2H), 7.14 (td, *J* = 7.7, 1.1 Hz, 1H), 6.77 (d, *J* = 7.7 Hz, 1H), 6.62 (td, *J* = 7.7, 1.0 Hz, 1H), 5.97 (dd, *J* = 7.7, 0.5 Hz, 1H), 3.28 (s, 3H) ppm; ^13^C-NMR (CDCl_3_): δ = 166.6, 146.1, 143.2, 142.5, 141.6, 141.4, 139.2, 130.4, 129.5, 129.3, 129.3, 129.0 125.7, 124.8, 124.7, 124.4, 123.9, 123.6, 122.2, 121.6, 107.8, 26.1 ppm; HRMS (EI): calcd. for C_24_H_17_NOS [M^+^]: 367.1031, found 367.1035.

*(E)-3-(Furan-3-yl(phenyl)methylene)-1-methylindolin-2-one* (**(*E*)-3s**): Yellow solid; m.p. = 122.9; *R*_f_ = 0.20 (silica gel, hexanes−EtOAc 4:1); IR (film) 3137, 3052, 2928, 1606, 1093, 740 cm^−1^; ^1^H-NMR (CDCl_3_): δ = 7.53 (t, *J* = 1.7 Hz, 1H), 7.40–7.34 (m, 7H), 7.21 (td, *J* = 7.7, 1.1 Hz, 1H), 6.85 (td, *J* = 7.7, 1.1 Hz, 1H), 6.79 (d, *J* = 7.7 Hz, 1H), 6.54 (dd, *J* = 1.8, 0.8 Hz, 1H), 3.18 (s, 3H) ppm; ^13^C-NMR (CDCl_3_): δ = 166.9, 145.2, 144.7, 143.6, 143.4, 140.2, 130.2, 129.4, 128.9, 128.0, 126.8, 124.0, 123.2, 123.1, 121.5, 111.9, 107.9, 26.0 ppm; HRMS (EI): calcd. for C_20_H_15_NO_2_ [M^+^]: 301.1103, found 301.1104.

*(Z)-3-(Furan-3-yl(phenyl)methylene)-1-methylindolin-2-one* (**(*Z*)-3s**): Yellow solid; m.p. = 132.5; *R*_f_ = 0.37 (silica gel, hexanes−EtOAc 4:1); IR (film) 3139, 3053, 2927, 1692, 1099, 733 cm^−1^; ^1^H-NMR (CDCl_3_): δ = 7.73–7.72 (m, 1H), 7.50–7.47 (m, 3H), 7.40 (t, *J* = 1.7 Hz, 1H), 7.32–7.31 (m, 2H), 7.10 (td, *J* = 7.7, 1.1 Hz, 1H), 6.87 (dd, *J* = 1.9, 0.8 Hz, 1H), 6.75 (d, *J* = 7.7 Hz, 1H), 6.59 (td, *J* = 7.7, 1.1 Hz, 1H), 3.28 (s, 3H) ppm; ^13^C-NMR (CDCl_3_): δ = 166.9, 148.0, 144.8, 142.8, 142.1, 141.4, 129.2, 129.1, 129.0, 128.2, 125.7, 123.9, 123.5, 122.9, 121.4, 113.1, 107.6, 26.1 ppm; HRMS (EI): calcd. for C_20_H_15_NO_2_ [M^+^]: 301.1103, found 301.1102.

*4,4′-Dinitro-1,1′-biphenyl* (**4d**) [24]: Yellow solid; m.p. = 235.7 °C (lit. [24] 235–237 °C); *R*_f_ = 0.31 (silica gel, hexanes−EtOAc 4:1); ^1^H-NMR (CDCl_3_): δ = 8.36 (d, *J* = 8.3 Hz, 4H), 7.79 (d, *J* = 8.3 Hz, 4H) ppm; ^13^C-NMR (CDCl_3_): δ = 148.2, 145.1, 128.5, 124.5 ppm.

*4-Methoxy-1,1′-biphenyl* (**4e**) [25]: White solid, m.p. = 86.1 °C (lit. [25] 86–88 °C); *R*_f_ = 0.36 (silica gel, hexanes−EtOAc 10:1); ^1^H-NMR (CDCl_3_): δ = 7.57–7.52 (m, 4H), 7.44–7.41 (m, 2H), 7.33–7.30 (m, 1H), 7.00–6.97 (m, 2H), 3.86 (s, 3H) ppm; ^13^C-NMR (CDCl_3_): δ = 159.2, 140.9, 133.9, 128.9, 128.3, 126.9, 126.8, 114.3, 55.5 ppm.

*4-Chloro-1,1'-biphenyl* (**4f**) [26]: White solid, m.p. = 69.8 °C (lit. [26] 71–73 °C); *R*_f_ = 0.46 (silica gel, hexane); ^1^H-NMR (CDCl_3_): δ = 7.56–7.54 (m, 2H), 7.53–7.50 (m, 2H), 7.46–7.43 (m, 2H), 7.42–7.39 (m, 2H), 7.38–7.34 (m, 1H) ppm; ^13^C-NMR (CDCl_3_): δ = 140.2, 139.8, 133.5, 129.1, 129.0, 128.5, 127.7, 127.1 ppm.

*4-Nitro-1,1′-biphenyl* (**4g**) [27]: Pale yellow solid; m.p. = 112.8 °C (lit. [27] 112–113 °C); *R*_f_ = 0.36 (silica gel, hexanes−EtOAc 10:1); ^1^H-NMR (CDCl_3_): δ = 8.32–8.29 (m, 2H), 7.76–7.73 (m, 2H), 7.64–7.62 (m, 2H), 7.52–7.49 (m, 2H), 7.47–7.44 (m, 1H) ppm; ^13^C-NMR (CDCl_3_): δ = 147.8, 147.2, 138.9, 129.3, 129.1, 127.9, 127.5, 124.3 ppm.

*9-Methyl-4-phenylpyrano*[2,3-b]*indol-2(9H)-one* (**5**): Yellow solid; m.p. = 146.2 °C; *R*_f_ = 0.44 (silica gel, hexanes−EtOAc 4:1); IR (film) 3055, 2924, 2853, 1732, 1527, 1470 cm^−1^; ^1^H-NMR (CDCl_3_): 7.65–7.64 (m, 2H), 7.57–7.54 (m, 3H), 7.35 (dd, *J* = 13.7, 8.1 Hz, 2H), 7.28 (td, *J* = 7.7, 1.1 Hz, 1H), 7.12–7.09 (m, 1H), 5.91 (s, 1H), 3.85 (s, 3H) ppm; ^13^C-NMR (CDCl_3_): δ = 161.0, 157.2, 153.2, 136.9, 134.4, 130.1, 128.9, 128.0, 123.3, 122.0, 121.0, 120.2, 109.7, 101.4, 95.1, 28.3 ppm; HRMS (EI): calcd. for C_18_H_13_NO_2_ [M^+^]: 275.0946, found 275.0948.

*(E)-3-((4-Chlorophenyl)(phenyl)methylene)indolin-2-one* (**(*E*)-7a**): Dark orange solid; m.p. = 83.5 °C; *R*_f_ = 0.31 (silica gel, hexanes−EtOAc 3:2); IR (film) 32,436, 2954, 2923, 2851, 1698, 1615, 1465 cm^−1^; ^1^H-NMR (CDCl_3_): δ = 8.60 (brs, 1H), 7.46–7.26 (m, 9H), 7.07 (td, *J* = 7.7, 1.0 Hz, 1H), 6.70–6.61 (m, 2H), 6.47 (d, *J* = 7.8 Hz, 1H) ppm; ^13^C-NMR (CDCl_3_): 153.6, 140.6, 139.8, 139.5, 135.6, 131.2, 130.5, 129.6, 129.4, 129.2, 128.1, 124.8, 123.9, 123.4, 121.6, 109.6 ppm; HRMS (EI): calcd. for C_23_H_18_ClNO [M^+^]: 331.0764, found 331.0762.

*(Z)-3-((4-Chlorophenyl)(phenyl)methylene)indolin-2-one one* (**(*Z*)-7a**): Yellow solid; m.p. = 214.8 °C; *R*_f_ = 0.26 (silica gel, hexanes−EtOAc 2:1); IR (film) 3247, 2953, 2923, 2853, 1732, 1699, 1485 cm^−1^; ^1^H-NMR (CDCl_3_): δ = 8.22 (brs, 1H), 7.49–6.43 (m, 2H), 7.33–7.30 (m, 2H), 7.08 (td, *J* = 7.7, 1.1 Hz, 1H), 6.73 (d, *J* = 7.7 Hz, 1H), 6.63 (td, *J* = 7.7, 1.0 Hz, 1H), 6.37 (d, *J* = 7.8 Hz, 1H) ppm; ^13^C-NMR (CDCl_3_): δ = 168.4, 153.5, 141.0, 140.7, 138.2, 135.5, 132.0, 129.6, 129.6, 129.2, 129.2, 128.2, 124.9, 124.0, 123.6, 121.6, 109.7 ppm; HRMS (EI): calcd. for C_23_H_18_ClNO [M^+^]: 331.0764, found 331.0762.

*(E)-1-Benzyl-3-((4-chlorophenyl)(phenyl)methylene)indolin-2-one* (**(*E*)-7b**): Yellow solid; m.p. = 144.4 °C; *R*_f_ = 0.23 (silica gel, hexanes−EtOAc 10:1); IR (film) 3059, 2923, 2852, 1699, 1345 cm^−1^; ^1^H-NMR (CD_2_Cl_2_): δ = 7.47–7.44 (m, 2H), 7.42–7.31 (m, 11H), 7.30–7.26 (m, 1H), 7.08 (td, *J* = 7.7, 1.0 Hz, 1H), 7.47–7.43 (m, 2H), 6.53 (d, *J* = 7.7 Hz, 1H), 4.91 (s, 2H) ppm; ^13^C-NMR (CD_2_Cl_2_): δ = 166.8, 153.2, 143.0, 140.24, 140.23, 136.9, 135.6, 131.3, 130.6, 129.6, 129.5, 129.3, 129.0, 128.2, 127.8, 127.7, 124.9, 123.53, 123.46, 121.8, 109.0, 43.7 ppm; HRMS (EI): calcd. for C_23_H_18_ClNO [M^+^]: 421.1233, found 421.1231.

*(Z)-1-Benzyl-3-((4-chlorophenyl)(phenyl)methylene)indolin-2-one* (**(*Z*)-7b**): Orange solid; m.p. = 132.8 °C; *R*_f_ = 0.28 (silica gel, hexanes−EtOAc 10:1); IR (film) 3056, 2922, 2853, 1698, 1345 cm^−1^; ^1^H-NMR (CD_2_Cl_2_): δ = 7.49–7.44 (m, 3H), 7.35–7.31 (m, 10H), 7.08–7.05 (m, 1H), 7.05 (td, *J* = 7.7, 1.1 Hz, 1H), 6.69 (d, *J* = 7.8 Hz, 1H), 6.62 (td, *J* = 7.7, 1.0 Hz, 1H), 6.39 (dd, *J* = 7.7, 0.5 Hz, 1H), 4.89 (s, 2H) ppm; ^13^C-NMR (CD_2_Cl_2_): δ = 166.9, 153.1, 143.0, 141.4, 139.0, 137.0, 135.2, 132.1, 129.7, 129.6, 129.5, 129.2, 129.0, 128.3, 127.8, 127.7, 124.9, 123.7, 123.6, 121.8, 108.9, 43.7 ppm; HRMS (EI): calcd. for C_23_H_18_ClNO [M^+^]: 421.1233, found 421.1231.

*(E)-4-((4-Chlorophenyl)(phenyl)methylene)-2-methyl-1,2-dihydroisoquinolin-3(4H)-one* (**(E)-7c**): Dark orange solid; m.p. = 171.9 °C; *R*_f_ = 0.15 (silica gel, hexanes−EtOAc 3:1); IR (film) 3211, 2923, 2851, 1642, 1399 cm^−1^; ^1^H-NMR (CDCl_3_): δ = 7.36–7.30 (m, 4H), 7.29–7.27 (m, 1H), 7.18 (d, *J* = 7.0 Hz, 1H), 7.15–7.12 (m, 3H), 6.98–6.94 (m, 3H), 6.85 (d, *J* = 7.8 Hz, 1H), 4.49 (s, 2H), 3.03 (s, 3H) ppm; ^13^C-NMR (CDCl_3_): δ = 165.8, 147.4, 141.7, 141.3, 134.8, 134.2, 133.8, 131.9, 130.1, 129.8, 129.6, 128.8, 127.9, 127.8, 127.27, 127.26, 125.3, 53.0, 35.3 ppm; HRMS (EI): calcd. for C_23_H_18_ClNO [M^+^]: 359.1077, found 359.1074.

*(Z)-4-((4-Chlorophenyl)(phenyl)methylene)-2-methyl-1,2-dihydroisoquinolin-3(4H)-one* (**(*Z*)-7c**): Gold solid; m.p. = 174.5 °C; *R*_f_ = 0.26 (silica gel, hexanes−EtOAc 3:1); IR (film) 3055, 2923, 2851, 1656, 1396 cm^−1^; ^1^H-NMR (CDCl_3_): δ = 7.33–7.35 (m, 4H), 7.18–7.15 (m, 4H), 7.11 (t, *J* = 7.1 Hz, 1H), 7.01–6.99 (m, 2H), 6.89 (t, *J* = 7.5 Hz, 1H), 6.83 (d, *J* = 7.8 Hz, 1H), 4.49 (s, 2H), 3.05 (s, 3H) ppm; ^13^C-NMR (CDCl_3_): δ = 165.9, 147.7, 142.4, 140.5, 131.6, 134.8, 134.0, 133.6, 131.6, 130.4, 129.9, 128.6, 128.0, 127.9, 127.2, 127.1, 125.2, 53.0, 35.3 ppm; HRMS (EI): calcd. for C_23_H_18_ClNO [M^+^]: 359.1077, found 359.1076.

*(E)-3-((4-Chlorophenyl)(phenyl)methylene)benzofuran-2(3H)-one* (**(*E*)-7d**): Yellow solid; m.p. = 145.0 °C; *R*_f_ = 0.26 (silica gel, hexanes−EtOAc 20:1); IR (film) 3359, 3188, 3058, 2922, 1783, 1460, 1074, 761 cm^−1^; ^1^H-NMR (CDCl_3_): δ = 7.45–7.43 (m, 3H), 7.40–7.37 (m, 2H), 7.32–7.28 (m, 4H), 7.26–7.24 (m, 1H), 7.08 (d, *J* = 8.0 Hz, 1H), 6.85 (td, *J* = 7.7, 1.0 Hz, 1H), 6.60 (dd, *J* = 7.8, 0.8 Hz, 1H) ppm; ^13^C-NMR (CDCl_3_): δ = 166.5, 156.8, 153.7, 138.8, 138.6, 136.3, 131.9, 131.1, 130.5, 130.4, 130.2, 129.6, 129.5, 129.3, 128.5, 128.3, 124.2, 123.4, 122.9, 119.5, 111.0 ppm; HRMS (EI): calcd. for C_21_H_13_ClO_2_ [M^+^]: 332.0604, found 332.0603.

*(Z)-3-((4-Chlorophenyl)(phenyl)methylene)benzofuran-2(3H)-one* (**(*Z*)-7d**): Yellow solid; m.p. = 106.4 °C; *R*_f_ = 0.31 (silica gel, hexanes−EtOAc 20:1); IR (film) 3057, 2921, 2850, 1784, 1460, 1064, 750 cm^−1^; ^1^H-NMR (CDCl_3_): δ = 7.51–7.49 (m, 1H), 7.48–7.45 (m, 2H), 7.36–7.34 (m, 2H), 7.32–7.30 (m, 2H), 7.29–7.27 (m, 2H), 7.22 (td, *J* = 7.8, 1.3 Hz, 1H), 7.07 (d, *J* = 7.7 Hz, 1H), 6.80 (td, *J* = 7.7, 1.1 Hz, 1H), 6.49 (dd, *J* = 7.8, 0.8 Hz, 1H) ppm; ^13^C-NMR (CDCl_3_): δ = 166.7, 156.8, 153.7, 140.1, 137.2, 136.4, 131.9, 130.3, 130.1, 129.5, 129.3, 128.5, 124.3, 123.4, 123.1, 119.6, 110.9 ppm; HRMS (EI): calcd. for C_21_H_13_ClO_2_ [M^+^]: 332.0604, found 332.0605.

## 4. Conclusions

In conclusion, consecutive one-pot conditions for the synthesis of 3-(diarylmethylene)oxindoles were successfully converted to more efficient domino MCRs through investigation of ligands, additives, and heating sources (thermal/microwave). Microwave irradiation facilitated domino MCRs and the addition of silver salt proved crucial for high yield and stereoselectivity. A wide range of substrates was tested to demonstrate that the domino MCRs are applicable to the synthesis of 3-(diarylmethylene)oxindoles bearing various substituents at the aryl or heteroaryl groups via three palladium-catalyzed reactions, namely Sonogashira, Heck and Suzuki-Miyaura reactions.

## Figures and Tables

**Table 1:**
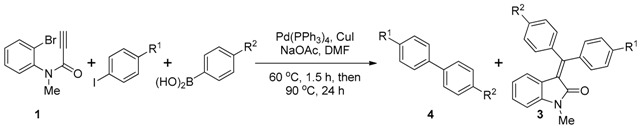
Preliminary results of thermal domino MCRs ^1^.

Entry	R^1^	R^2^	4	Yield (%) ^2^	3	Yield (%) ^2^	*E/Z* Ratio ^3^	Consecutive One-Pot ^4,5^	
								Yield (%) ^2^	*E/Z* Ratio ^3^
1	H	H	**4a**	–	**3a**	68	–	81	–
2	MeO	MeO	**4b**	–	**3b**	50	–	85	–
3	Cl	Cl	**4c**	–	**3c**	86	–	80	–
4 ^5^	NO_2_	NO_2_	**4d**	15	**3d**	66	–	67	–
5	H	MeO	**4e**	14	**3e**	66	1:2	52	1:1
6	H	Cl	**4f**	12	**3f**	78	2:1	57	1:1
7 ^5^	H	NO_2_	**4g**	5	**3g**	75	1:1.1	52	1:1
8	MeO	H	**4e**	–	**3e**	59	1.5:1	70	1.6:1
9	Cl	H	**4f**	–	**3f**	84	1:2.4	77	1:3.4
10 ^6^	NO_2_	H	**4g**	10	**3g**	80	1:13	73	1:10

^1^ Reaction conditions: **1** (1.0 eq), aryl iodide (1.1 eq), arylboronic acid (1.2 eq), Pd(PPh_3_)_4_ (10 mol %), CuI (5 mol %), NaOAc (3 eq), DMF (0.05 M), 60 °C, 1.5 h, then 90 °C, 24 h; ^2^ Isolated yield or combined yield of isolated *E*- and *Z*-isomers; ^3^ Ratio between isolated *E*- and *Z*-isomers; ^4^ Reaction conditions: **1** (1.0 eq), aryl iodide (1.1 eq), Pd(PPh_3_)_4_ (10 mol %), CuI (5 mol %), NaOAc (3 eq), DMF (0.05 M), 60 °C, 1.5 h, then arylboronic acid (1.2 eq), 90 °C, 24 h; ^5^ Reported data in reference [8]; ^6^ Reaction temperature was raised from 60 °C to 110 °C.

**Table 2:**
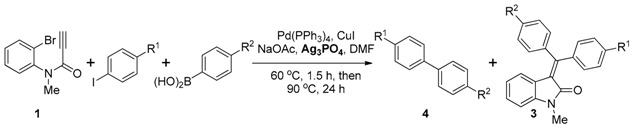
Effect of silver salt on thermal domino MCRs ^1^.

Entry	R^1^	R^2^	4	Yield (%) ^2^	3	Yield (%) ^2^	*E/Z* Ratio ^3^	Consecutive One-Pot (Ag_3_PO_4_) ^4^		Consecutive One-Pot (AgOTf) ^4,5^	
								Yield (%) ^2^	*E/Z* Ratio	Yield (%) ^2^	*E/Z* Ratio
1	H	MeO	**4e**	45	**3e**	42	3.5:1	63	10:1	70	2.5:1
2	H	Cl	**4f**	20	**3f**	39	4.5:1	86	9:1	45	7:1
3 ^6^	H	NO_2_	**4g**	25	**3g**	50	4:1	66	3:1	56	5.2:1

^1^ Reaction conditions: **1** (1.0 eq), aryl iodide (1.1 eq), arylboronic acid (1.2 eq), Pd(PPh_3_)_4_ (10 mol %), CuI (5 mol %), NaOAc (3 eq), Ag_3_PO_4_ (1.1 eq), DMF (0.05 M), 60 °C, 1.5 h, then 90 °C, 24 h; ^2^ Isolated yield or combined yield of isolated *E*- and *Z*-isomers; ^3^ Ratio between isolated *E*- and *Z*-isomers; ^4^ Reaction conditions: **1** (1.0 eq), aryl iodide (1.1 eq), Pd(PPh_3_)_4_ (10 mol %), CuI (5 mol %), NaOAc (3 eq), DMF (0.05 M), 60 °C, 1.5 h, then arylboronic acid (1.2 eq), silver salt (1.1 eq), 90 °C, 24 h; ^5^ Reported data in reference [8]; ^6^ Reaction temperature was raised from 60 °C to 110 °C.

**Table 3:**
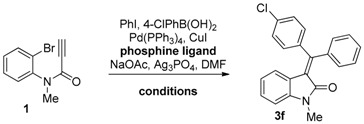
Phosphine ligand effect and further optimization ^1^.

Entry	Phosphine Ligand	Conditions	Yield (%) ^2^	*E*/*Z* Ratio ^3^
1	dppp	60 °C, 1.5 h then 90 °C, 24 h	0	-
2	dppb	60 °C, 1.5 h then 90 °C, 24 h	0	-
3	dppf	60 °C, 1.5 h then 90 °C, 24 h	0	-
4	P(*o*-tol)_3_	60 °C, 1.5 h then 90 °C, 24 h	13	3.5:1
5	PPh_3_	60 °C, 1.5 h then 90 °C, 24 h	50	5:1
6	PPh_3_	90 °C, 24 h	67	7.5:1
7	PPh_3_	150 °C, 1.5 h	45	3.5:1
8	PPh_3_	150 °C, 3 h	61	3.5:1

^1^ Reaction conditions: **1** (1.0 eq), phenyl iodide (1.1 eq), 4-chlorophenylboronic acid (1.2 eq), Pd(PPh_3_)_4_ (10 mol %), CuI (5 mol %), NaOAc (3 eq), Ag_3_PO_4_ (1.1 eq), phosphine ligand (30 mol %), DMF (0.05 M), conditions; ^2^ Combined yield of isolated *E*- and *Z*-isomers; ^3^ Ratio between isolated *E*- and *Z*-isomers. dppp: 1,3-bis(diphenylphosphino)propane; dppb: 1,4-bis(diphenylphosphino)-butane; dppf: 1,1′-bis(diphenylphosphino)ferrocene.

**Table 4:**
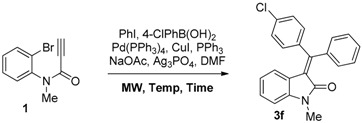
Optimization of microwave reactions ^1^.

Entry	Temp (°C)	Time (min)	Yield (%) ^2^	*E/Z* Ratio ^3^
1	100	10	46	12:1
2	100	30	67	18:1
3	100	60	80	9:1
4	110	10	54	7:1
5	130	10	92	13:1
6	150	10	99	13:1
7	160	5	98	7.5:1
8 ^4^	150	10	92	8:1
9 ^5^	150	10	36	1:1.2
10 ^6^	150	10	35	1:1.5
11 ^7^	150	10	79	10:1

^1^ Reaction conditions: **1** (1.0 eq), phenyl iodide (1.1 eq), 4-chlorophenylboronic acid (1.2 eq), Pd(PPh_3_)_4_ (10 mol %), CuI (5 mol %), NaOAc (3 eq), Ag_3_PO_4_ (1.1 eq), PPh_3_ (30 mol %), DMF (0.05 M), microwave irradiation, temperature and time on table; ^2^ Combined yield of isolated *E*- and *Z*-isomers; ^3^ Ratio between isolated *E*- and *Z*-isomers; ^4^ Reaction without PPh_3_; ^5^ Reaction without Ag_3_PO_4_; ^6^ Reaction without PPh_3_ and Ag_3_PO_4_; ^7^ Two equivalents of Ag_3_PO_4_ were used without NaOAc.

**Table 5:**
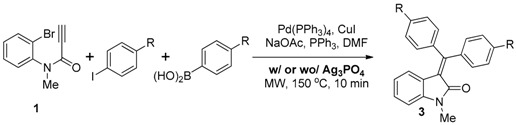
Substrate scope for symmetric products and the effect of silver salt ^1^.

Entry	R	3	With Ag_3_PO_4_^2^	Without Ag_3_PO_4_
			Yield (%) ^3^	Yield (%) ^3^
1	H	**3a**	75	27
2	MeO	**3b**	75	55
3	Cl	**3c**	72	15
4	NO_2_	**3d**	90	9

^1^ Reaction conditions: **1** (1.0 eq), aryl iodide (1.1 eq), arylboronic acid (1.2 eq), Pd(PPh_3_)_4_ (10 mol %), CuI (5 mol %), NaOAc (3 eq), PPh_3_ (30 mol %), with or without Ag_3_PO_4_ (1.1 eq), DMF (0.05 M), microwave irradiation, 150 °C, 10 min; ^2^ Reported data in reference [9]; ^3^ Isolated yield.

**Table 6:**
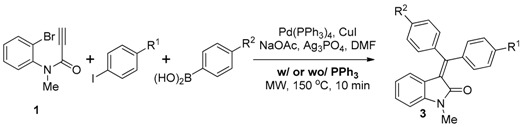
Basic substrate scope for unsymmetric products and the ligand effect ^1^.

Entry	R^1^	R^2^	3	With PPh_3_^2^		Without PPh_3_	
				Yield (%) ^3^	*E/Z* Ratio ^4^	Yield (%) ^3^	*E/Z* Ratio ^4^
1	H	MeO	**3e**	85	8:1	88	8:1
2	H	Cl	**3f**	99	13:1	92	8:1
3	H	NO_2_	**3g**	89	9.5:1	86	9:1
4	MeO	H	**3e**	80	1.2:1	68	1.6:1
5	Cl	H	**3f**	85	1:15	81	1:18
6	NO_2_	H	**3g**	88	1:>20	84	1:>20

^1^ Reaction conditions: **1** (1.0 eq), aryl iodide (1.1 eq), arylboronic acid (1.2 eq), Pd(PPh_3_)_4_ (10 mol %), CuI (5 mol %), NaOAc (3 eq), with or without PPh_3_ (30 mol %), Ag_3_PO_4_ (1.1 eq), DMF (0.05 M), microwave irradiation, 150 °C, 10 min; ^2^ Reported data in reference [9]; ^3^ Isolated yield or combined yield of isolated *E*- and *Z*-isomers; ^4^ Ratio between isolated *E*- and *Z*-isomers.

**Table 7:**
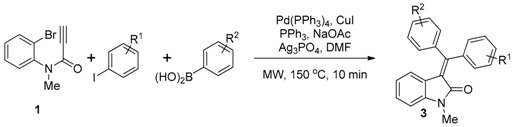
Expanded substrate scope for unsymmetric products ^1^.

Entry	R^1^	R^2^	3	Yield (%) ^2^	*E/Z* Ratio ^3^
1	H	4-AcO	**3h**	45	10:1
2	4-AcO	H	**3h**	82	1:8
3	H	3-MeO	**3i**	98	9:1
4	H	3-Cl	**3j**	72	10:1
5 ^4^	H	3-Cl	**3j**	94	7.5:1
6	H	3-NO_2_	**3k**	93	12:1
7	3-MeO	H	**3i**	85	1:8
8	3-Cl	H	**3j**	81	1:13
9	3-NO_2_	H	**3k**	65	1:>20
10 ^4^	3-NO_2_	H	**3k**	94	1:>20
11	H	2-MeO	**3l**	81	6.5:1
12	H	2-Cl	**3m**	84	8:1
13	H	2-NO_2_	**3n**	0	-
14	2-MeO	H	**3l**	57	1.2:1
15	2-Cl	H	**3m**	85	1:14
16	2-NO_2_	H	**3n**	17	1:>20

^1^ Reaction conditions: **1** (1.0 eq), aryl iodide (1.1 eq), arylboronic acid (1.2 eq), Pd(PPh_3_)_4_ (10 mol %), CuI (5 mol %), PPh_3_ (30 mol %), NaOAc (3 eq), Ag_3_PO_4_ (1.1 eq), DMF (0.05 M), microwave irradiation, 150 °C, 10 min; ^2^ Isolated yield or combined yield of isolated *E*- and *Z*-isomers; ^3^ Ratio between isolated *E*- and *Z*-isomers; ^4^ Two equivalents of arylboronic acid were used.

**Table 8:**
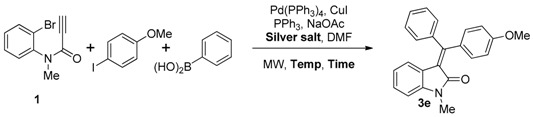
Further optimization for the reaction with 4-methoxyphenyl iodide ^1^.

Entry	Silver Salt (eq)	Temp (°C)	Time (min)	Yield (%) ^2^	*E*/*Z* Ratio ^3^
1	Ag_3_PO_4_ (1.1)	150	10	80	1.2:1
2	Ag_3_PO_4_ (1.5)	150	10	85	1:1
3	Ag_3_PO_4_ (2.0)	150	10	61	1:1.3
4 ^4^	Ag_3_PO_4_ (4.0)	150	10	41	1:1.5
5	Ag_2_CO_3_ (1.1)	150	10	56	1.3:1
6	AgBF_4_ (1.1)	150	10	12	3:1
7	AgOTf (1.1)	150	10	25	2.3:1
8	AgOAc (1.1)	150	10	0	-
9	Ag_3_PO_4_ (1.1)	130	10	70	1:1.5
10	Ag_3_PO_4_ (1.1)	100	60	70	1:4
11	Ag_3_PO_4_ (1.1)	100	180	79	1:3

^1^ Reaction conditions: **1** (1.0 eq), 4-methoxylphenyl iodide (1.1 eq), phenylboronic acid (1.2 eq), Pd(PPh_3_)_4_ (10 mol %), CuI (5 mol %), PPh_3_ (30 mol %), NaOAc (3 eq), silver salt, DMF (0.05 M), microwave irradiation, indicated temperature and time in table; ^2^ Combined yield of isolated *E*- and *Z*-isomers; ^3^ Ratio between isolated *E*- and *Z*-isomers; ^4^ No base (NaOAc) was used.

**Table 9:**
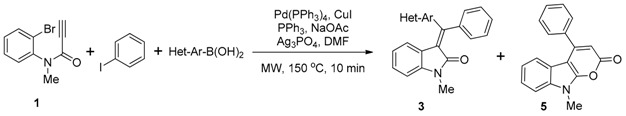
Substrate scope with heteroarylboronic acids ^1^.

Entry	Het-Ar-B(OH)_2_	3	Yield (%) ^2^	*E/Z* Ratio ^3^	5 (%) ^2^
1	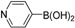	**3o**	77	>20:1	0
2	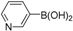	**3p**	86	14:1	0
3	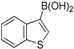	**3q**	52	16:1	25
4 ^4^	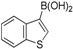	**3q**	83	8.5:1	0
5	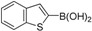	**3r**	56	1.3:1	19
6 ^4^	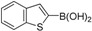	**3r**	97	1.3:1	0
7	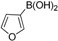	**3s**	68	9:1	10
8 ^4^	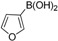	**3s**	97	6:1	0

^1^ Reaction conditions: **1** (1.0 eq), phenyl iodide (1.1 eq), heteroarylboronic acid (1.2 eq), Pd(PPh_3_)_4_ (10 mol %), CuI (5 mol %), PPh_3_ (30 mol %), NaOAc (3 eq), Ag_3_PO_4_ (1.1 eq), DMF (0.05 M), microwave irradiation, 150 °C, 10 min; ^2^ Isolated yield or combined yield of isolated *E*- and *Z*-isomers; ^3^ Ratio between isolated *E*- and *Z*-isomers; ^4^ Two equivalents of heteroarylboronic acid were used.

**Table 10:**
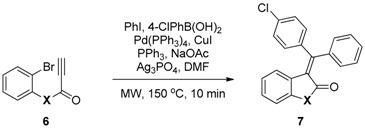
*N*-Substituent effect and application to other heterocycles ^1^.

Entry	6	X	7	Yield (%) ^2^	*E/Z* Ratio ^3^
1	**6a**	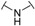	**7a**	85	2.2:1
2	**6b**	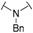	**7b**	98	3.3:1
3	**6c**	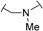	**7c**	35	2.5:1
4 ^4^	**6c**	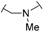	**7c**	45	1.1:1
5	**6d**	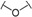	**7d**	35	1.1:1

^1^ Reaction conditions: **6** (1.0 eq), phenyl iodide (1.1 eq), 4-chlorophenylboronic acid (1.2 eq), Pd(PPh_3_)_4_ (10 mol %), CuI (5 mol %), PPh_3_ (30 mol %), NaOAc (3 eq), Ag_3_PO_4_ (1.1 eq), DMF (0.05 M), microwave irradiation, 150 °C, 10 min; ^2^ Combined yield of isolated *E*- and *Z*-isomers; ^3^ Ratio between isolated *E*- and *Z*-isomers; ^4^ Reaction was run at 180 °C.

**Table 11:**
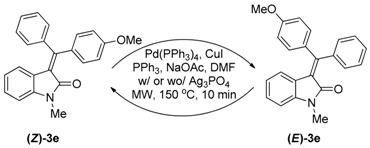
Isomerization studies ^1^.

Entry	Starting Material	Silver Salt	Conversion Rate (%)
1	**(*Z*)-3e**	Ag_3_PO_4_	2
2	**(*Z*)-3e**	-	2
3	**(*E*)-3e**	Ag_3_PO_4_	15
4	**(*E*)-3e**	-	6

^1^ Reaction conditions: **(*Z*)-** or **(*E*)-3e** (1.0 eq), Pd(PPh_3_)_4_ (10 mol %), CuI (5 mol %), PPh_3_ (30 mol %), NaOAc (3 eq), with or without Ag_3_PO_4_ (1.1 eq), DMF (0.05 M), microwave irradiation, 150 °C, 10 min.

## Supplementary Materials

Supplementary materials are available online. copies of NMR spectra of compounds **1** and **3**–**7**.
