# Supplementary material for: Consecutive One-Pot versus Domino Multicomponent Approaches to 3-(Diarylmethylene)oxindoles
Source: Molecules. 2017 Mar 22;22(3):503. doi: 10.3390/molecules22030503 (PMC6155274; doi:10.3390/molecules22030503)

<Supporting Information>

Sequential One-pot versus Tandem Multicomponent Approaches to 3-(Diarylmethylene)oxindoles

Sunhwa Park, Jiyun Lee, Kye Jung Shin, Euichaul Oh and Jae Hong Seo *

N-(2-bromophenyl)-*N*-methylpropiolamide (1)

^{1}H NMR spectrum of 1

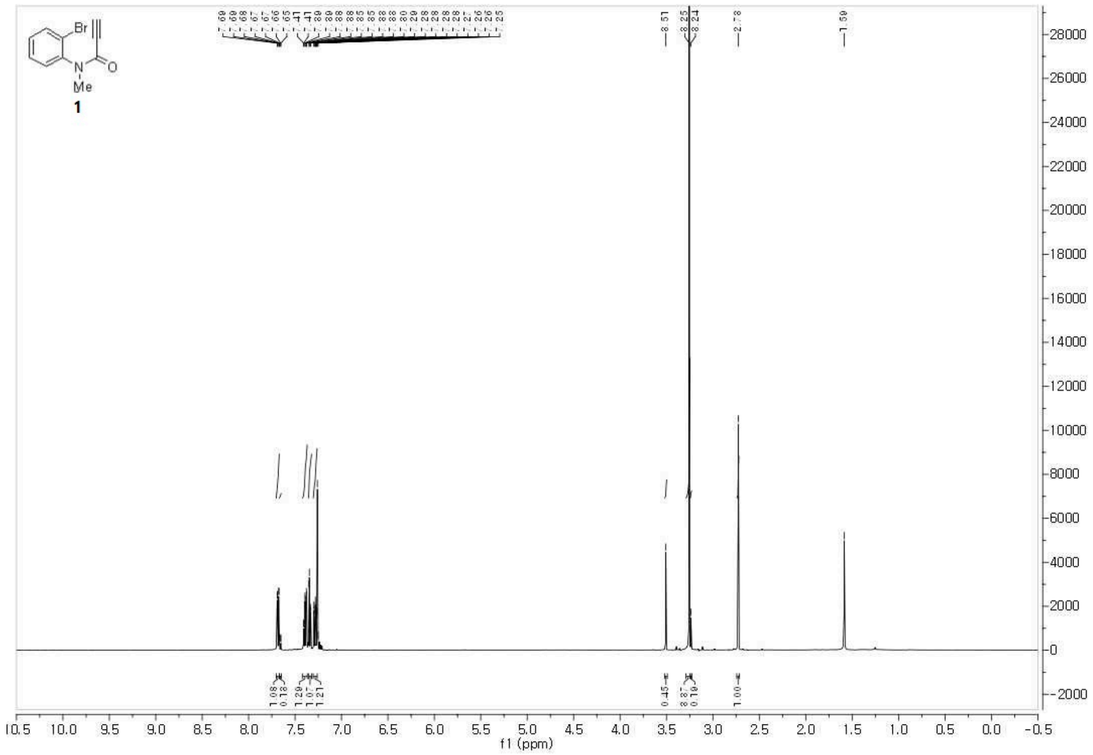

^{13}C NMR spectrum of 1

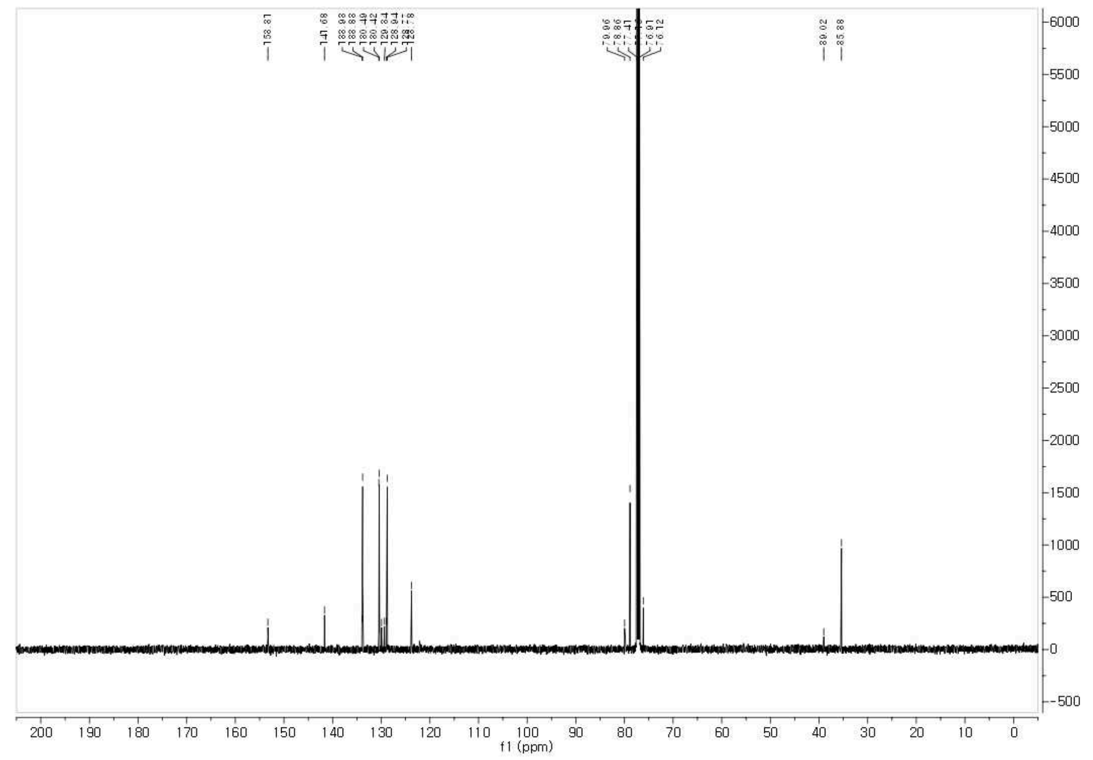

3-(diphenylmethylene)-1-methylindolin-2-one (3a)

^{1}H NMR spectrum of 3a

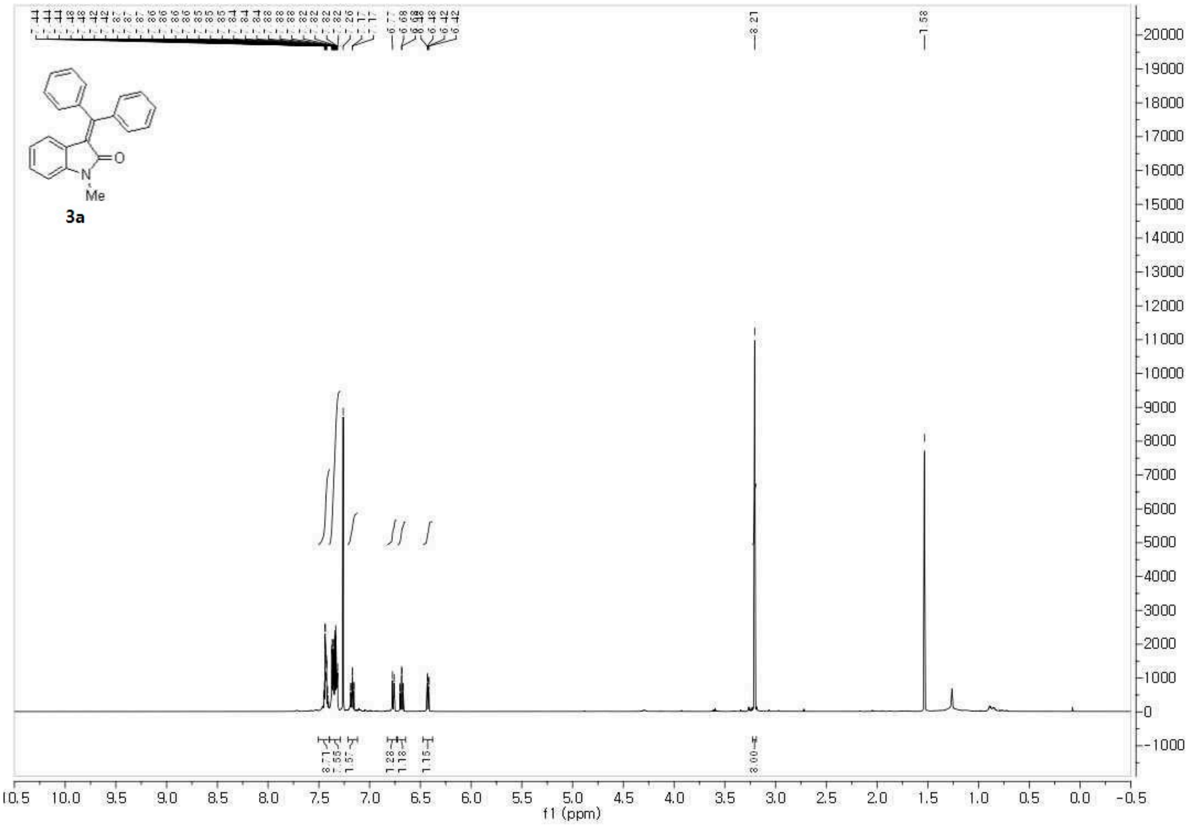

^{13}C NMR spectrum of 3a

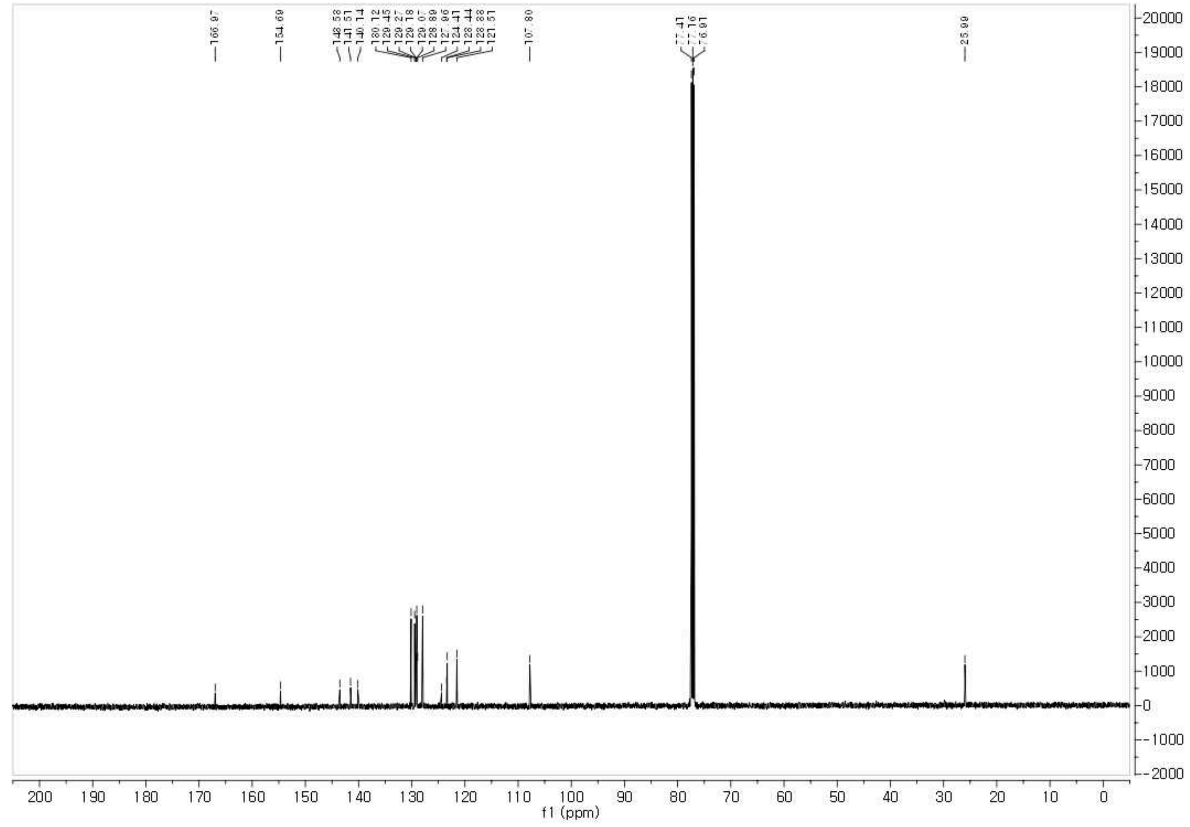

3-(bis(4-methoxyphenyl)methylene)-1-methylindolin-2-one (3b)

^{1}H NMR spectrum of 3b

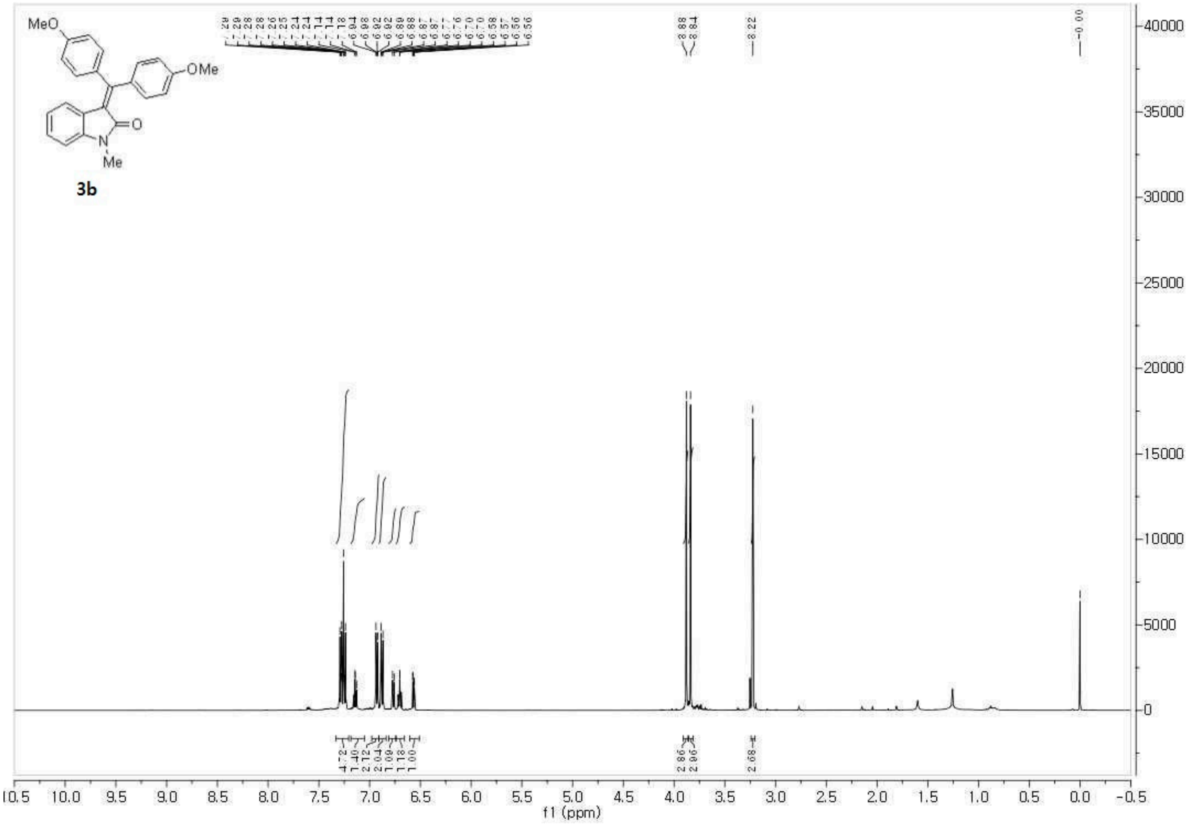

^{13}C NMR spectrum of 3b

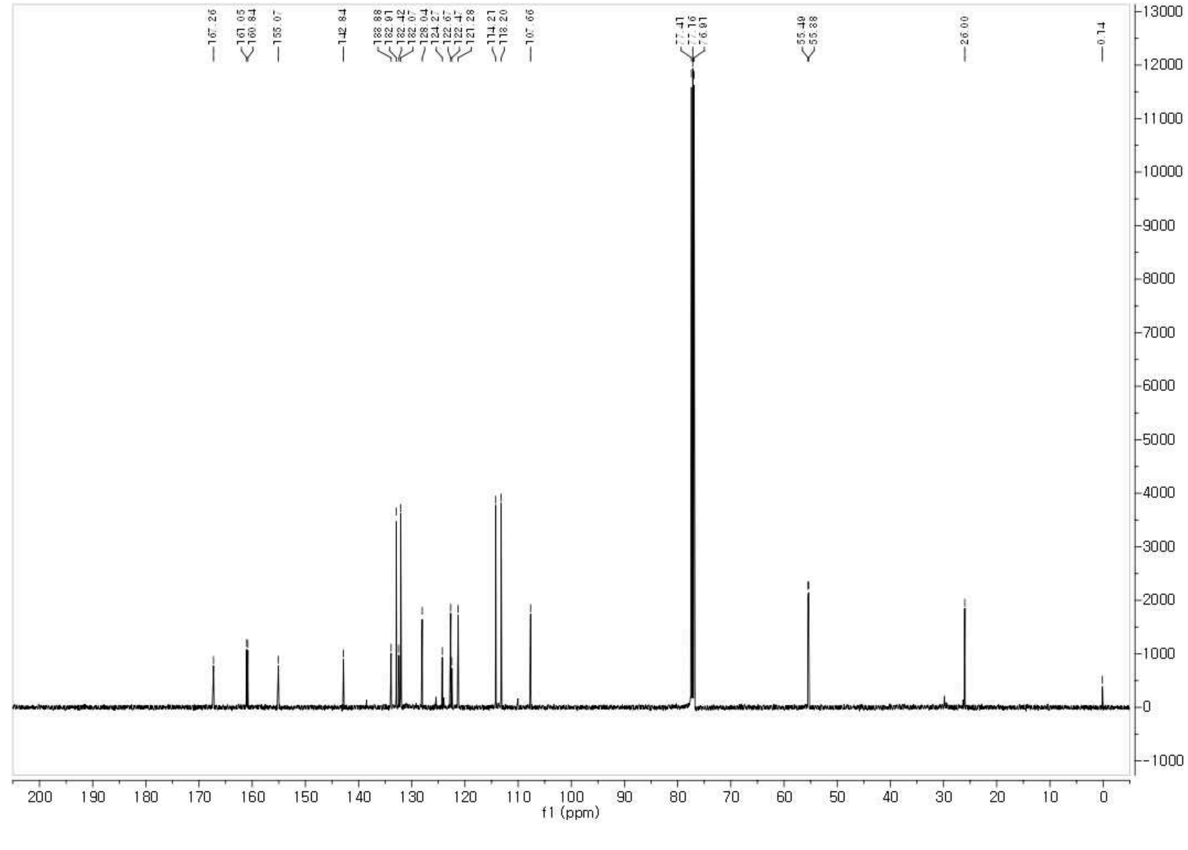

3-(bis(4-chlorophenyl)methylene)-1-methylindolin-2-one (3c)

^{1}H NMR spectrum of 3c

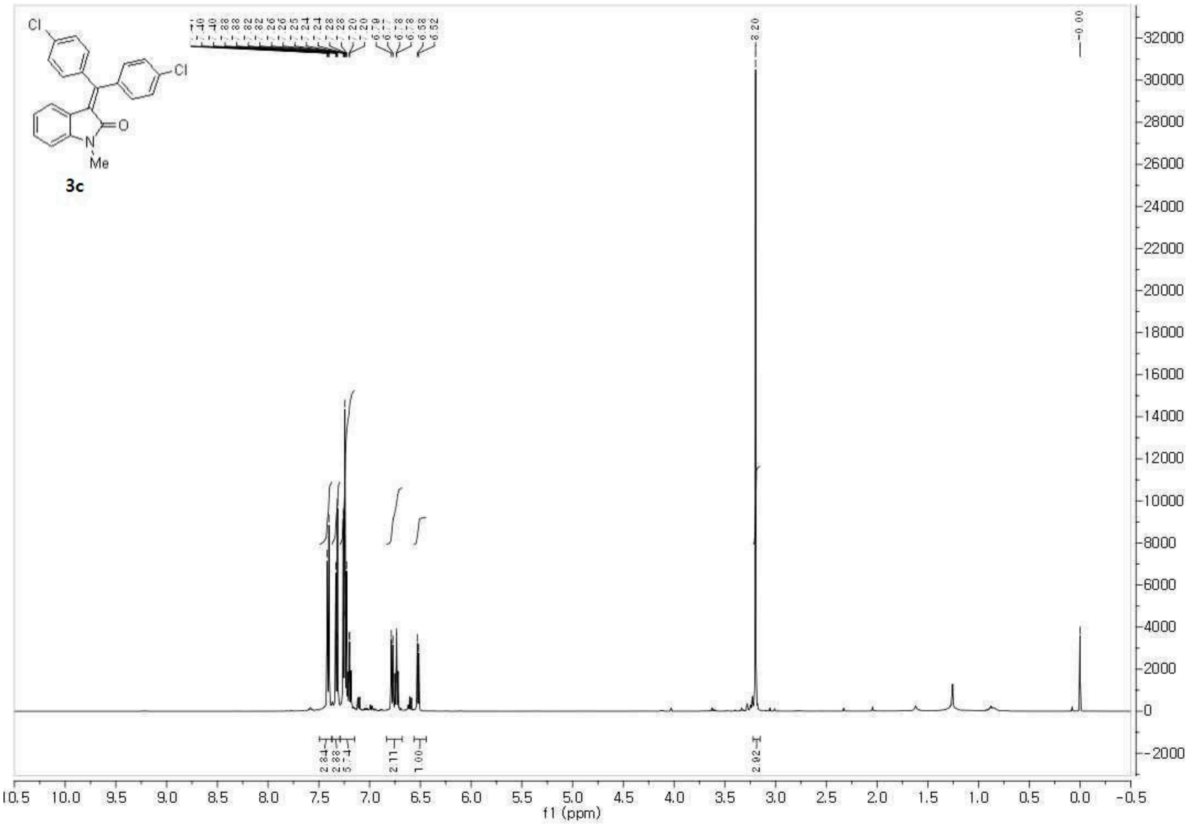

^{13}C NMR spectrum of 3c

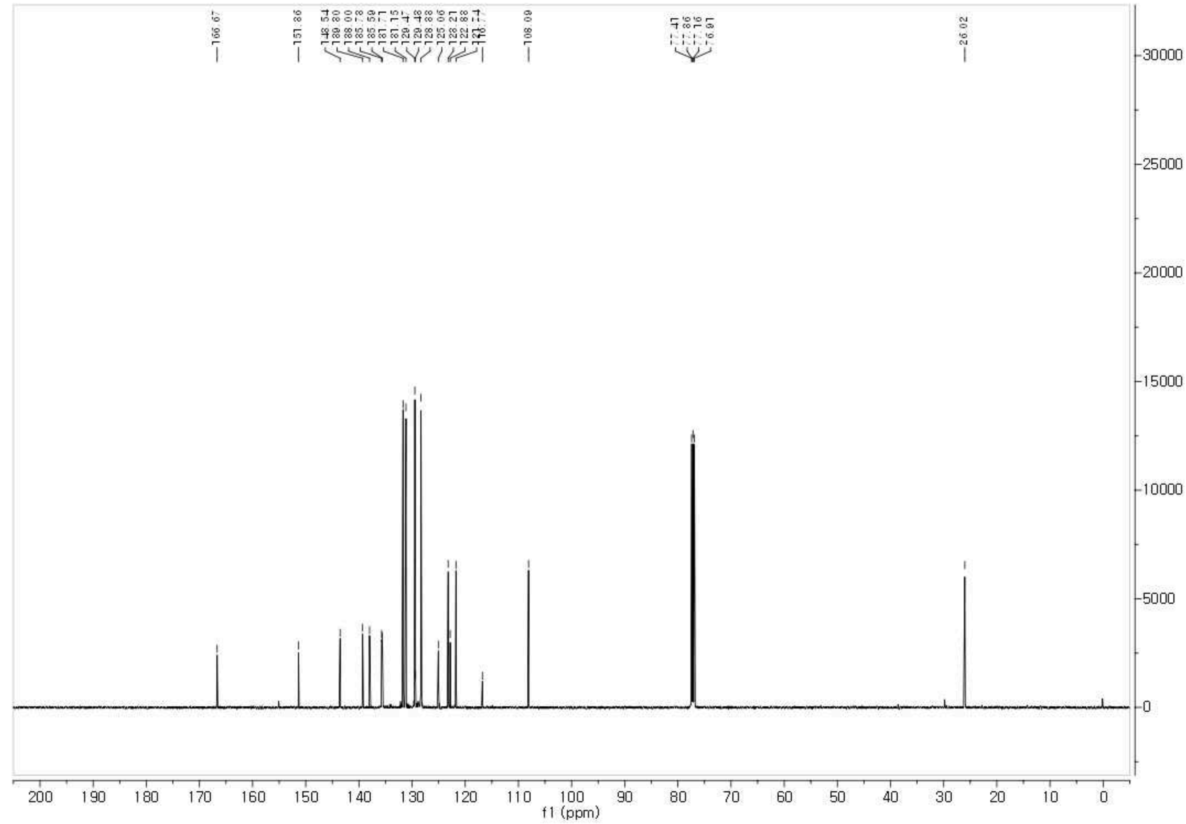

3-(bis(4-nitrophenyl)methylene)-1-methylindolin-2-one (3d)

^{1}H NMR spectrum of 3d

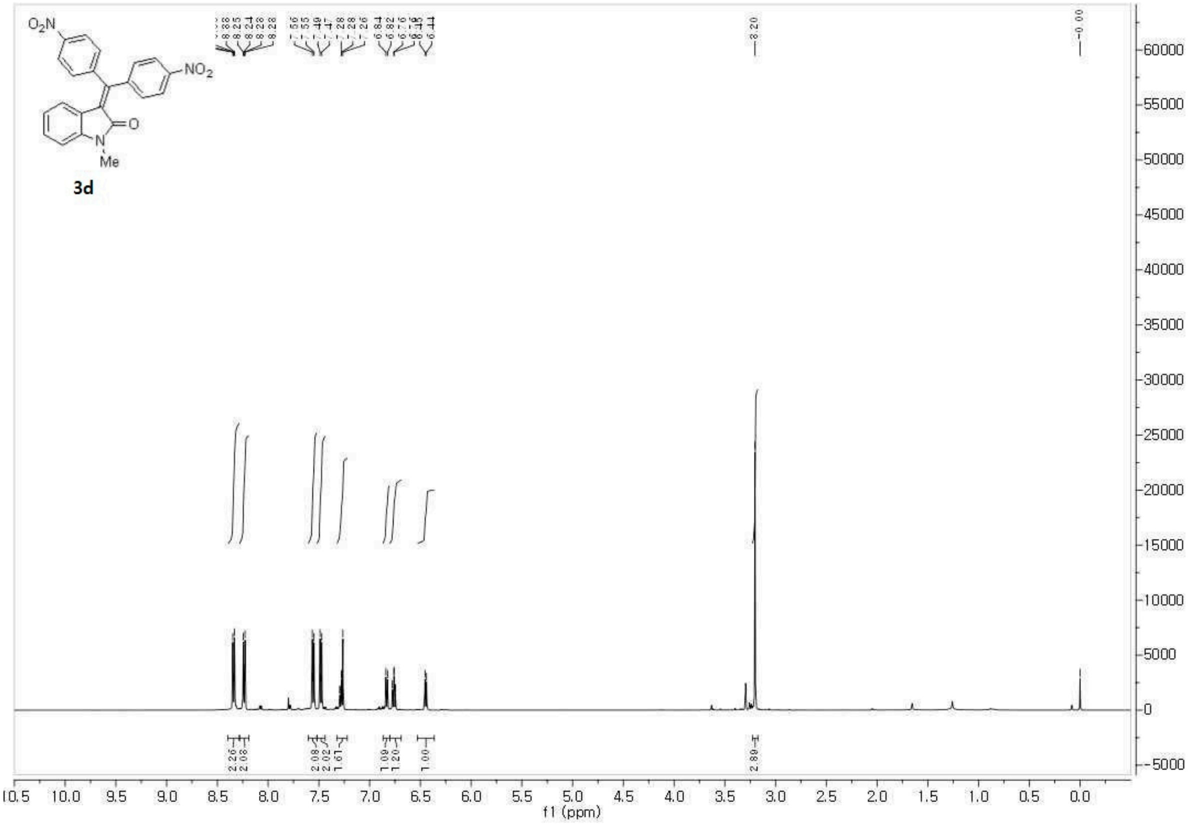

^{13}C NMR spectrum of 3d

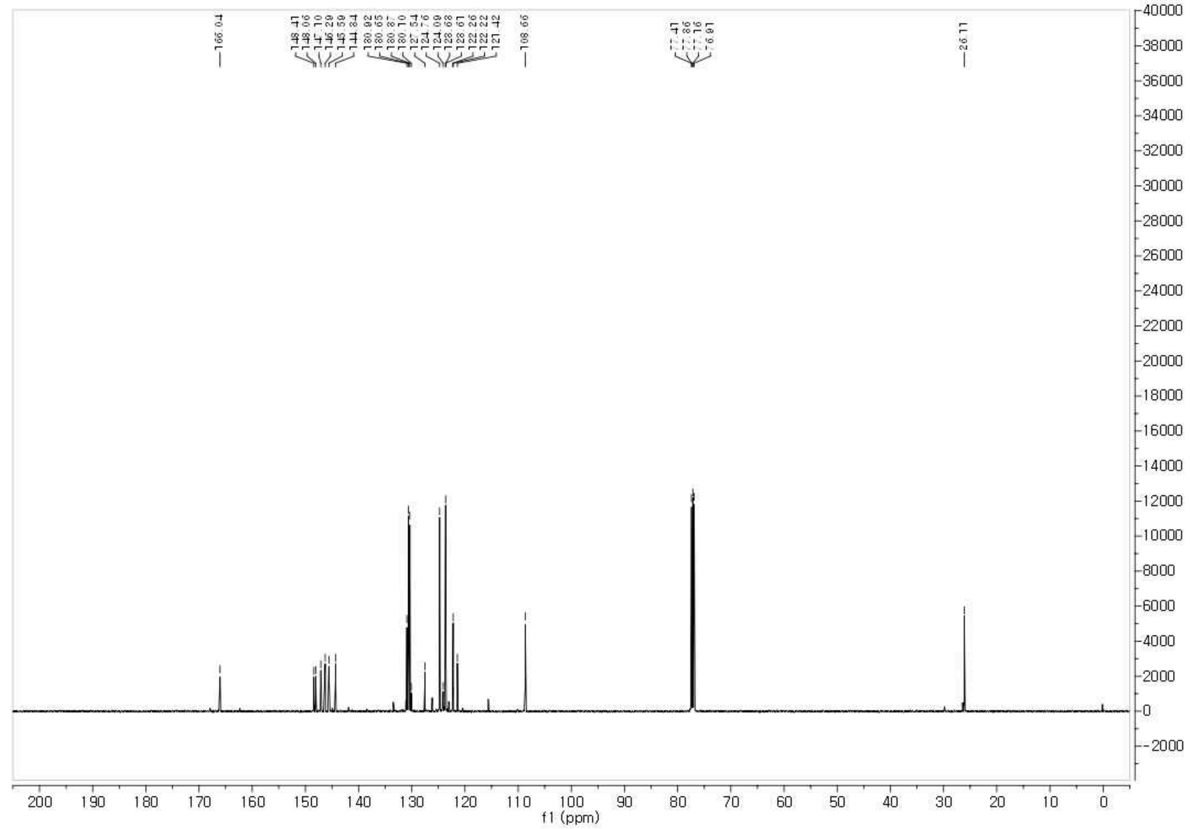

(E)-3-((4-methoxyphenyl)(phenyl)methylene)-1-methylindolin-2-one ((E)-3e)

^{1}H NMR spectrum of (E)-3e

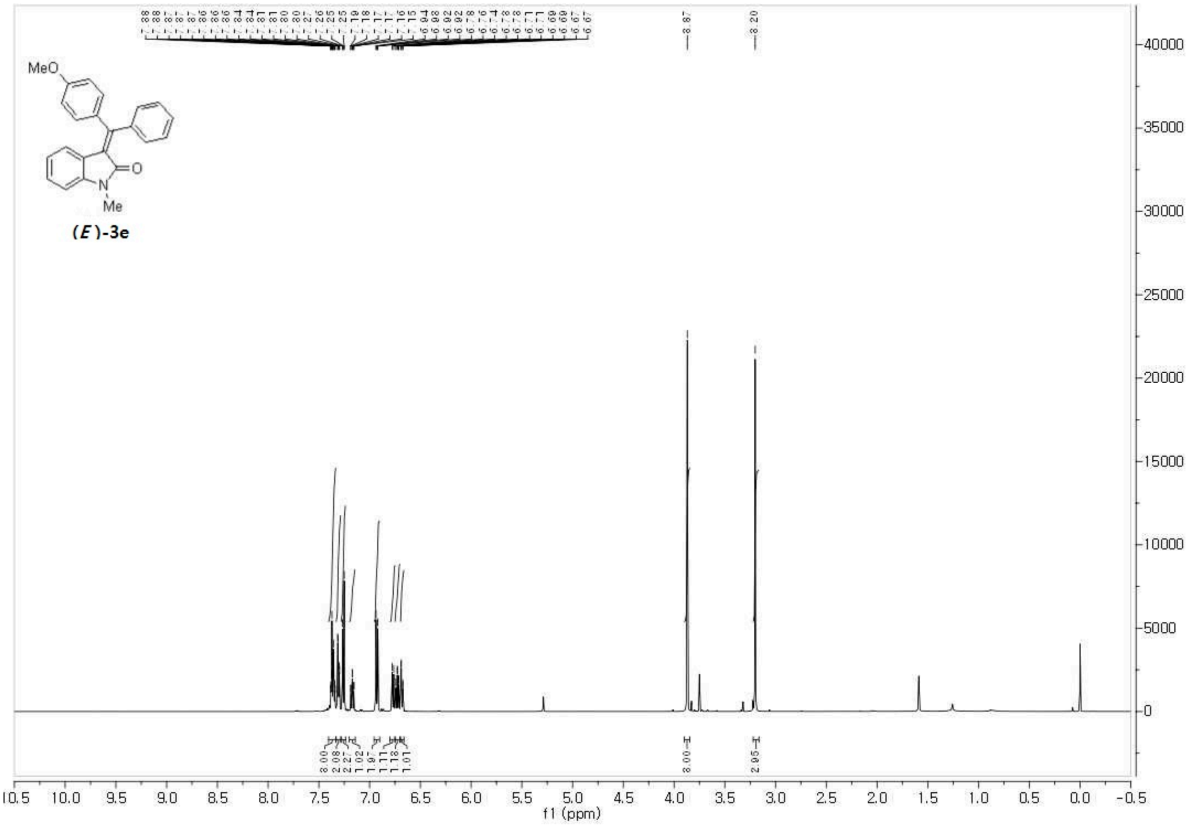

^{13}C NMR spectrum of (E)-3e

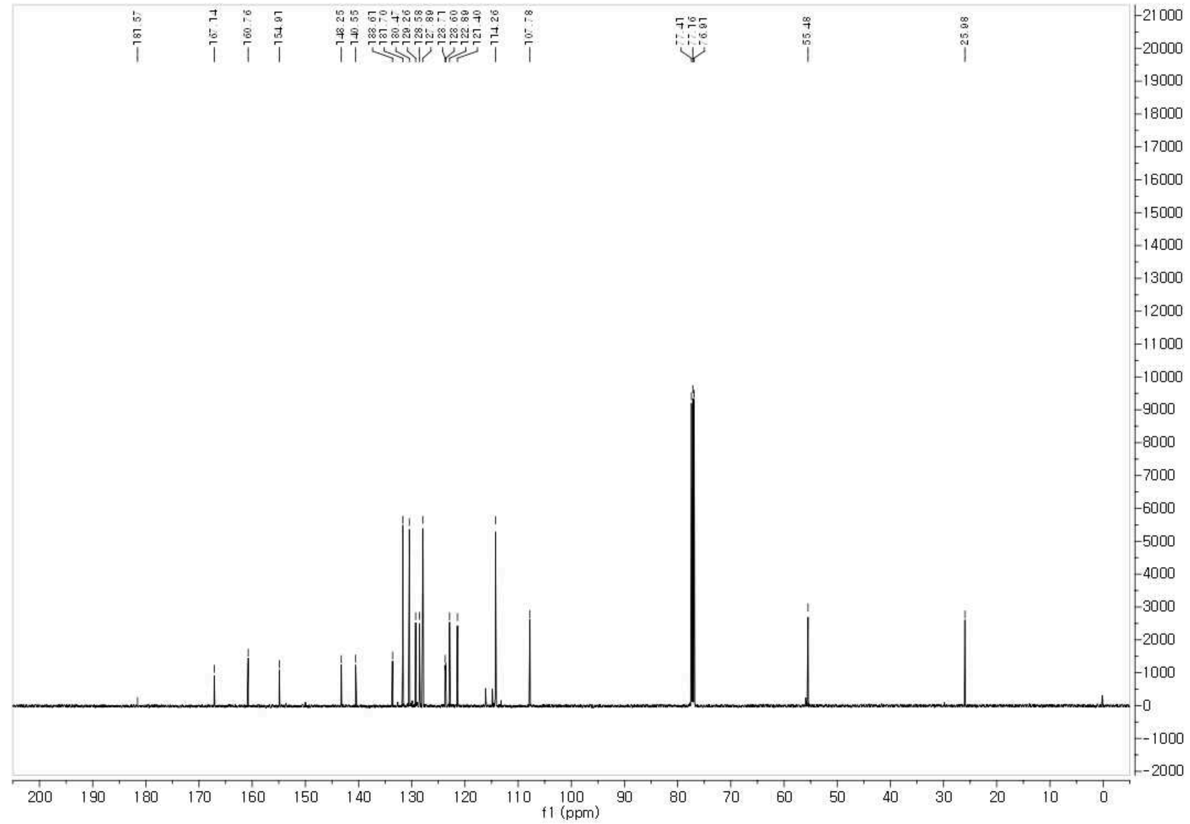

(*Z*)-3-((4-methoxyphenyl)(phenyl)methylene)-1-methylindolin-2-one ((*Z*)-3e)

^{1}H NMR spectrum of (*Z*)-3e

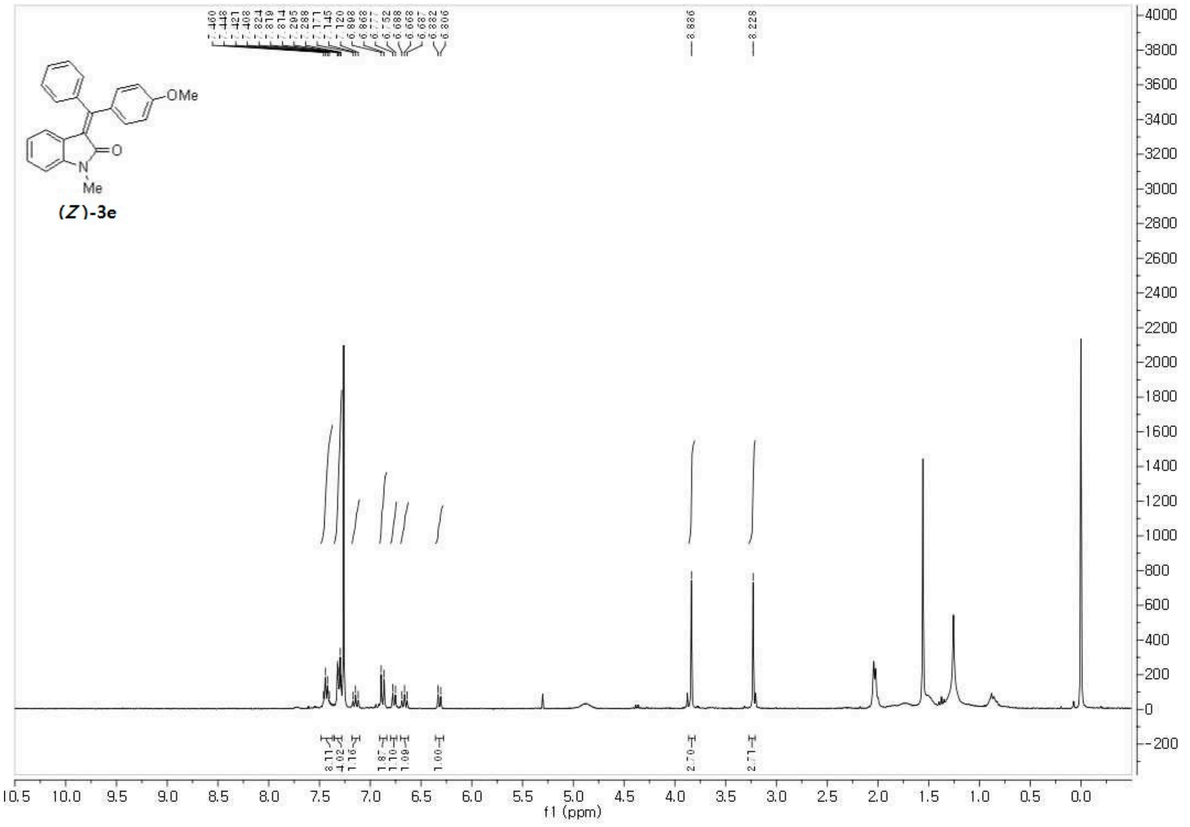

^{13}C NMR spectrum of (*Z*)-3e

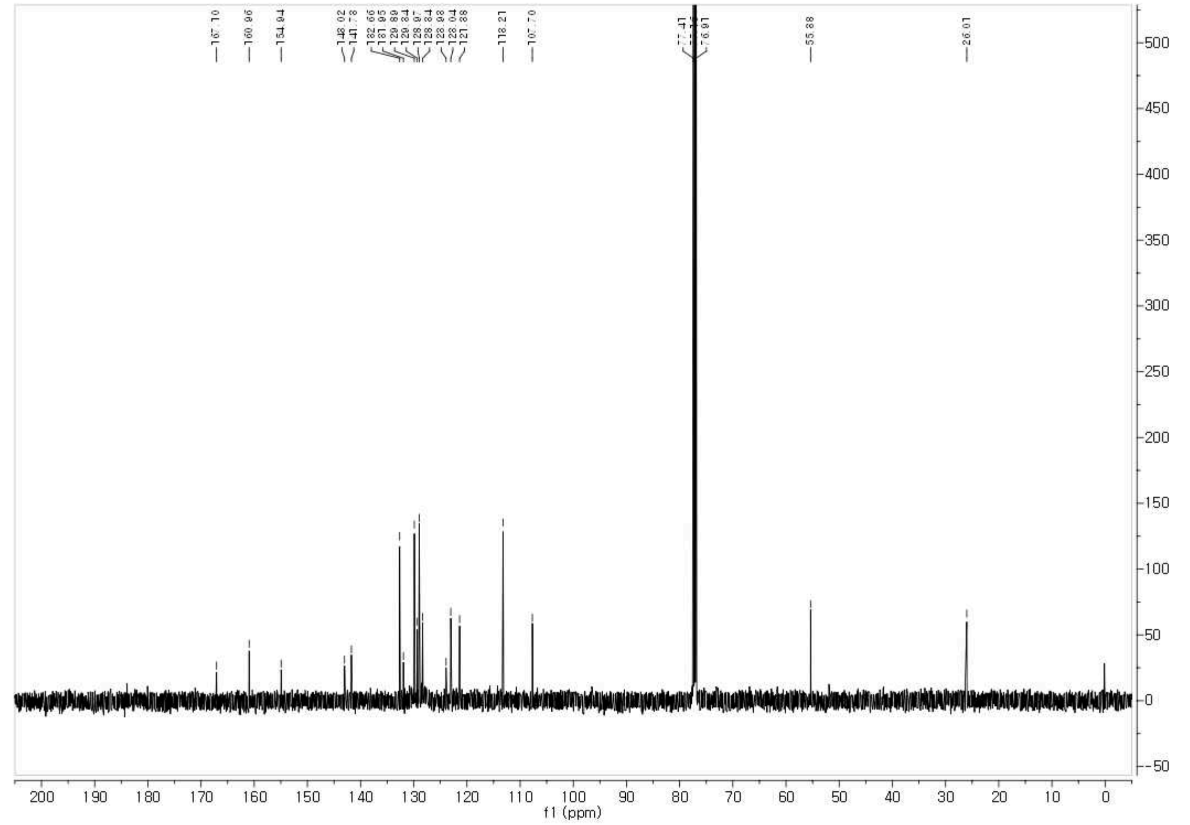

(*E*)-3-((4-chlorophenyl)(phenyl)methylene)-1-methylindolin-2-one ((*E*)-3f)

^{1}H NMR spectrum of (*E*)-3f

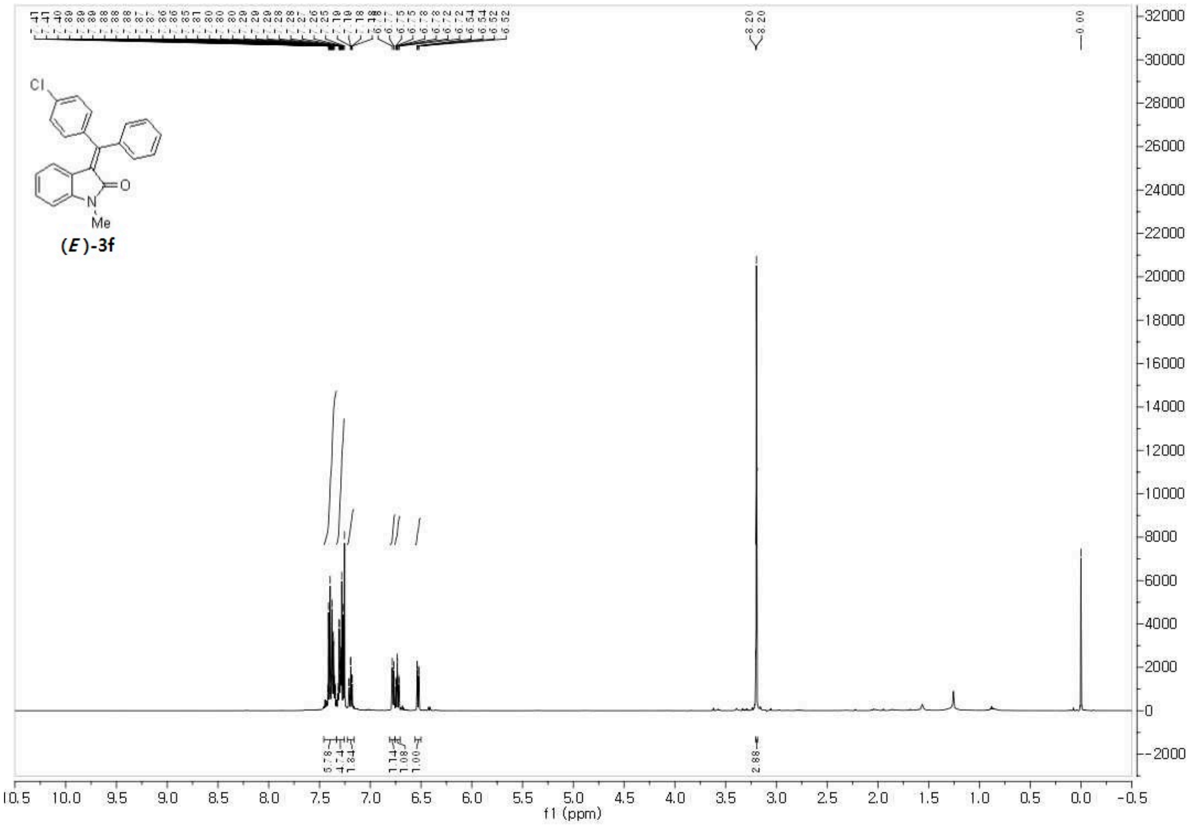

^{13}C NMR spectrum of (*E*)-3f

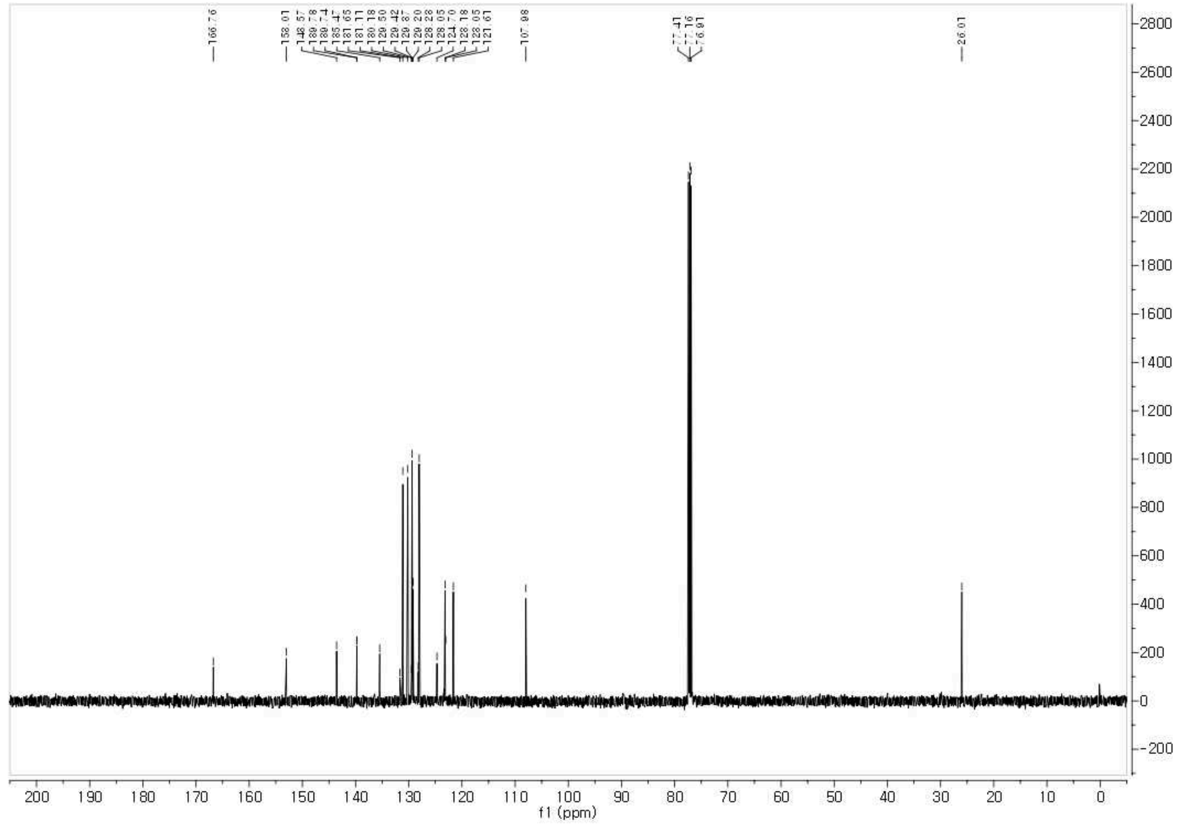

(*Z*)-3-((4-chlorophenyl)(phenyl)methylene)-1-methylindolin-2-one ((*Z*)-3f)

^{1}H NMR spectrum of (*Z*)-3f

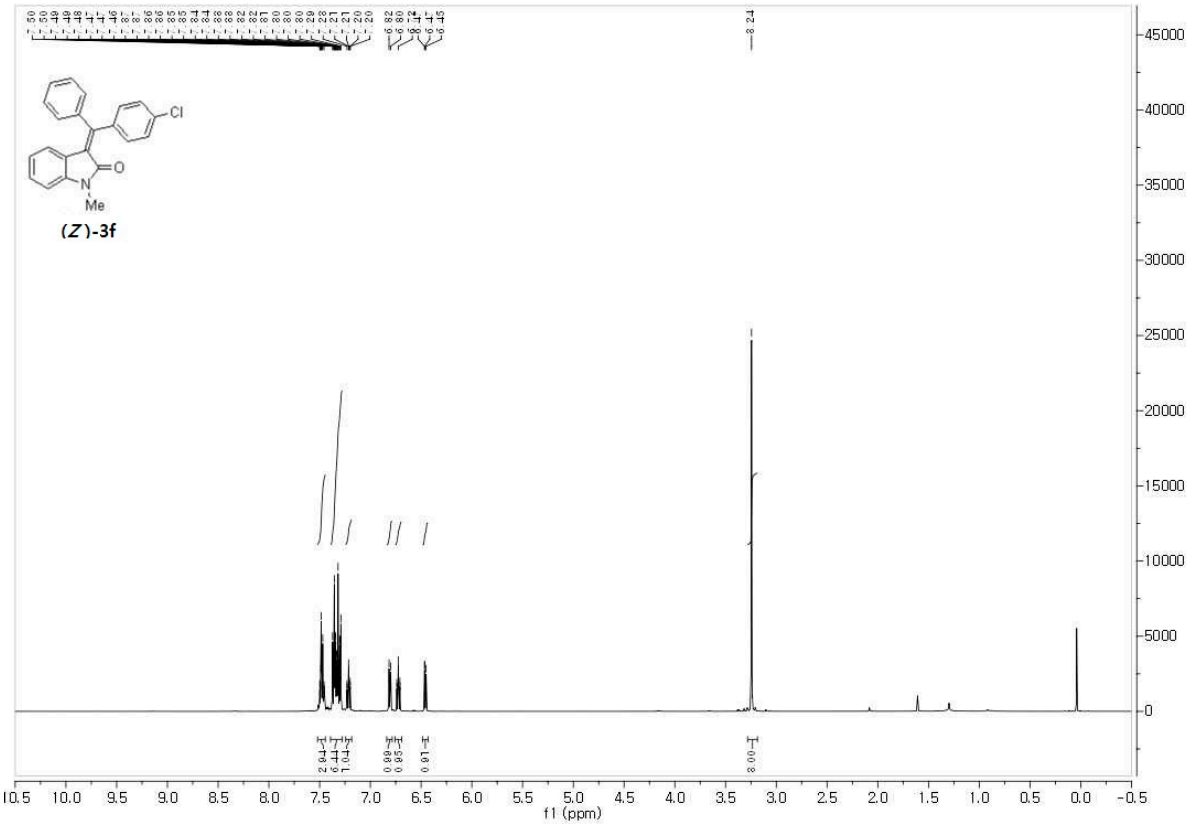

^{13}C NMR spectrum of (*Z*)-3f

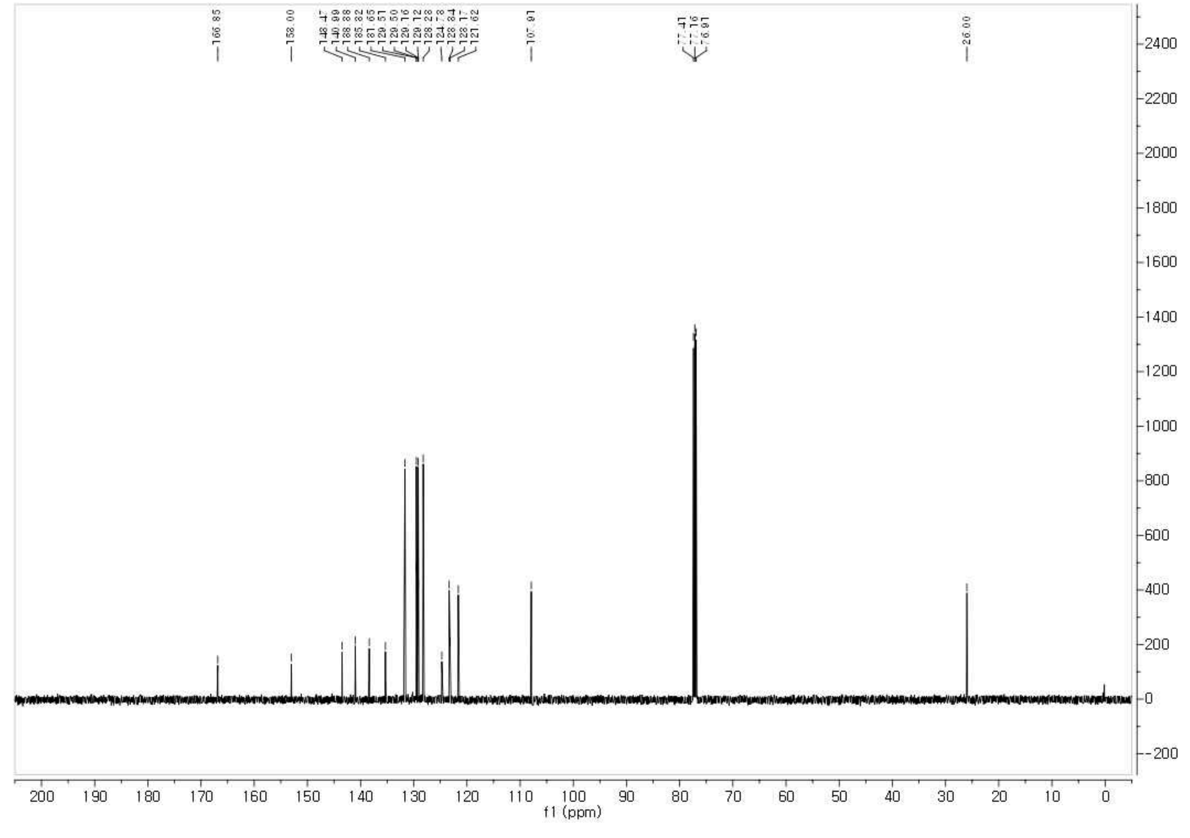

(*E*)-1-methyl-3-((4-nitrophenyl)(phenyl)methylene)indolin-2-one ((*E*)-3g)

^{1}H NMR spectrum of (*E*)-3g

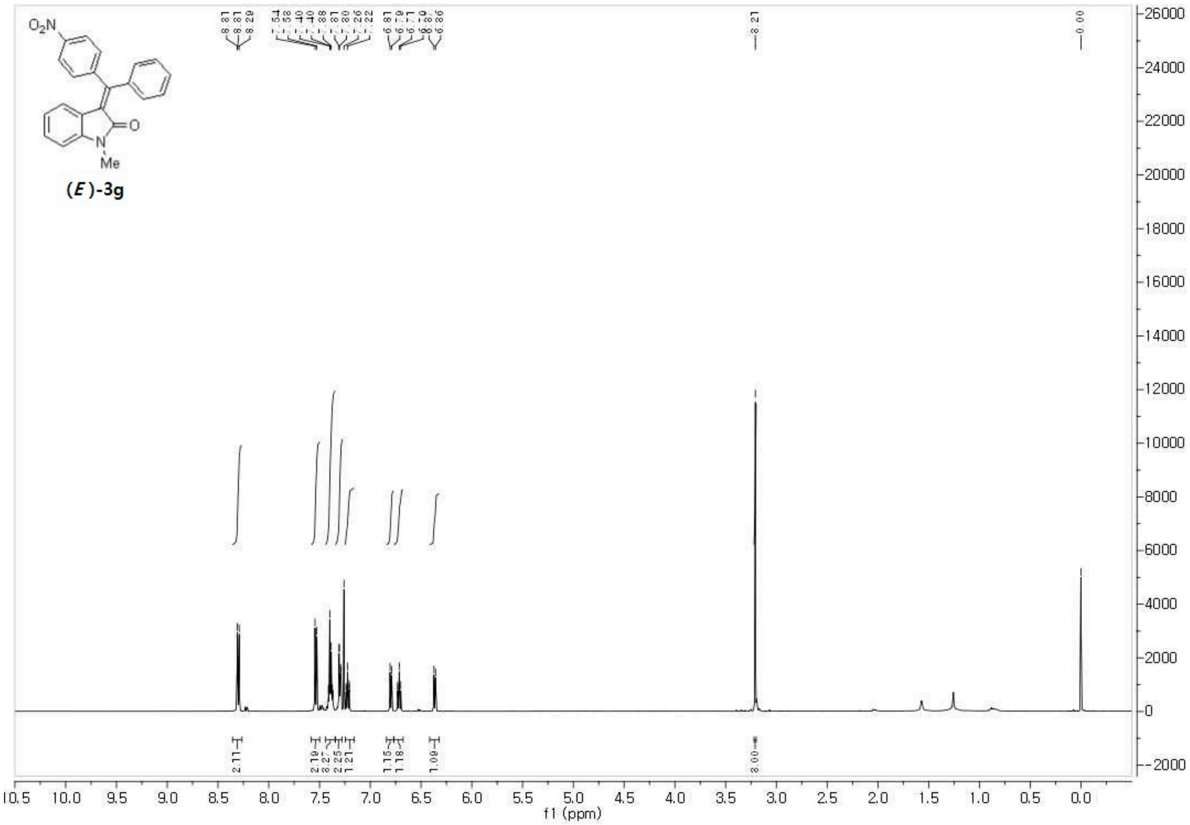

^{13}C NMR spectrum of (*E*)-3g

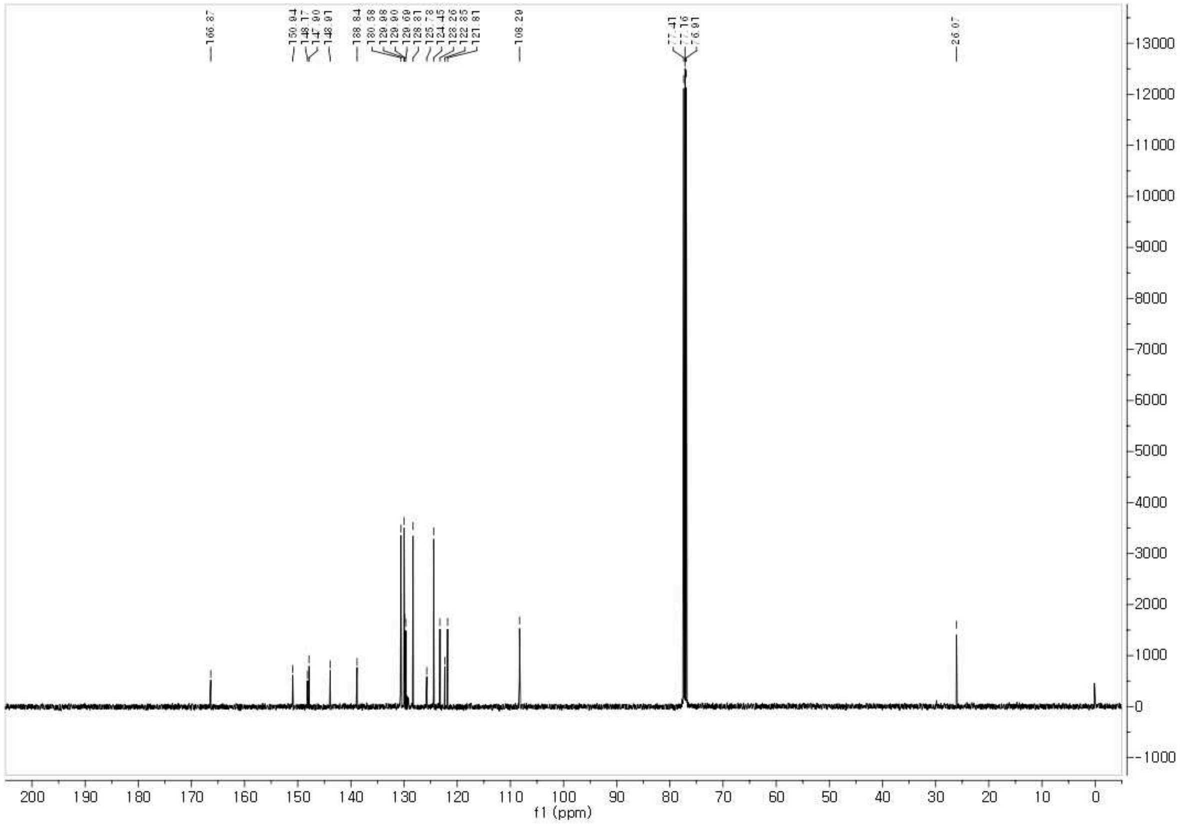

(*Z*)-1-methyl-3-((4-nitrophenyl)(phenyl)methylene)indolin-2-one ((*Z*)-3g)

^{1}H NMR spectrum of (*Z*)-3g

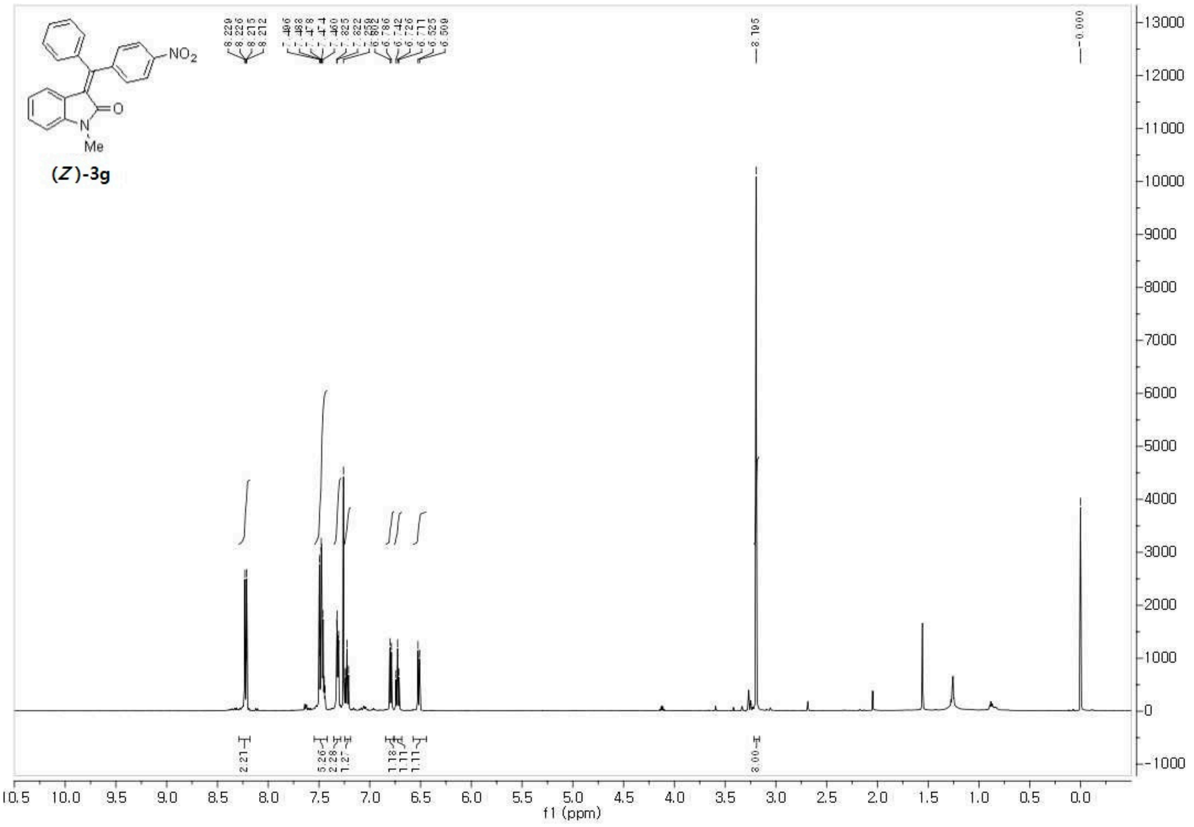

^{13}C NMR spectrum of (*Z*)-3g

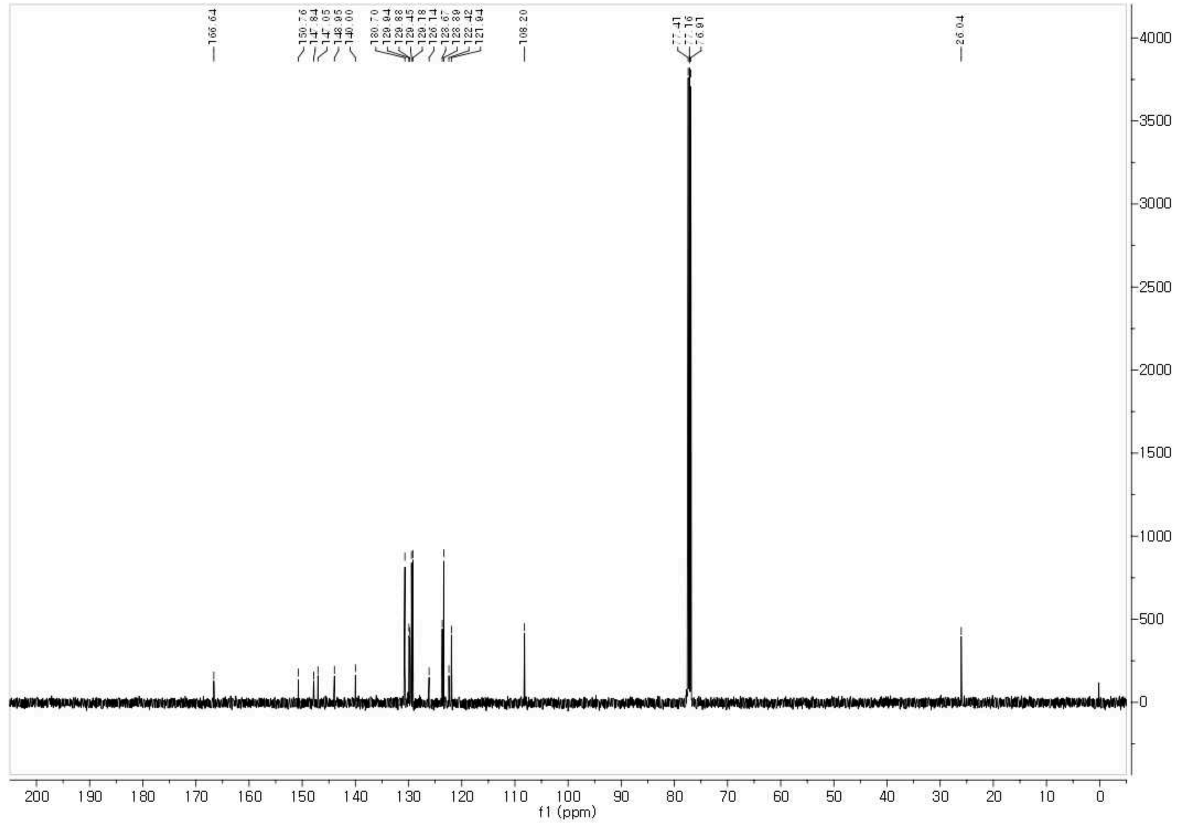

(*Z*)-4-((1-methyl-2-oxoindolin-3-ylidene)(phenyl)methyl)phenyl acetate ((*Z*)-3h)

^{1}H NMR spectrum of (*Z*)-3h

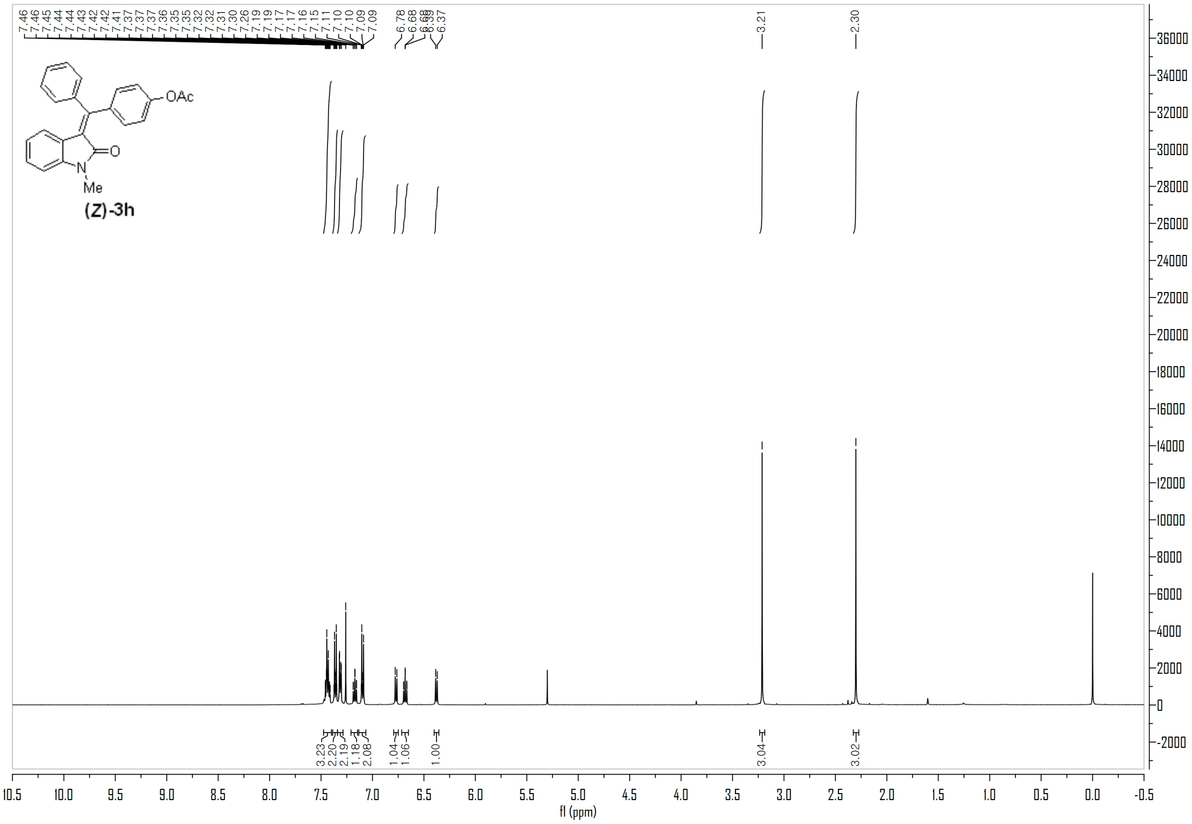

^{13}C NMR spectrum of (*Z*)-3h

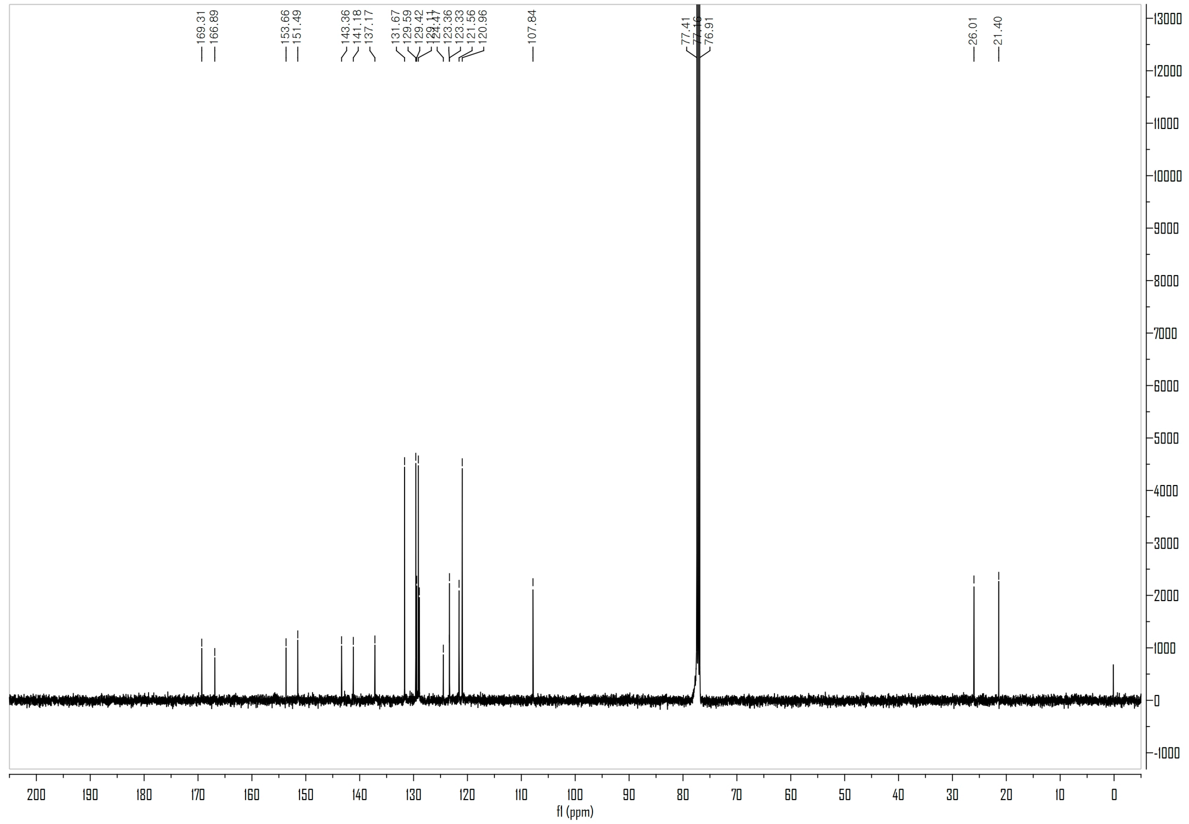

HSQC spectrum of (*Z*)-3h

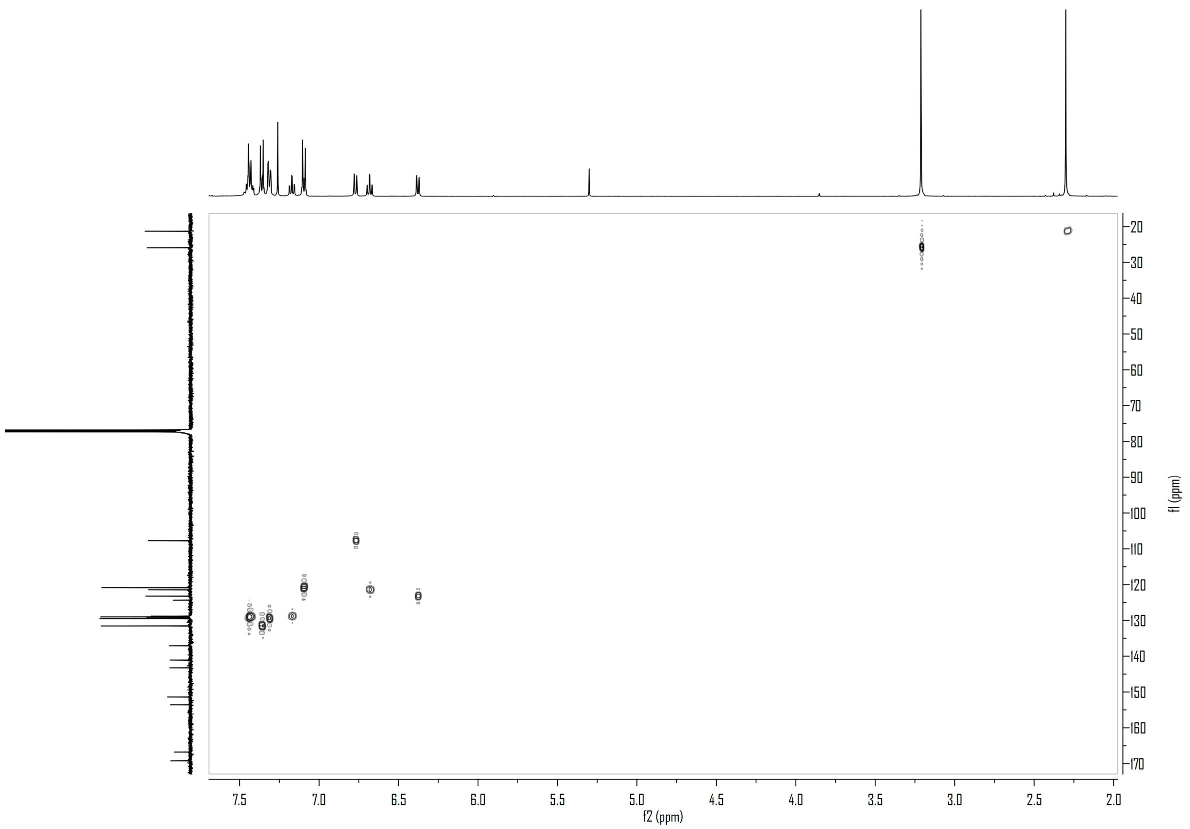

HMBC spectrum of (*Z*)-3h

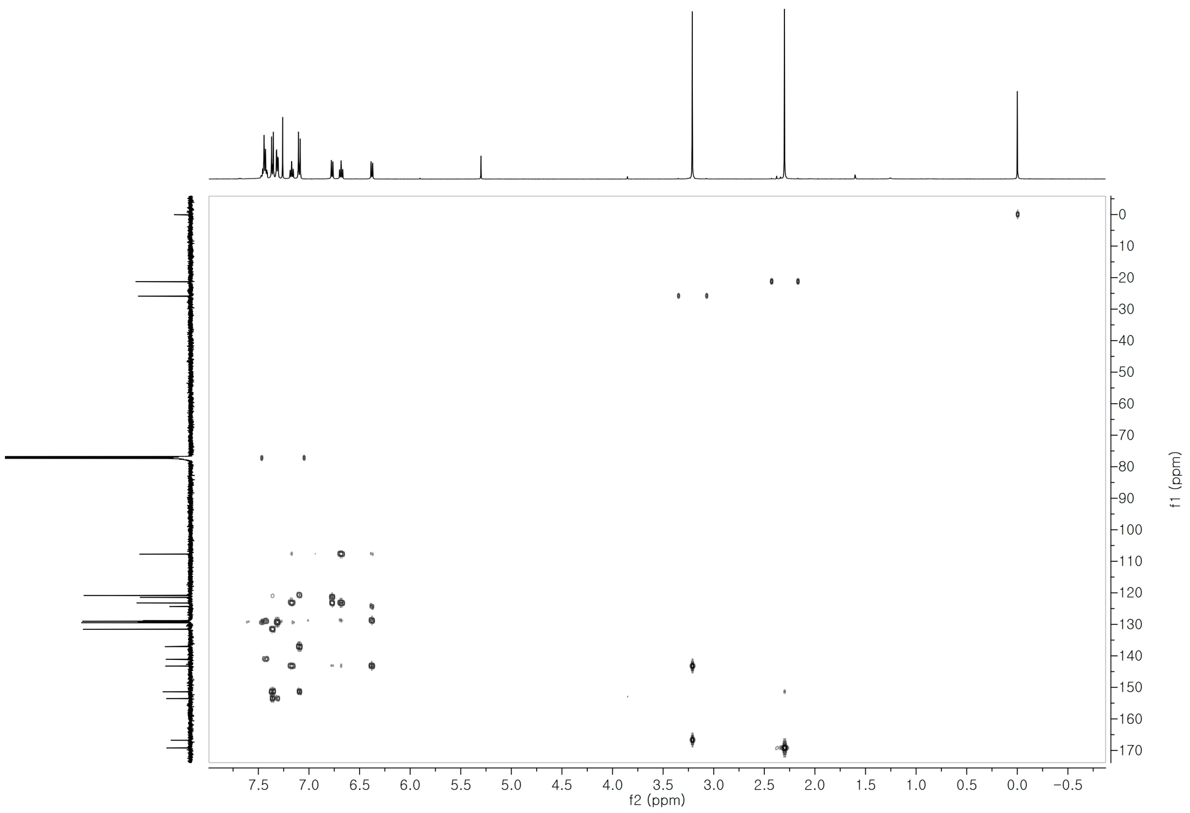

COSY spectrum of (*Z*)-3h

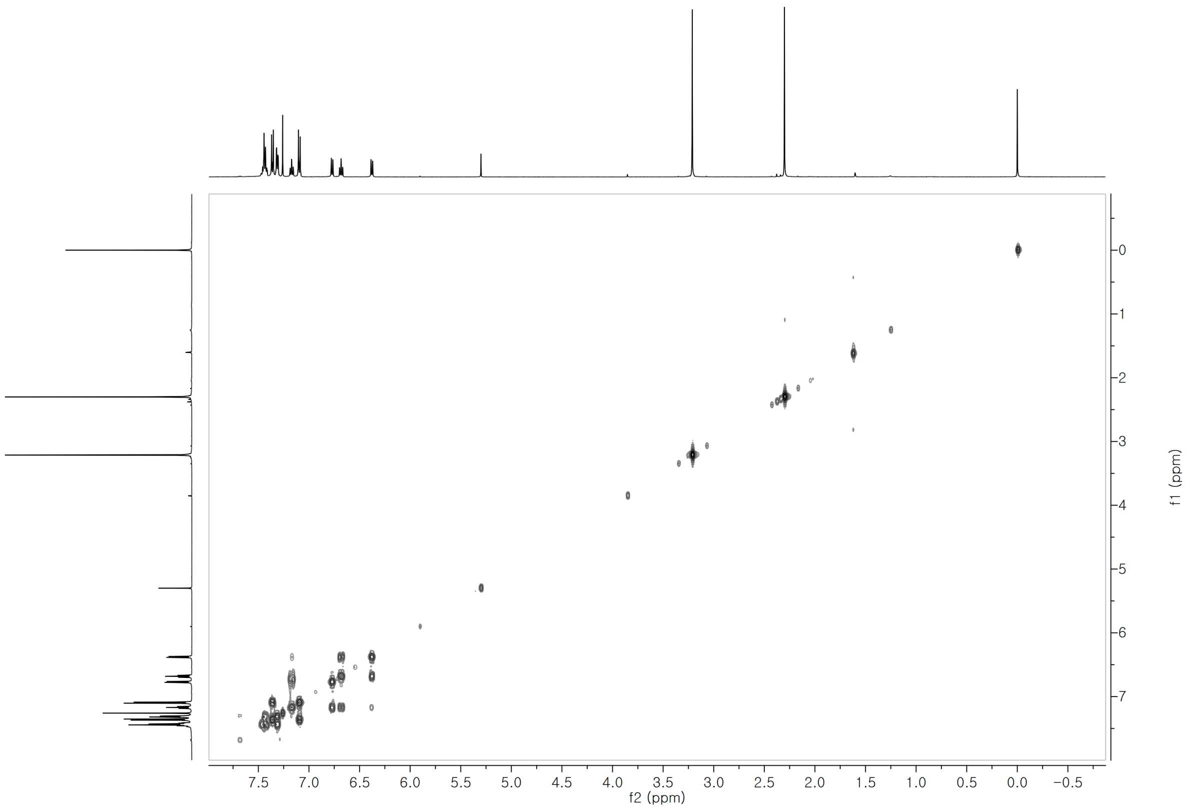

ROESY spectrum of (*Z*)-3h

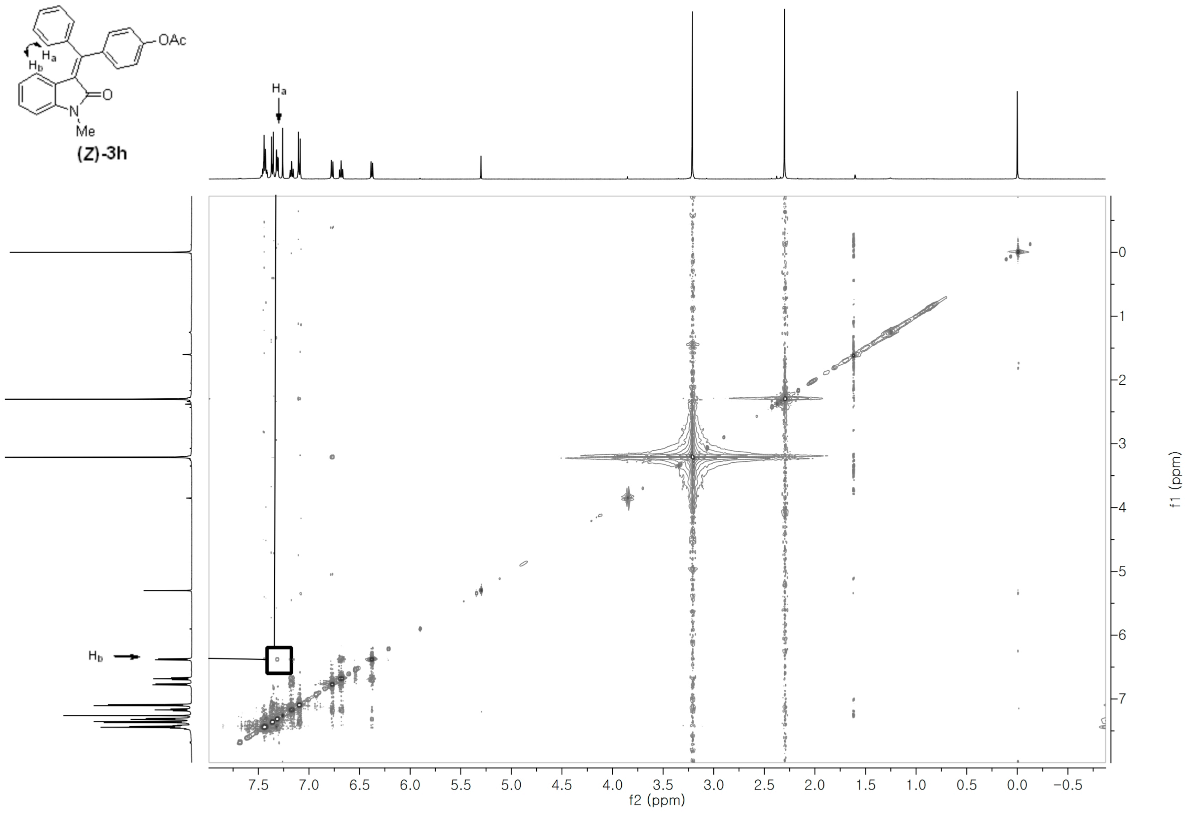

(E)-4-((1-methyl-2-oxoindolin-3-ylidene)(phenyl)methyl)phenyl acetate ((E)-3h)

^{1}H NMR spectrum of (E)-3h

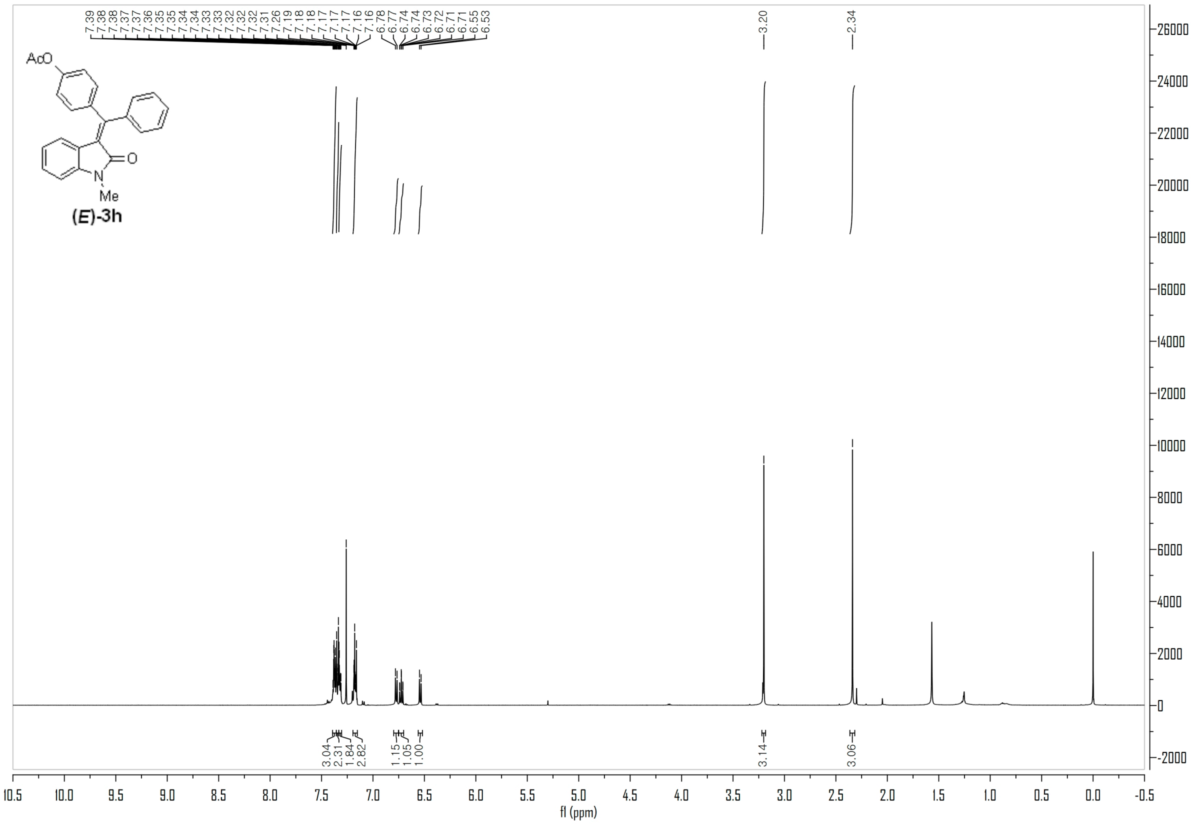

^{13}C NMR spectrum of (E)-3h

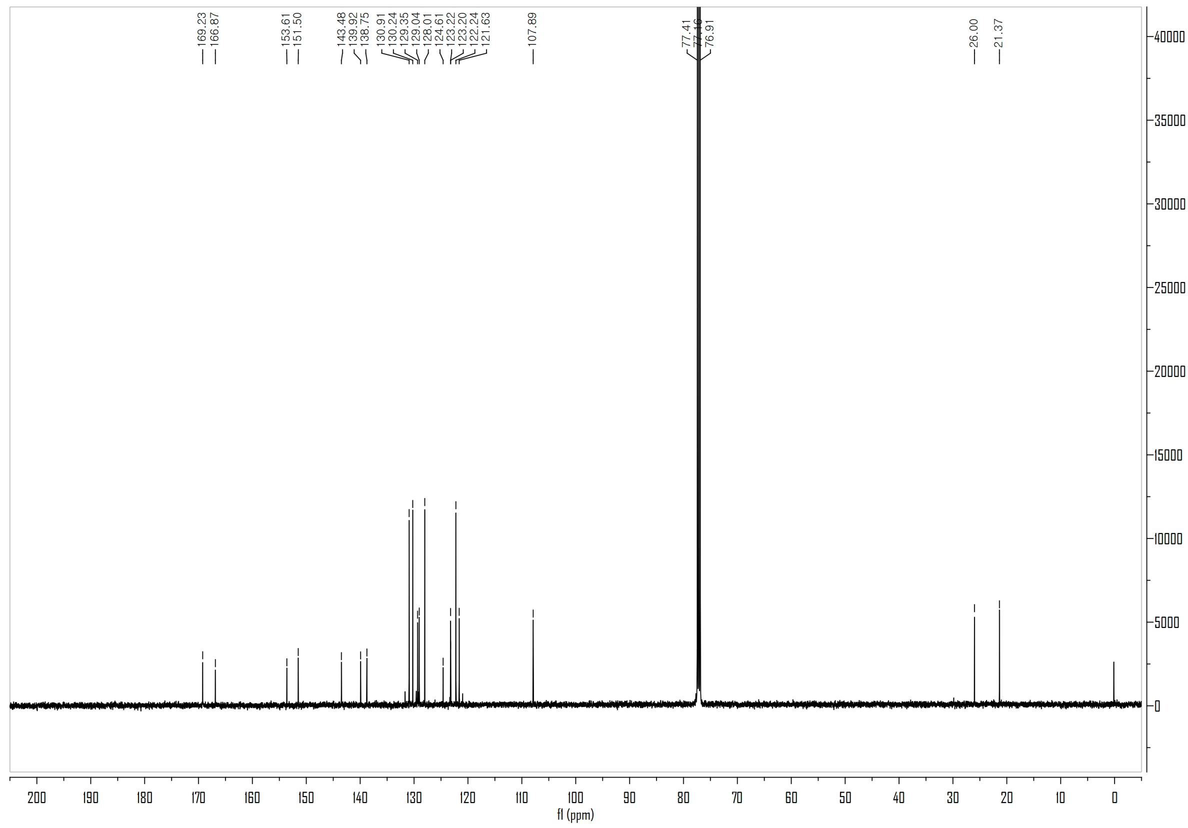

HSQC spectrum of (*E*)-3h

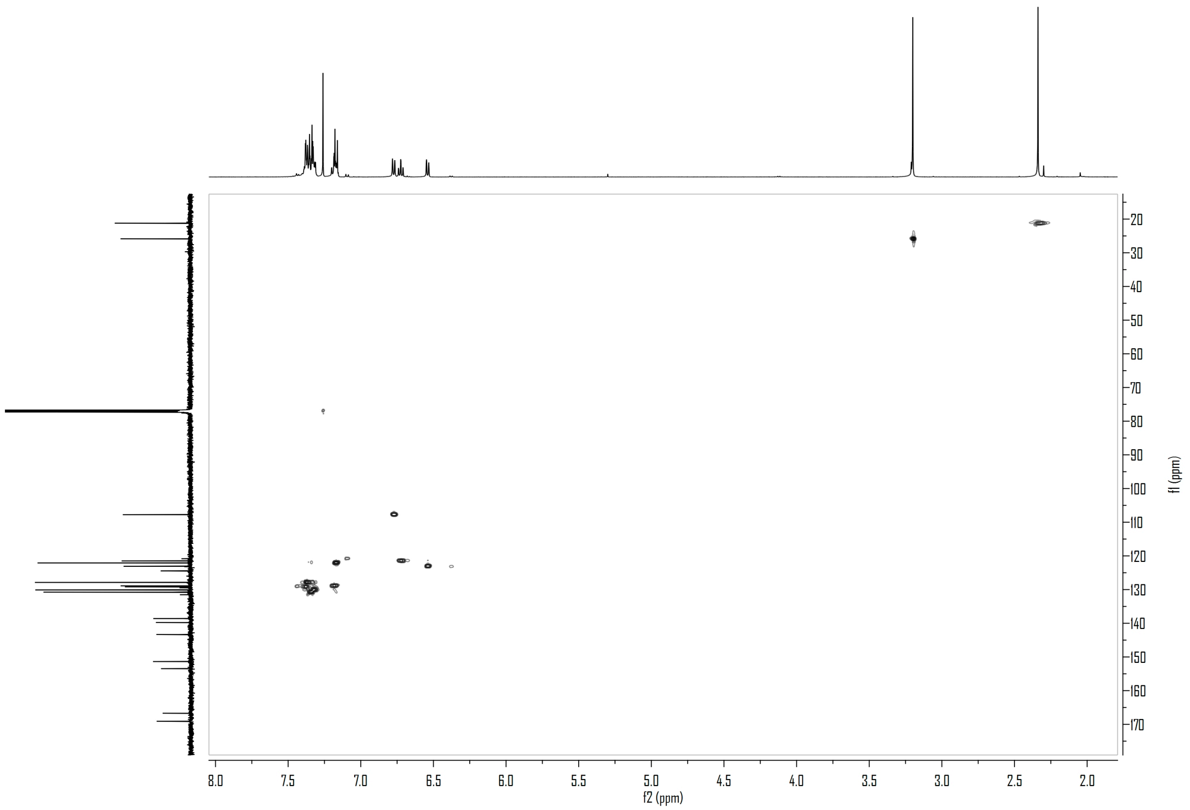

HMBC spectrum of (*E*)-3h

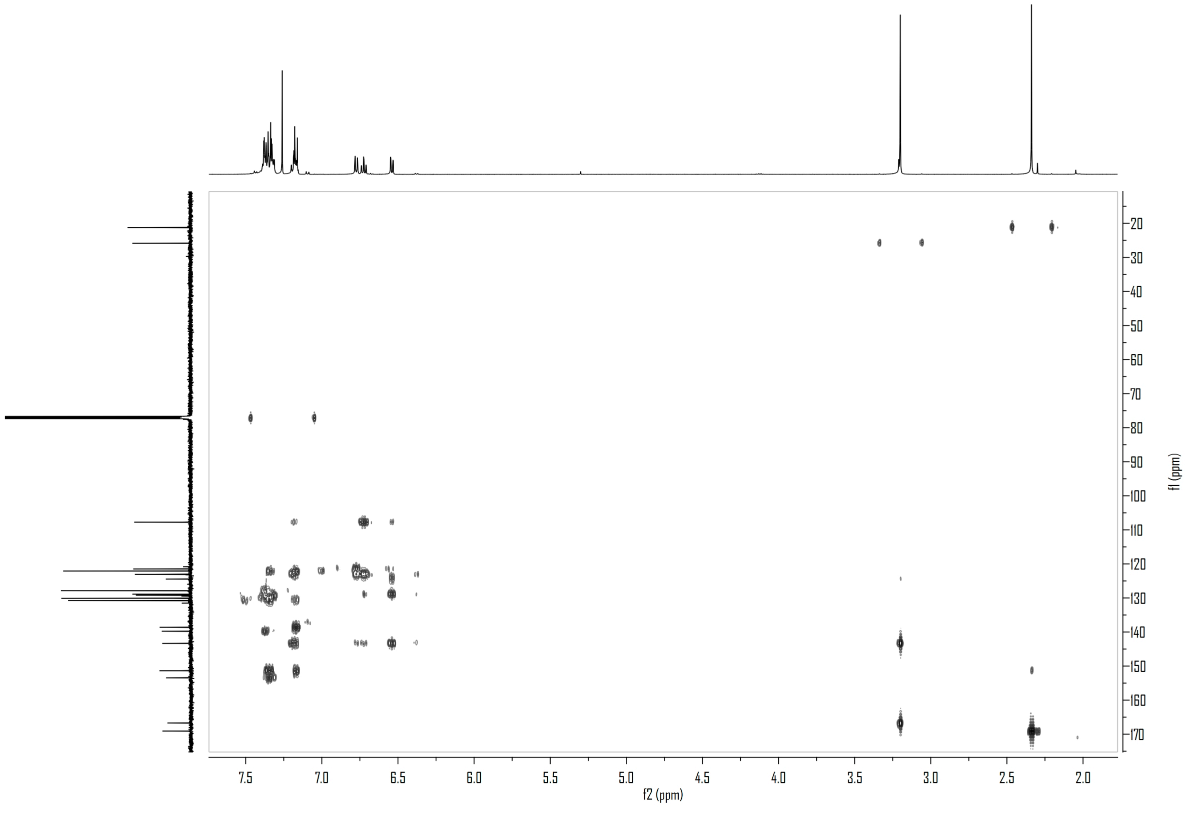

COSY spectrum of (*E*)-3h

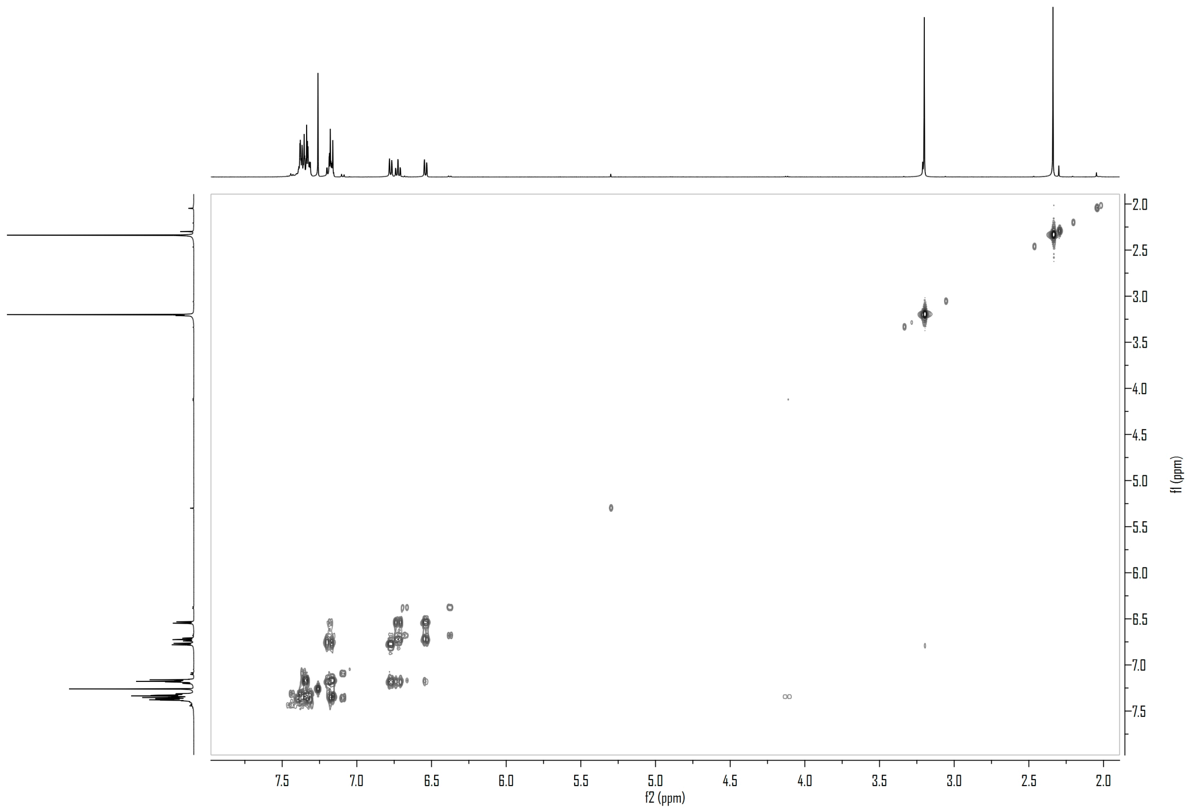

ROESY spectrum (*E*)-3h

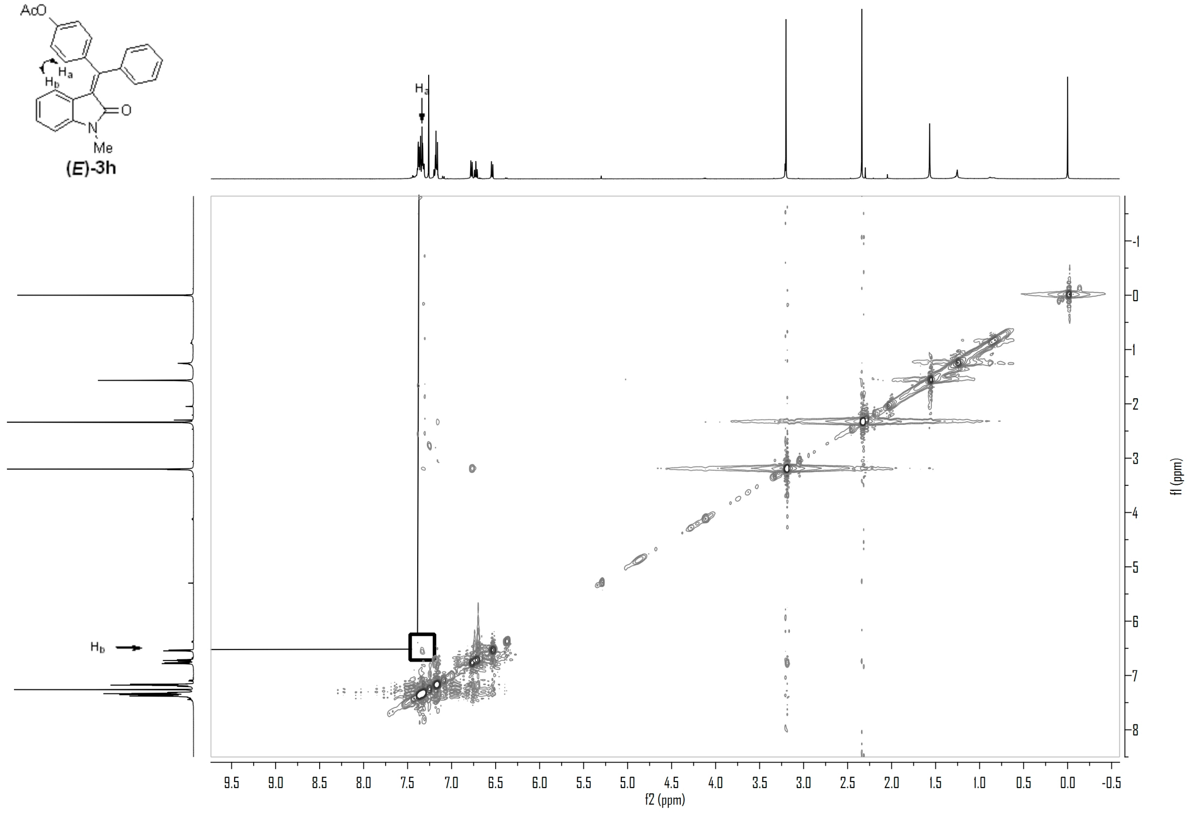

(*Z*)-3-((3-methoxyphenyl)(phenyl)methylene)-1-methylindolin-2-one ((*Z*)-3i)

^{1}H NMR spectrum of (*Z*)-3i

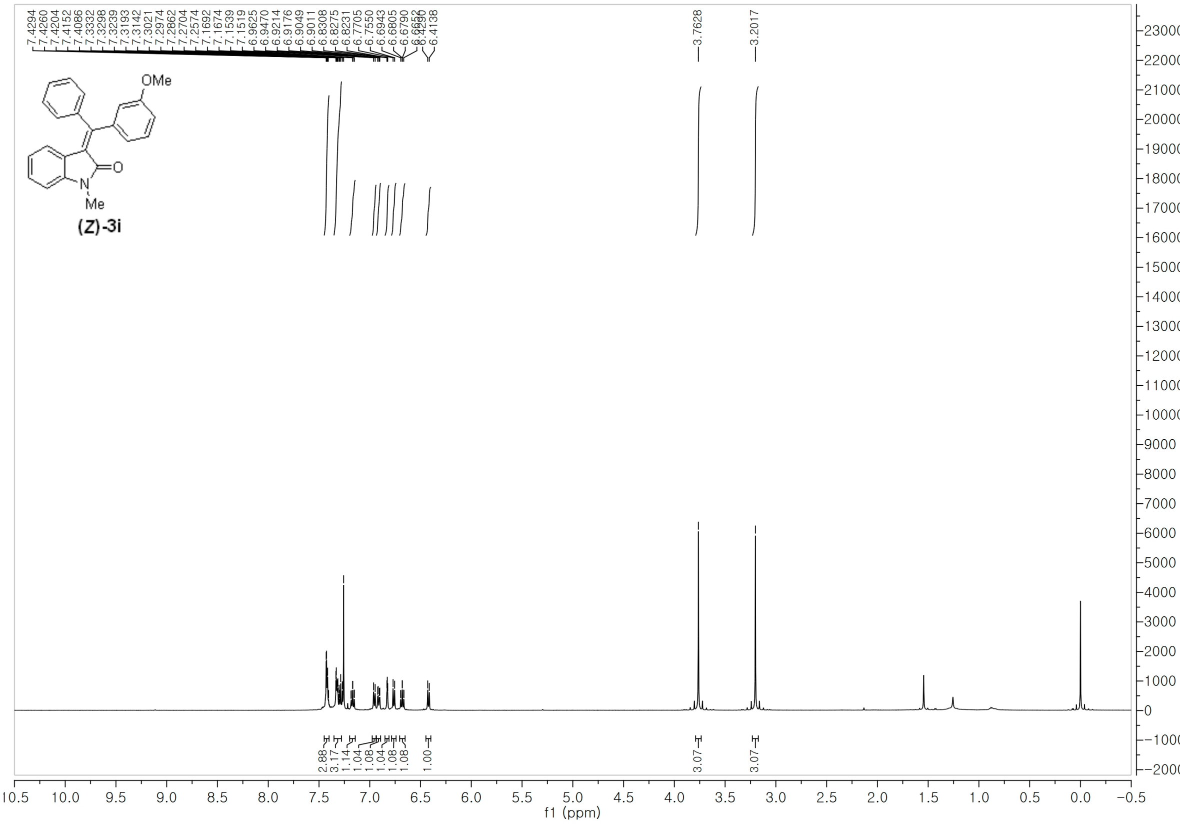

^{13}C NMR spectrum of (*Z*)-3i

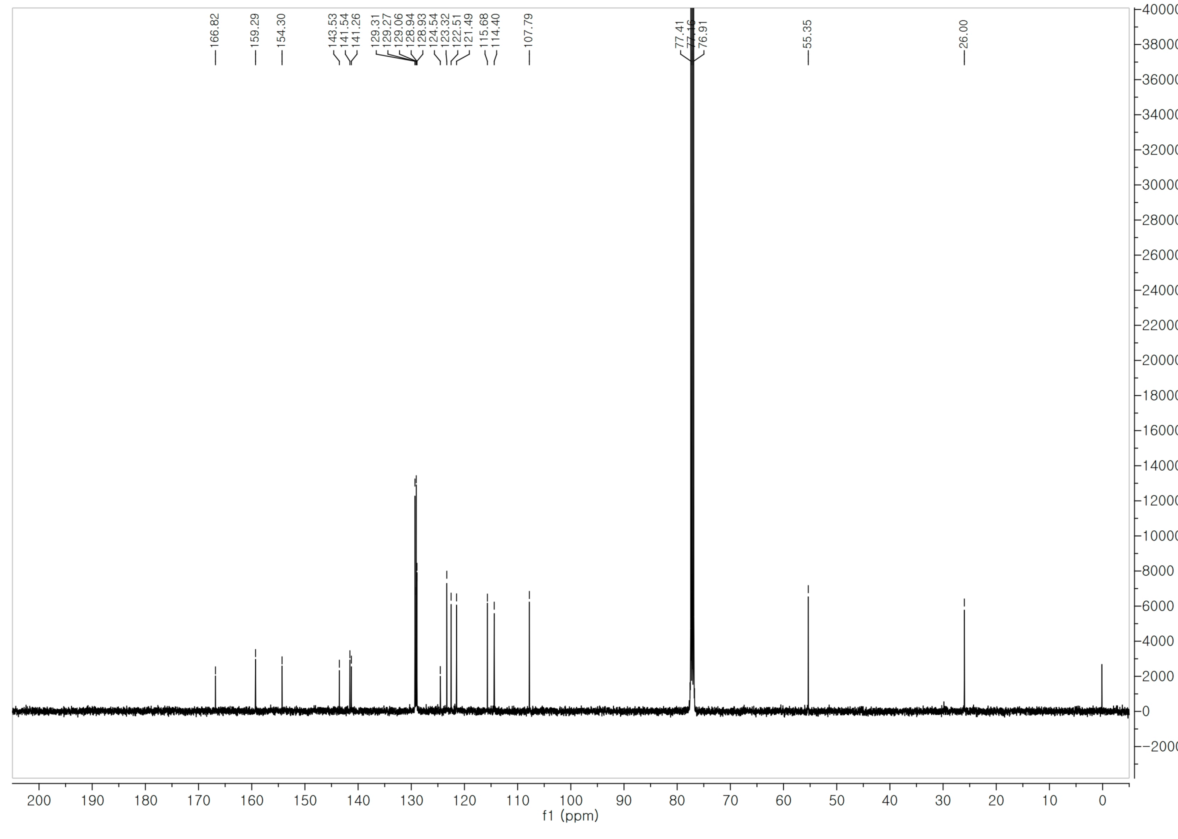

HSQC spectrum of (*Z*)-3i

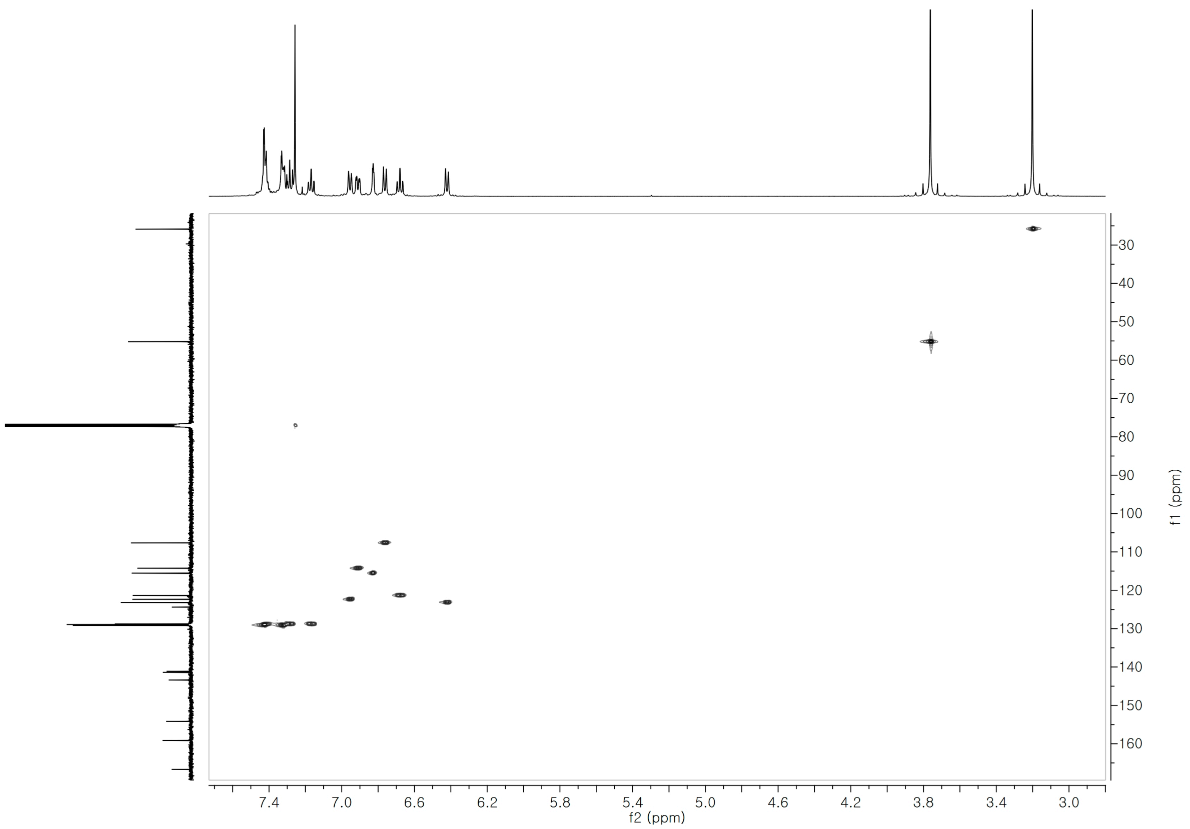

HMBC spectrum of (*Z*)-3i

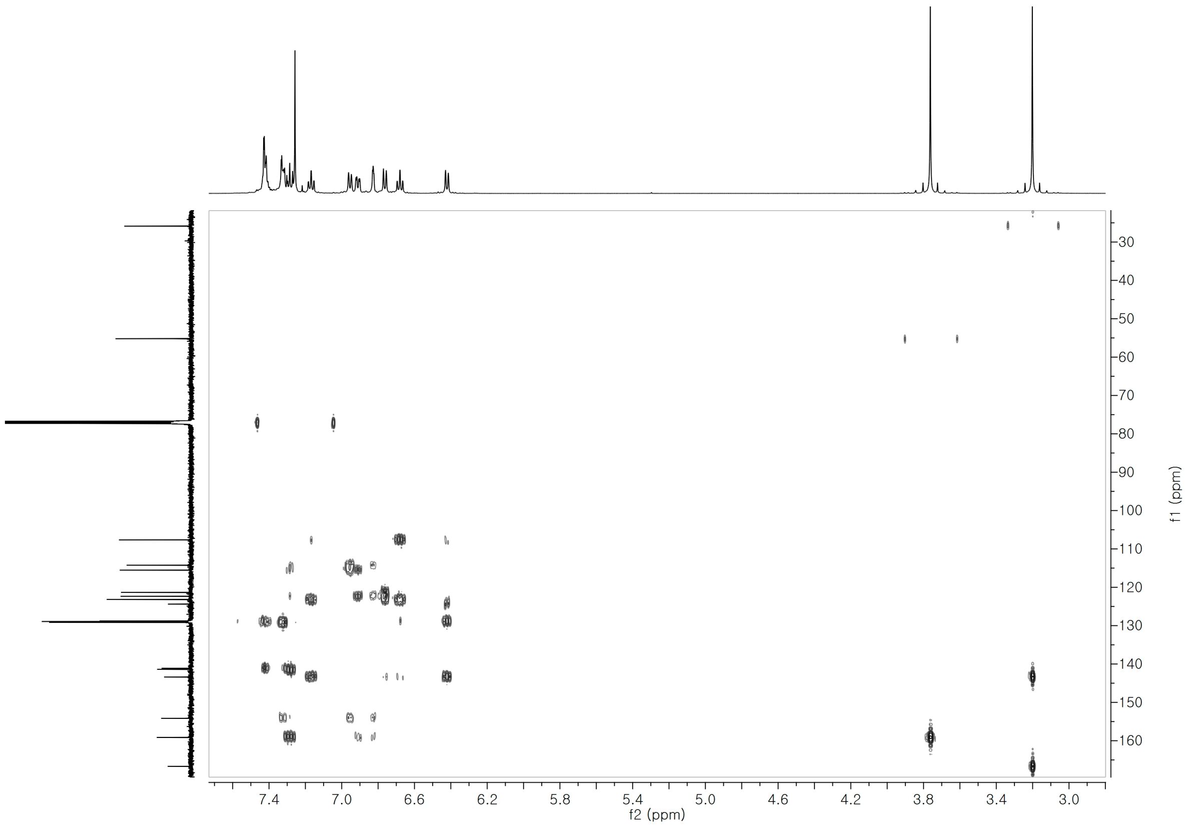

COSY spectrum of (*Z*)-3i

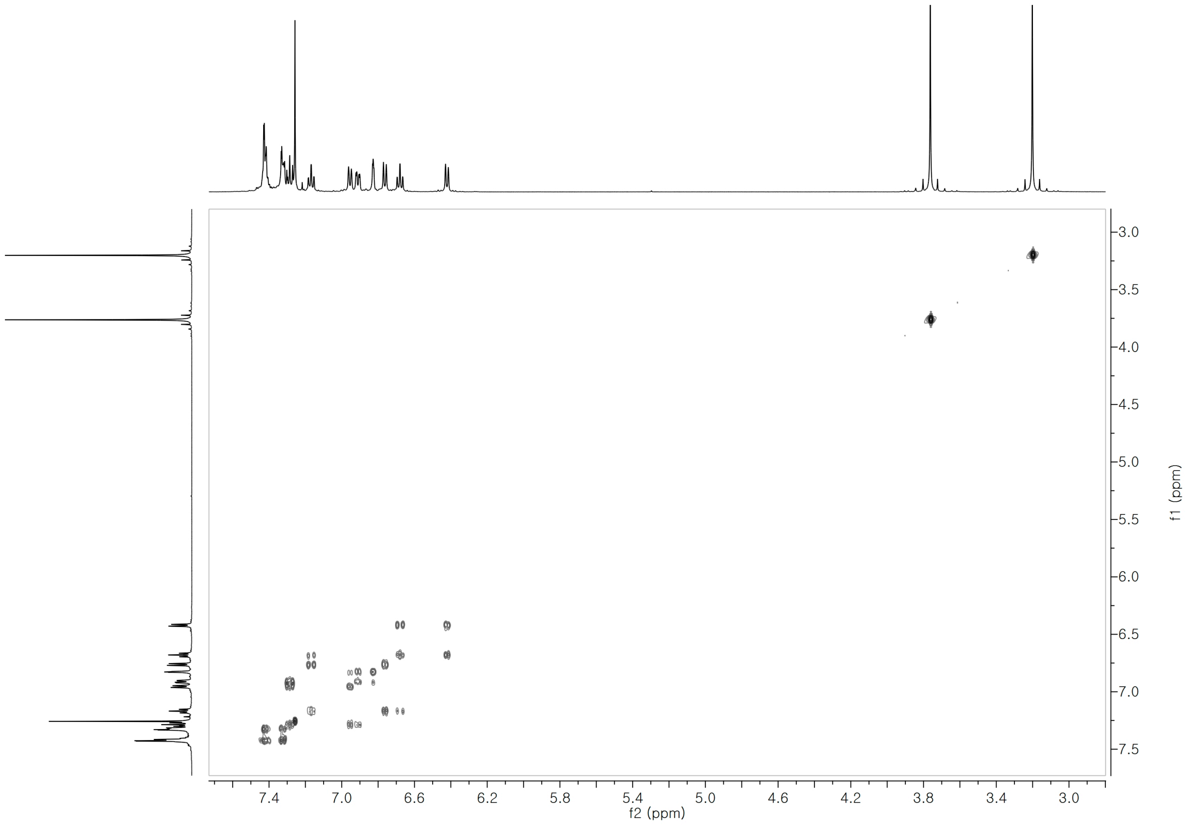

ROESY spectrum of (*Z*)-3i

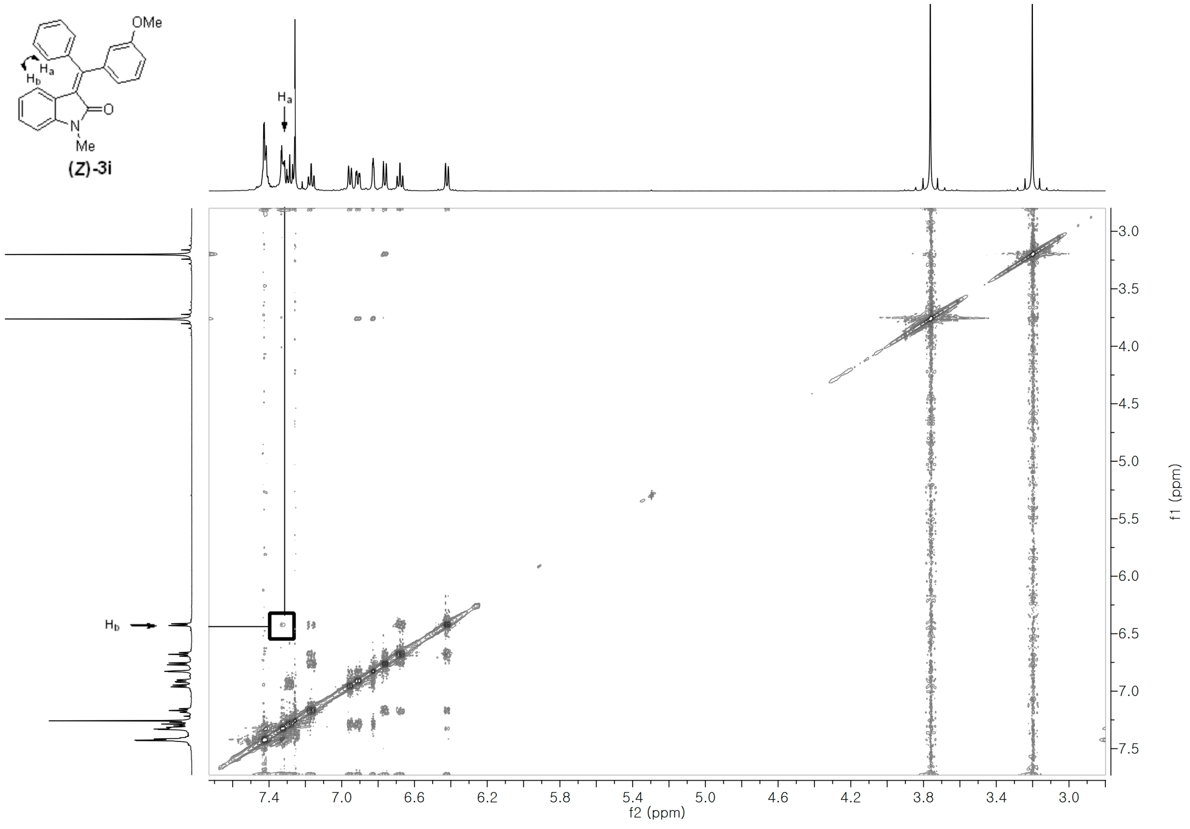

(*E*)-3-((3-methoxyphenyl)(phenyl)methylene)-1-methylindolin-2-one ((*E*)-3i)

^{1}H NMR spectrum of (*E*)-3i

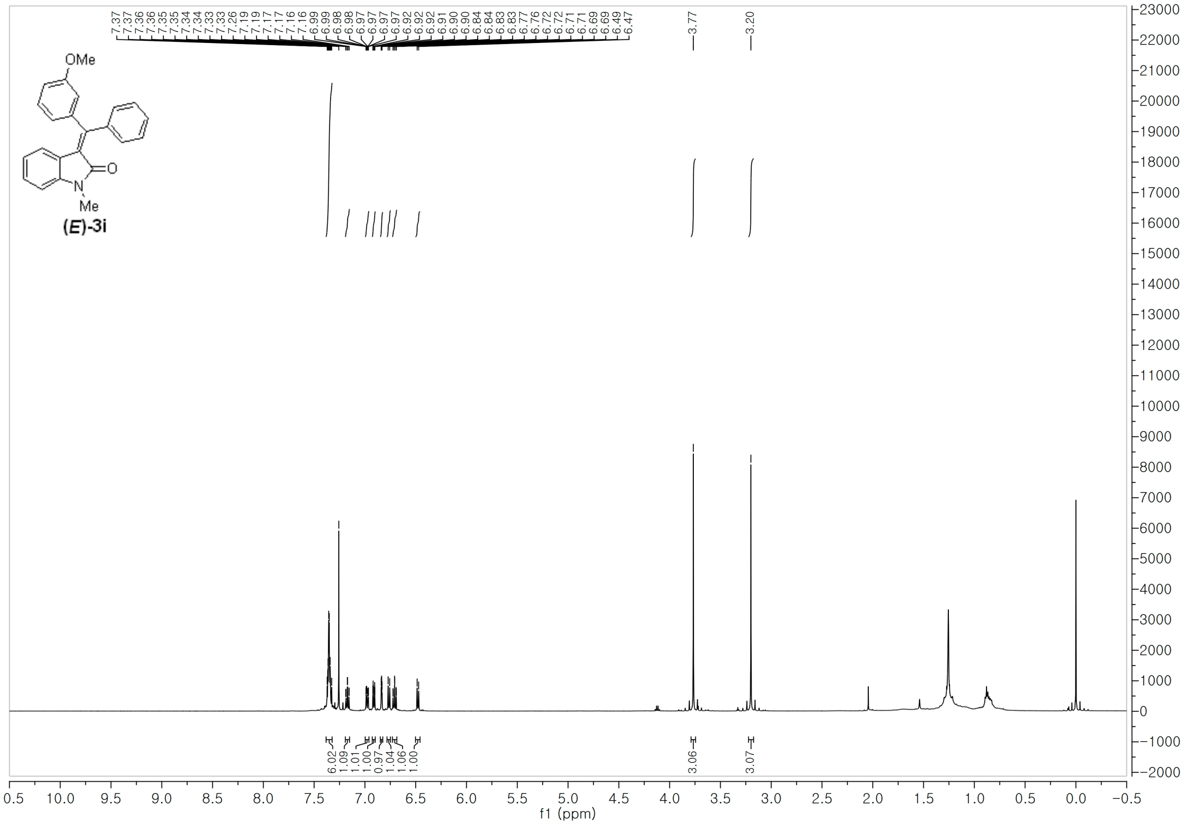

^{13}C NMR spectrum of (*E*)-3i

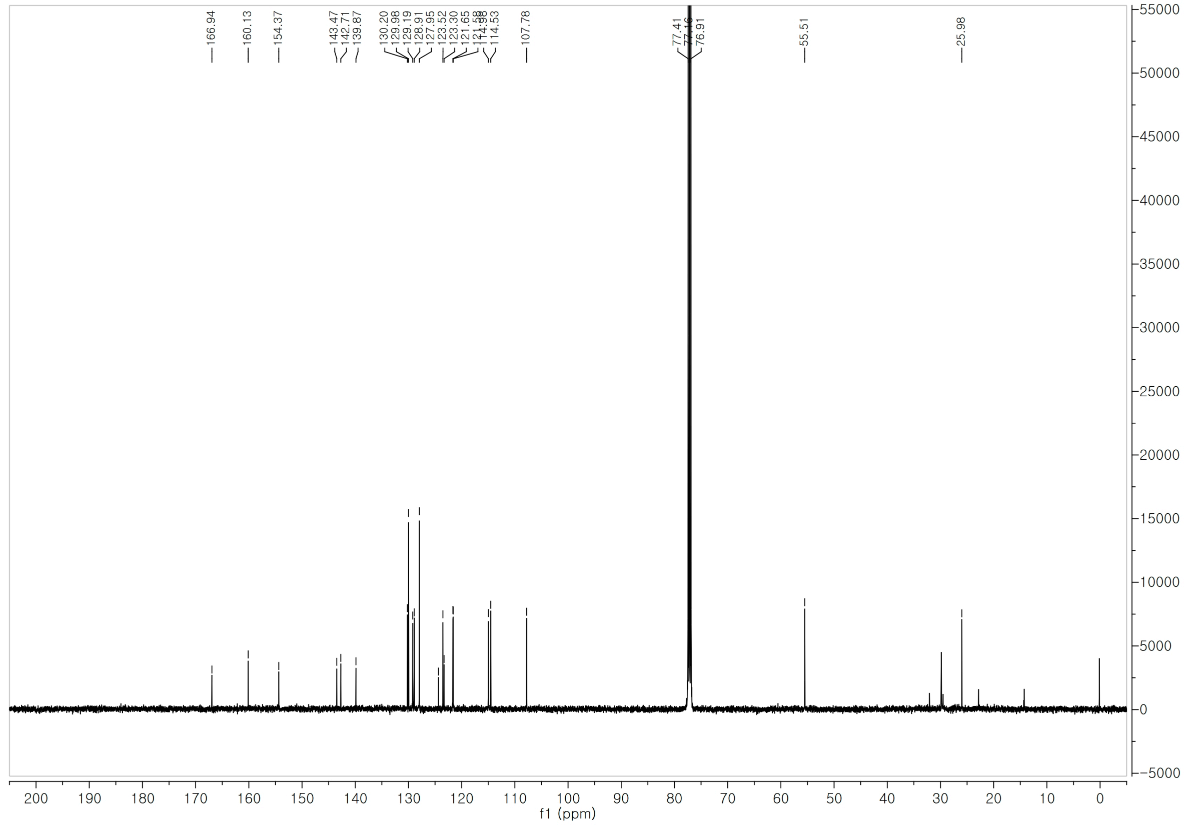

HSQC spectrum of (*E*)-3i

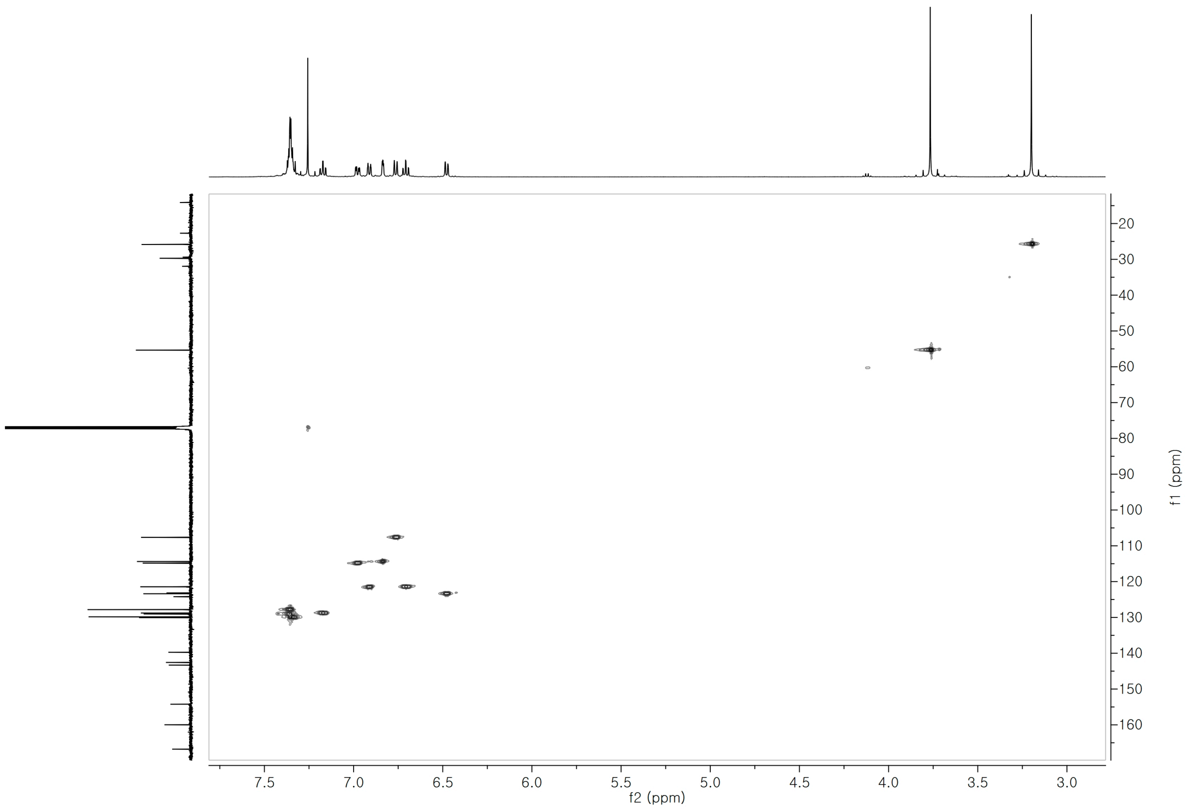

HMBC spectrum of (*E*)-3i

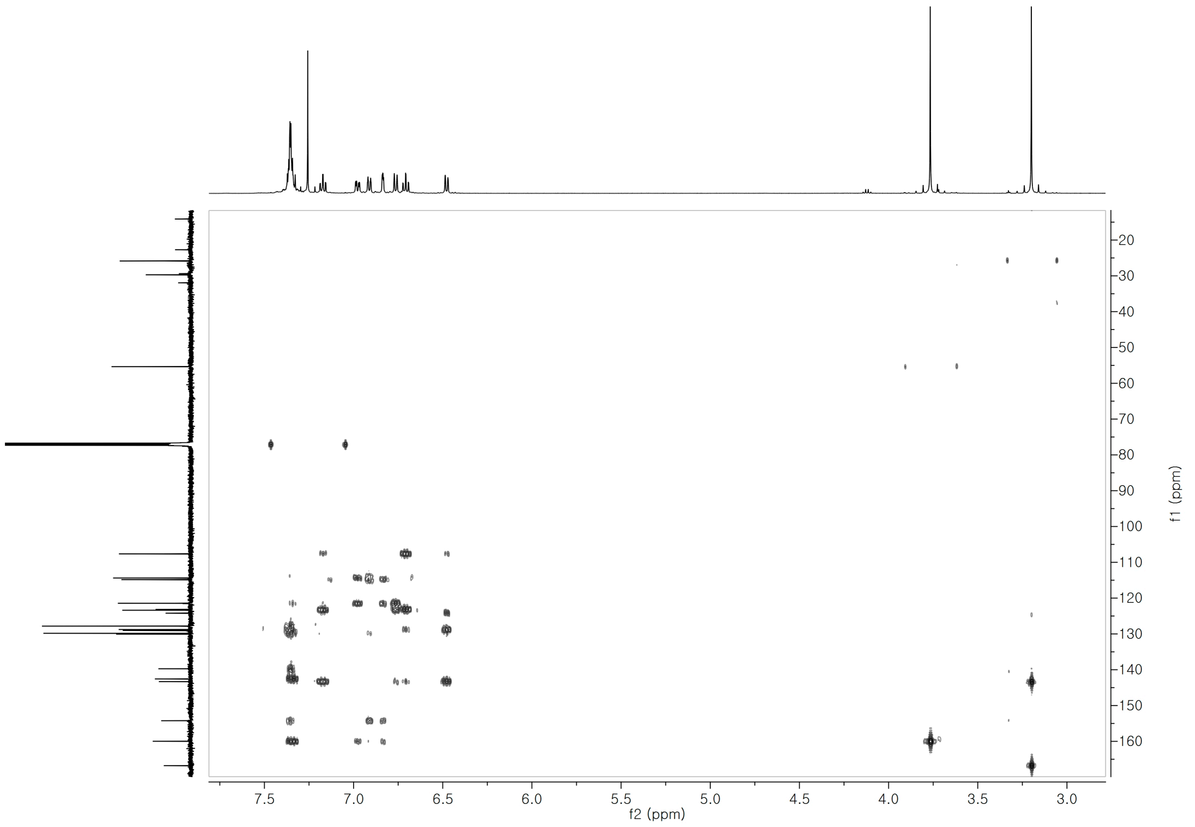

COSY spectrum of (*E*)-3i

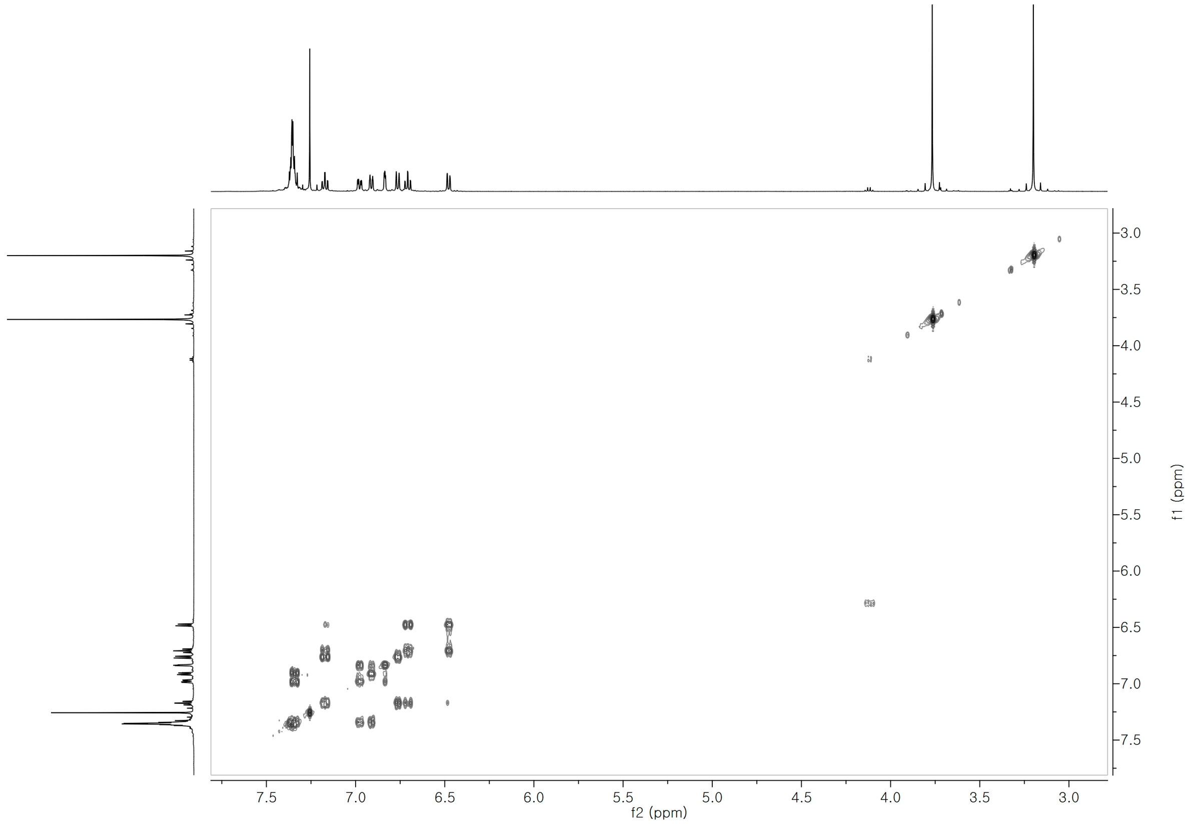

ROESY spectrum of (*E*)-3i

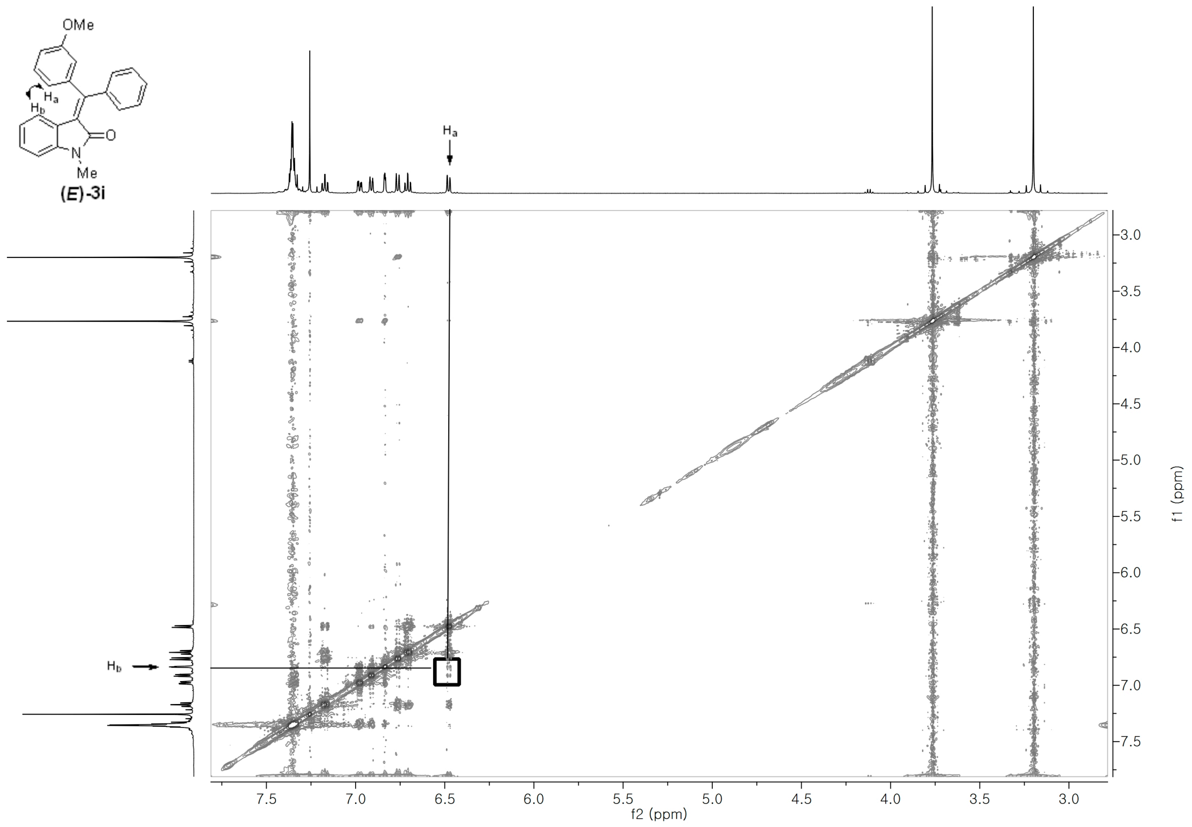

(*Z*)-3-((3-chlorophenyl)(phenyl)methylene)-1-methylindolin-2-one ((*Z*)-3j)

^{1}H NMR spectrum of (*Z*)-3j

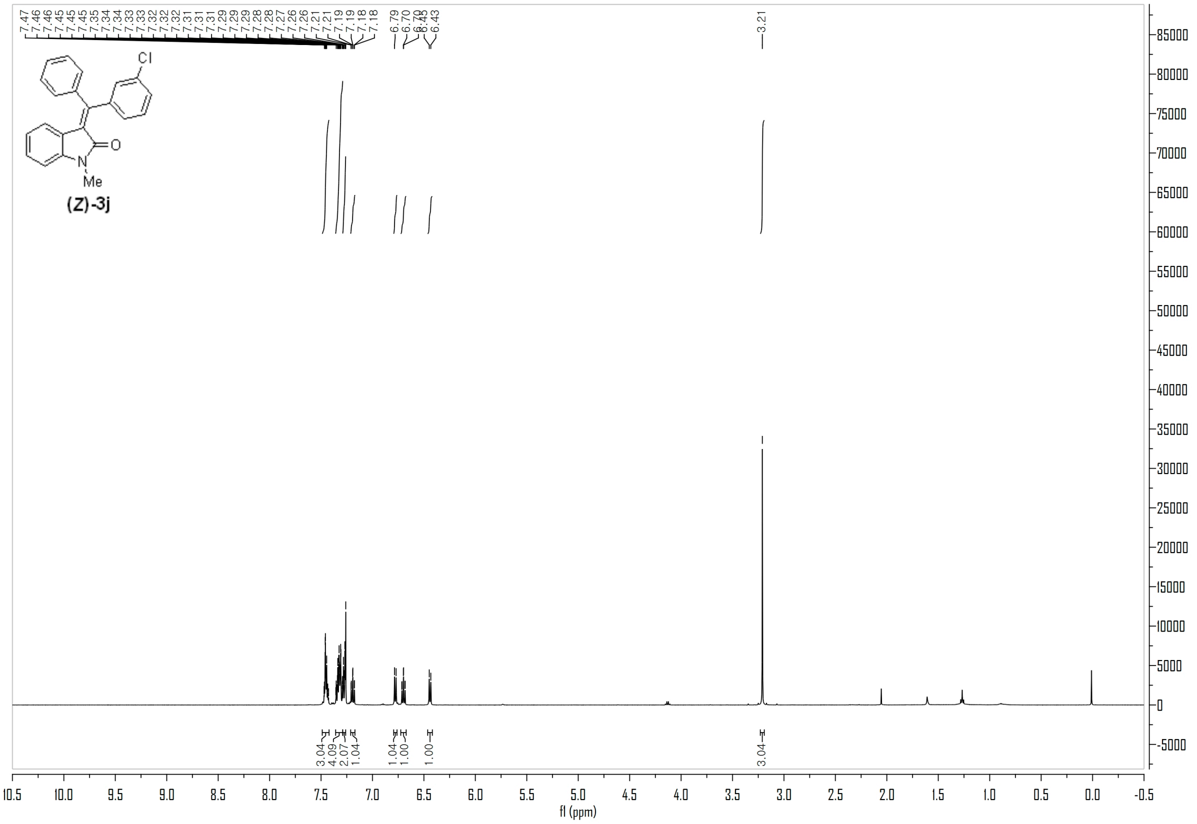

^{13}C NMR spectrum of (*Z*)-3j

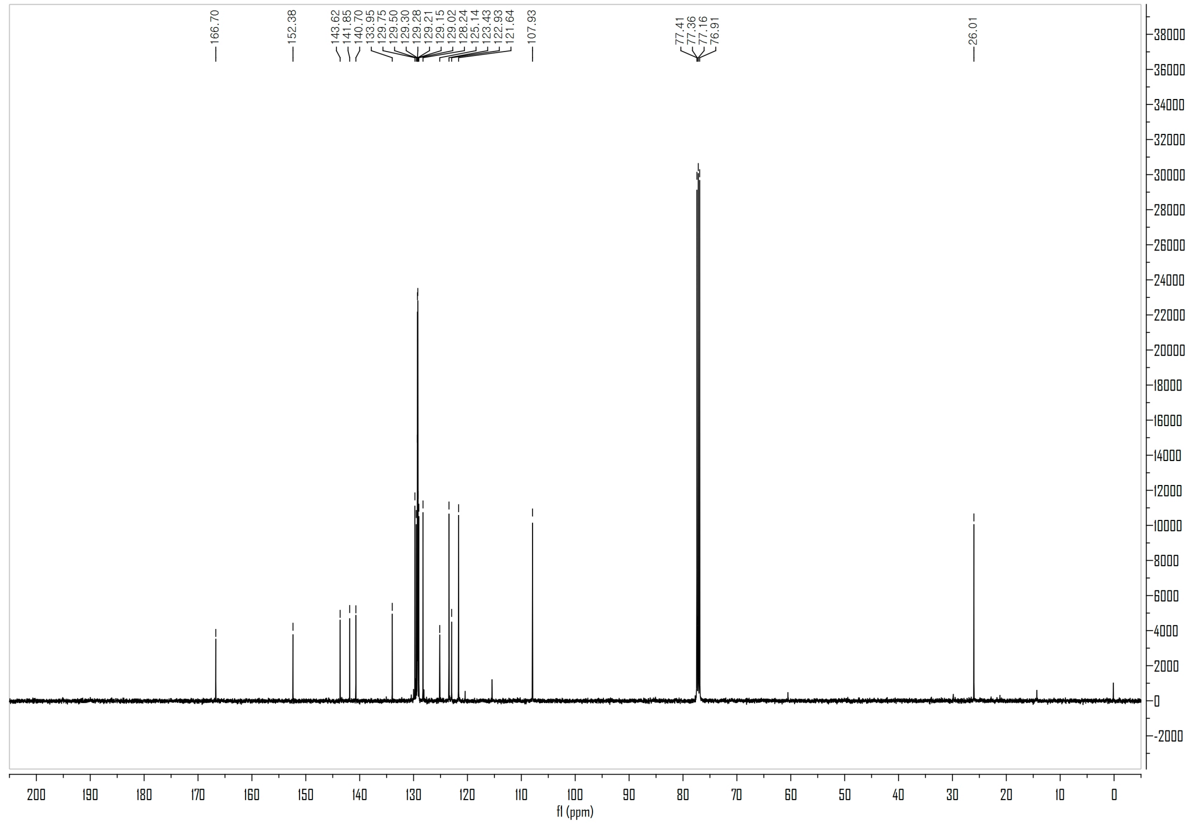

HSQC spectrum of (*Z*)-3j

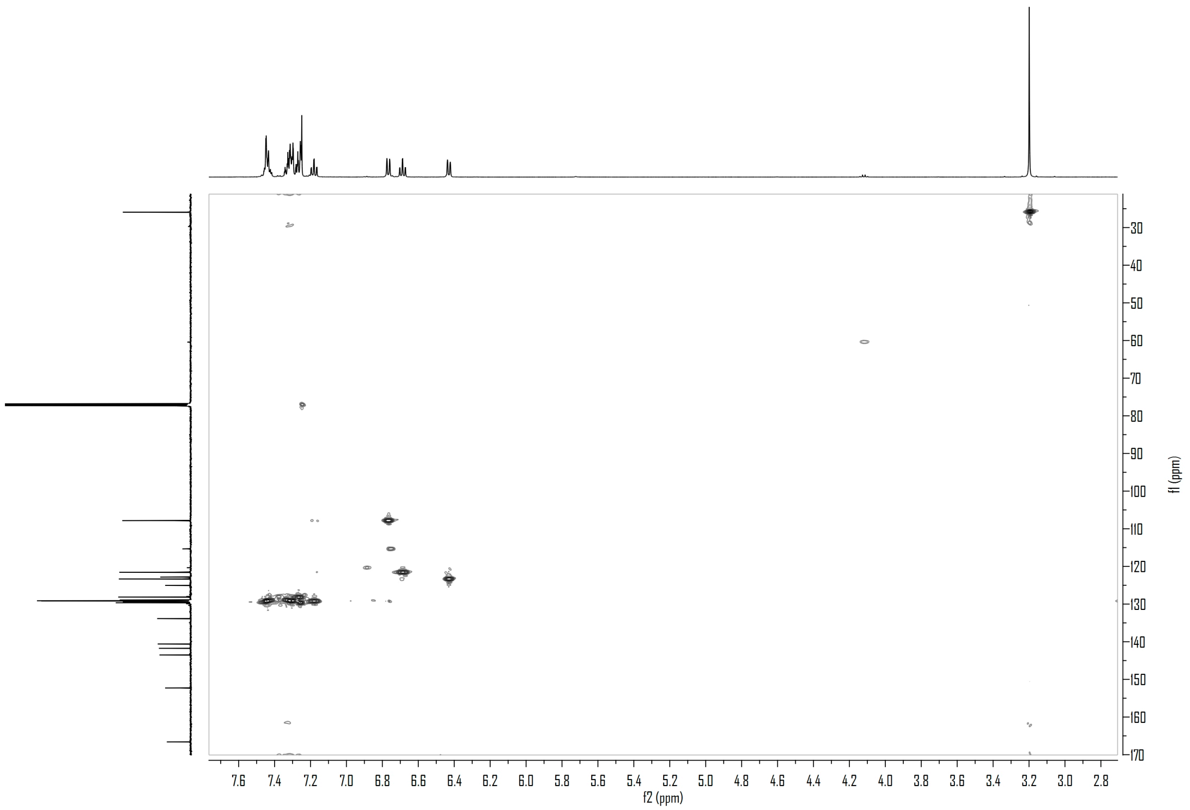

HMBC spectrum of (*Z*)-3j

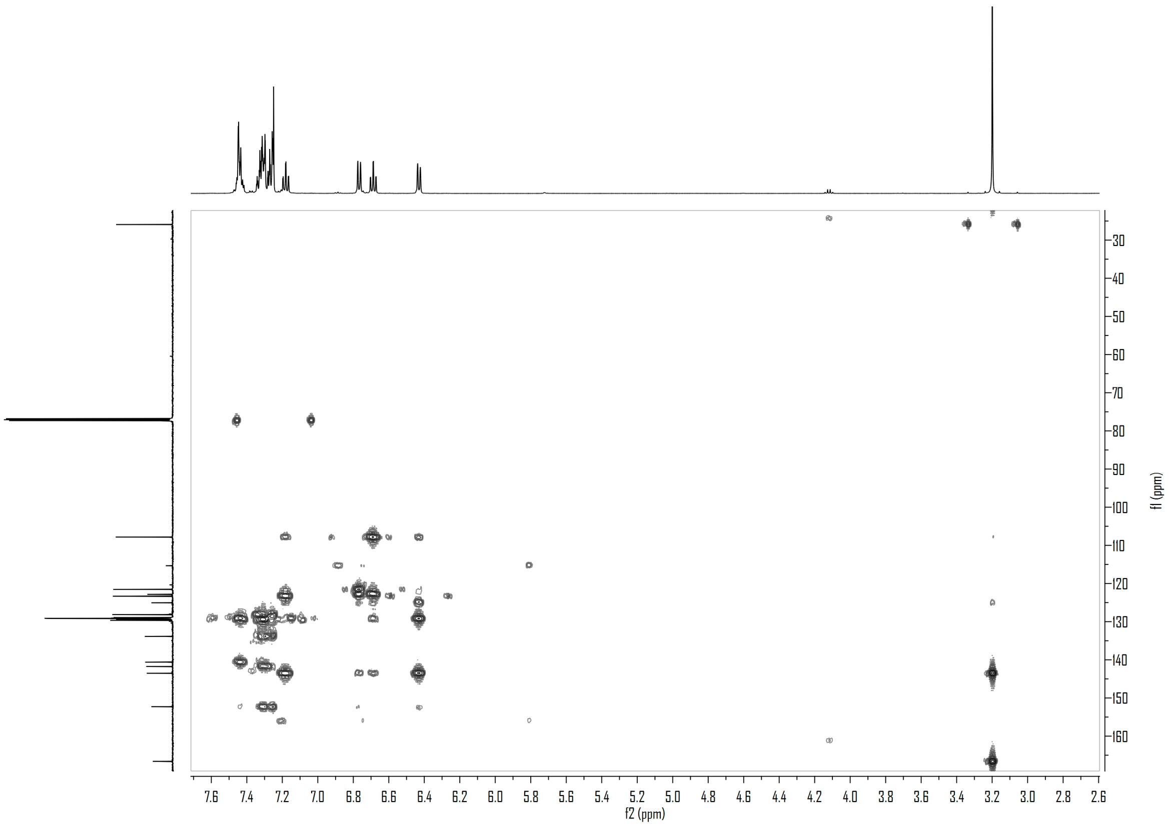

COSY spectrum of (*Z*)-3j

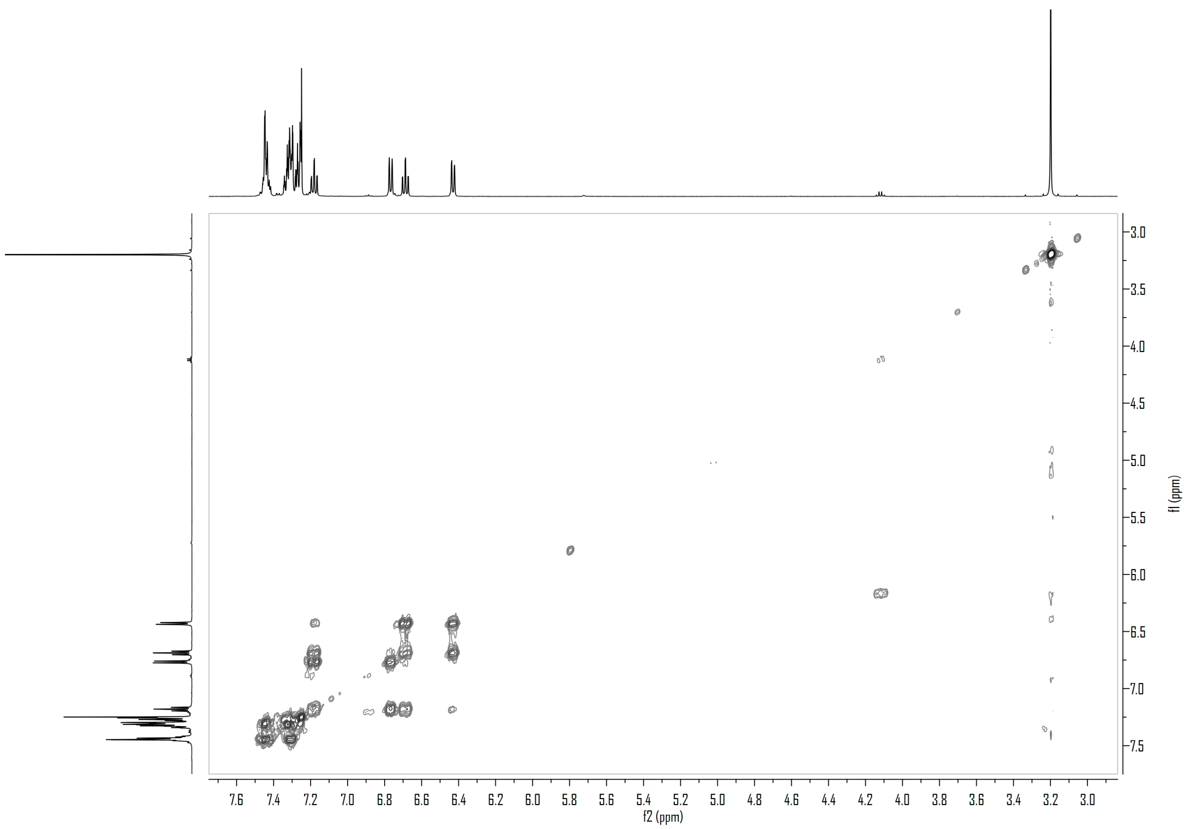

ROESY spectrum of (*Z*)-3j

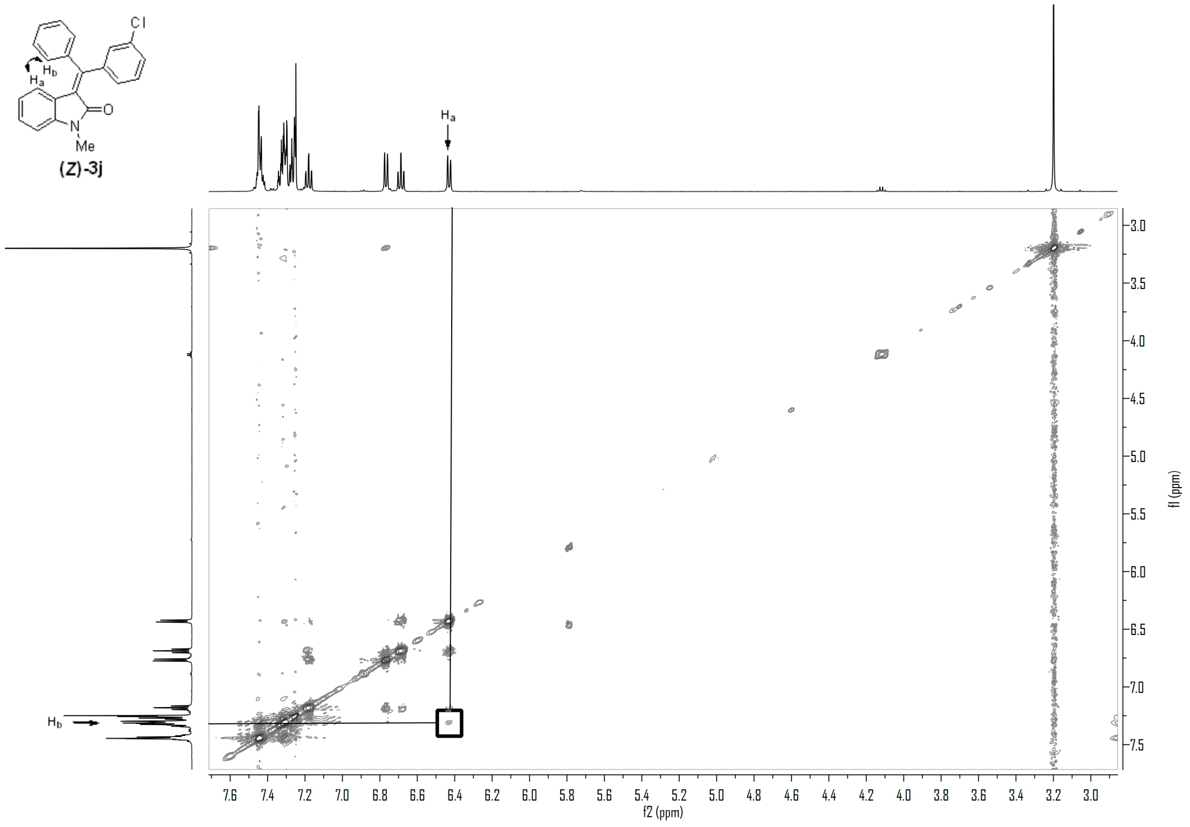

(*E*)-3-((3-chlorophenyl)(phenyl)methylene)-1-methylindolin-2-one ((*E*)-3j)

^{1}H NMR spectrum of (*E*)-3j

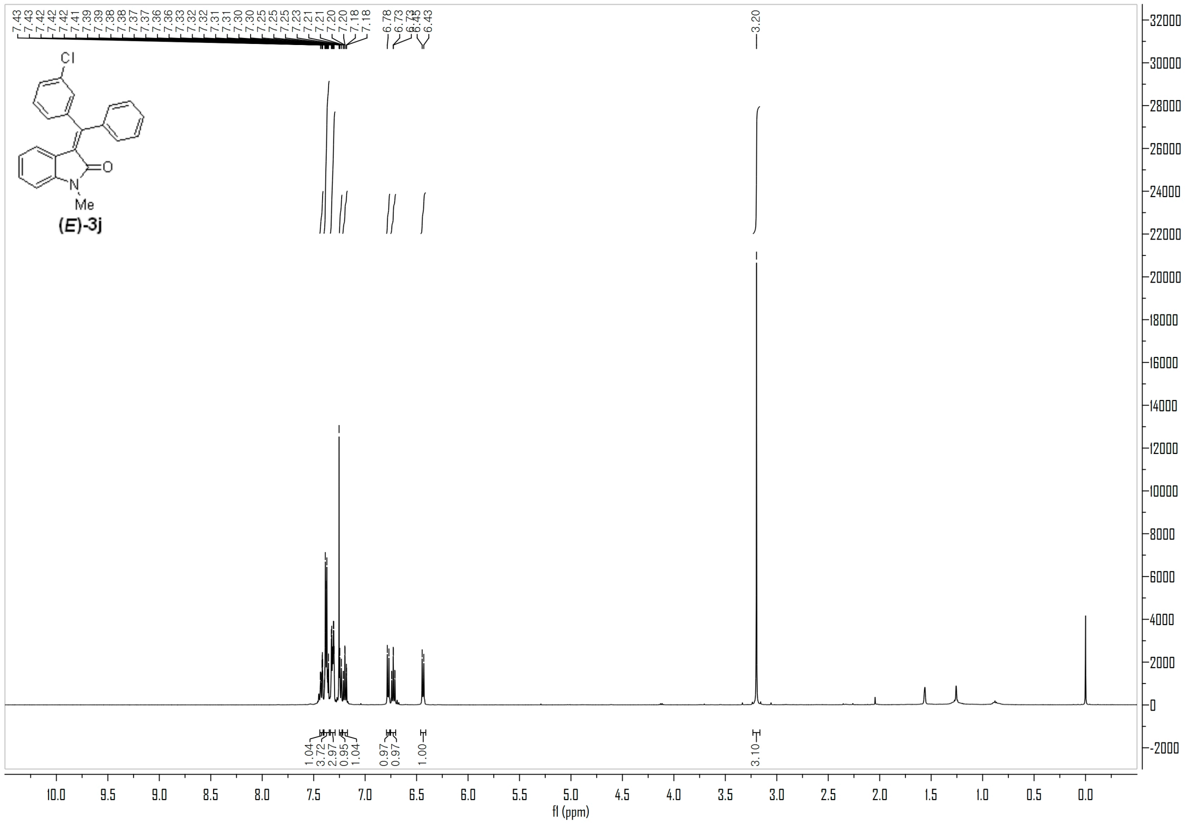

^{13}C NMR spectrum of (*E*)-3j

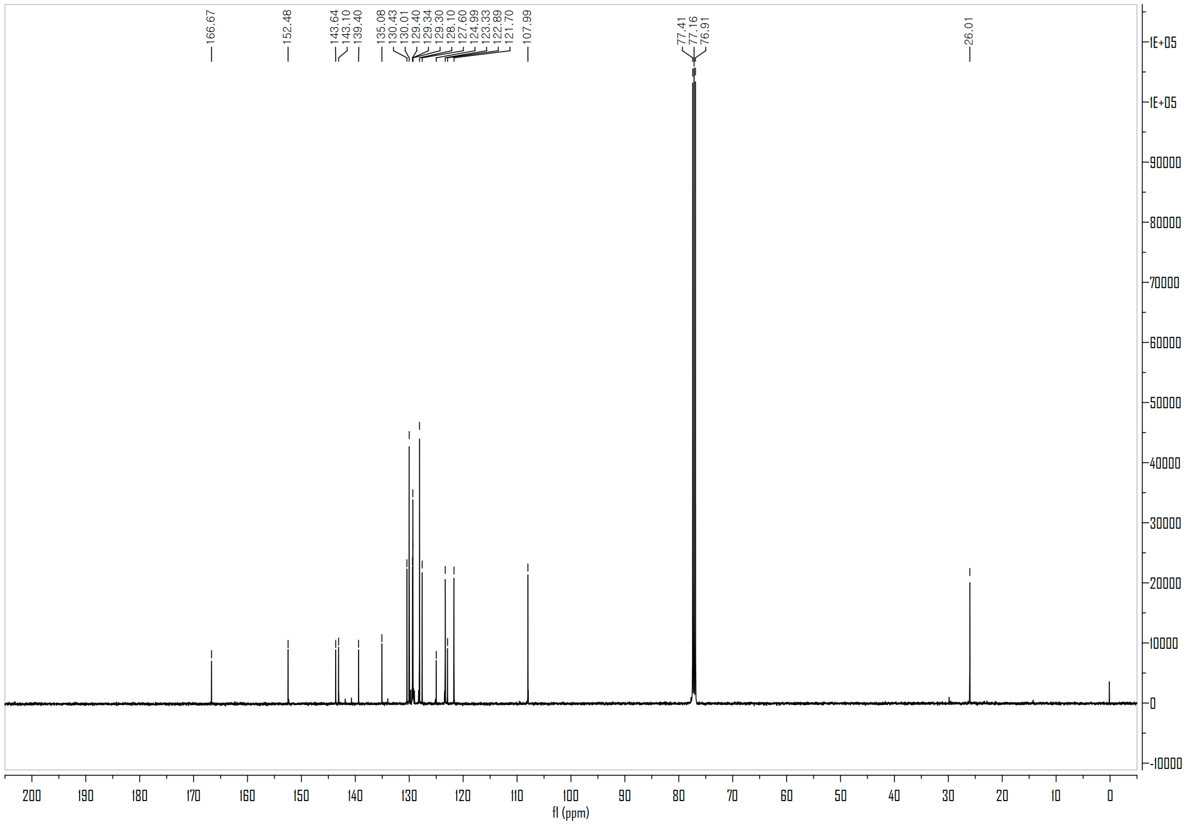

HSQC spectrum of (*E*)-3j

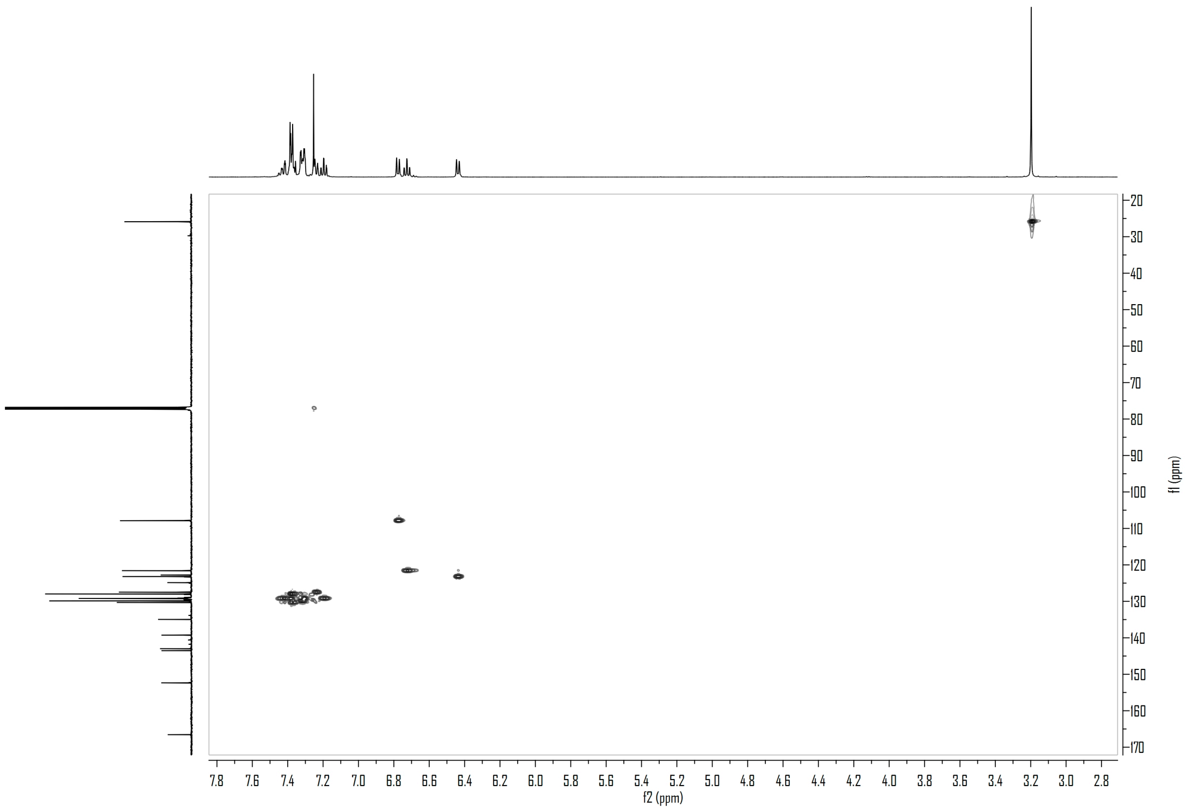

HMBC spectrum of (*E*)-3j

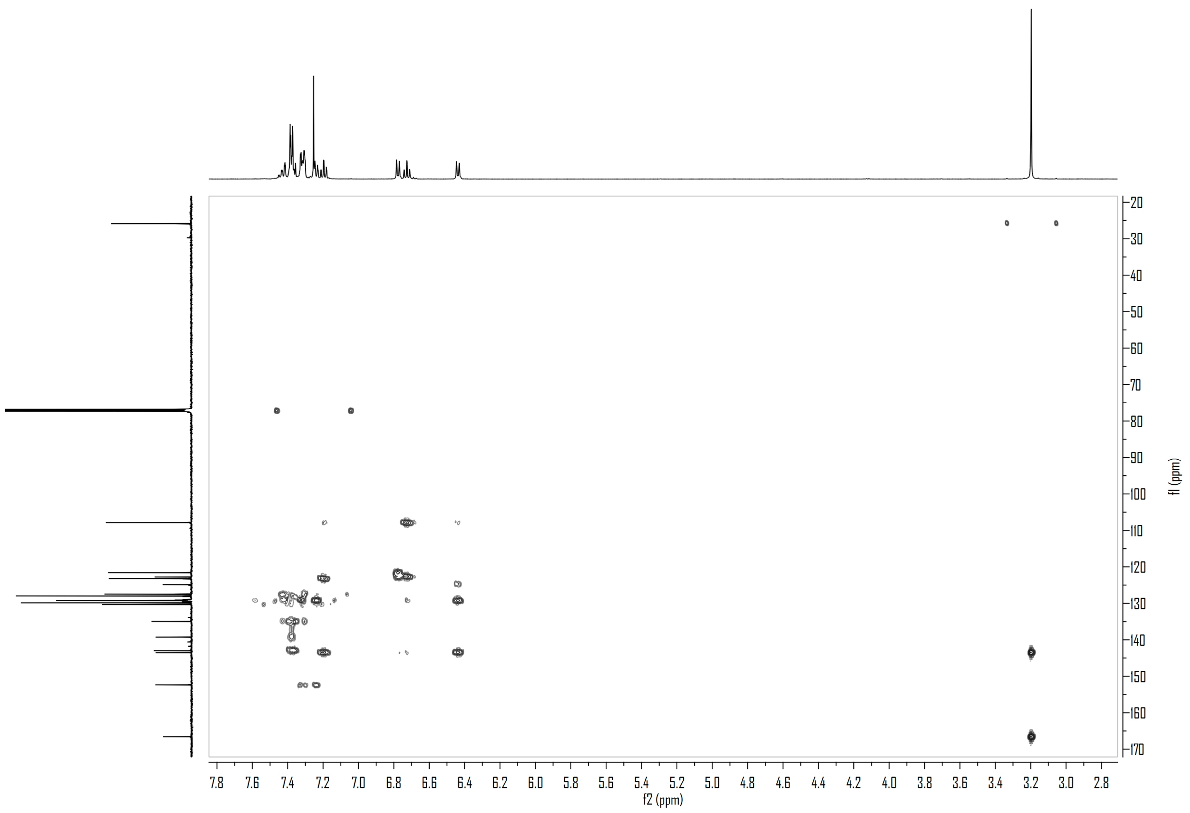

COSY spectrum of (*E*)-3j

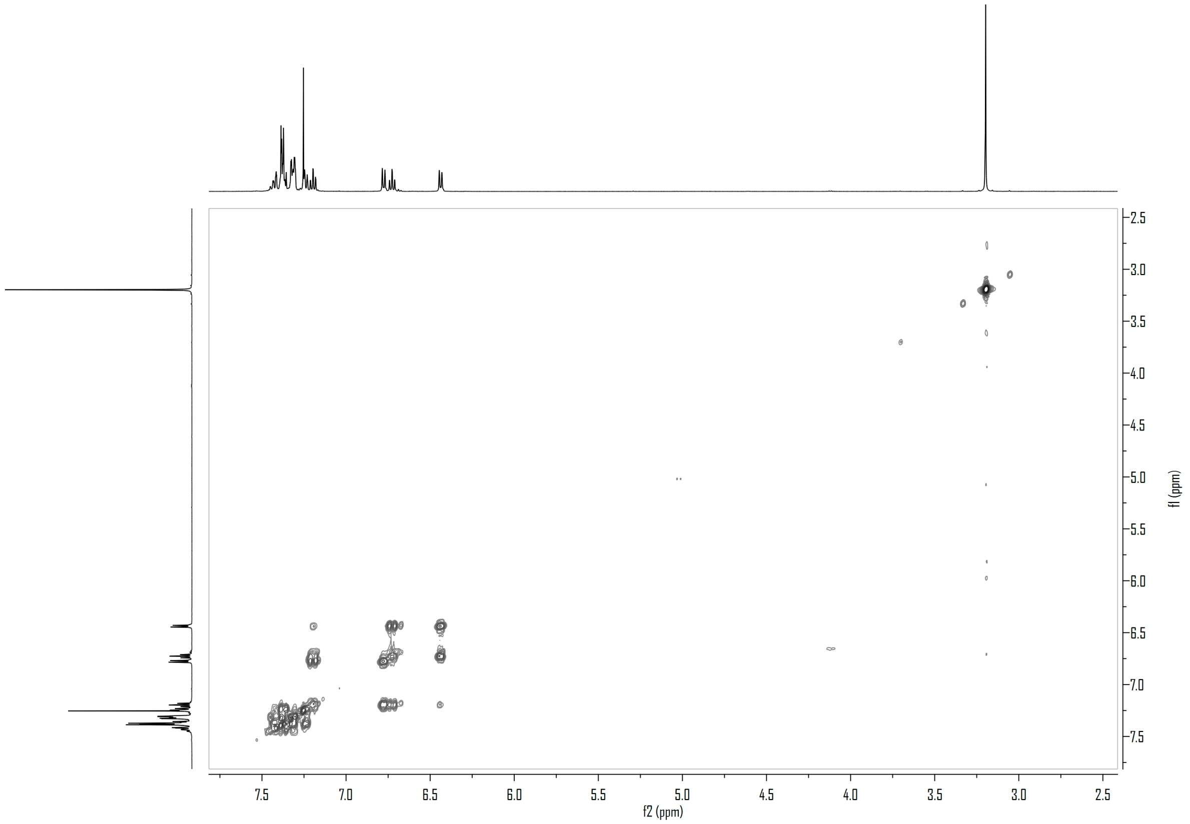

ROESY spectrum of (*E*)-3j

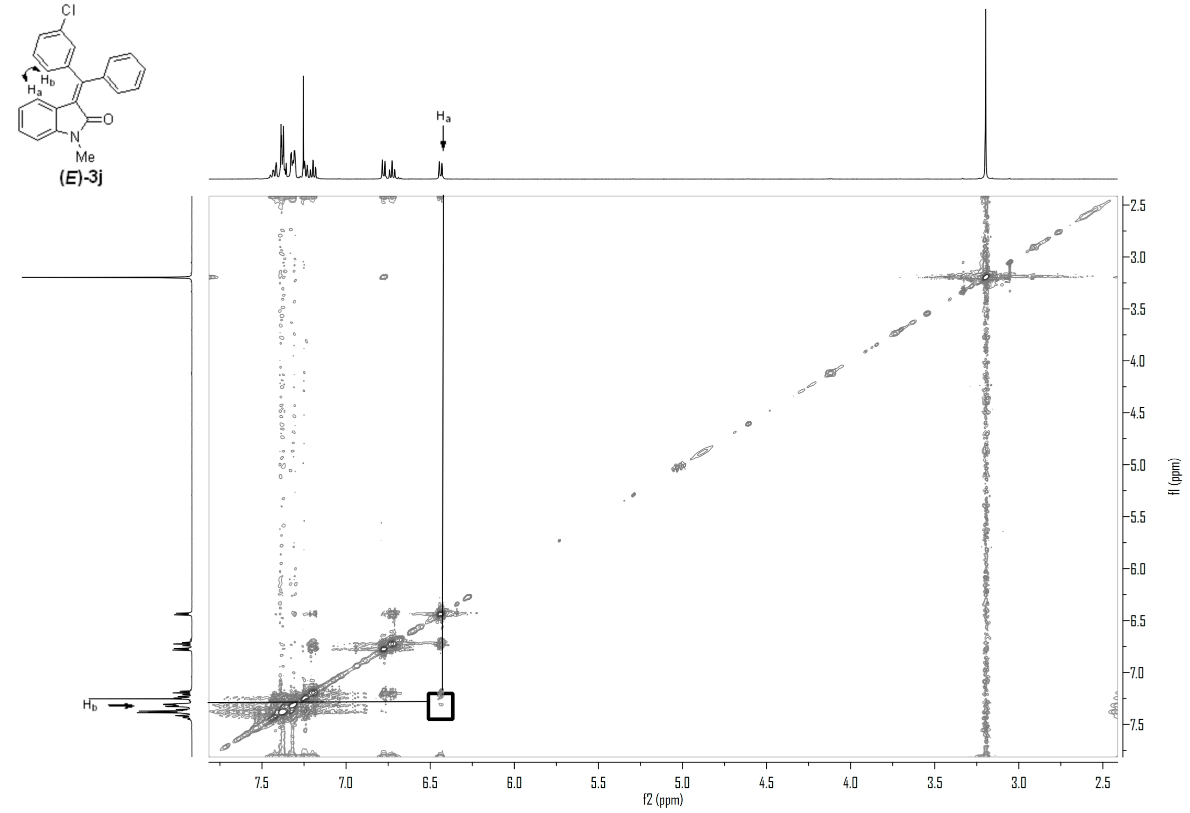

(Z)-1-methyl-3-((3-nitrophenyl)(phenyl)methylene)indolin-2-one ((Z)-3k)

^{1}H NMR spectrum of (Z)-3k

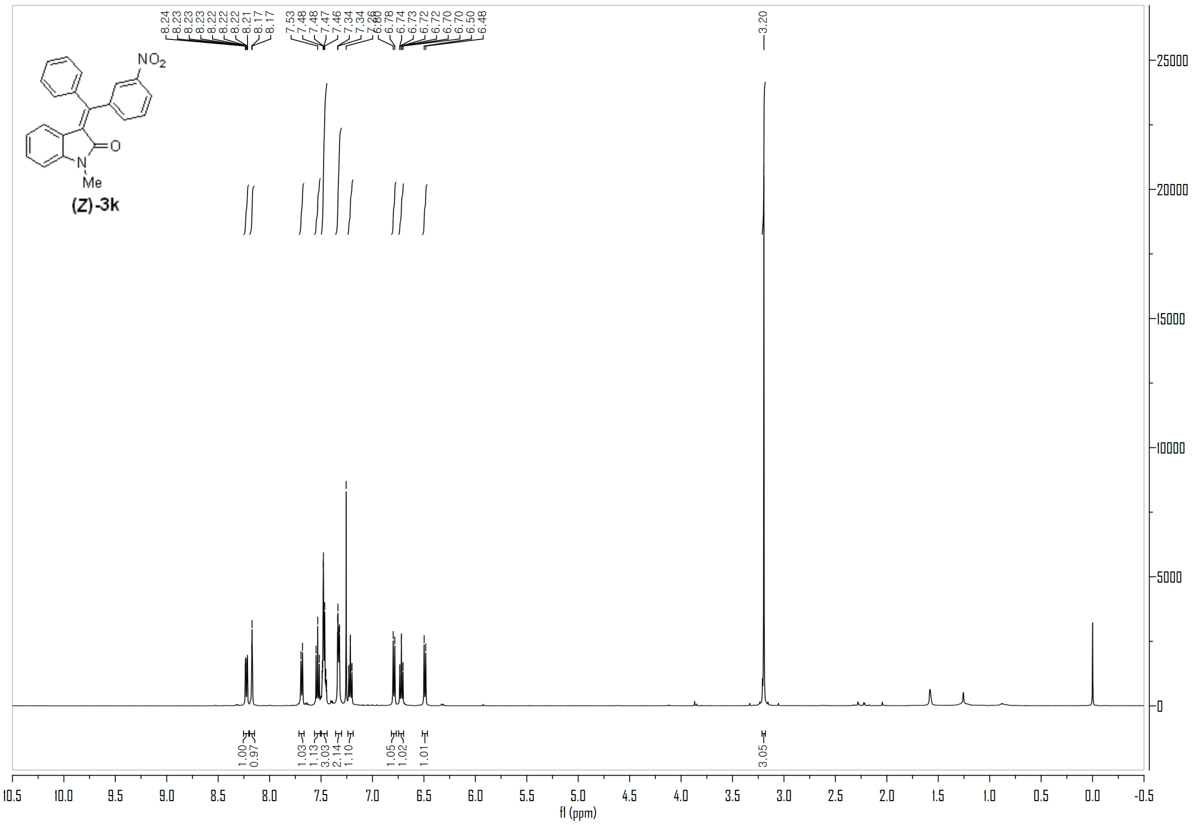

^{13}C NMR spectrum of (Z)-3k

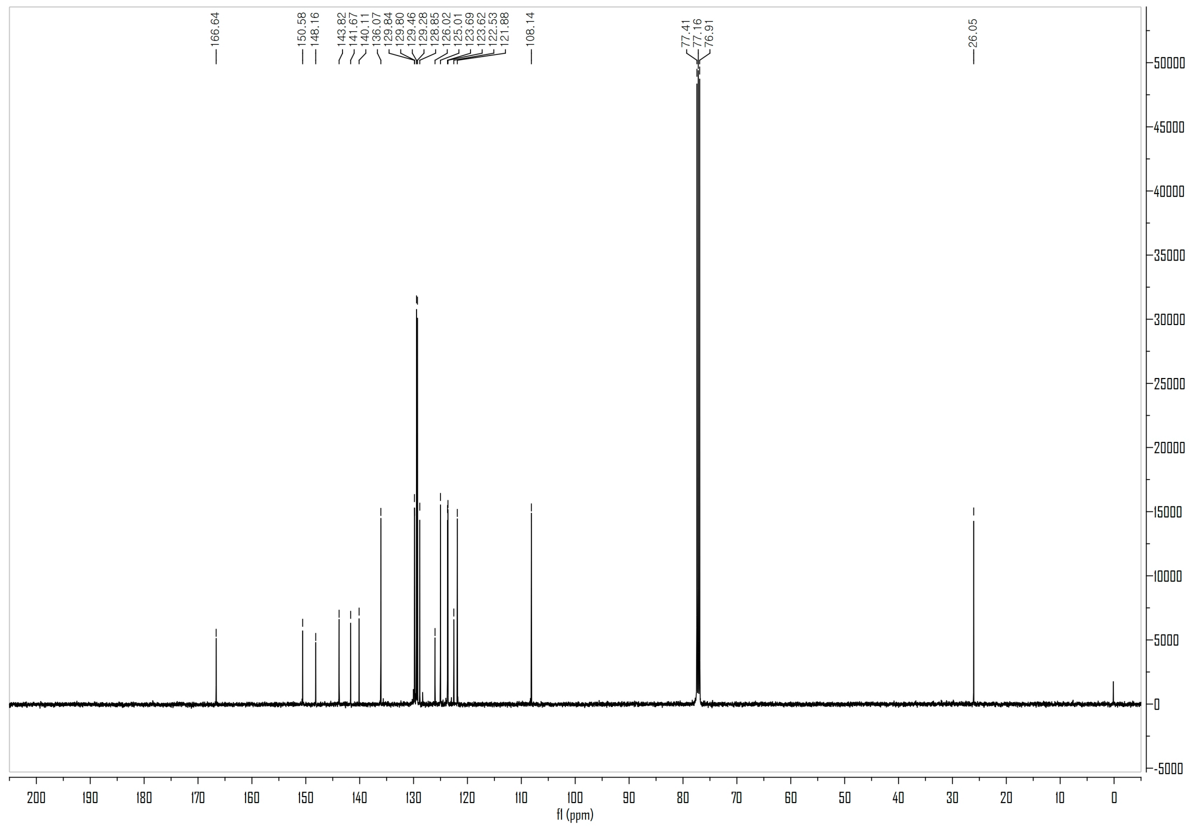

HSQC spectrum of (*Z*)-3k

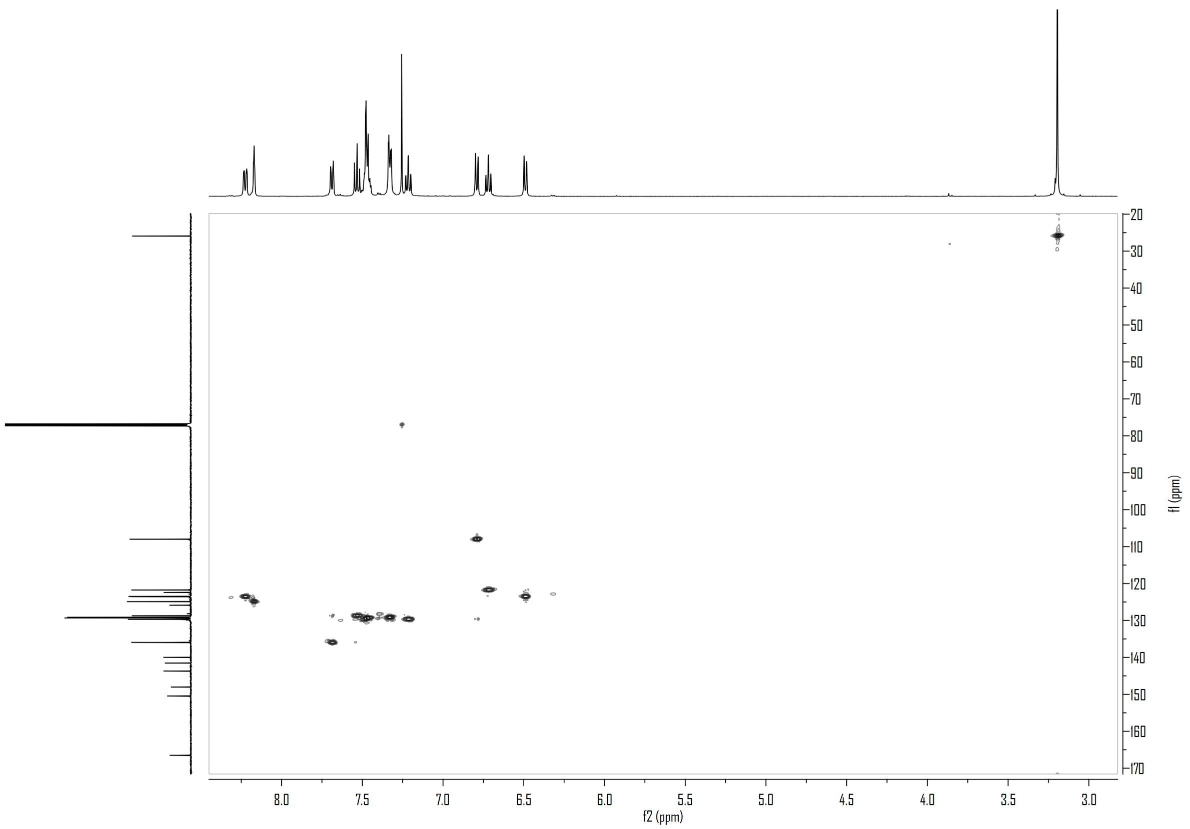

HMBC spectrum of (*Z*)-3k

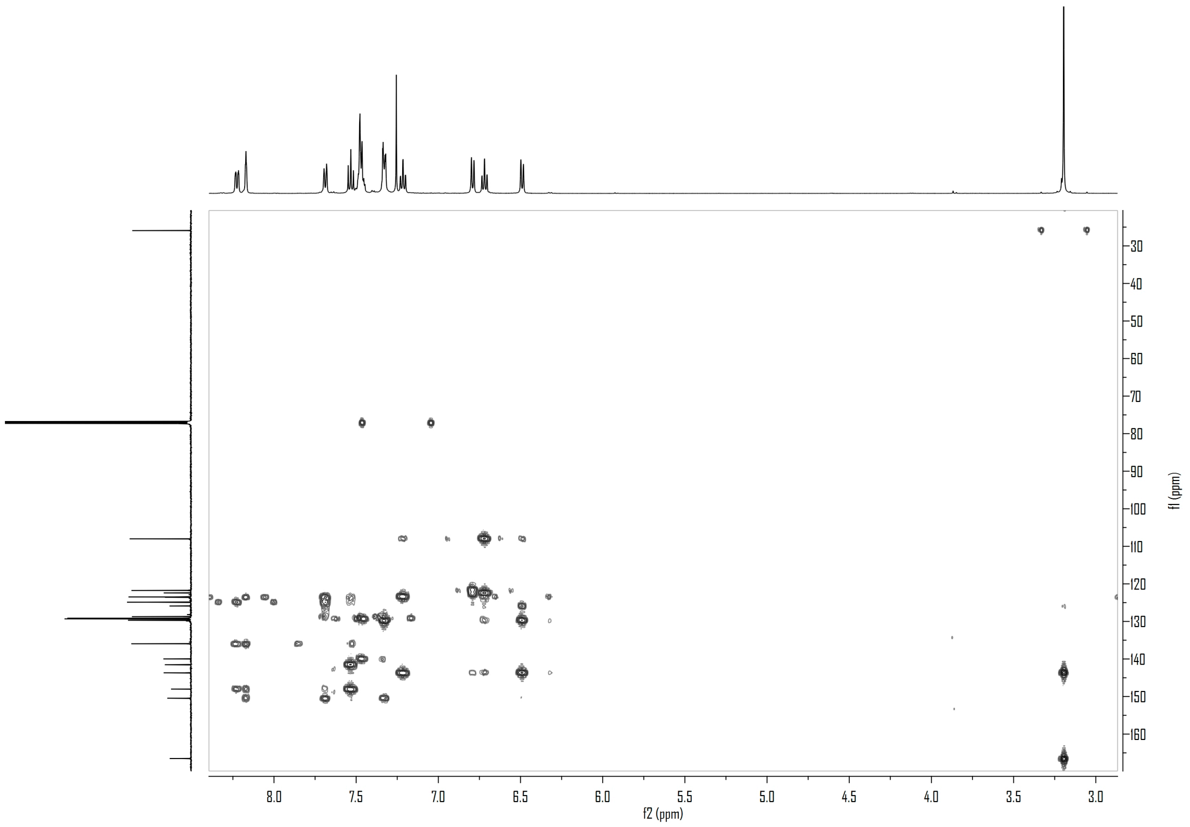

COSY spectrum of (*Z*)-3k

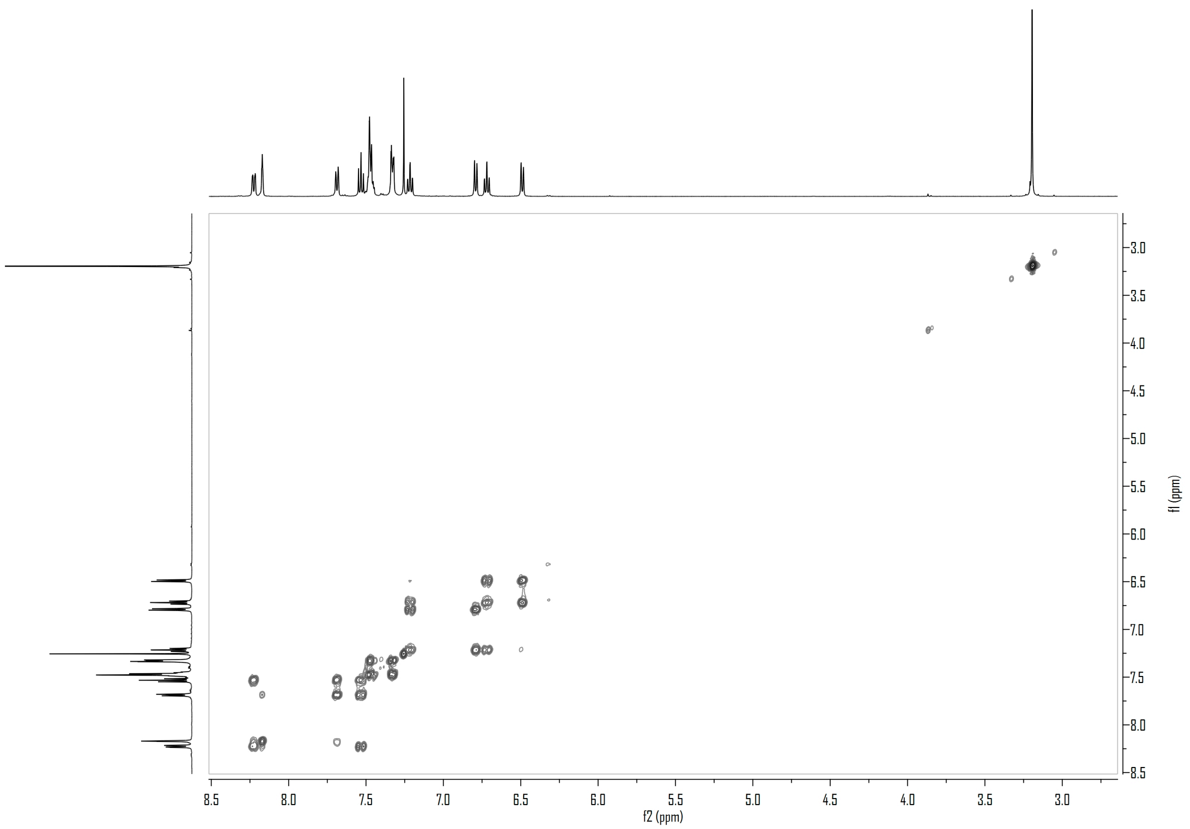

ROESY spectrum of (*Z*)-3k

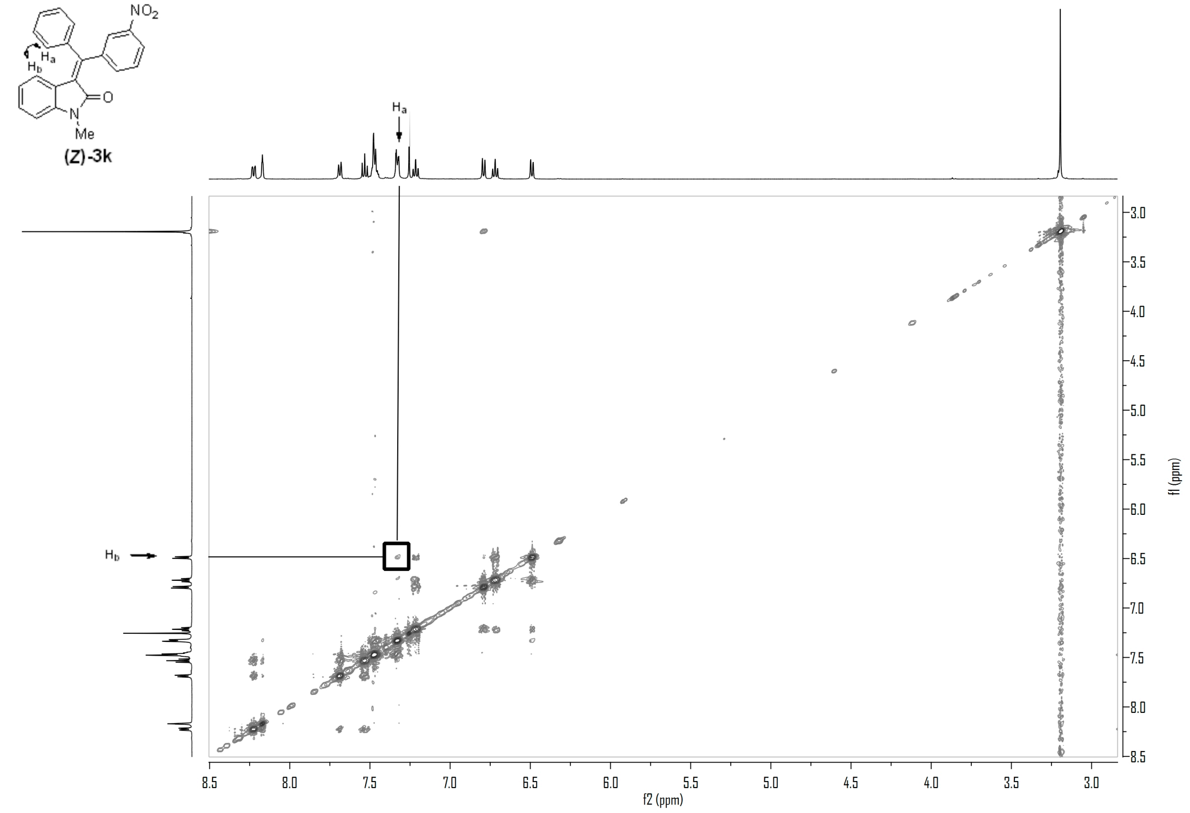

(*E*)-1-methyl-3-((3-nitrophenyl)(phenyl)methylene)indolin-2-one ((*E*)-3k)

^{1}H NMR spectrum of (*E*)-3k

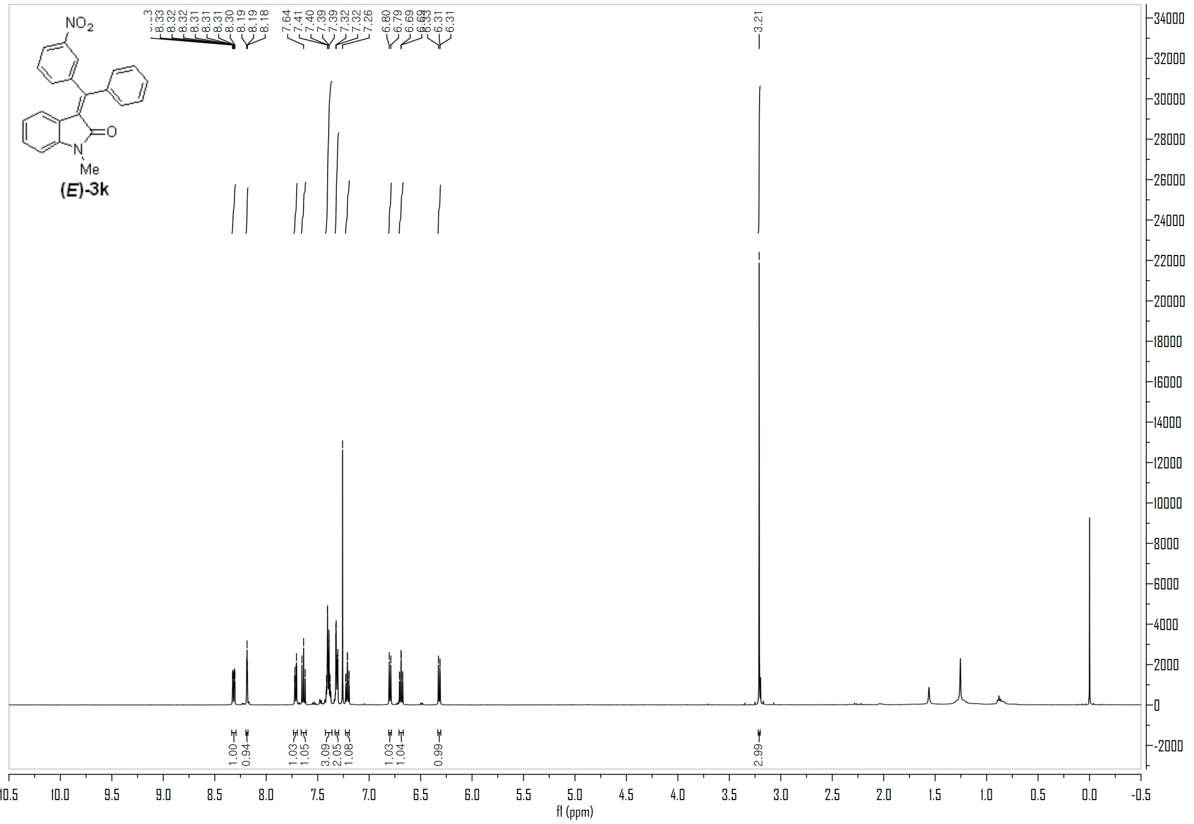

^{13}C NMR spectrum of (*E*)-3k

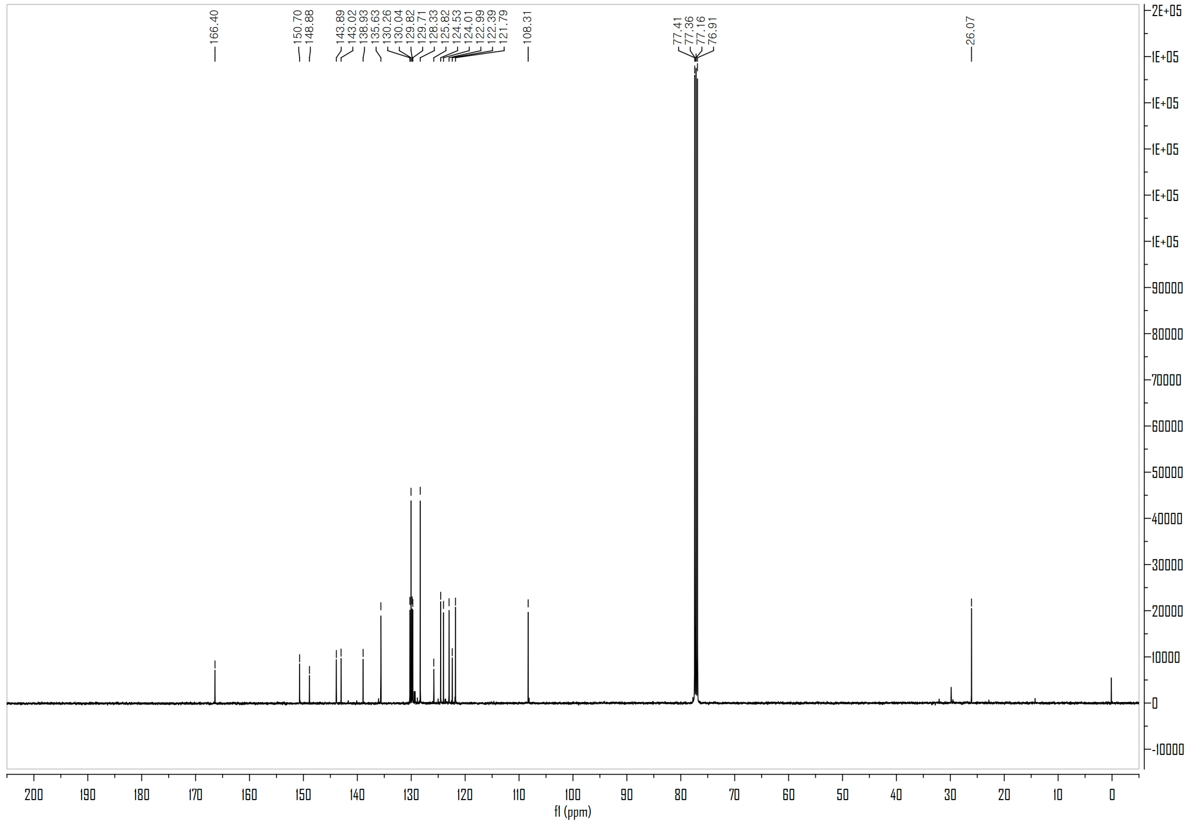

HSQC spectrum of (*E*)-3k

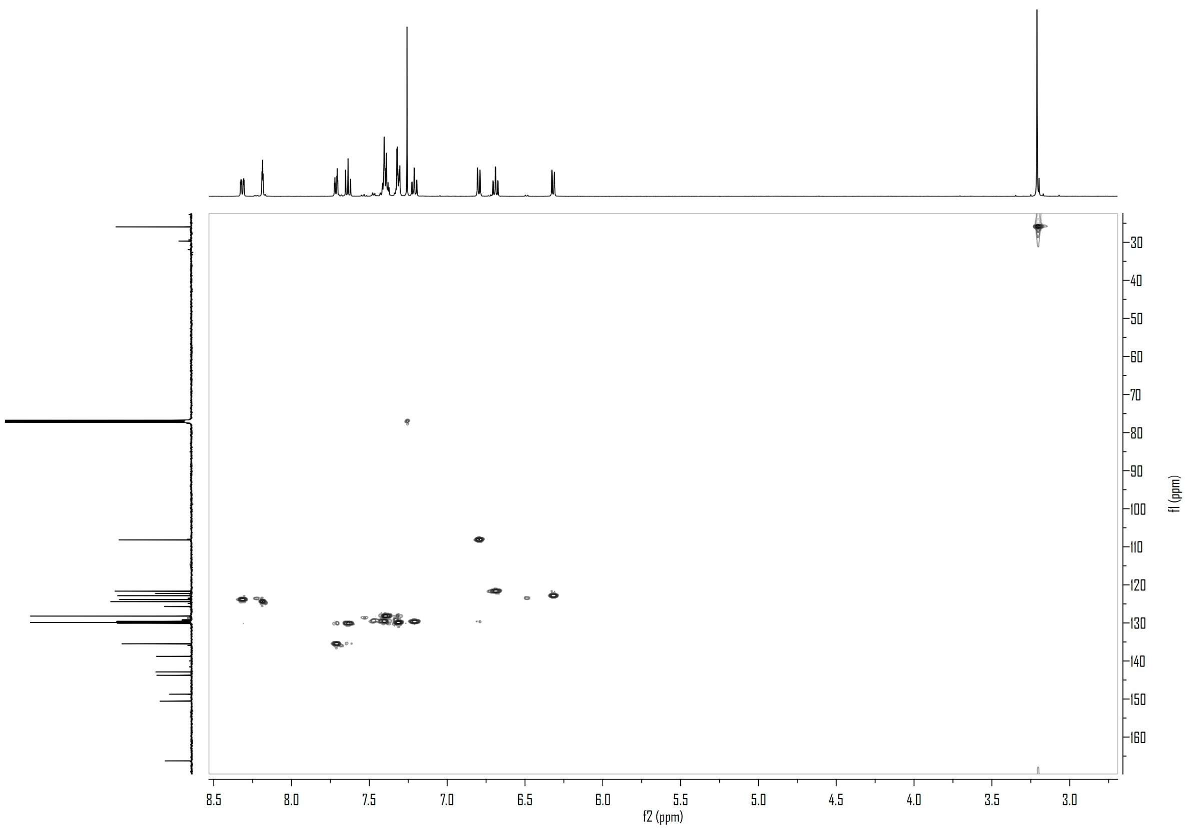

HMBC spectrum of (*E*)-3k

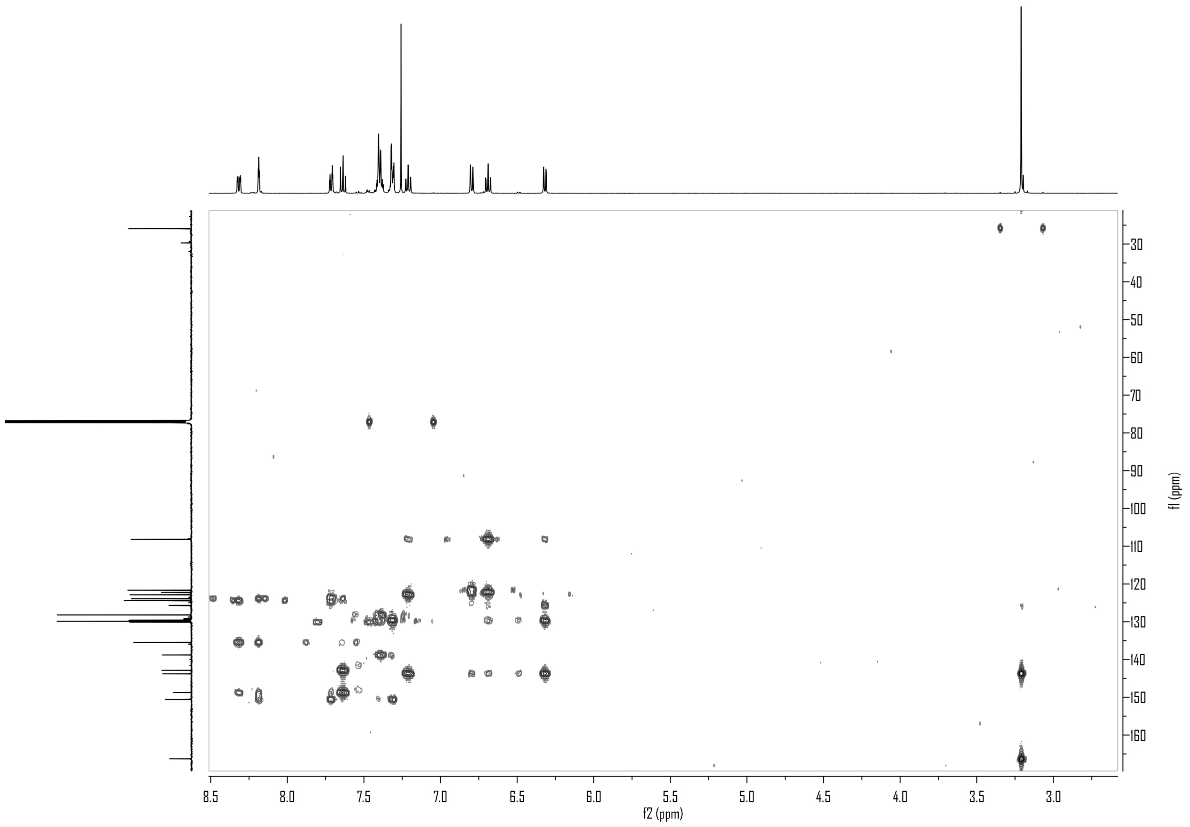

COSY spectrum of (*E*)-3k

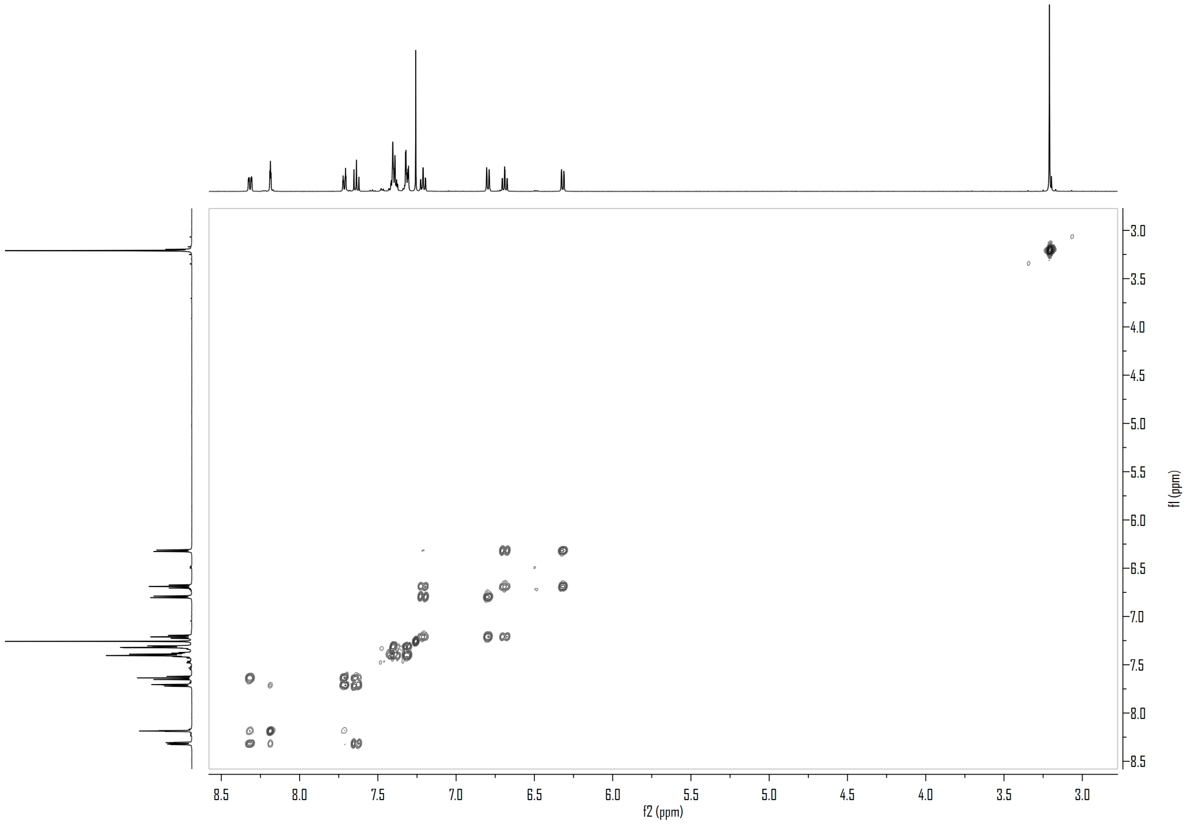

ROESY spectrum of (*E*)-3k

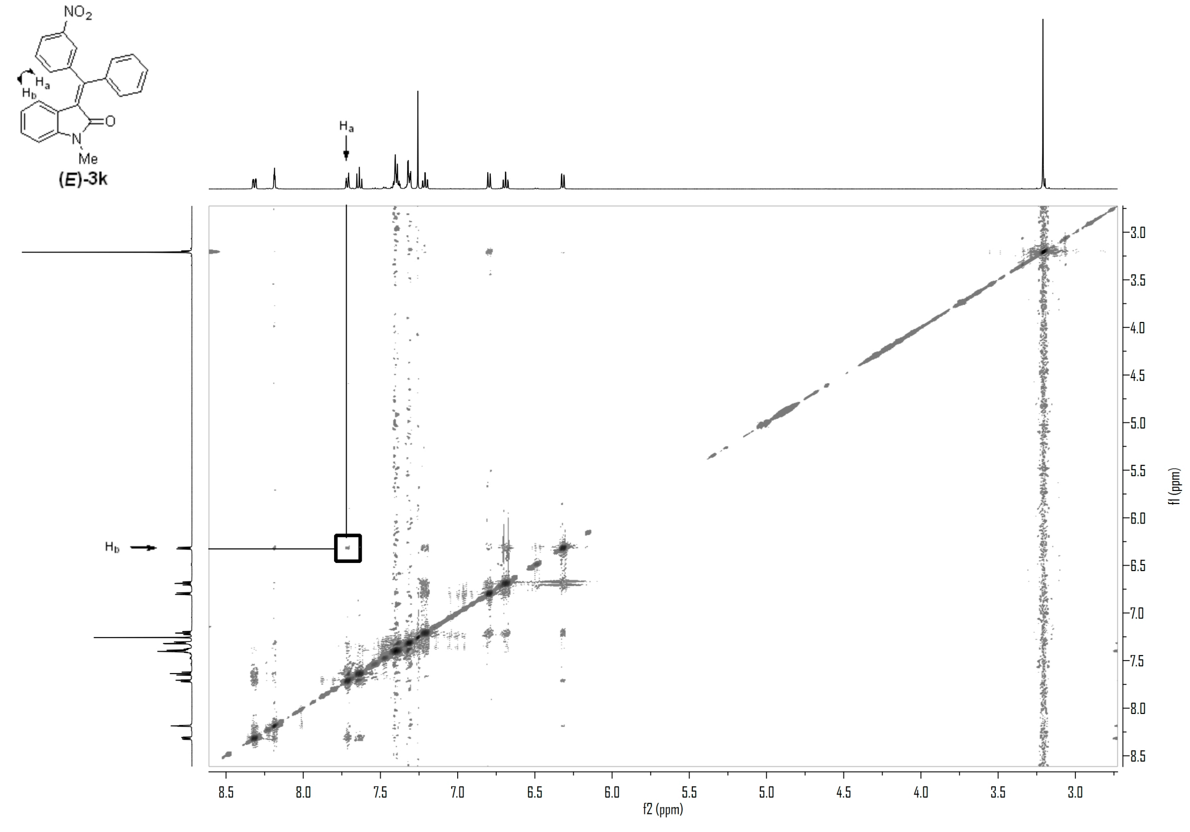

(*Z*)-3-((2-methoxyphenyl)(phenyl)methylene)-1-methylindolin-2-one ((*Z*)-3l)

^{1}H NMR spectrum of (*Z*)-3l

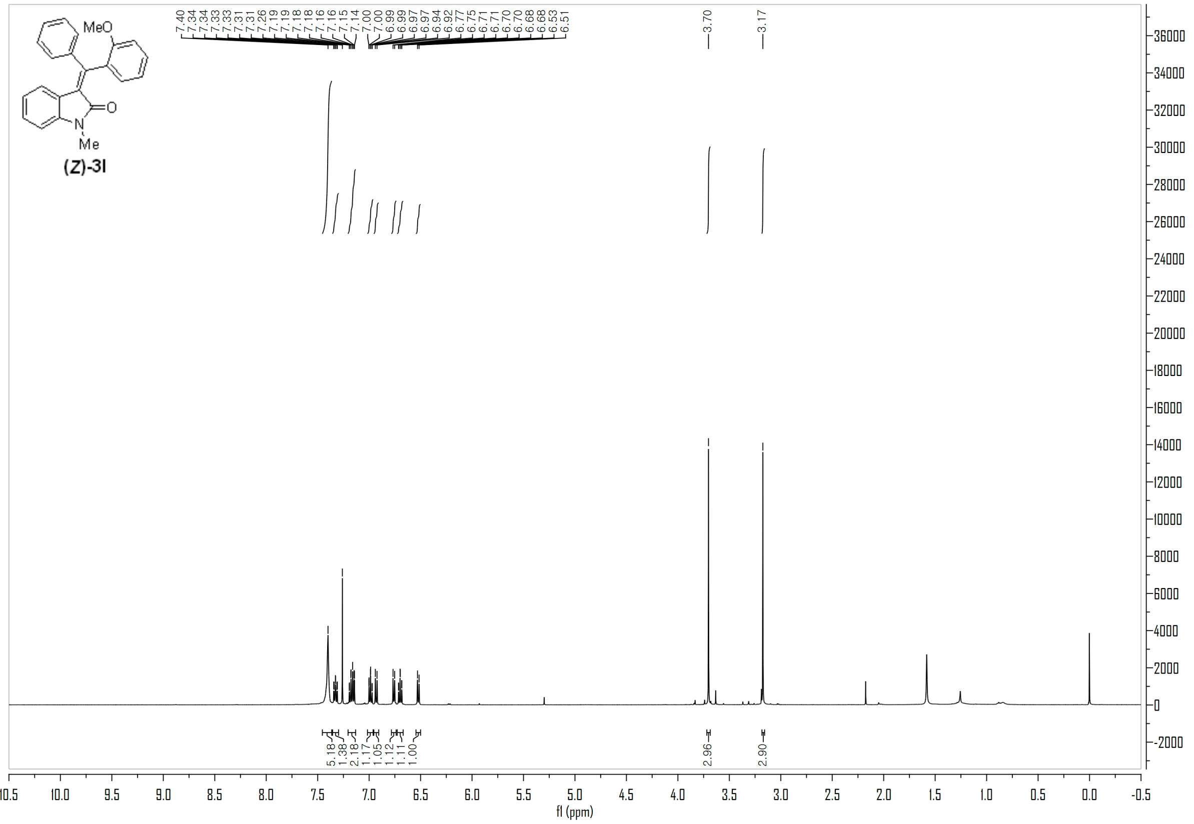

^{13}C NMR spectrum of (*Z*)-3l

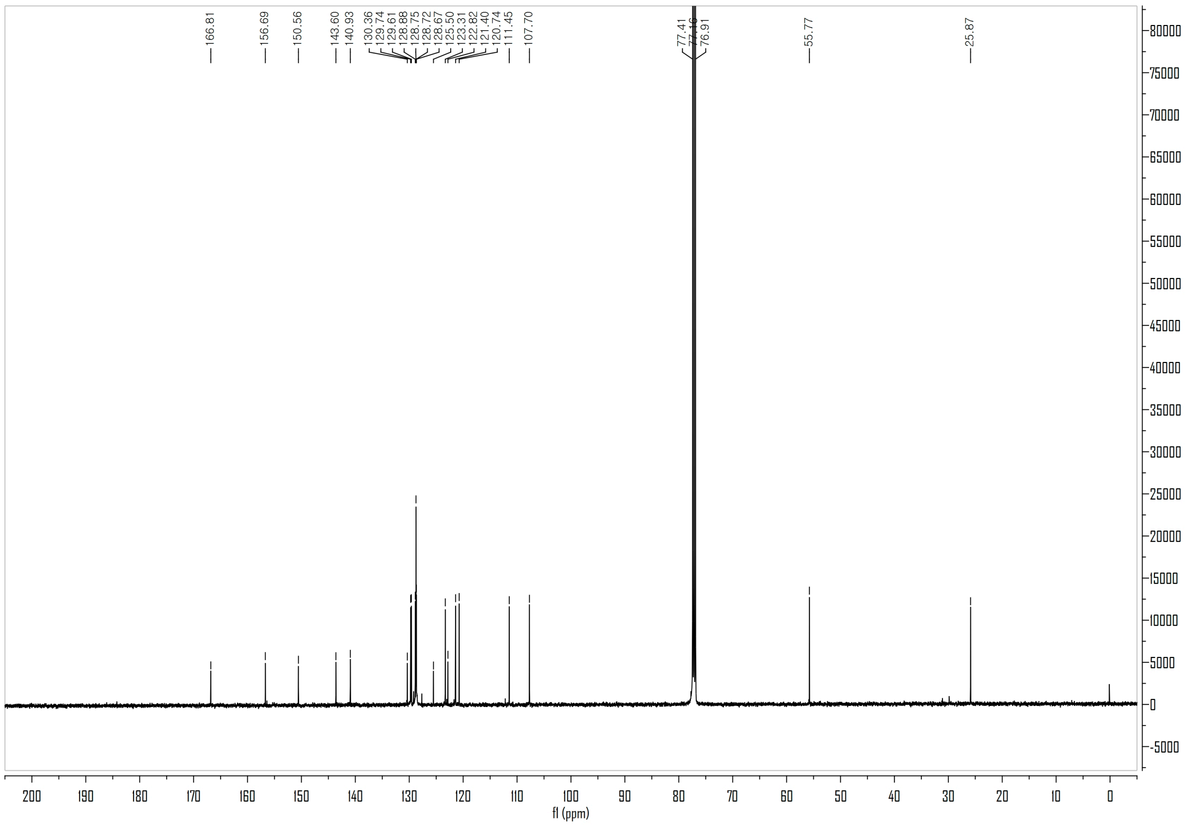

HSQC spectrum of (*Z*)-3l

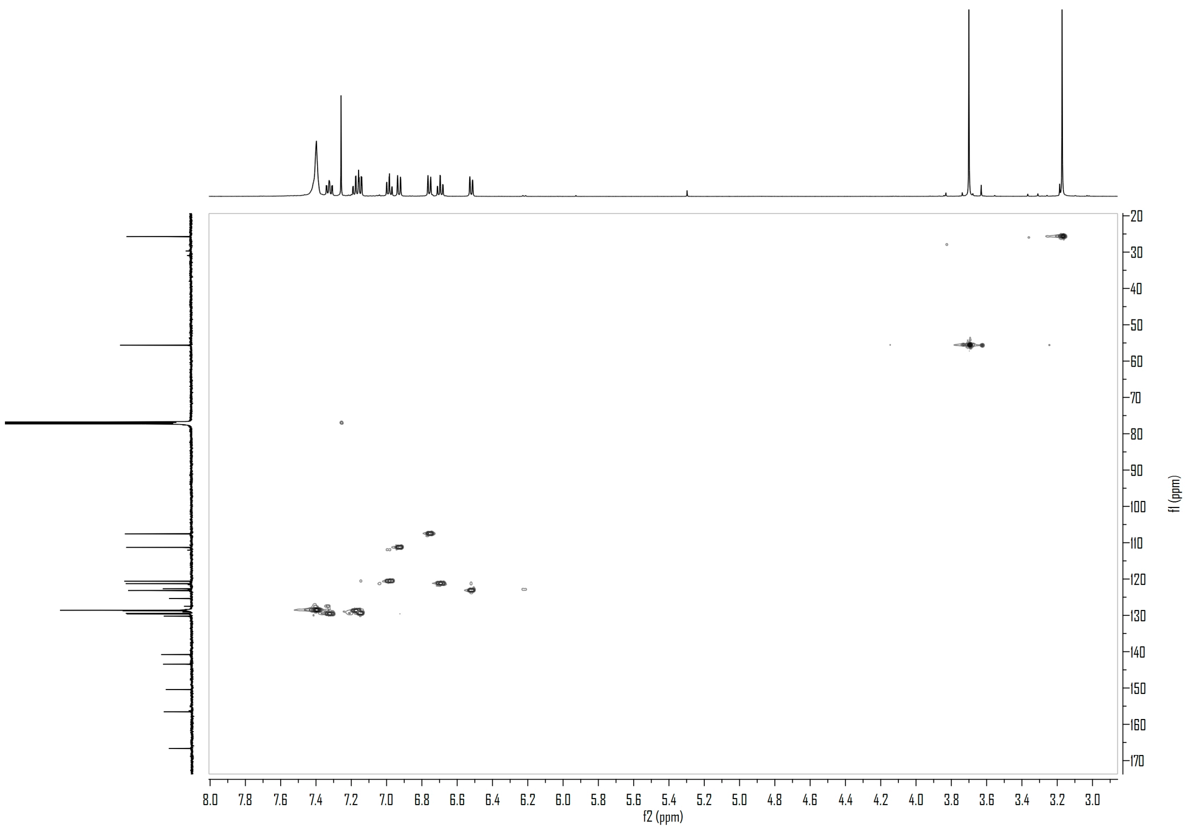

HMBC spectrum of (*Z*)-3l

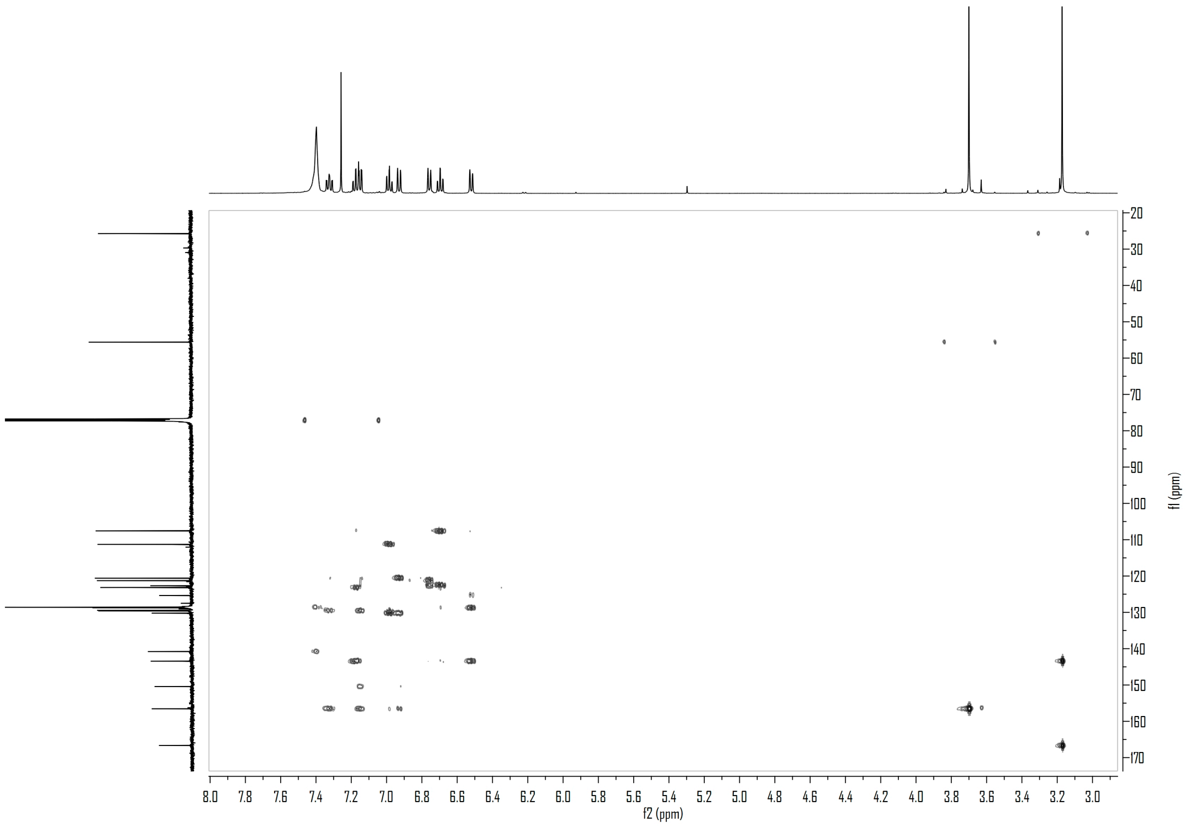

COSY spectrum of (*Z*)-3l

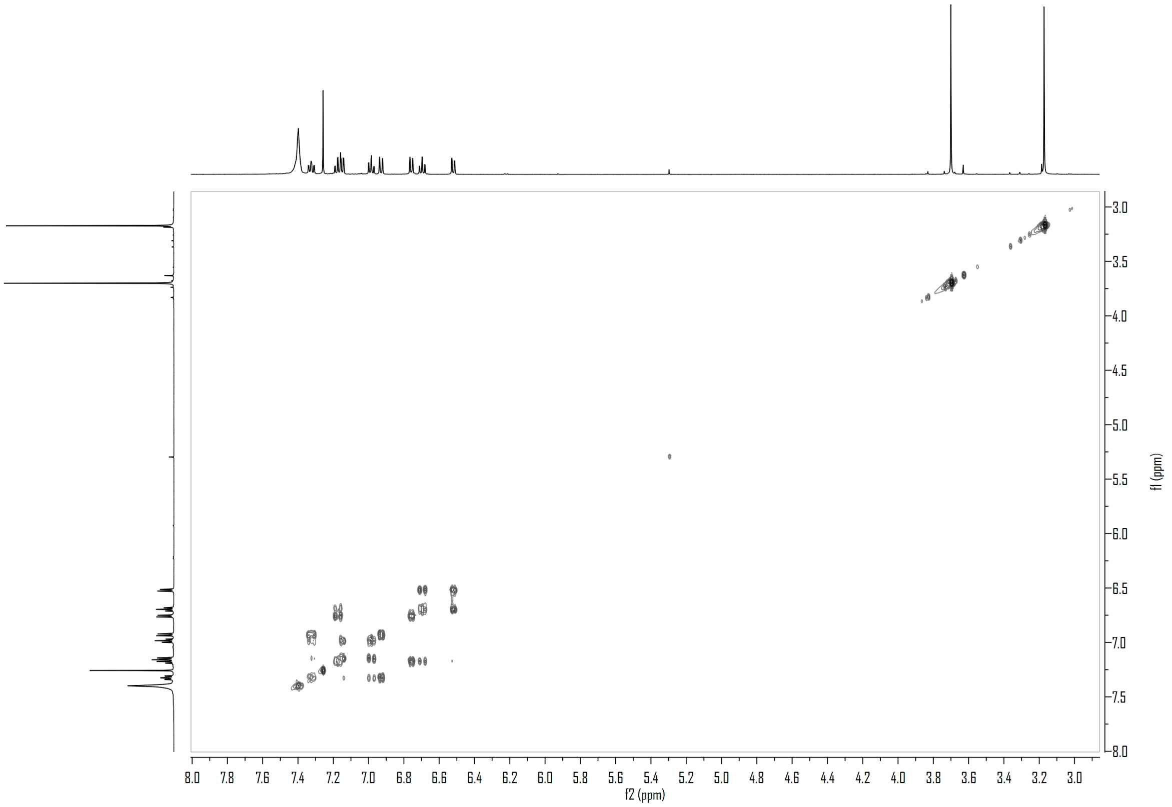

ROESY spectrum of (*Z*)-3l

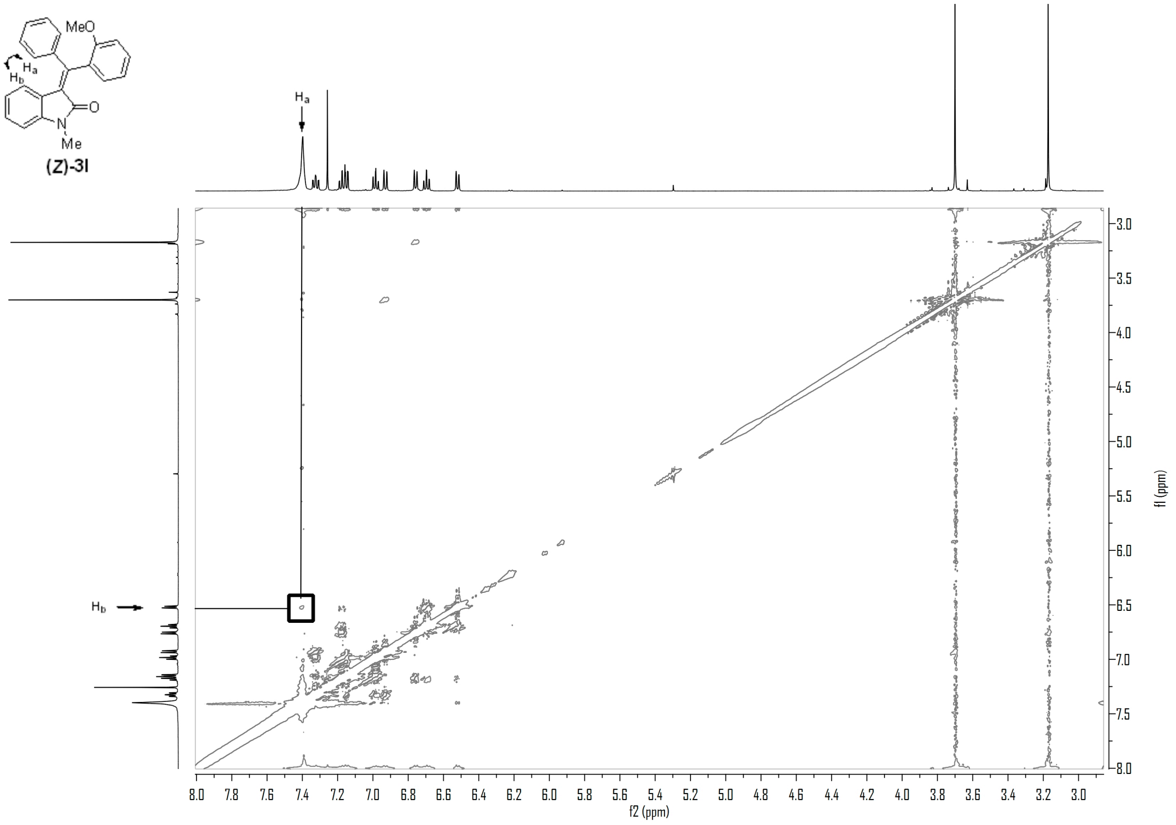

(E)-3-((2-methoxyphenyl)(phenyl)methylene)-1-methylindolin-2-one ((E)-3l)

^{1}H NMR spectrum of (E)-3l

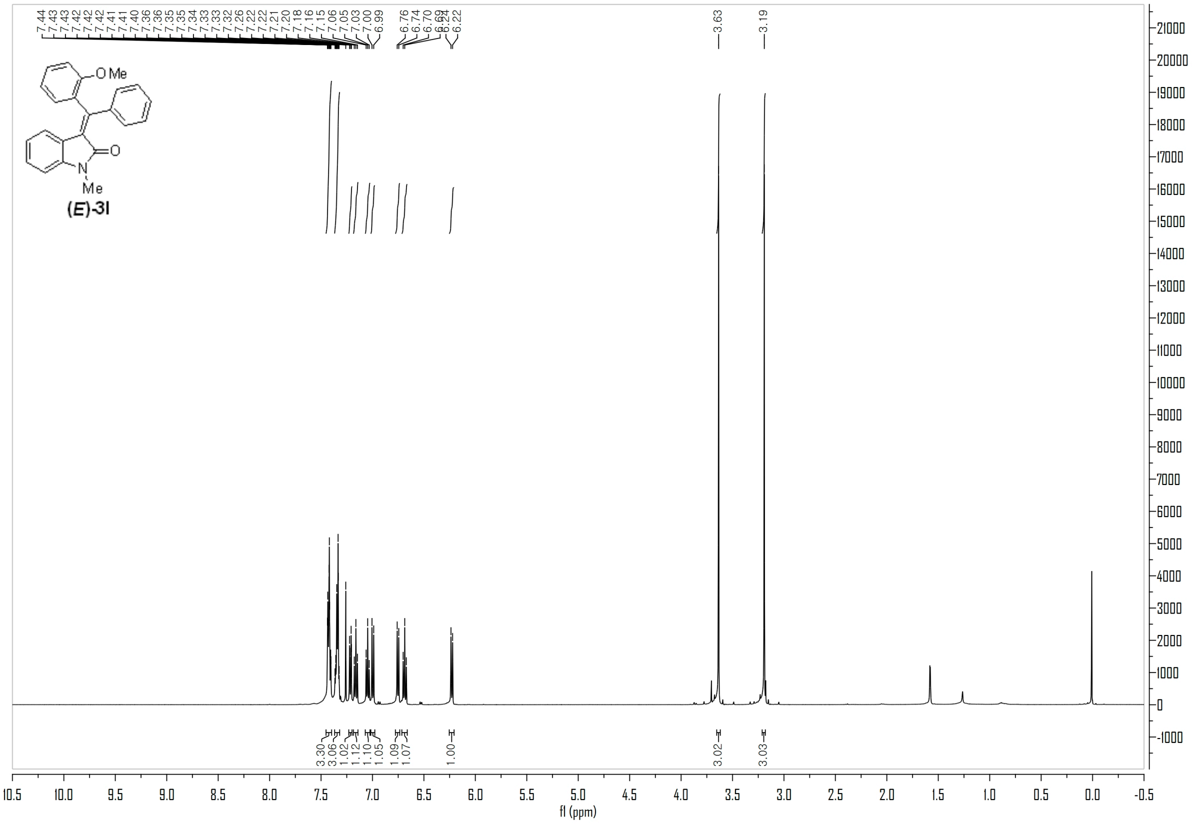

^{13}C NMR spectrum of (E)-3l

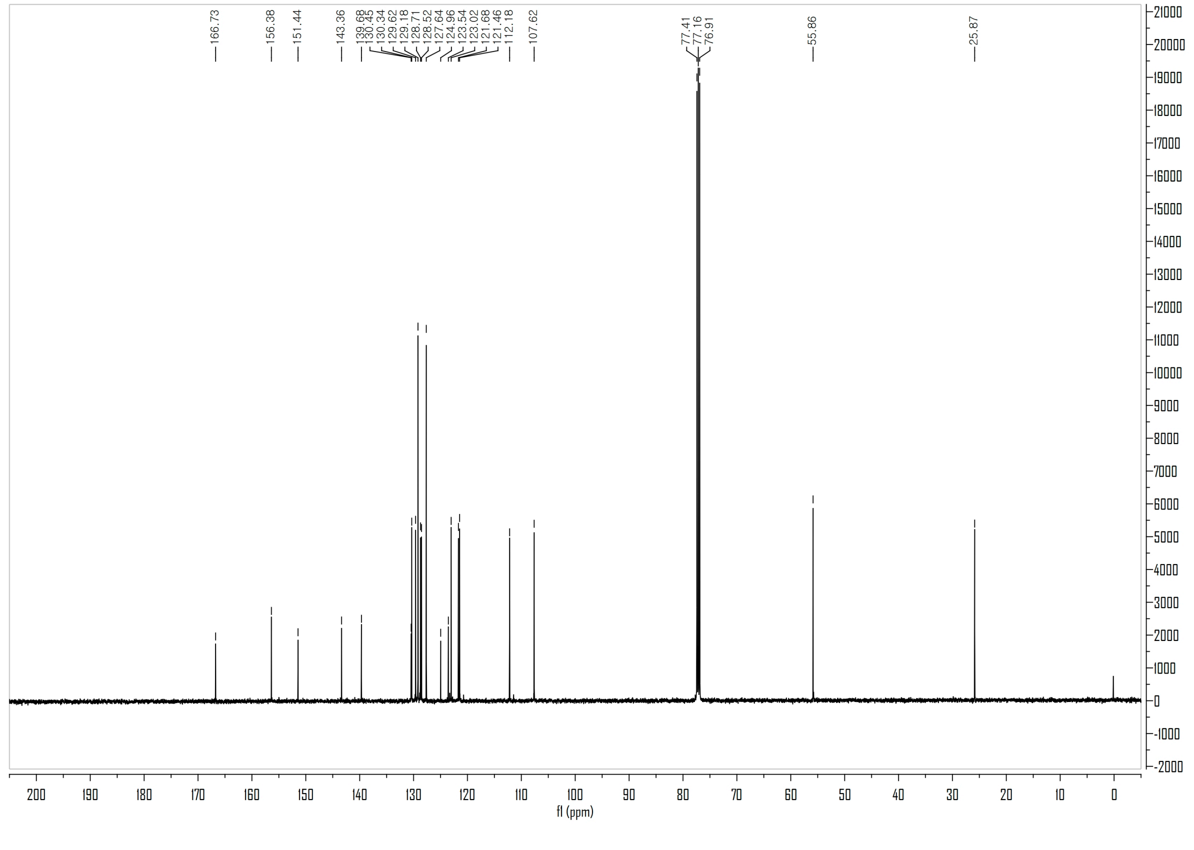

HSQC spectrum of (*E*)-3l

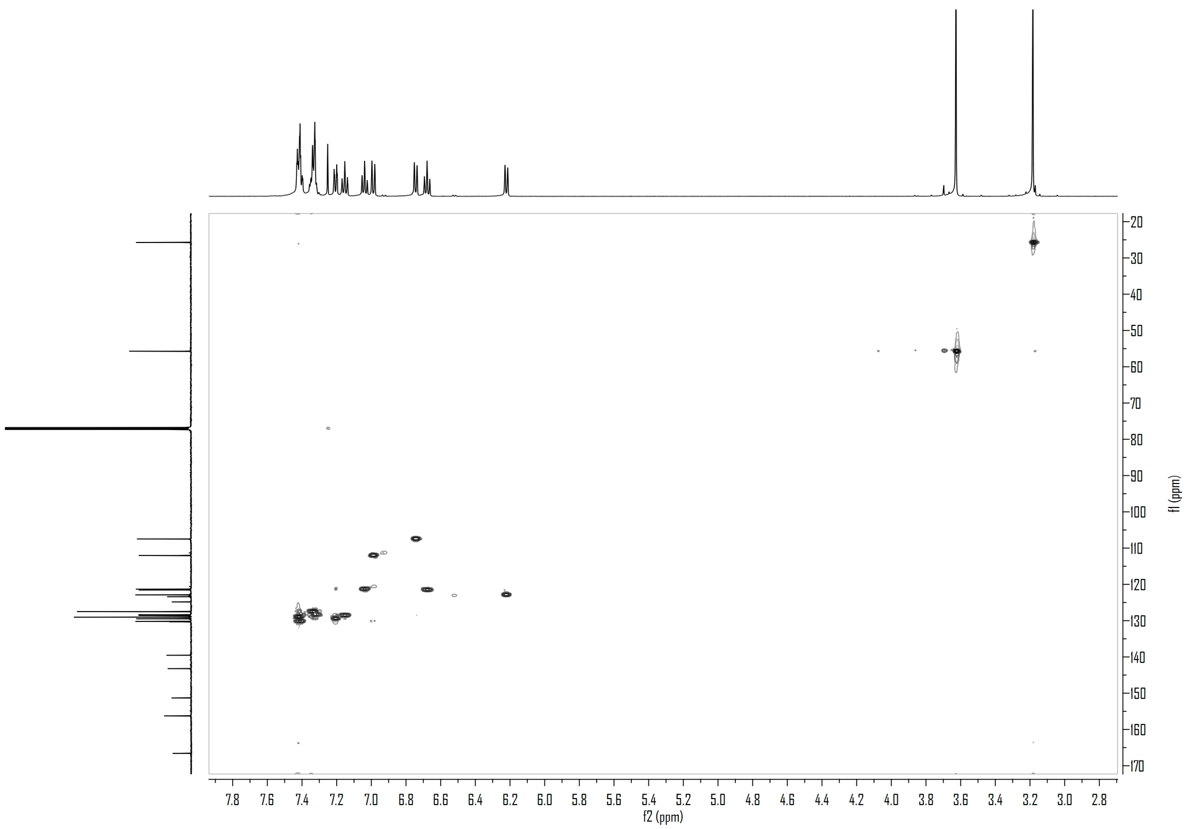

HMBC spectrum of (*E*)-3l

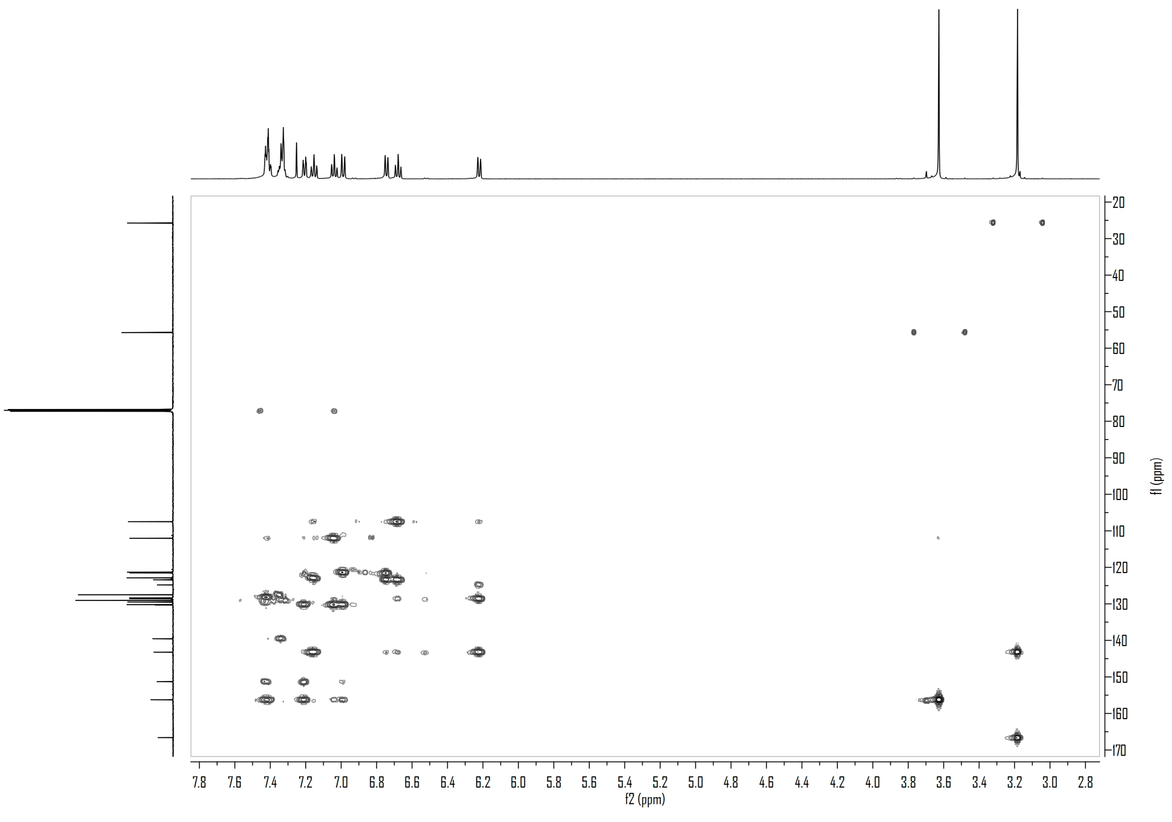

COSY spectrum of (*E*)-3l

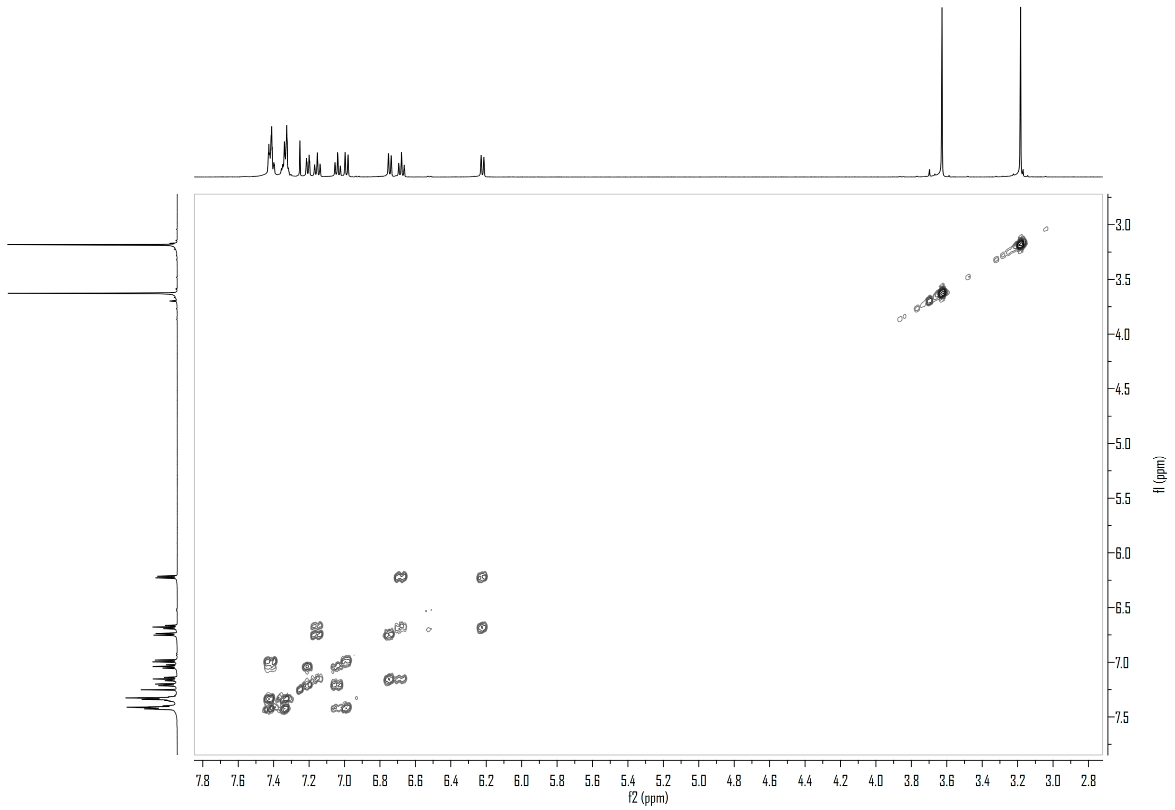

ROESY spectrum of (*E*)-3l

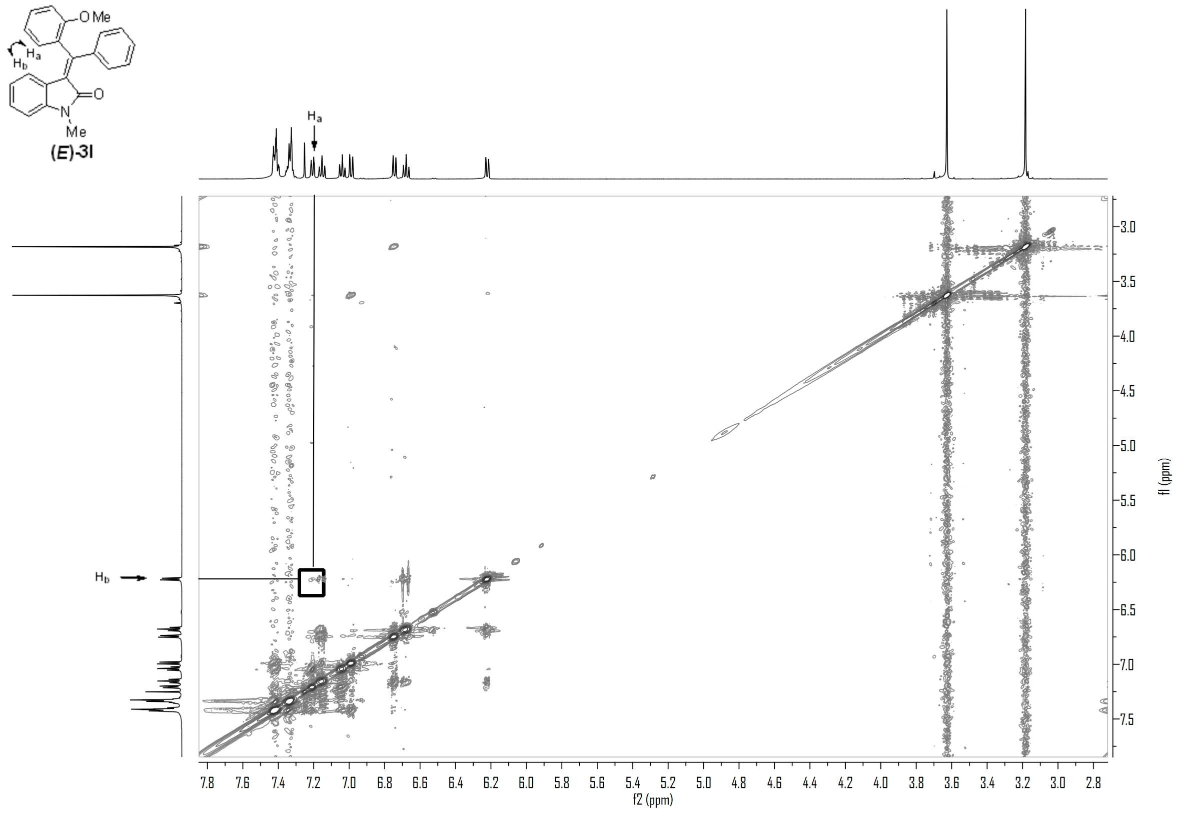

(*Z*)-3-((2-chlorophenyl)(phenyl)methylene)-1-methylindolin-2-one ((*Z*)-3m)

^{1}H NMR spectrum of (*Z*)-3m

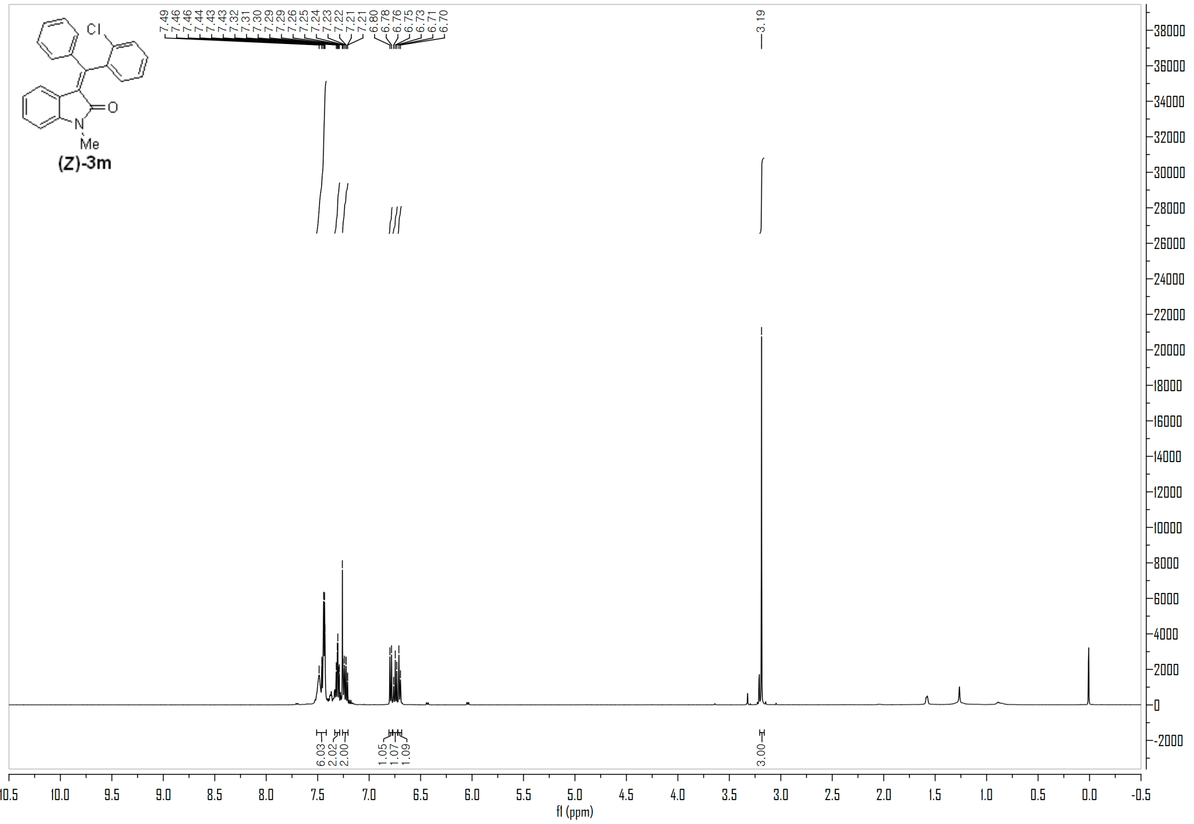

^{13}C NMR spectrum of (*Z*)-3m

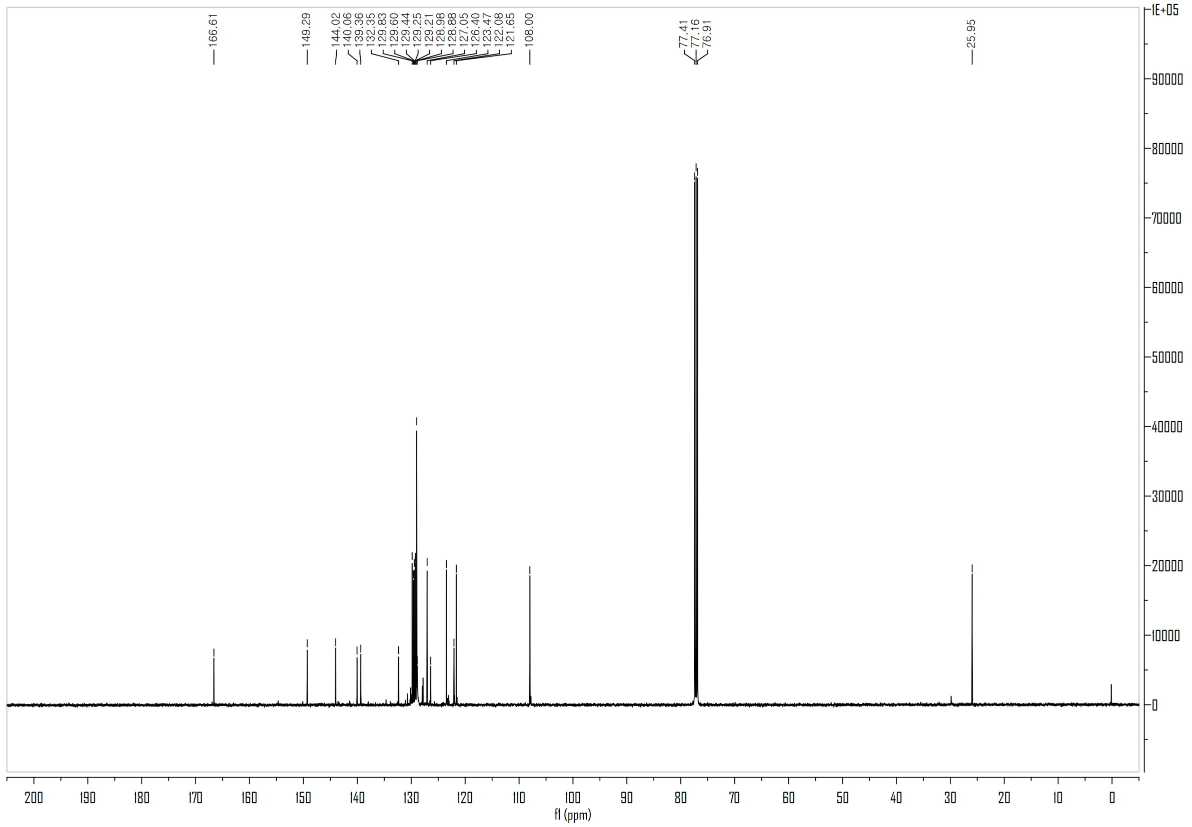

HSQC spectrum of (*Z*)-3m

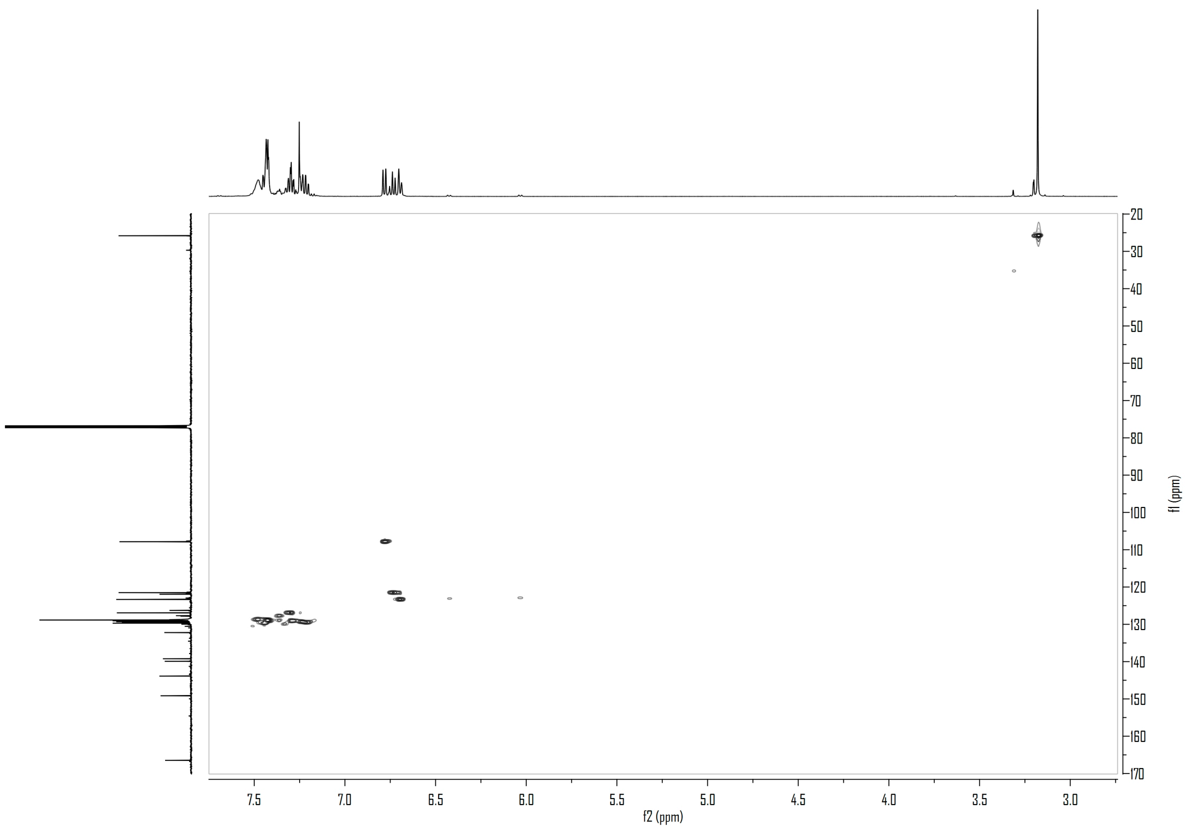

HMBC spectrum of (*Z*)-3m

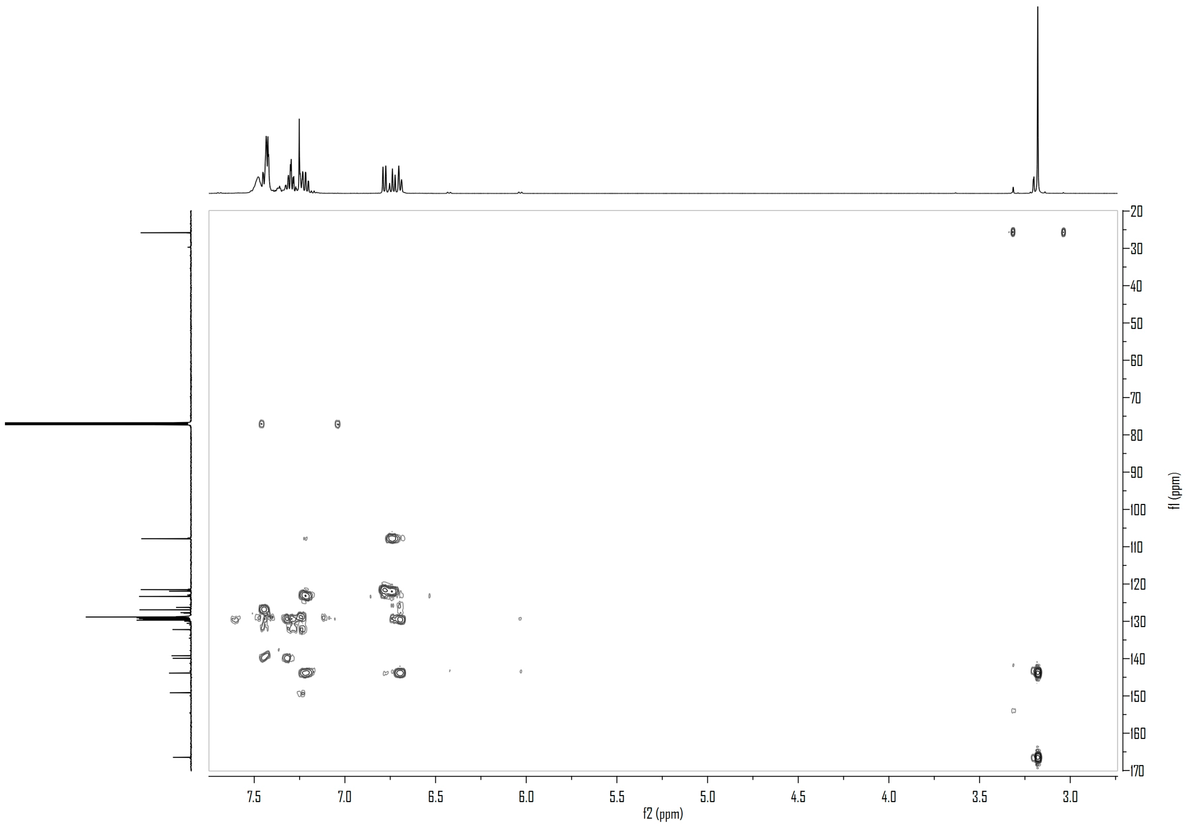

COSY spectrum of (*Z*)-3m

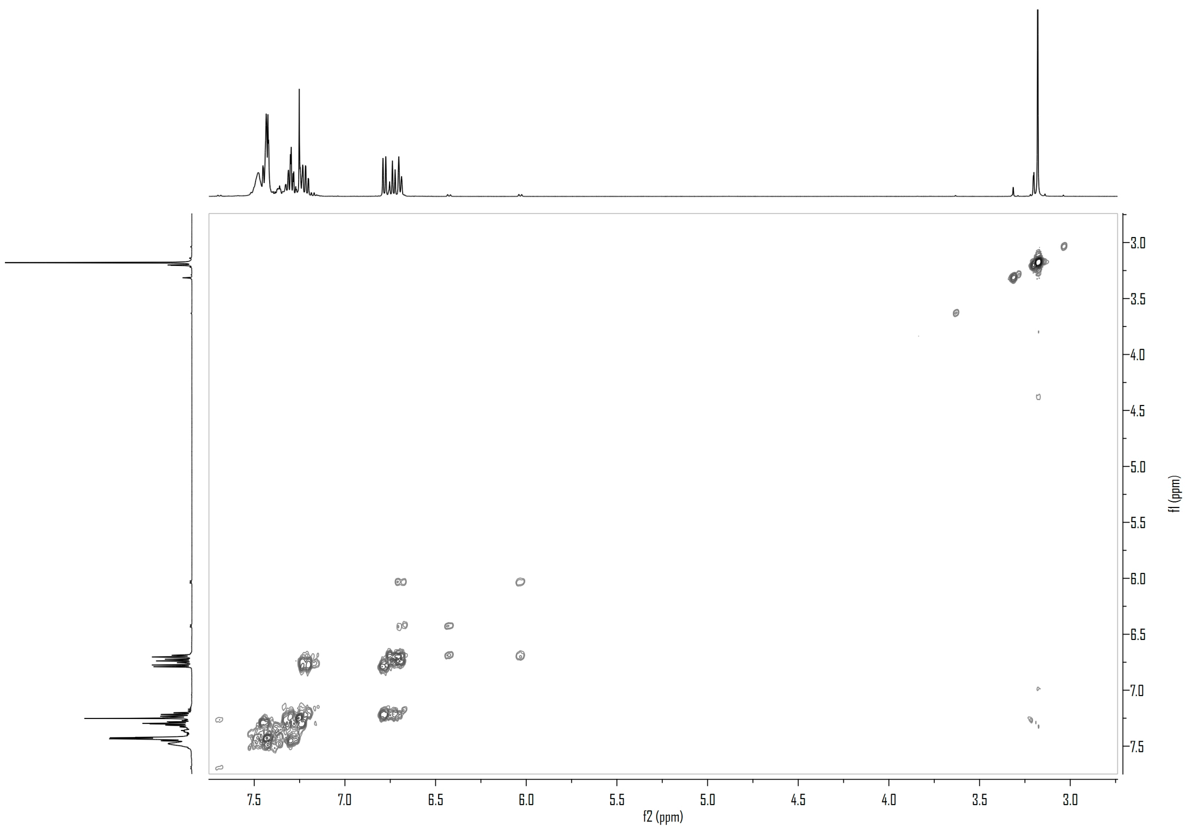

ROESY spectrum of (*Z*)-3m

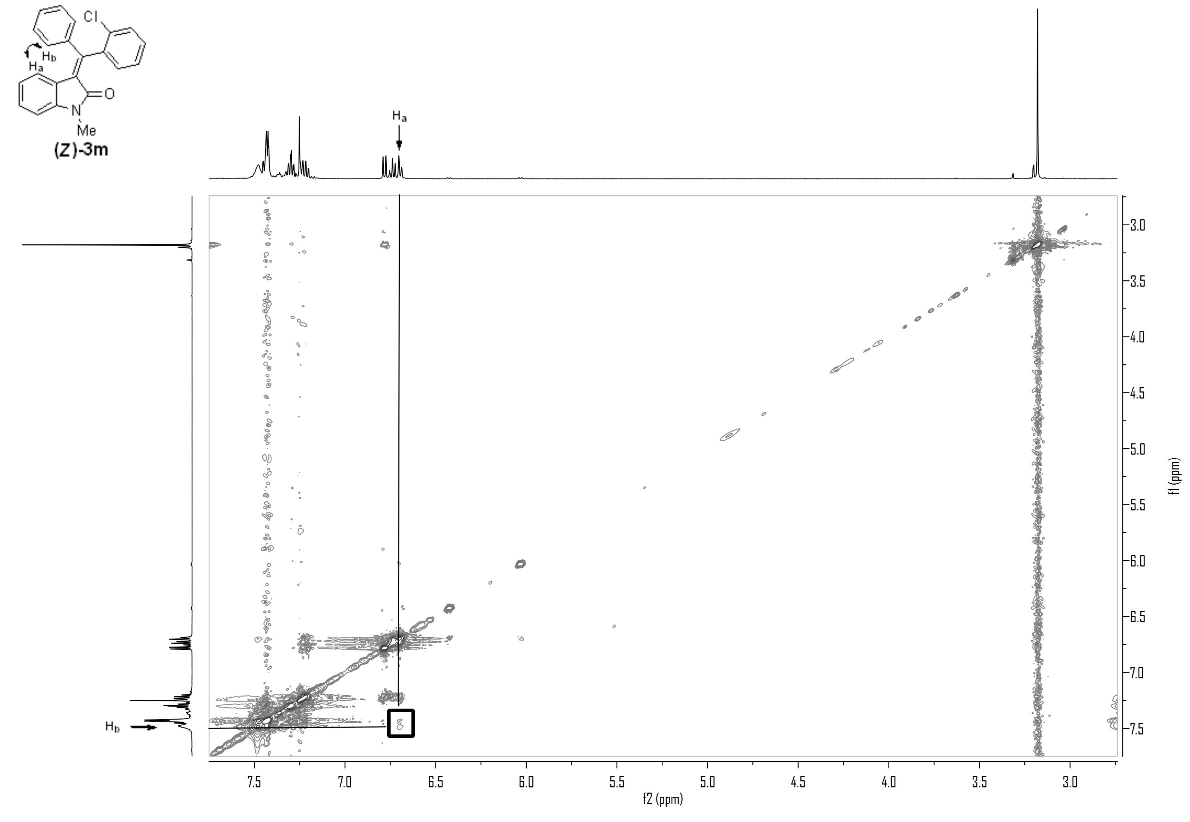

(E)-3-((2-chlorophenyl)(phenyl)methylene)-1-methylindolin-2-one ((E)-3m)

^{1}H NMR spectrum of (E)-3m

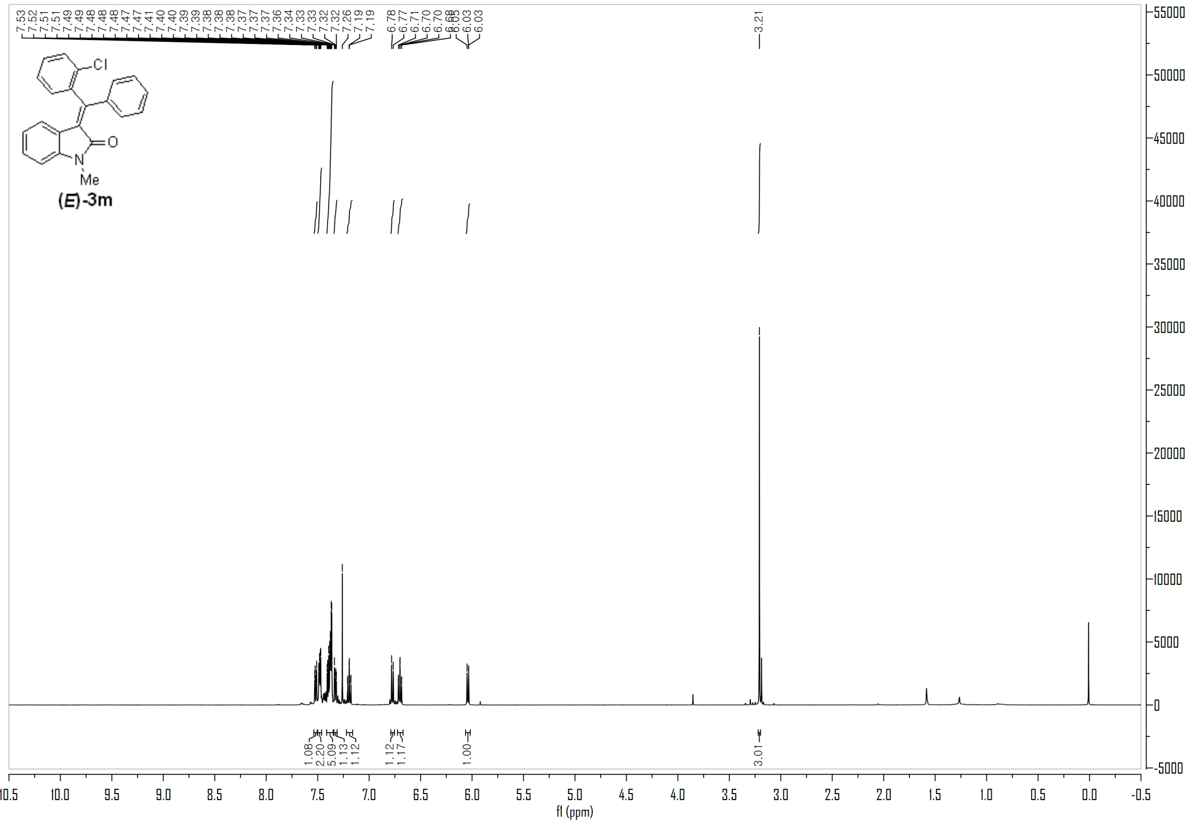

^{13}C NMR spectrum of (E)-3m

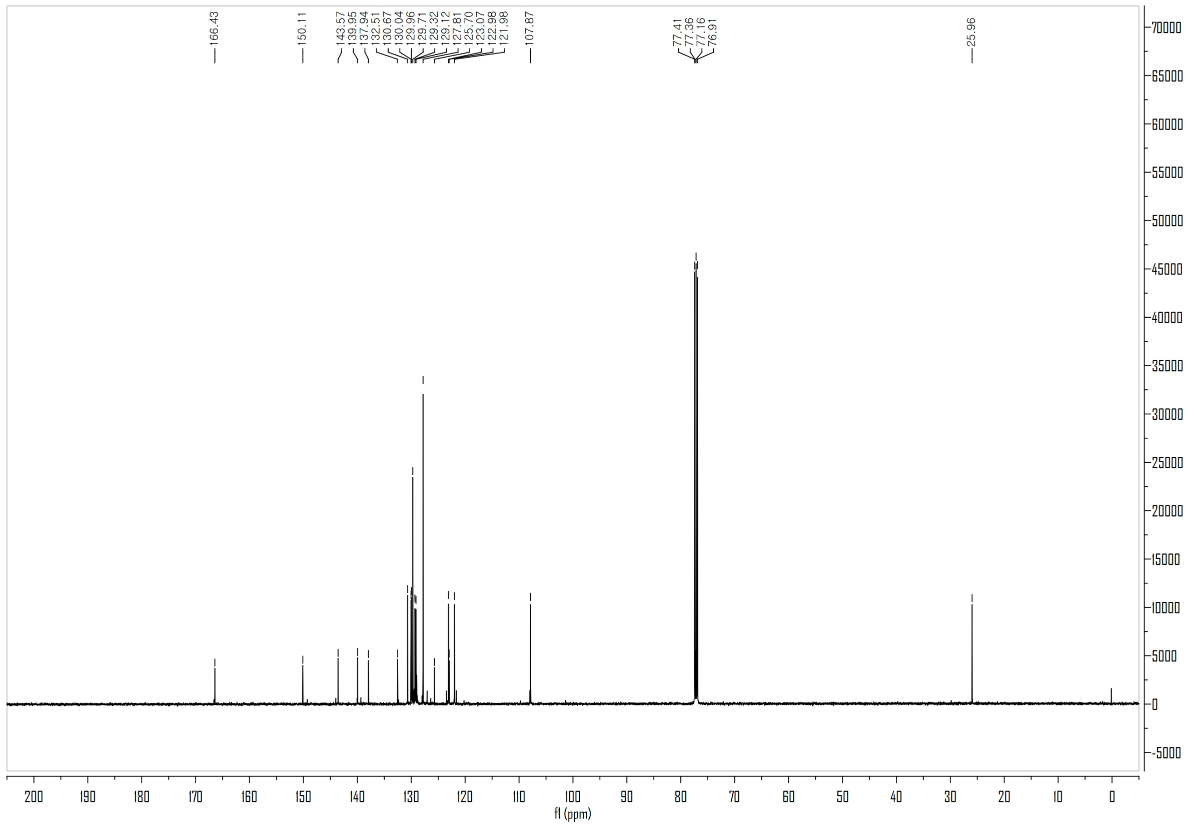

HSQC spectrum of (*E*)-3m

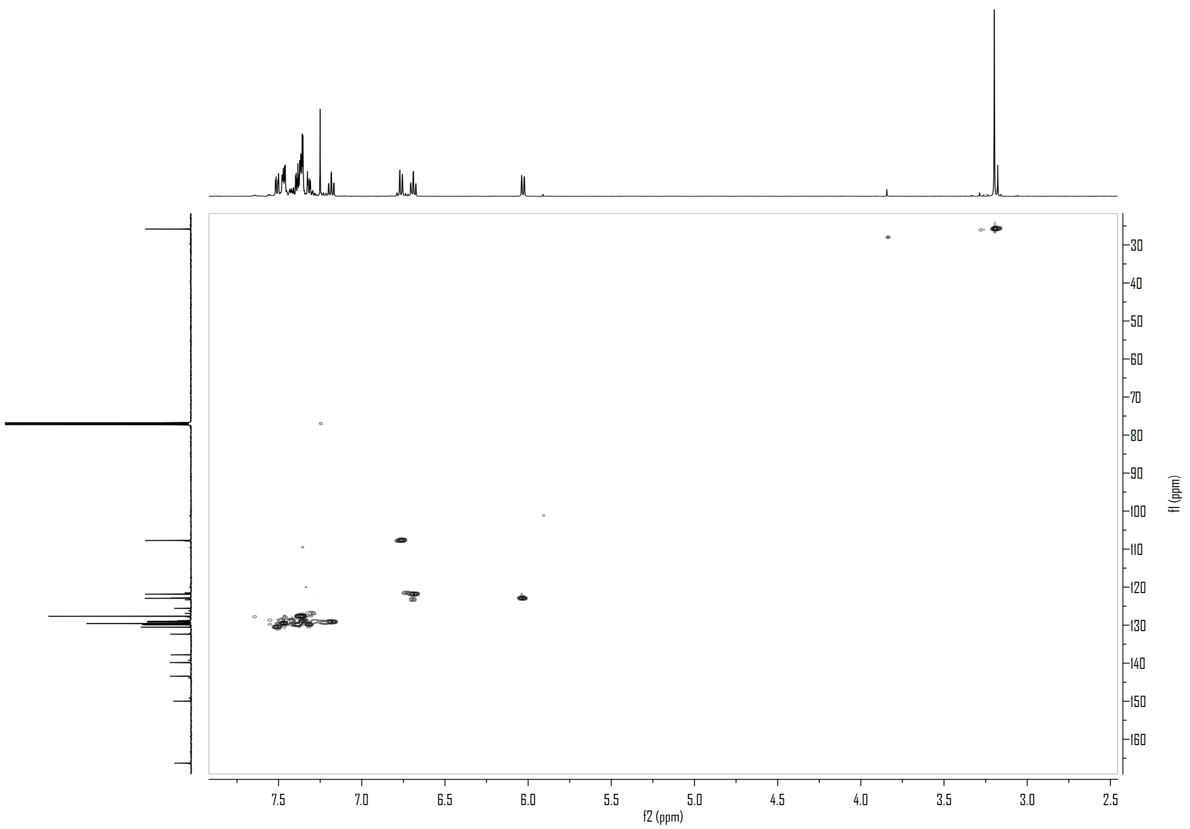

HMBC spectrum of (*E*)-3m

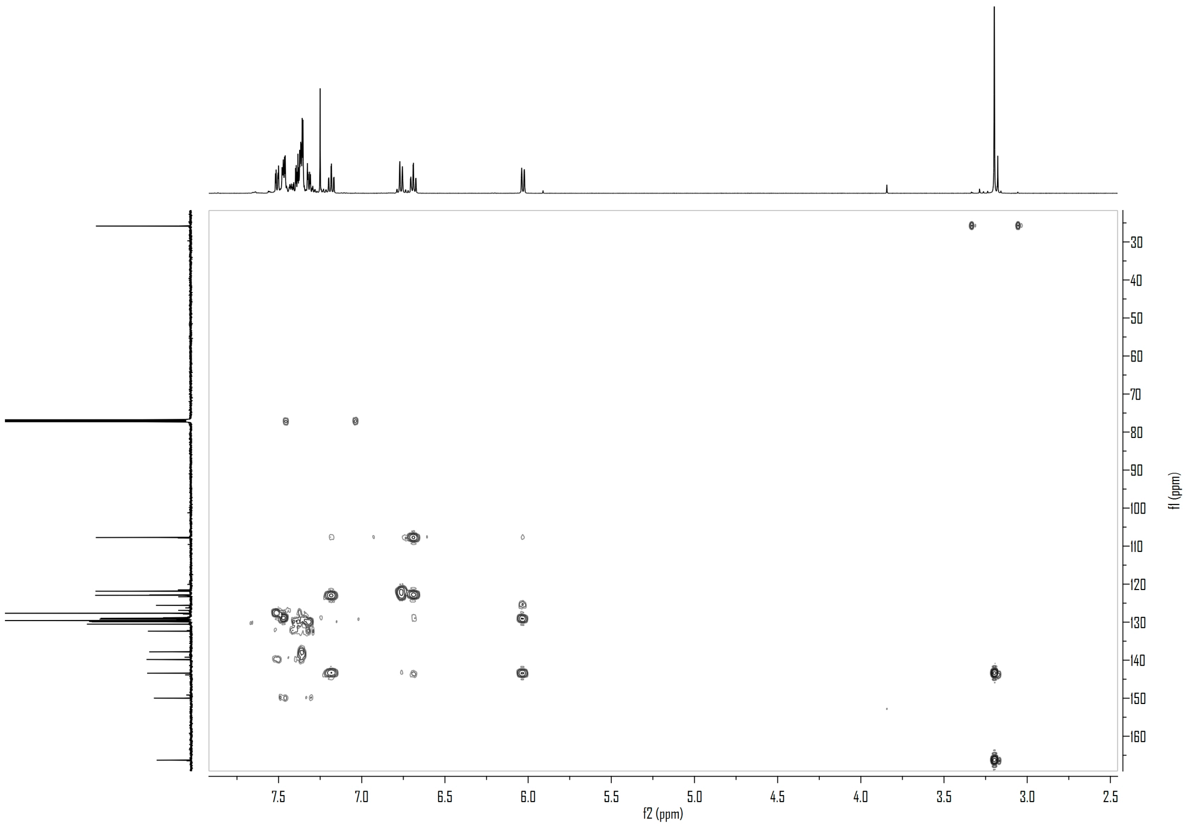

COSY spectrum of (*E*)-3m

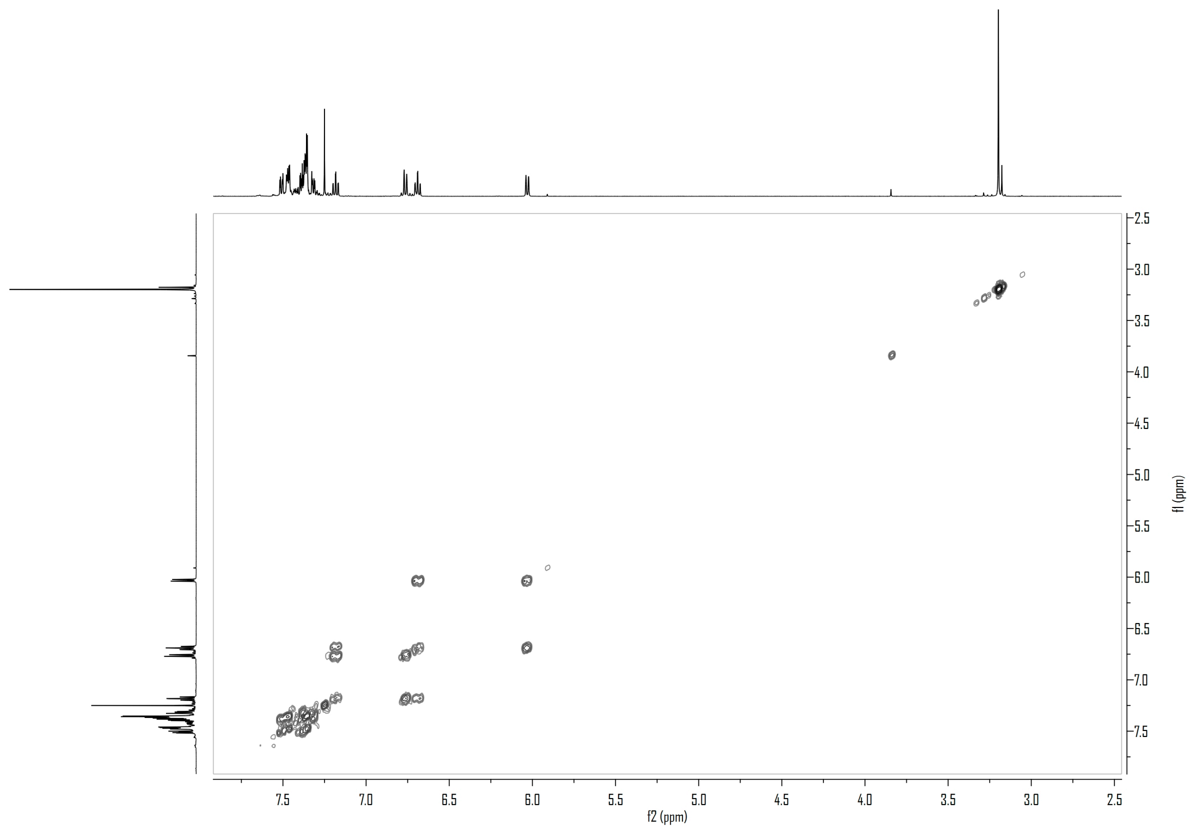

ROESY spectrum of (*E*)-3m

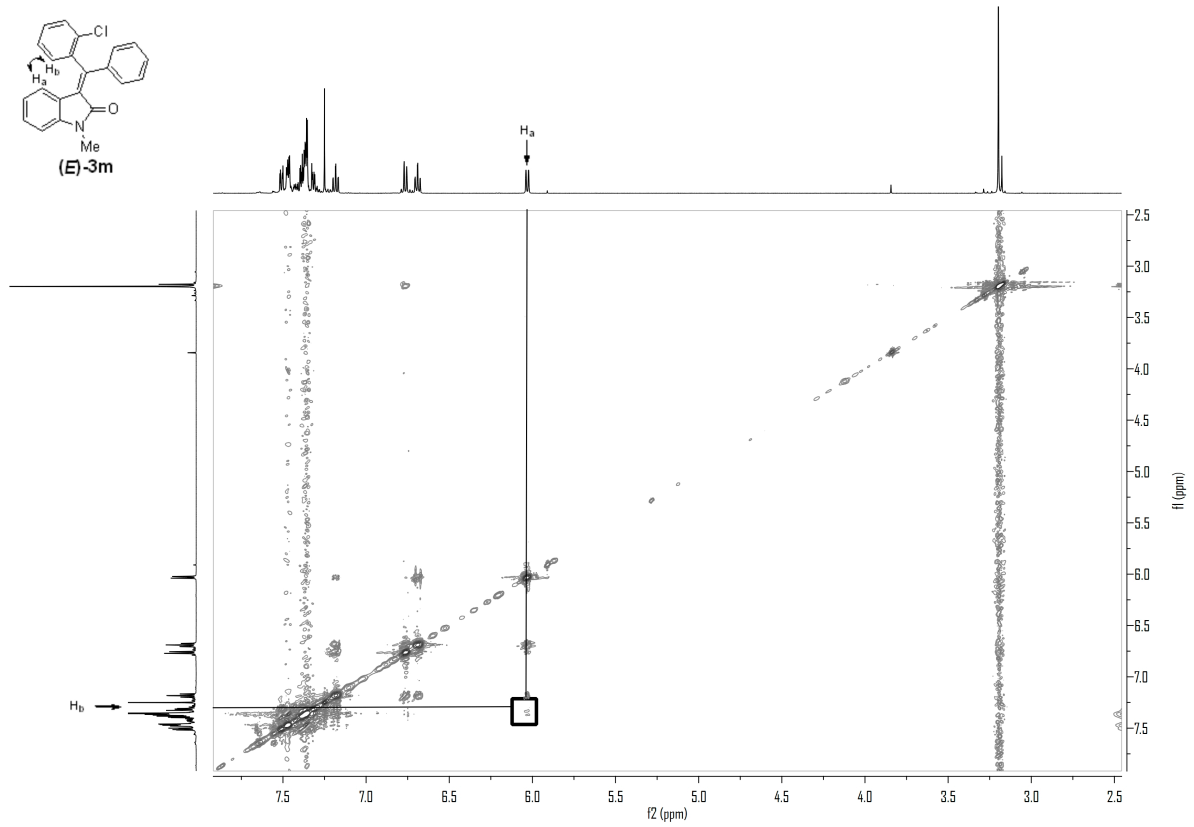

(*Z*)-1-methyl-3-((2-nitrophenyl)(phenyl)methylene)indolin-2-one ((*Z*)-3n)

^{1}H NMR spectrum of (*Z*)-3n

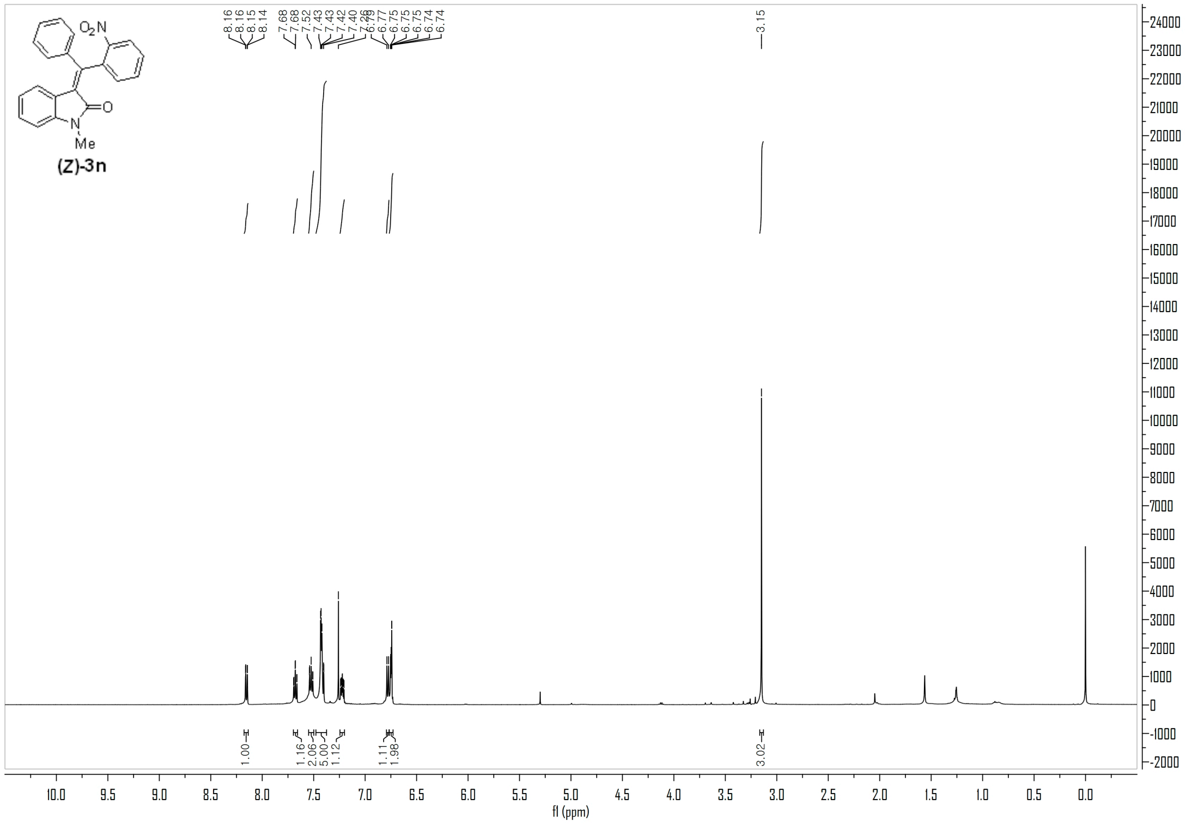

^{13}C NMR spectrum of (*Z*)-3n

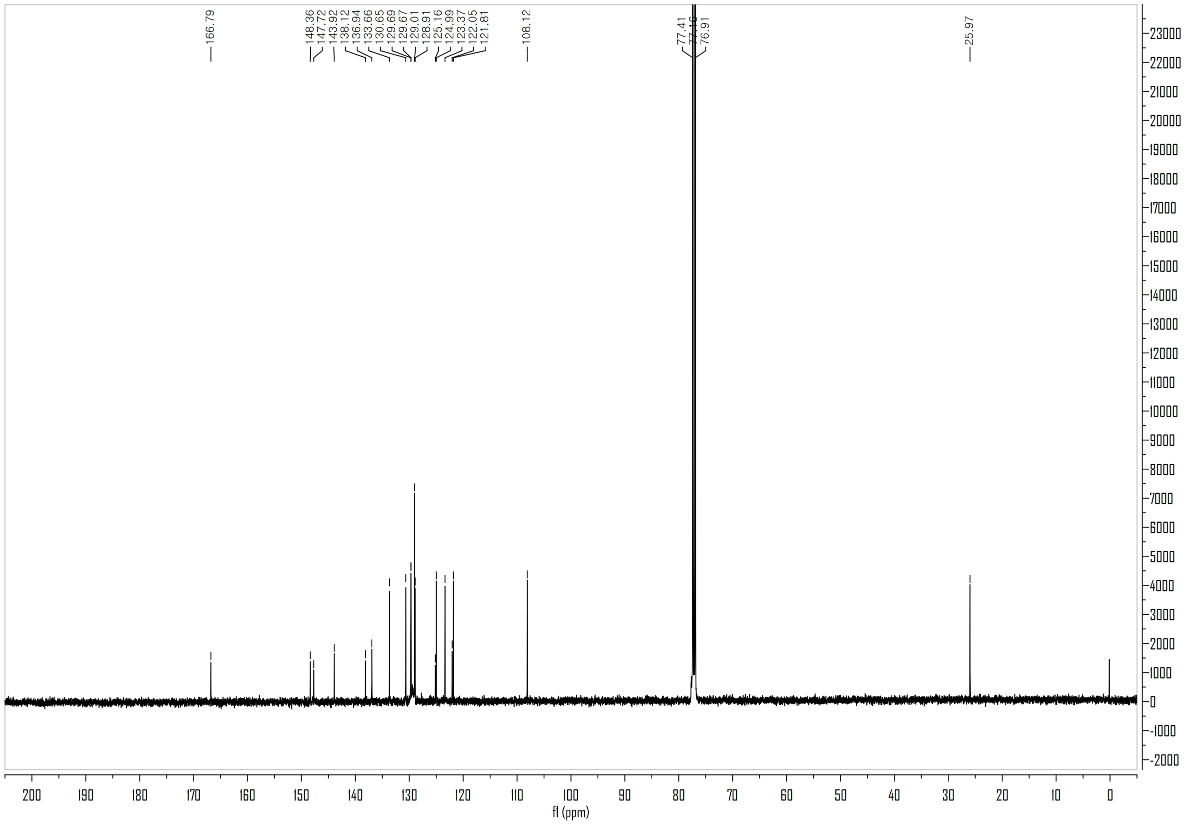

HSQC spectrum of (*Z*)-3n

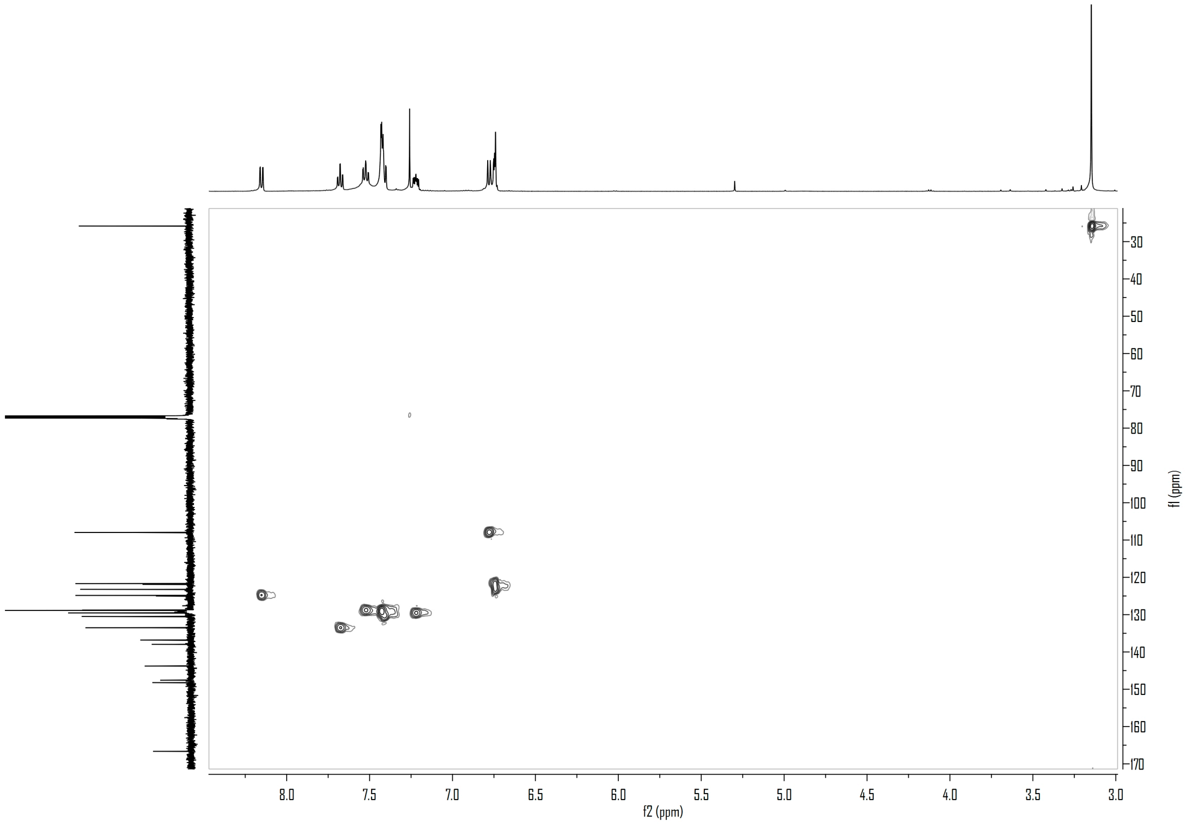

HMBC spectrum of (*Z*)-3n

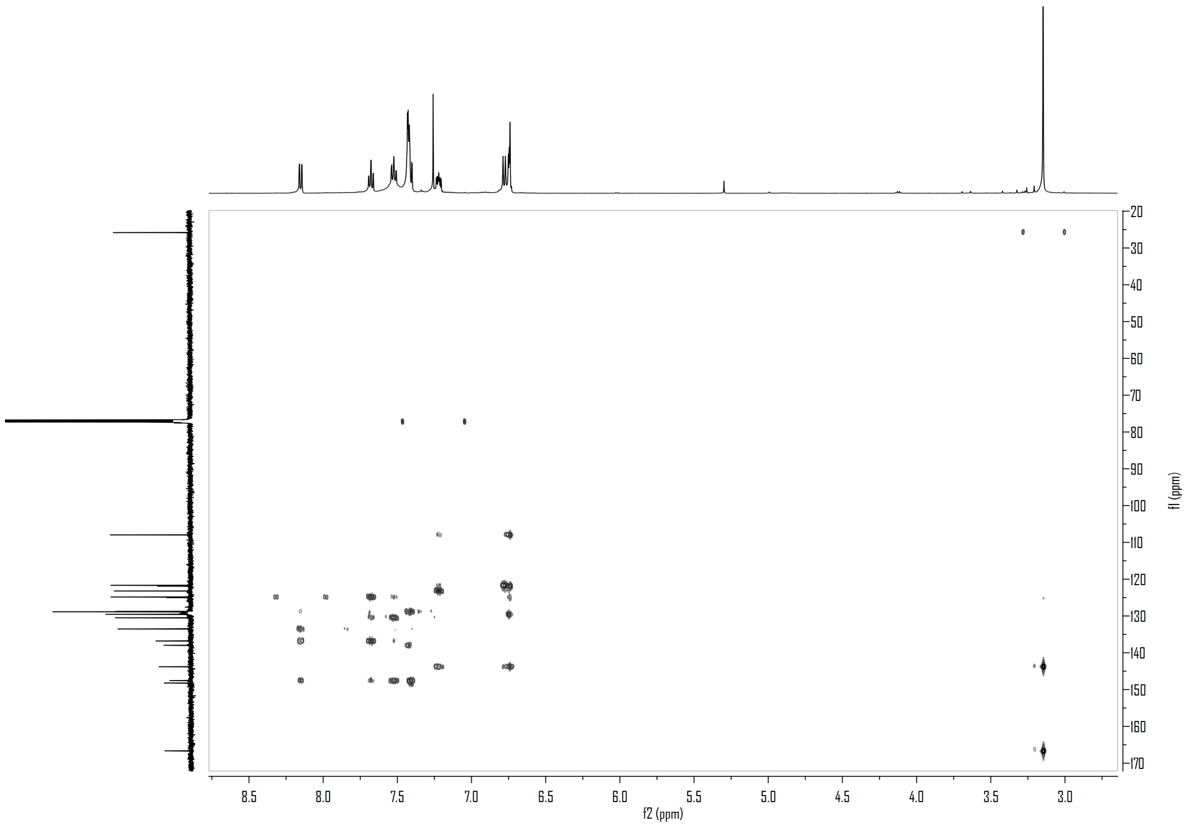

COSY spectrum of (*Z*)-3n

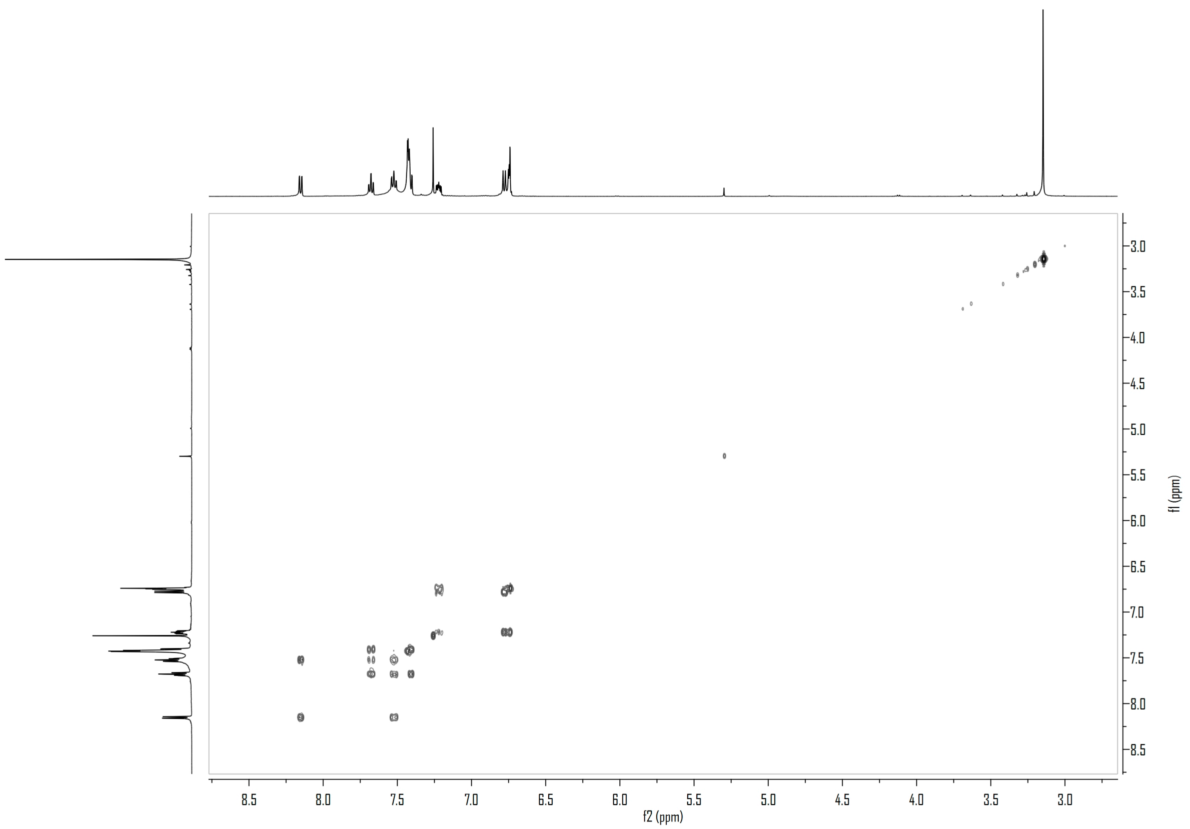

ROESY spectrum of (*Z*)-3n

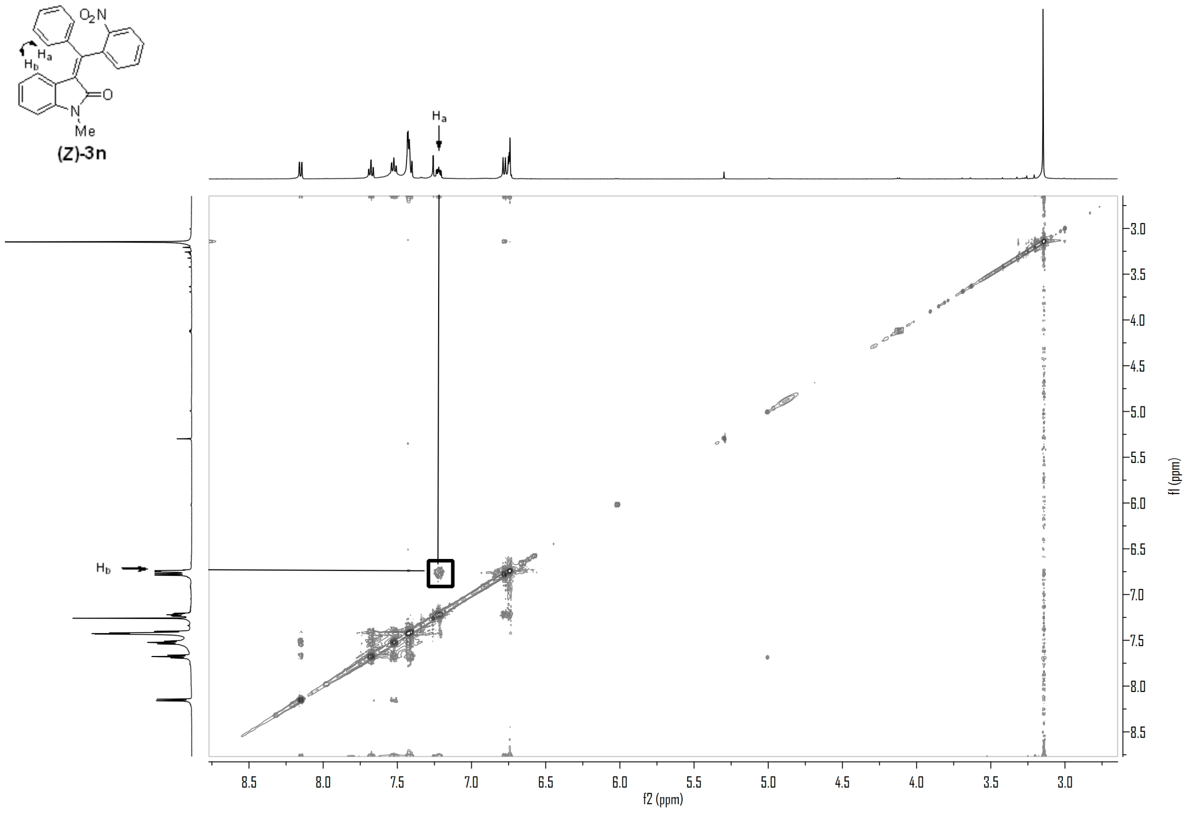

(E)-1-methyl-3-(phenyl-(pyridin-4-yl)methylene)indolin-2-one ((E)-3o)

^{1}H NMR spectrum of (E)-3o

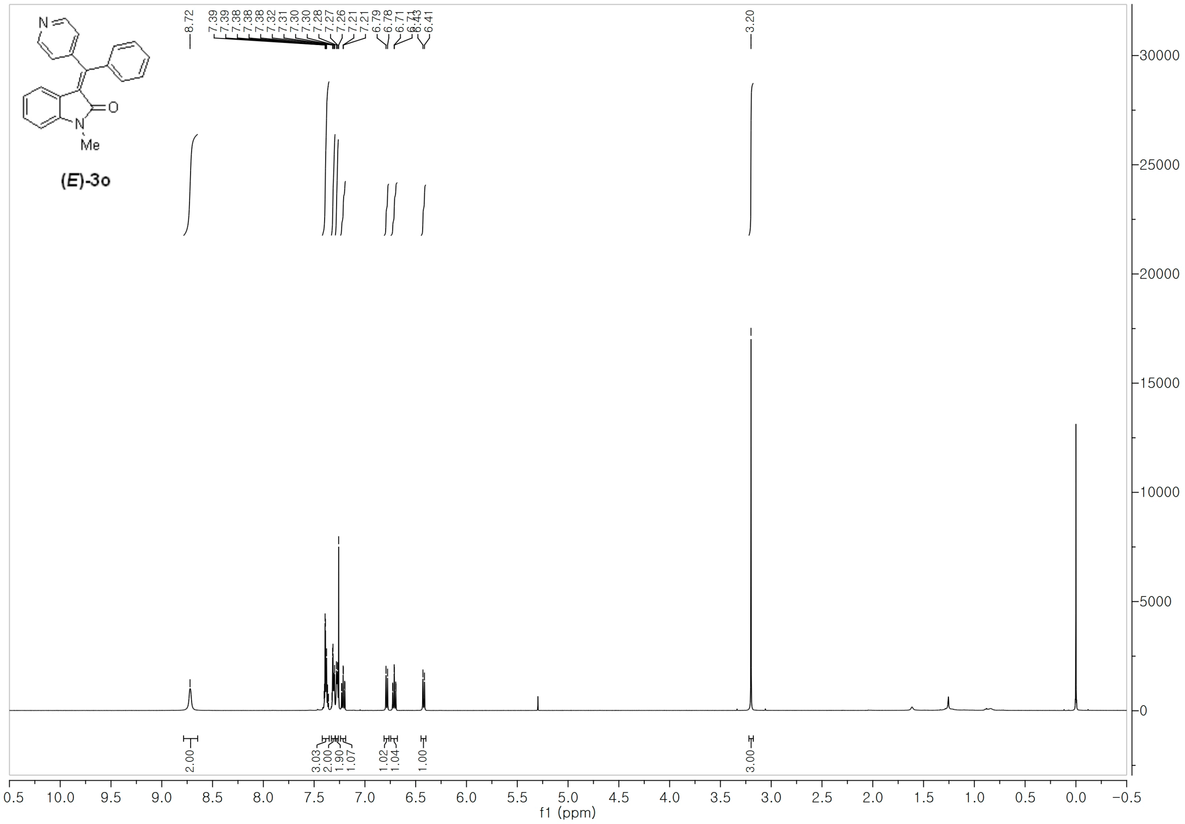

^{13}C NMR spectrum of (E)-3o

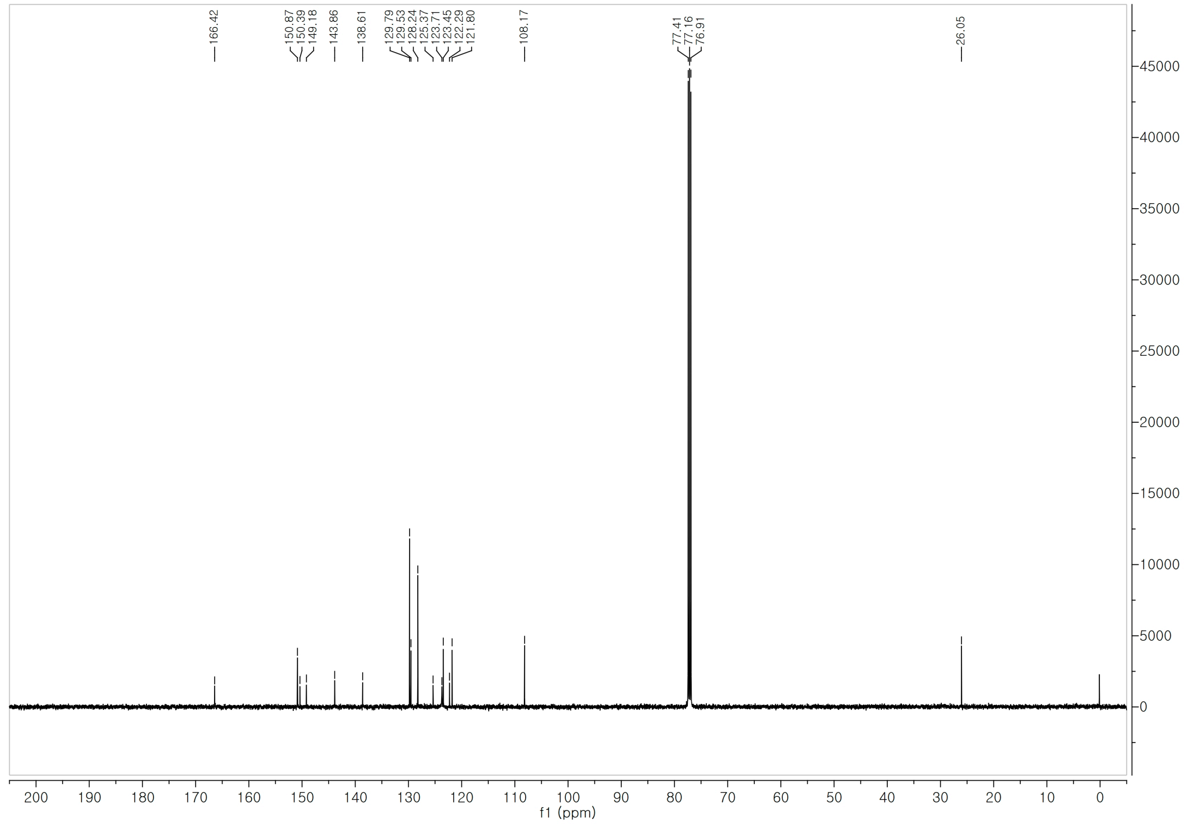

HSQC spectrum of (*E*)-3o

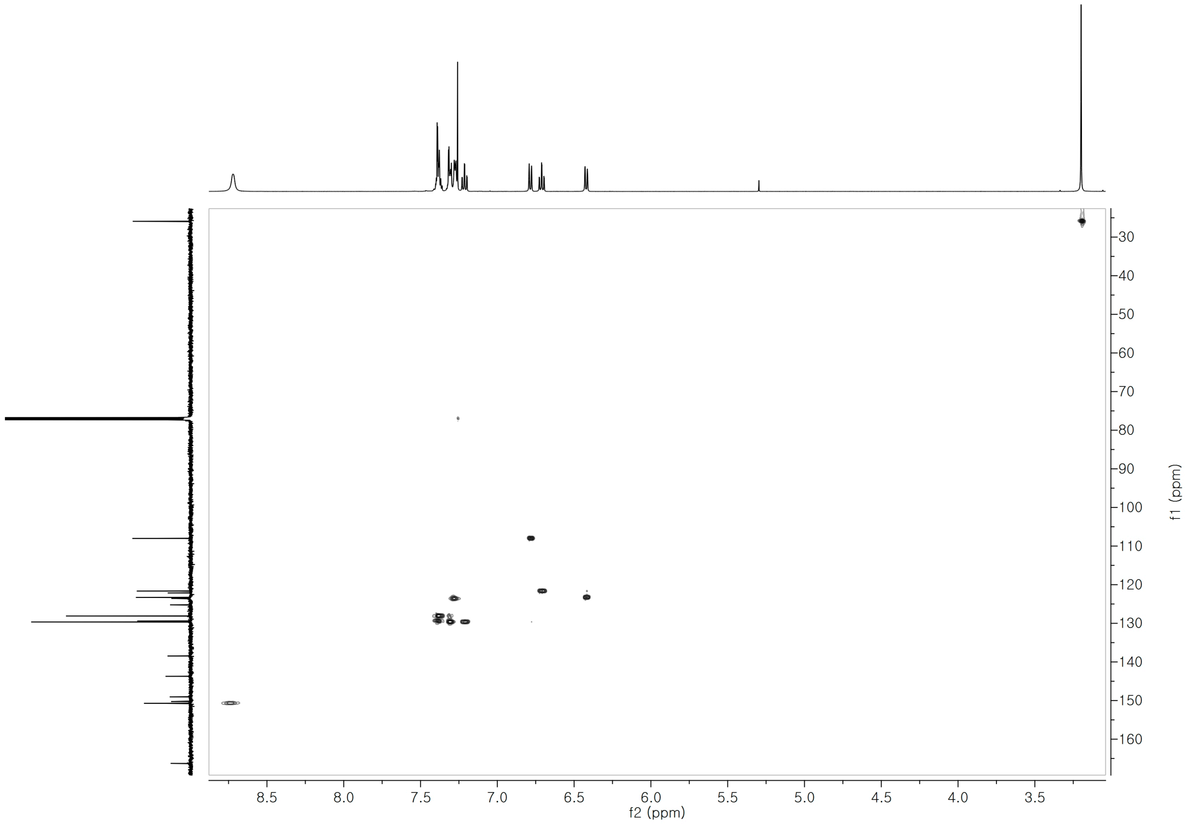

HMBC spectrum of (*E*)-3o

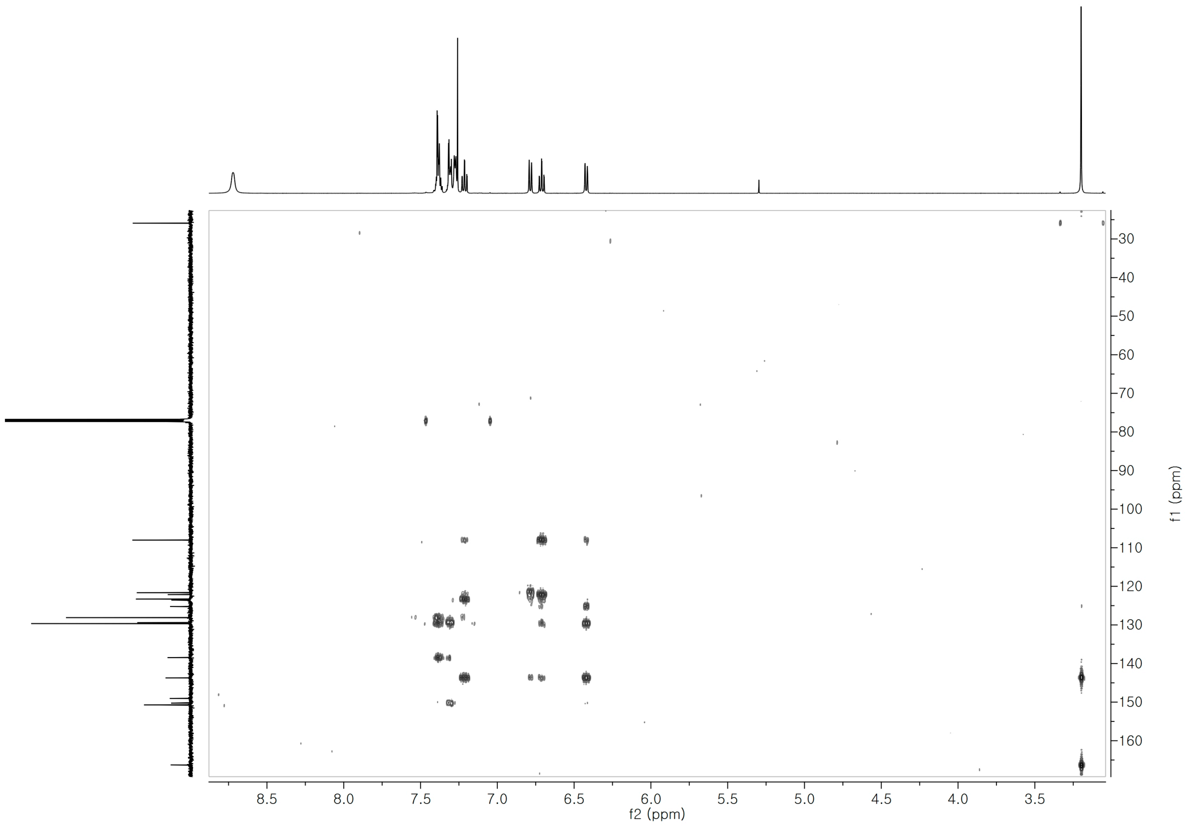

COSY spectrum of (*E*)-3o

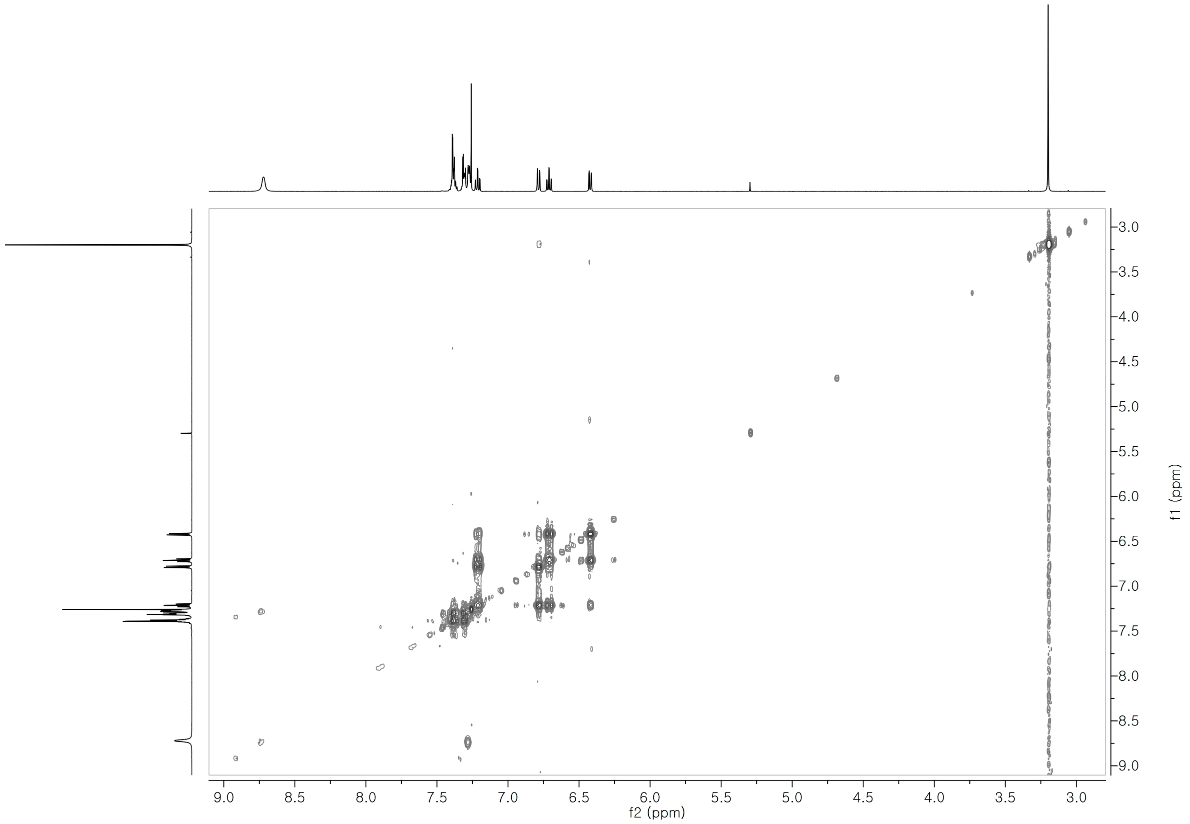

ROESY spectrum of (*E*)-3o

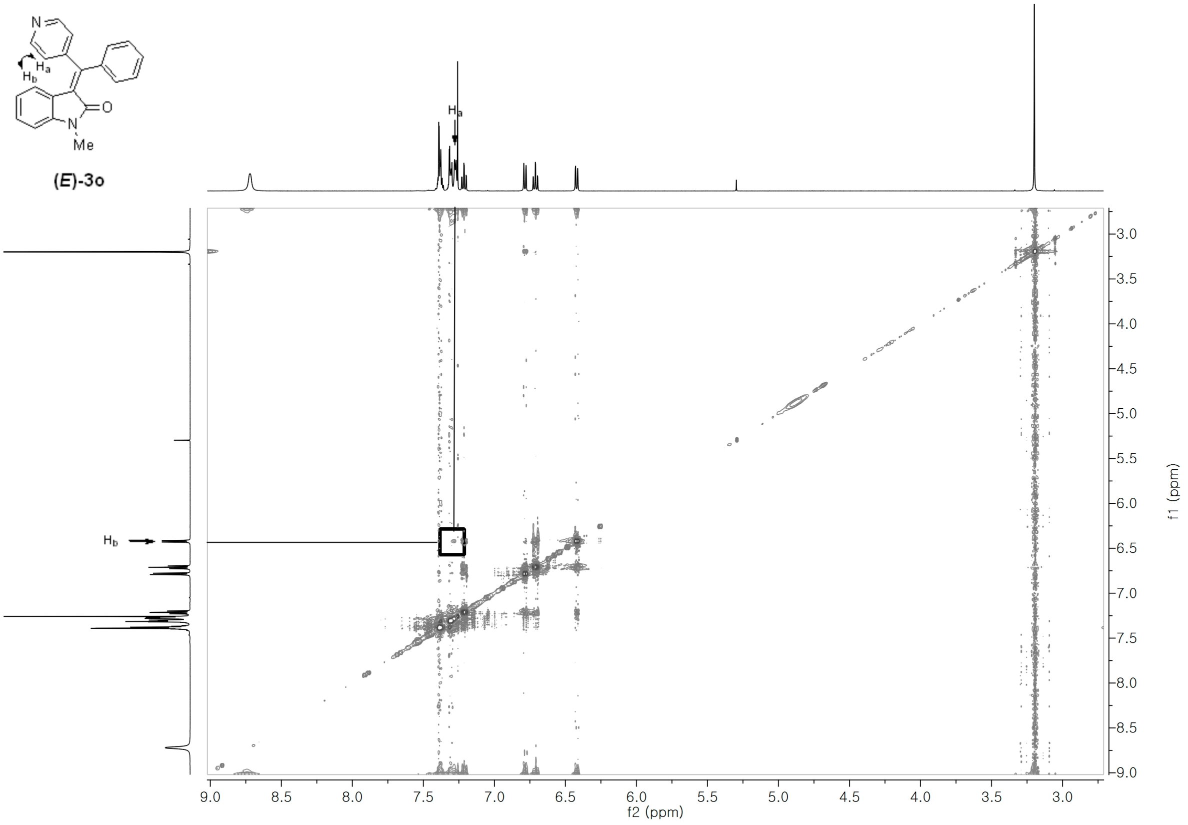

(*E*)-1-methyl-3-(phenyl(pyridin-3-yl)methylene)indolin-2-one ((*E*)-3p)

^{1}H NMR spectrum of (*E*)-3p

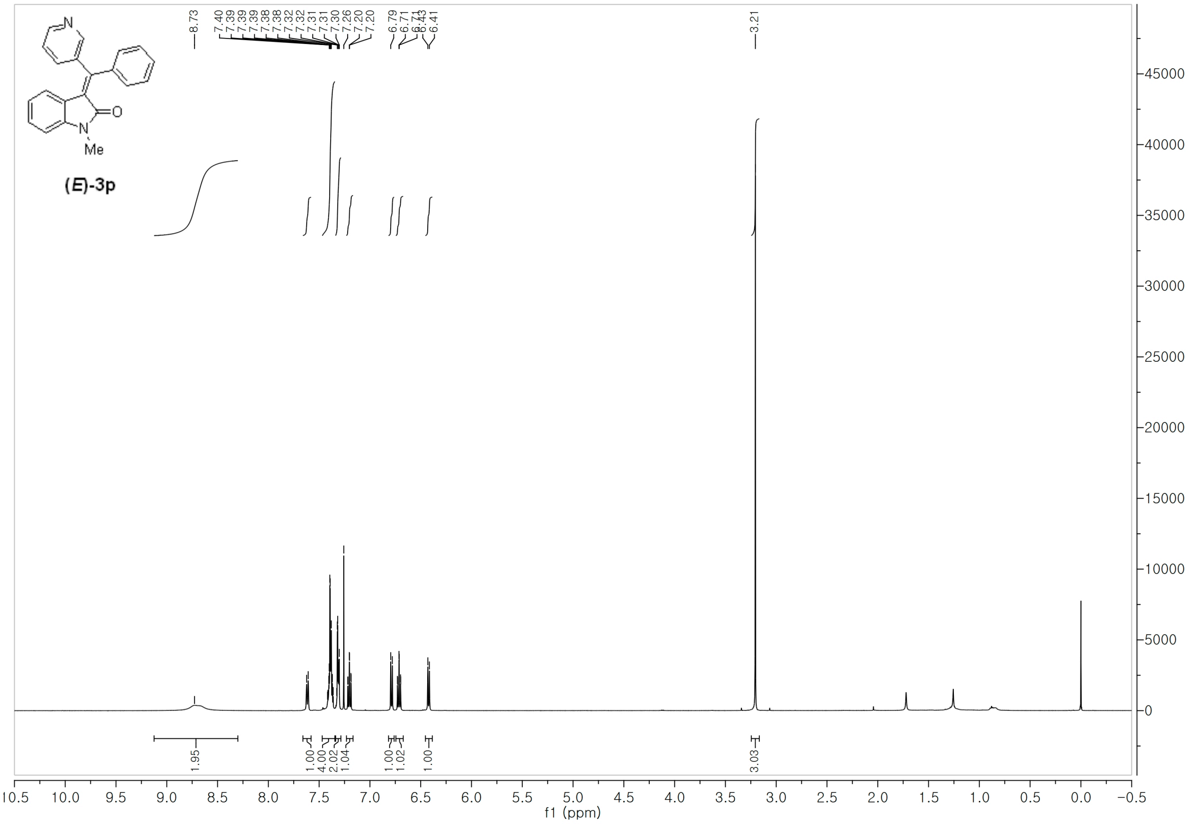

^{13}C NMR spectrum of (*E*)-3p

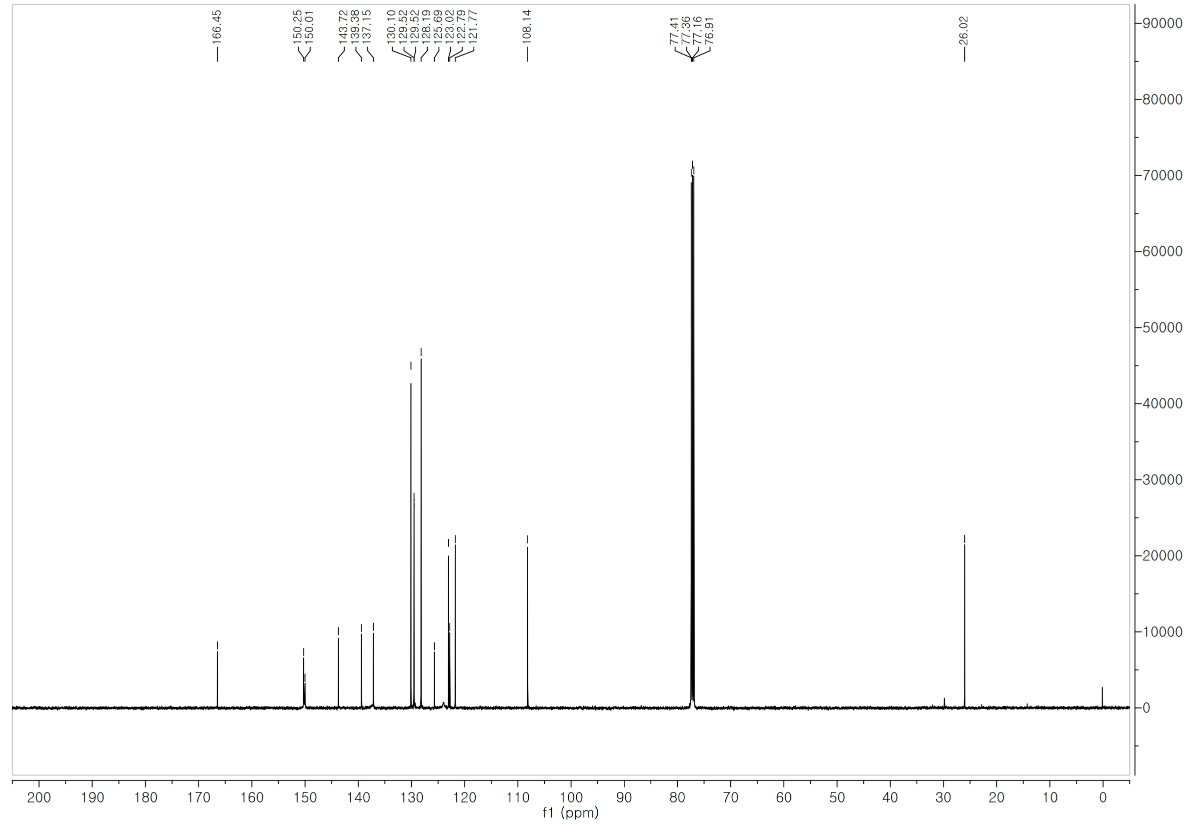

HSQC spectrum of (*E*)-3p

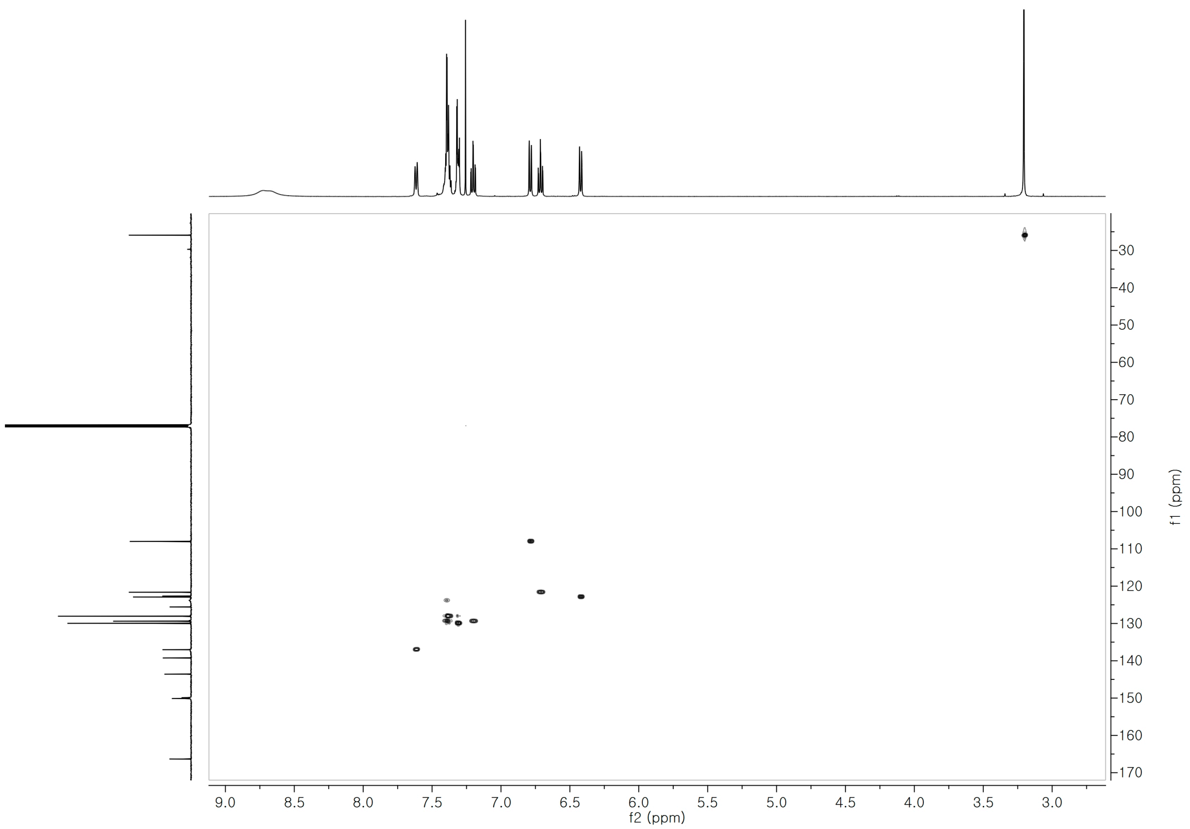

HMBC spectrum of (*E*)-3p

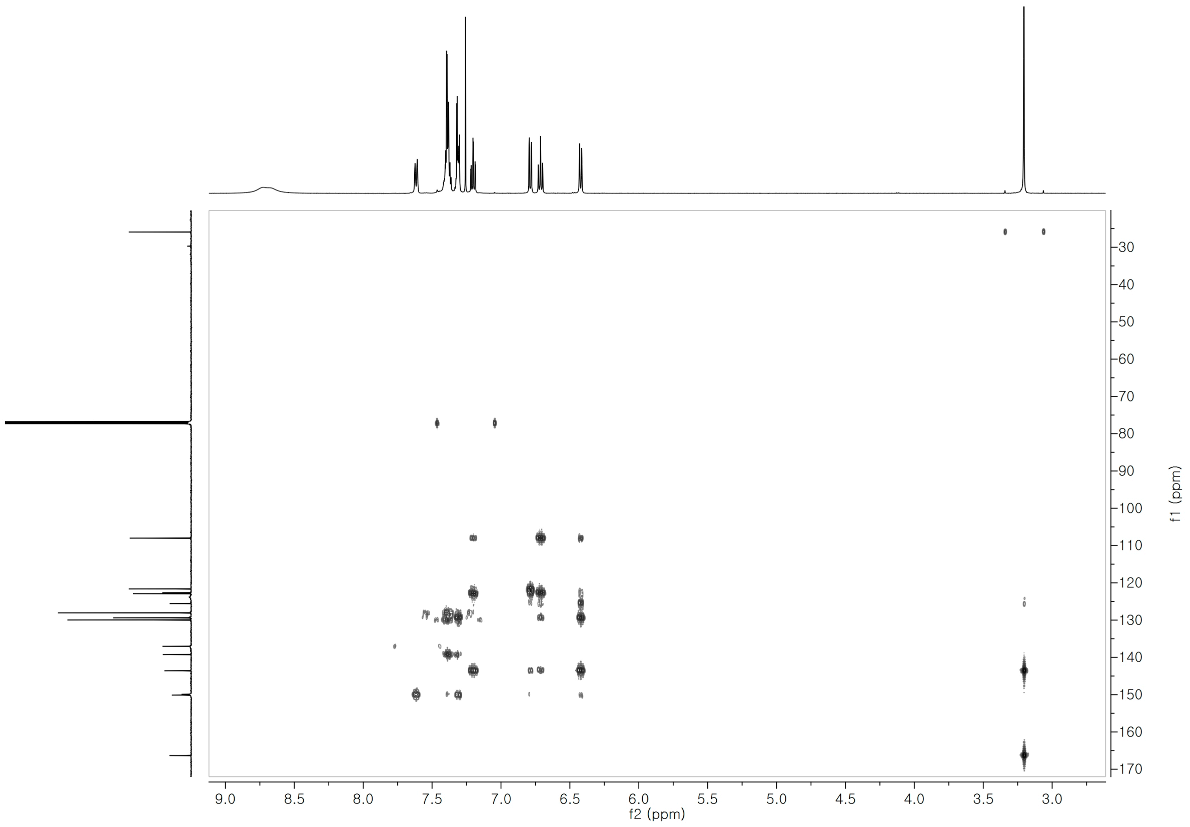

COSY spectrum of (*E*)-3p

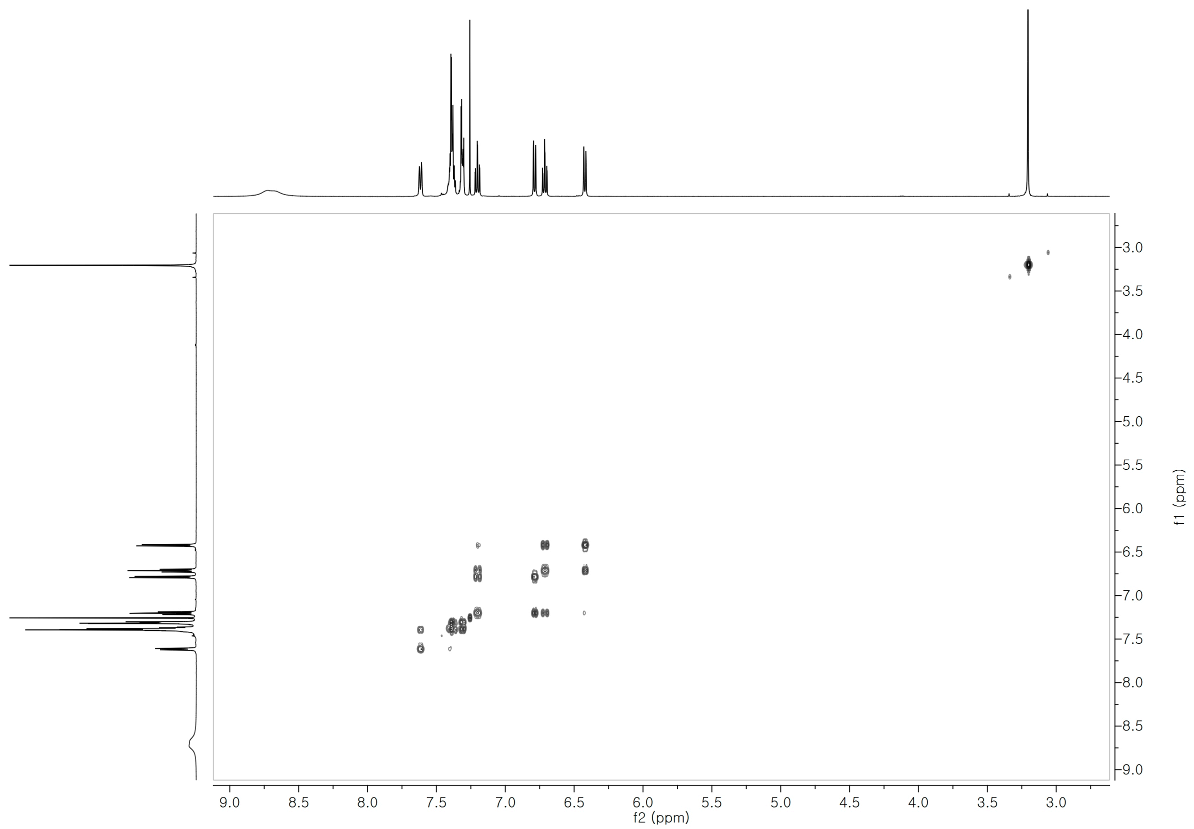

ROESY spectrum of (*E*)-3p

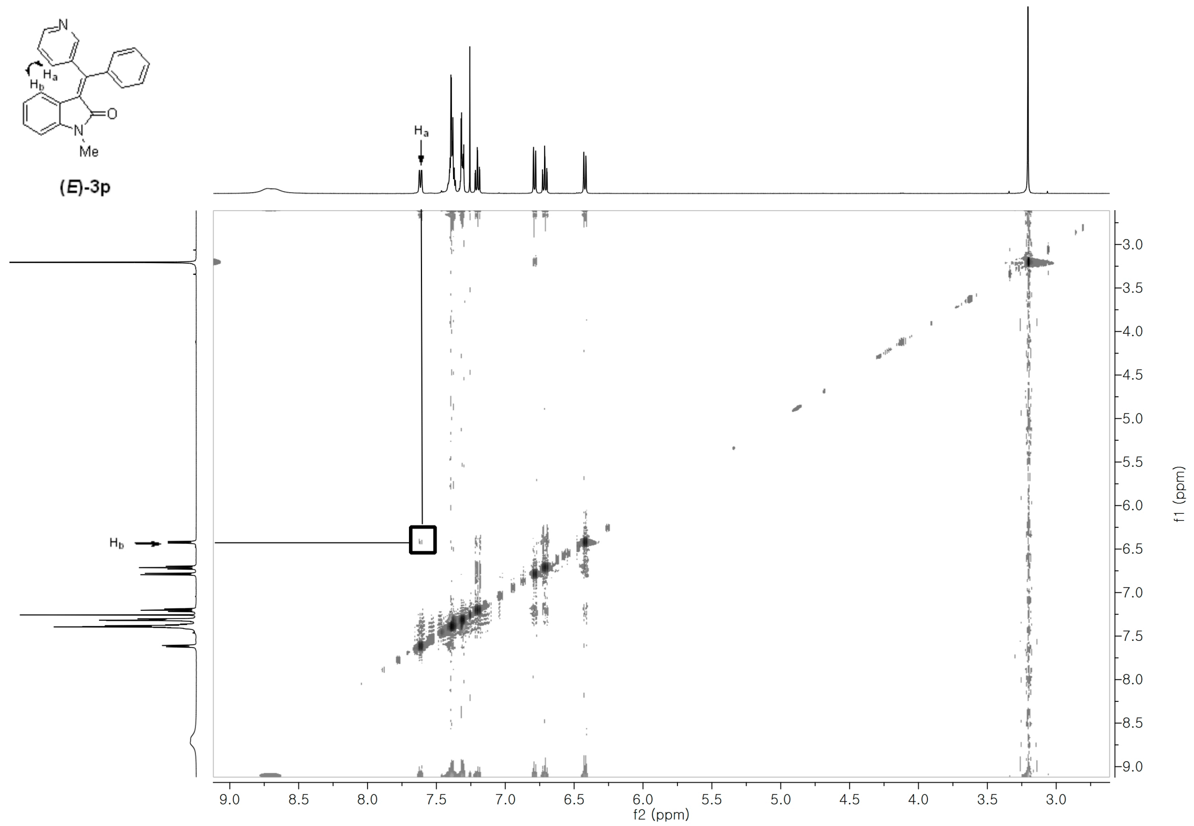

(*Z*)-1-methyl-3-(phenyl(pyridin-3-yl)methylene)indolin-2-one ((*Z*)-3p)

^{1}H NMR spectrum of (*Z*)-3p

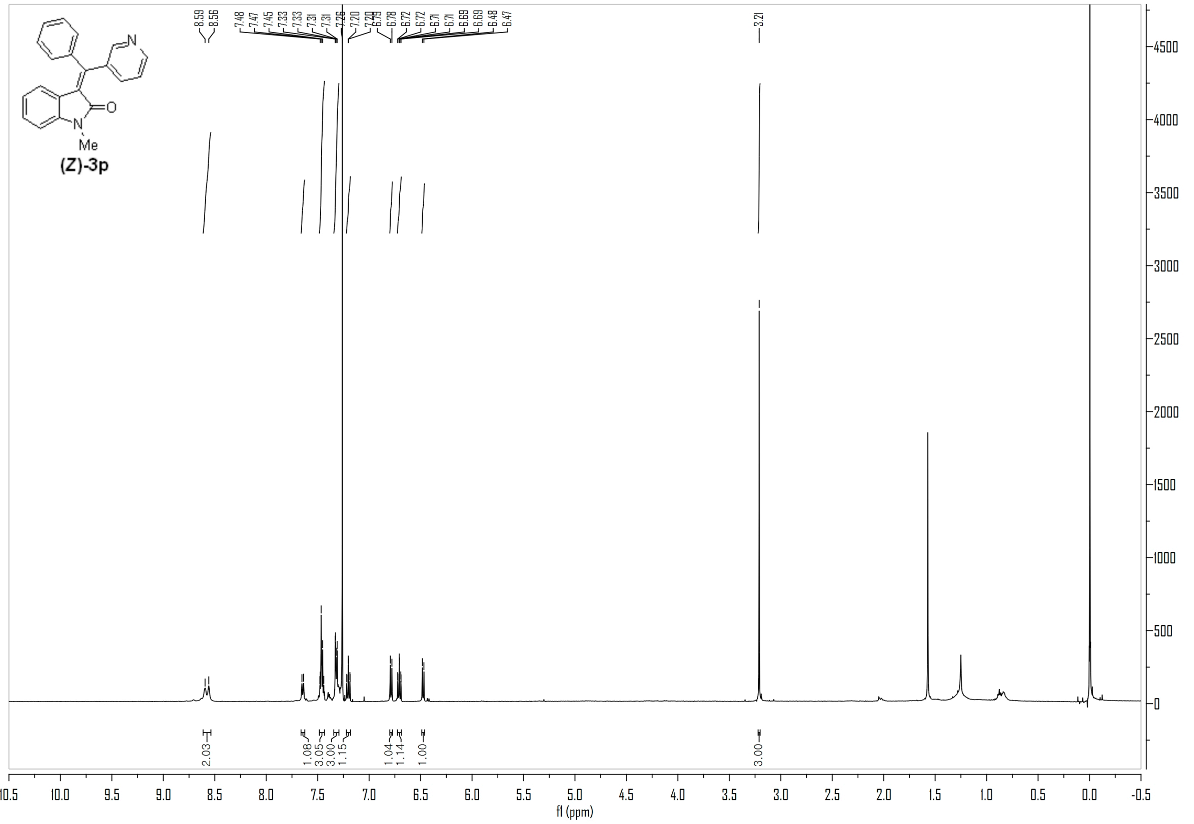

^{13}C NMR spectrum of (*Z*)-3p

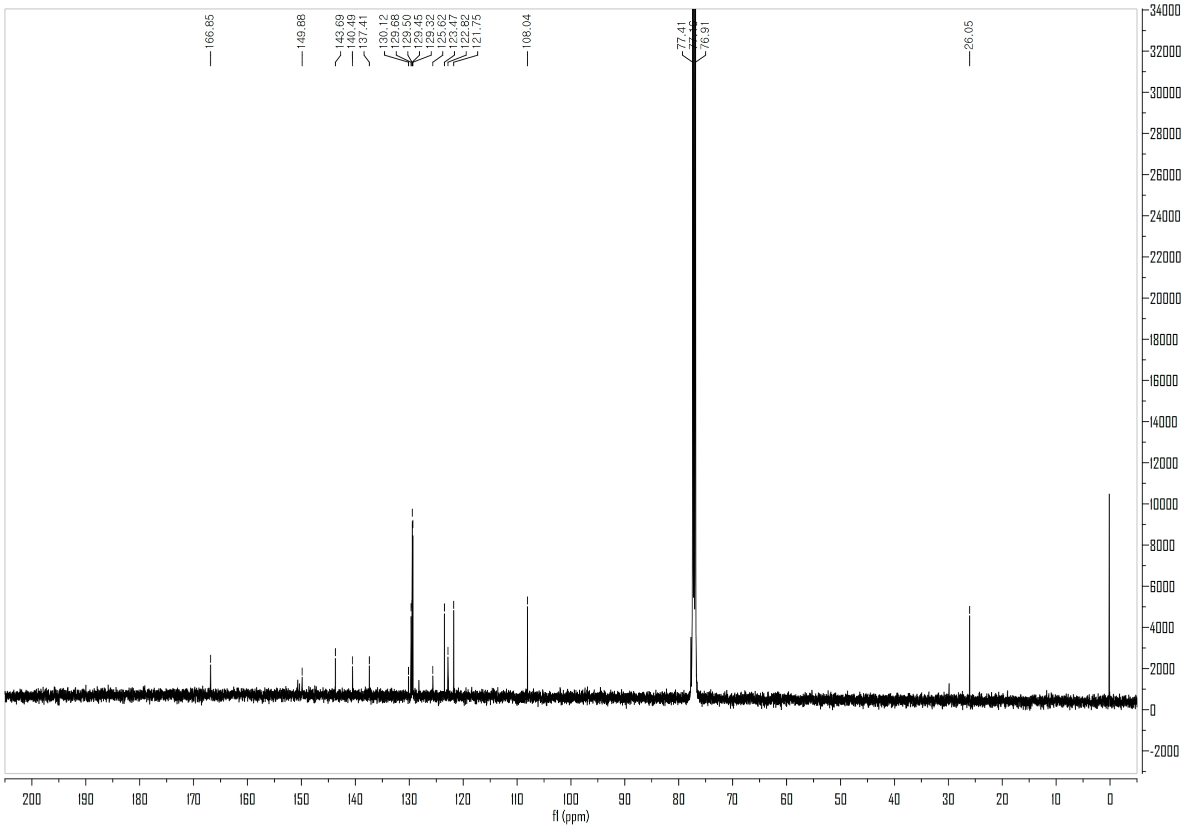

HSQC spectrum of (*Z*)-3p

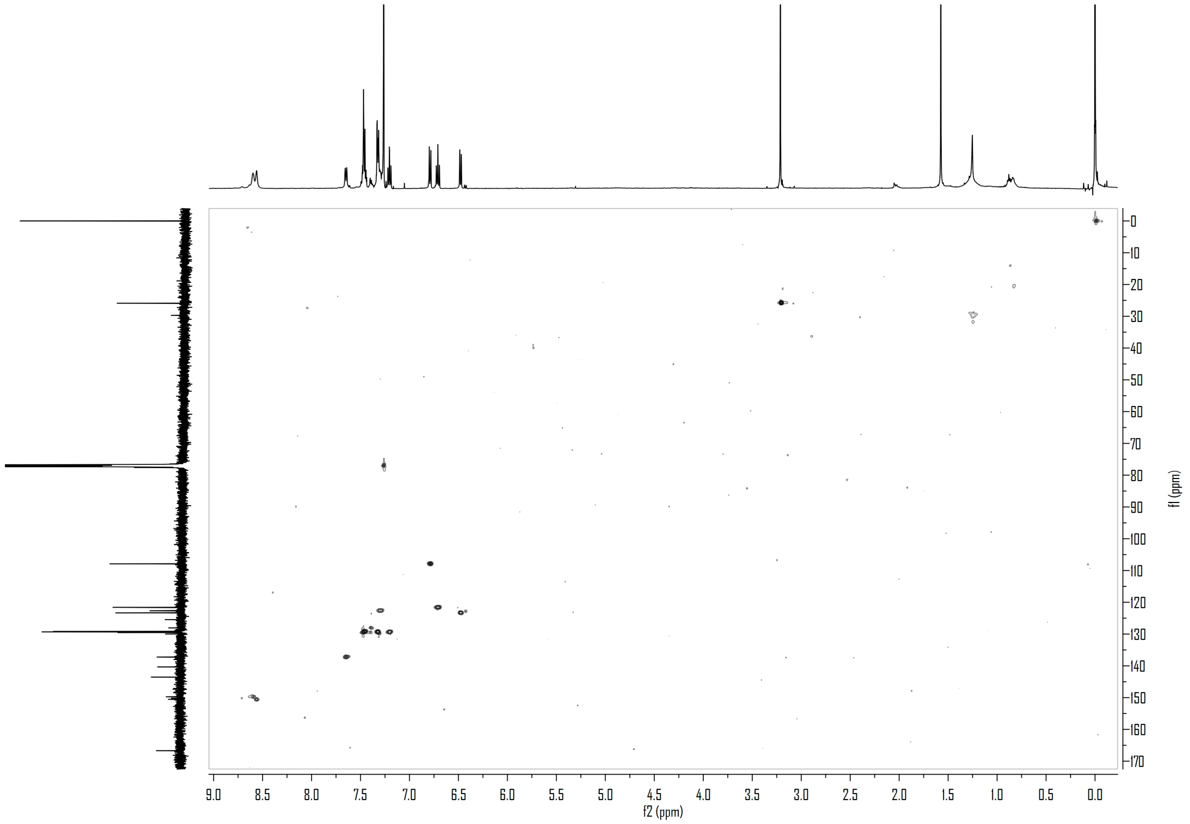

HMBC spectrum of (*Z*)-3p

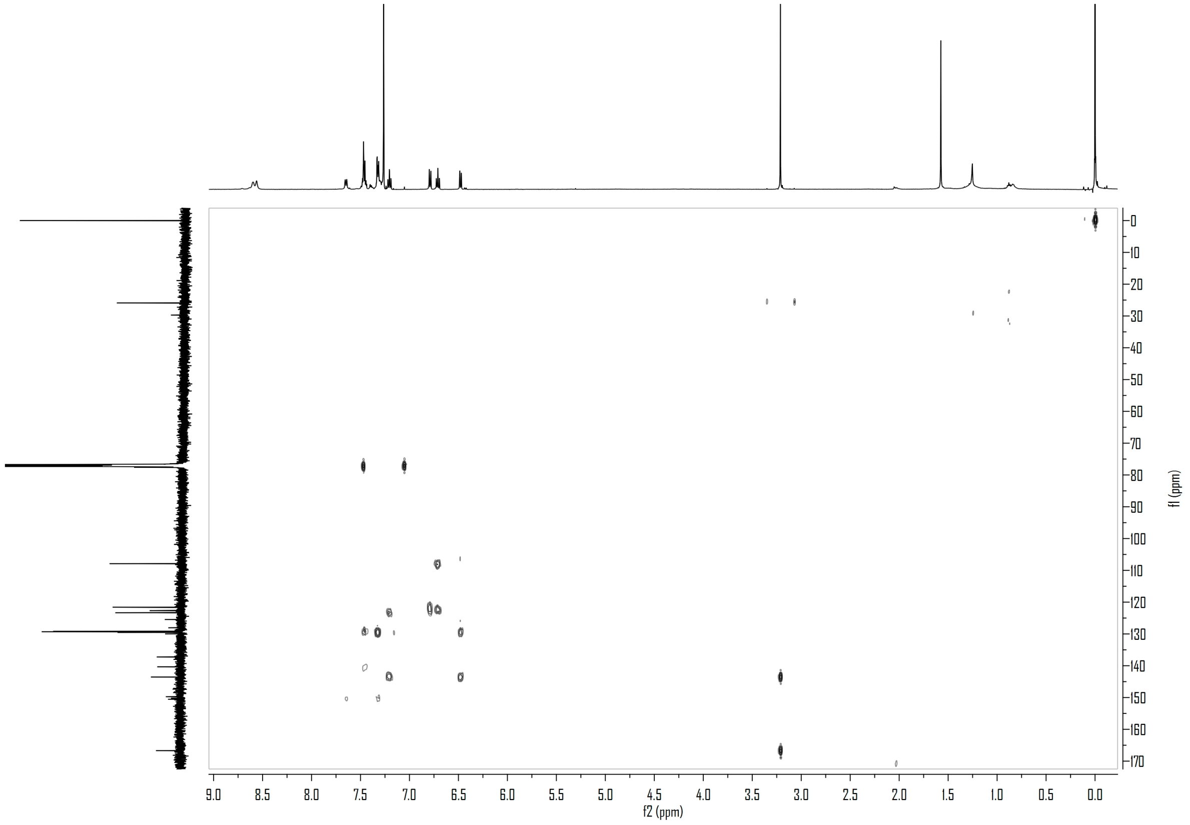

COSY spectrum of (*Z*)-3p

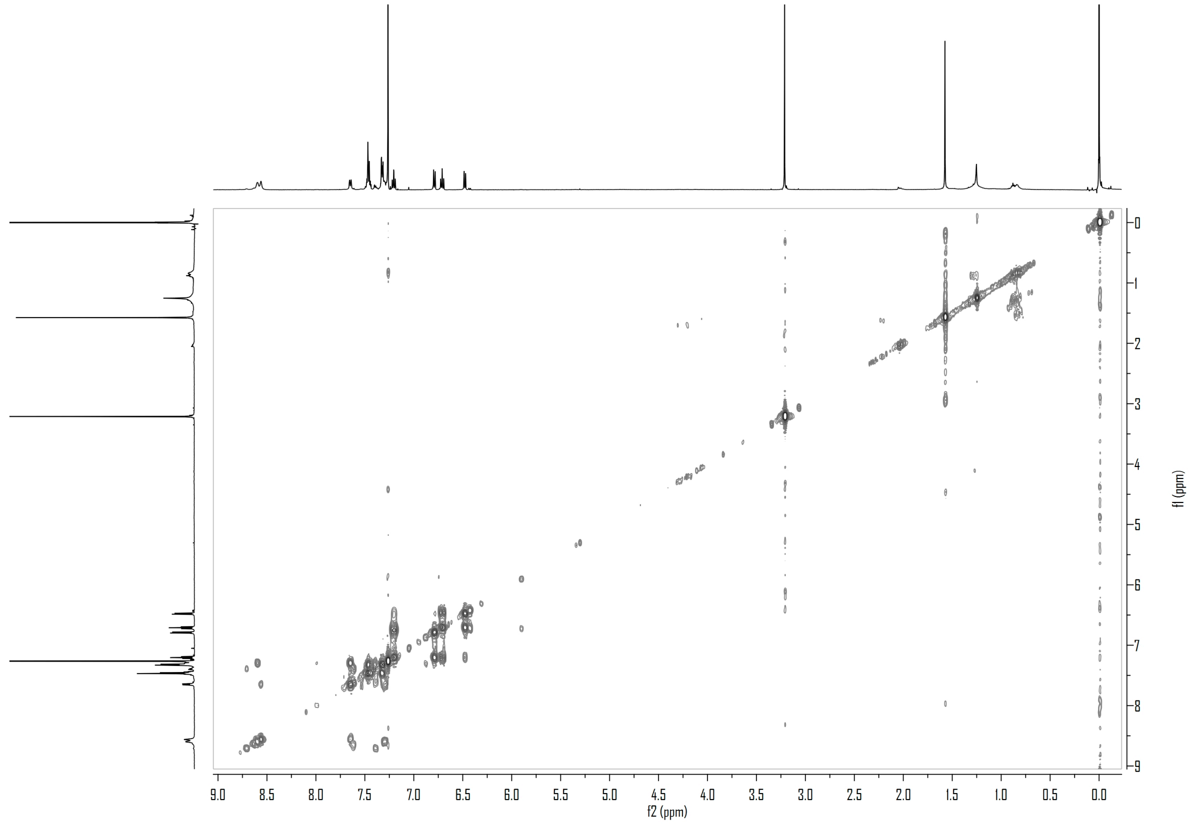

ROESY spectrum of (*Z*)-3p

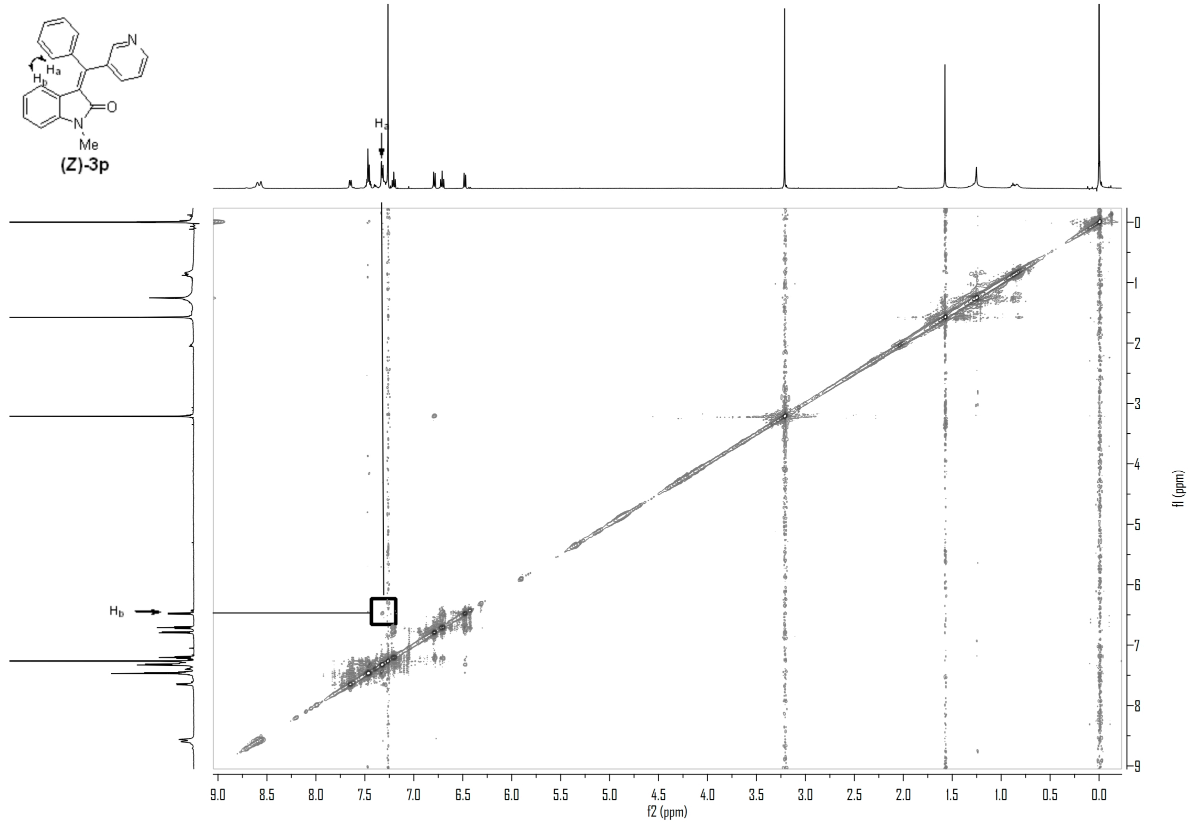

(*E*)-3-(benzo[b]thiophen-3-yl(phenyl)methylene)-1-methylindolin-2-one ((*E*)-3q)

^{1}H NMR spectrum of (*E*)-3q

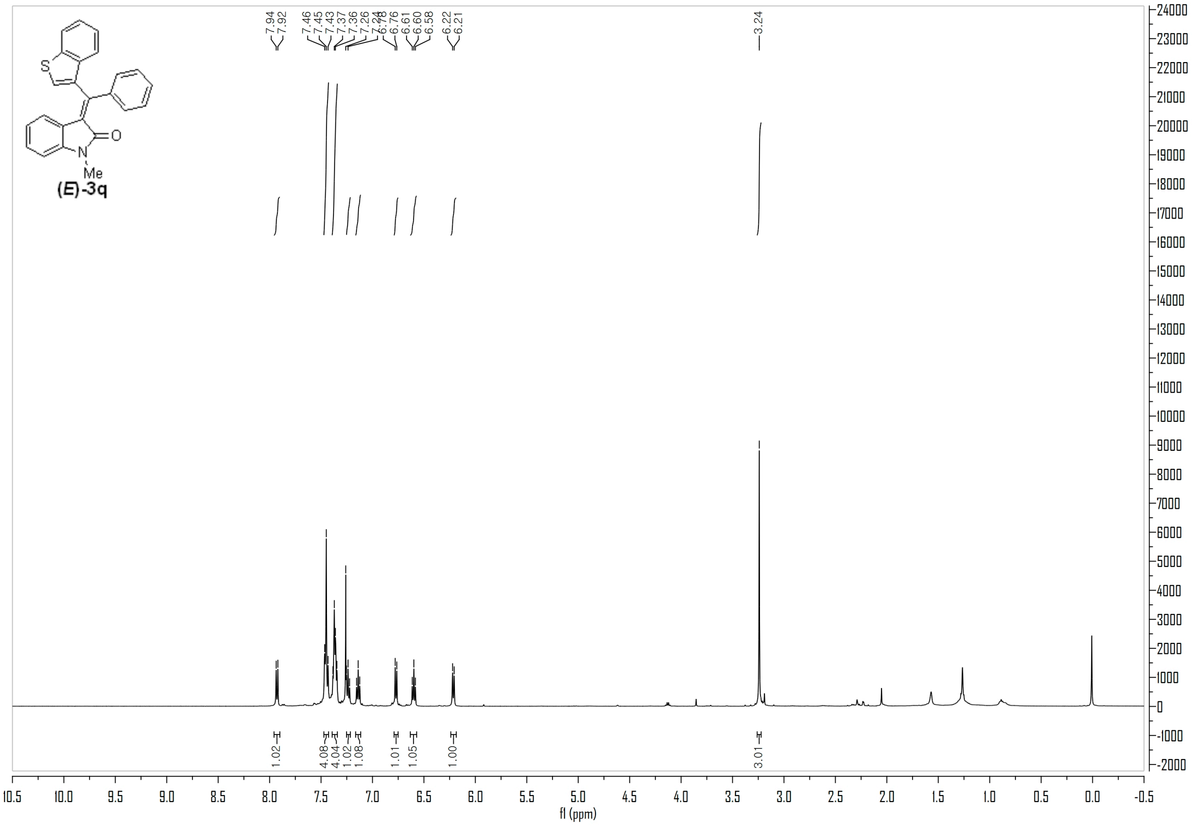

^{13}C NMR spectrum of (*E*)-3q

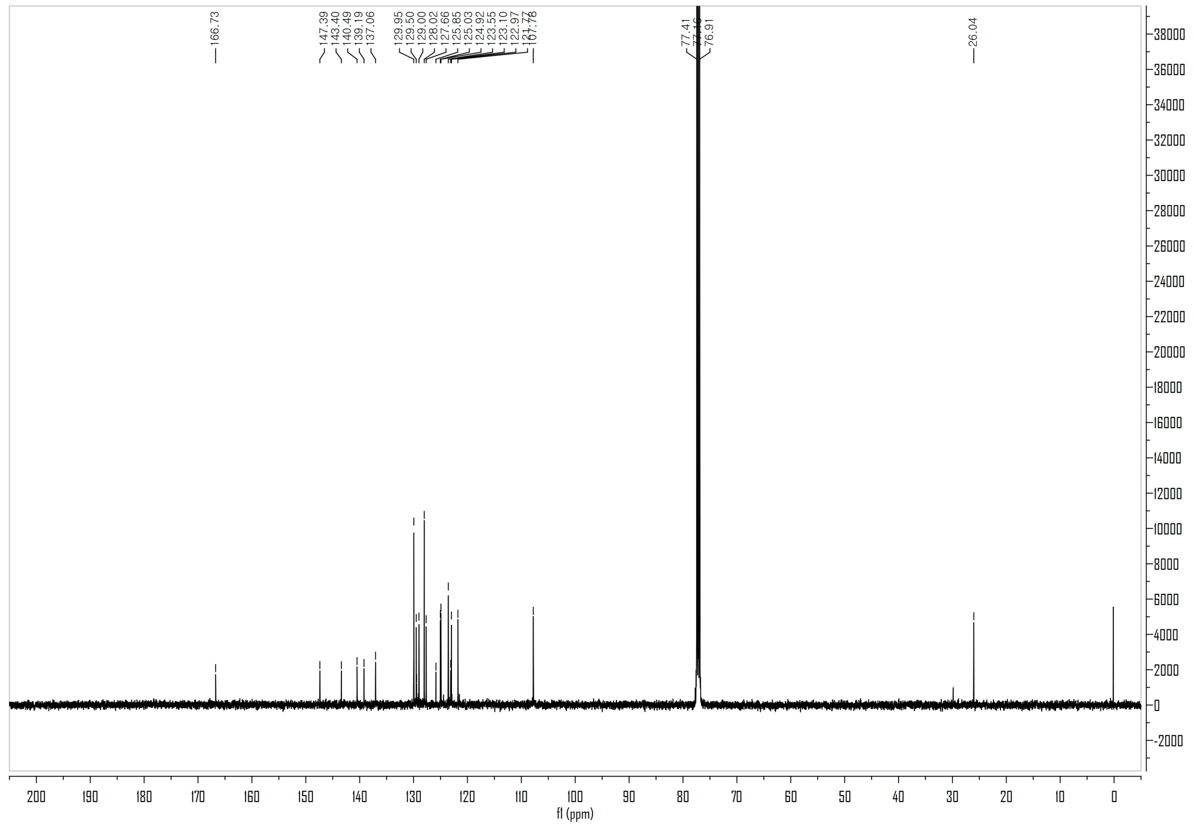

HSQC spectrum of (*E*)-3q

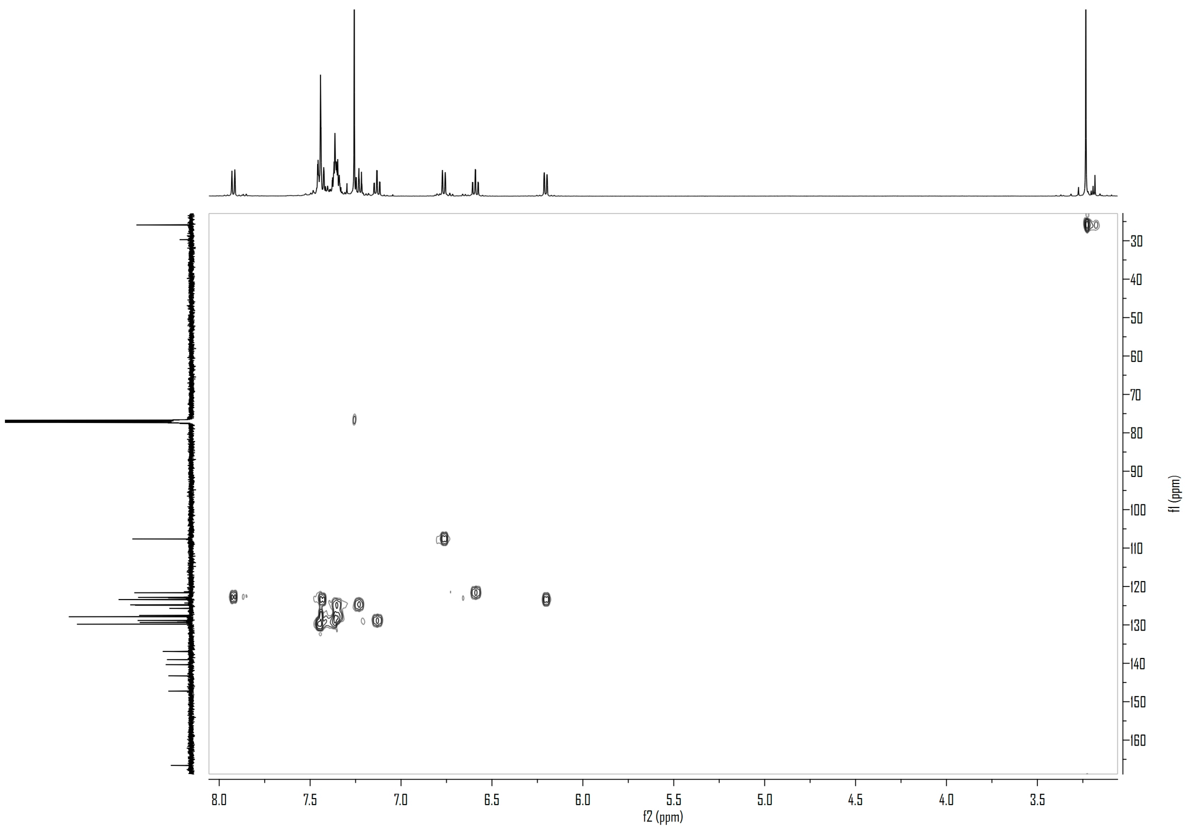

HMBC spectrum of (*E*)-3q

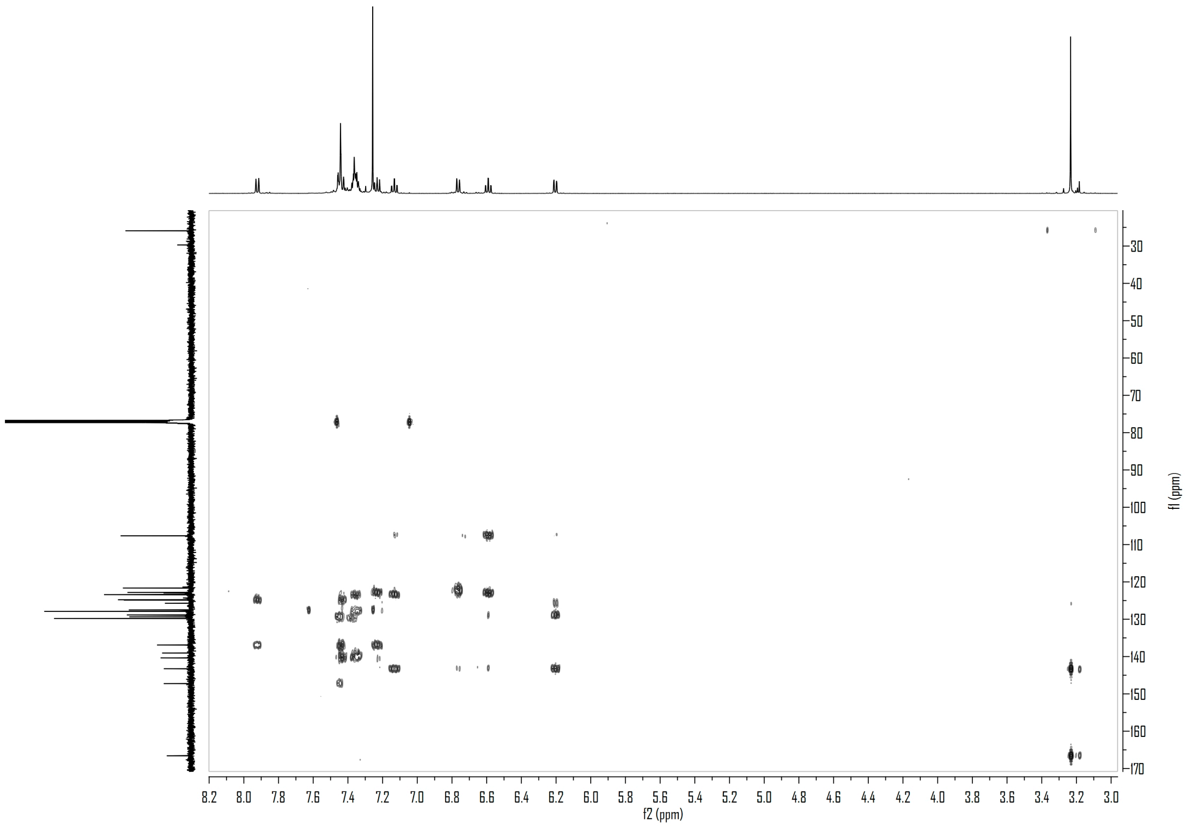

COSY spectrum of (*E*)-3q

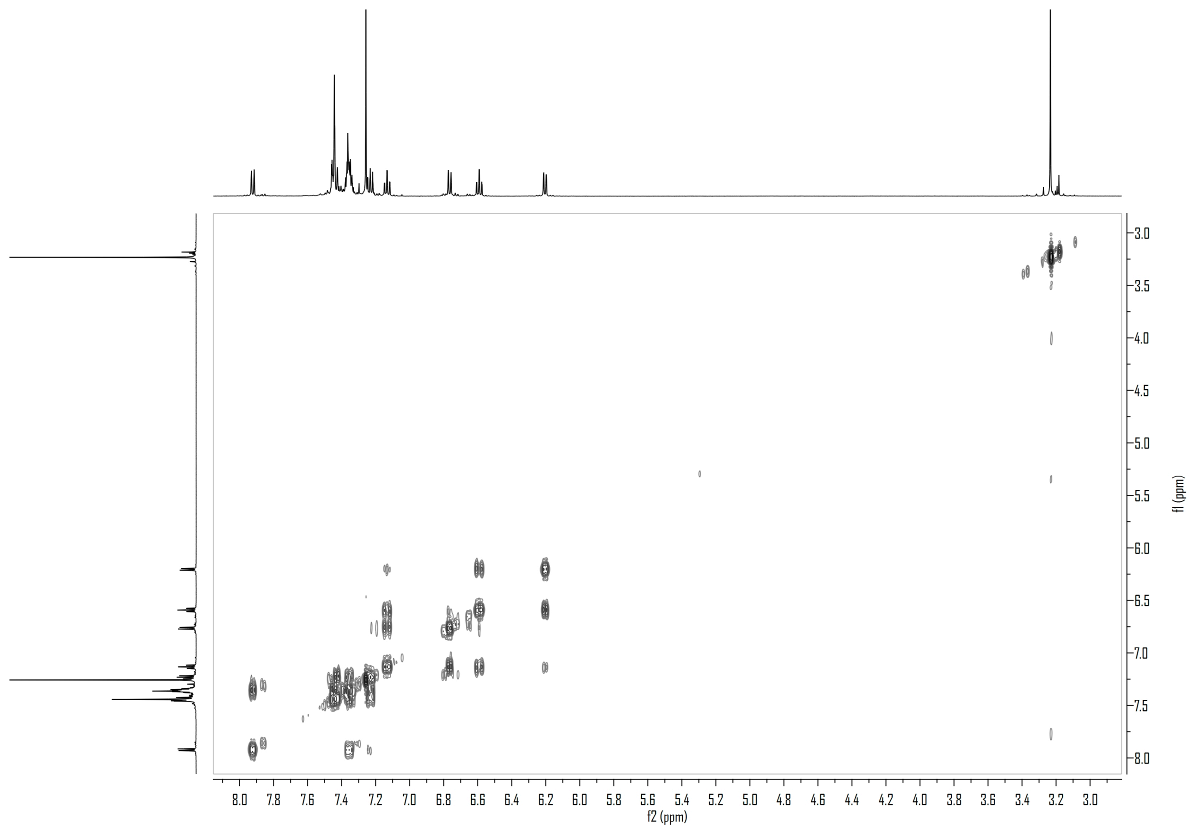

ROESY spectrum of (*E*)-3q

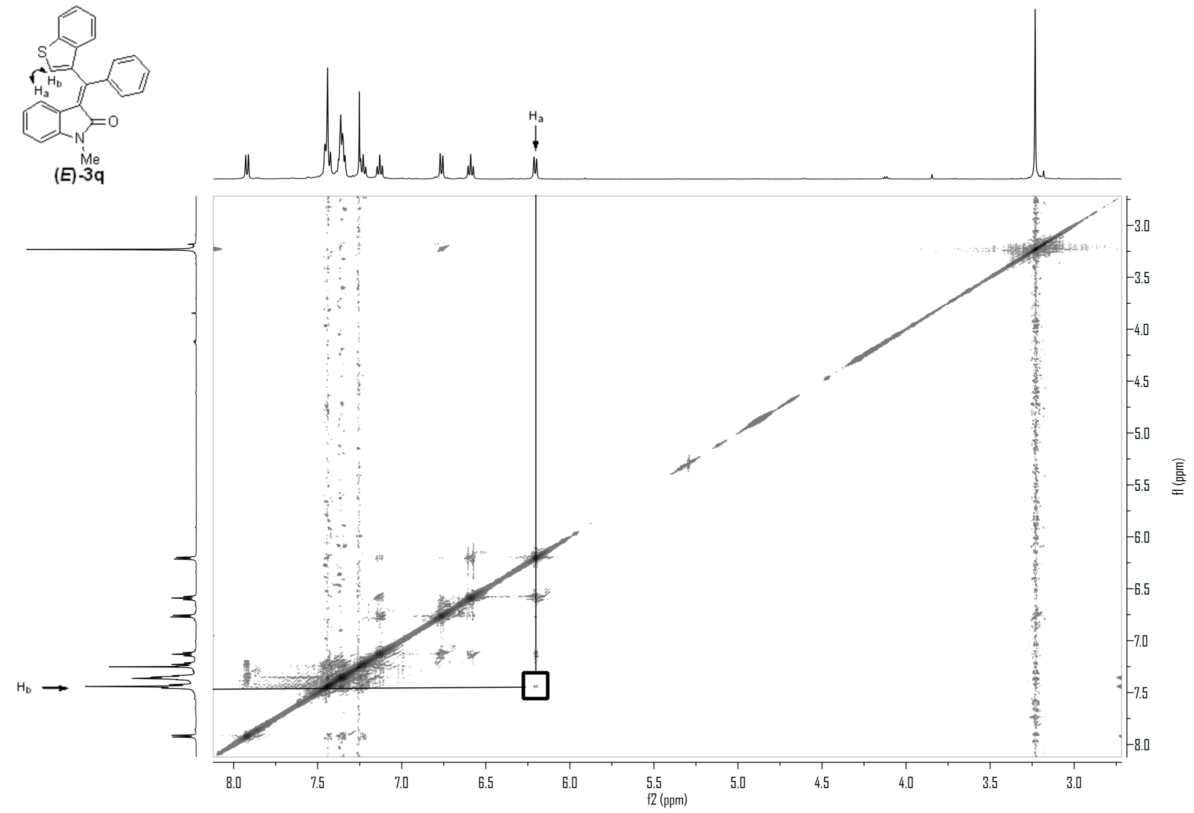

(*Z*)-3-(benzo[b]thiophen-3-yl(phenyl)methylene)-1-methylindolin-2-one ((*Z*)-3q)

^{1}H NMR spectrum of (*Z*)-3q

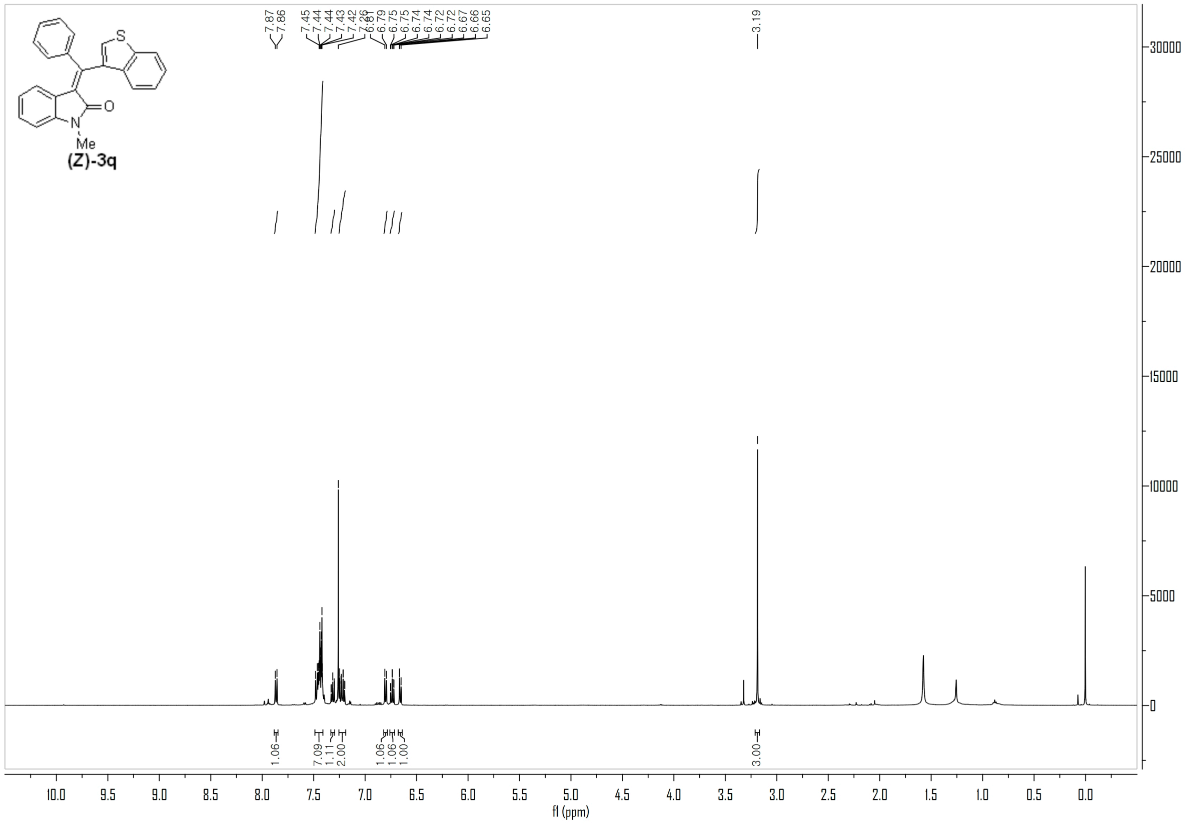

^{13}C NMR spectrum of (*Z*)-3q

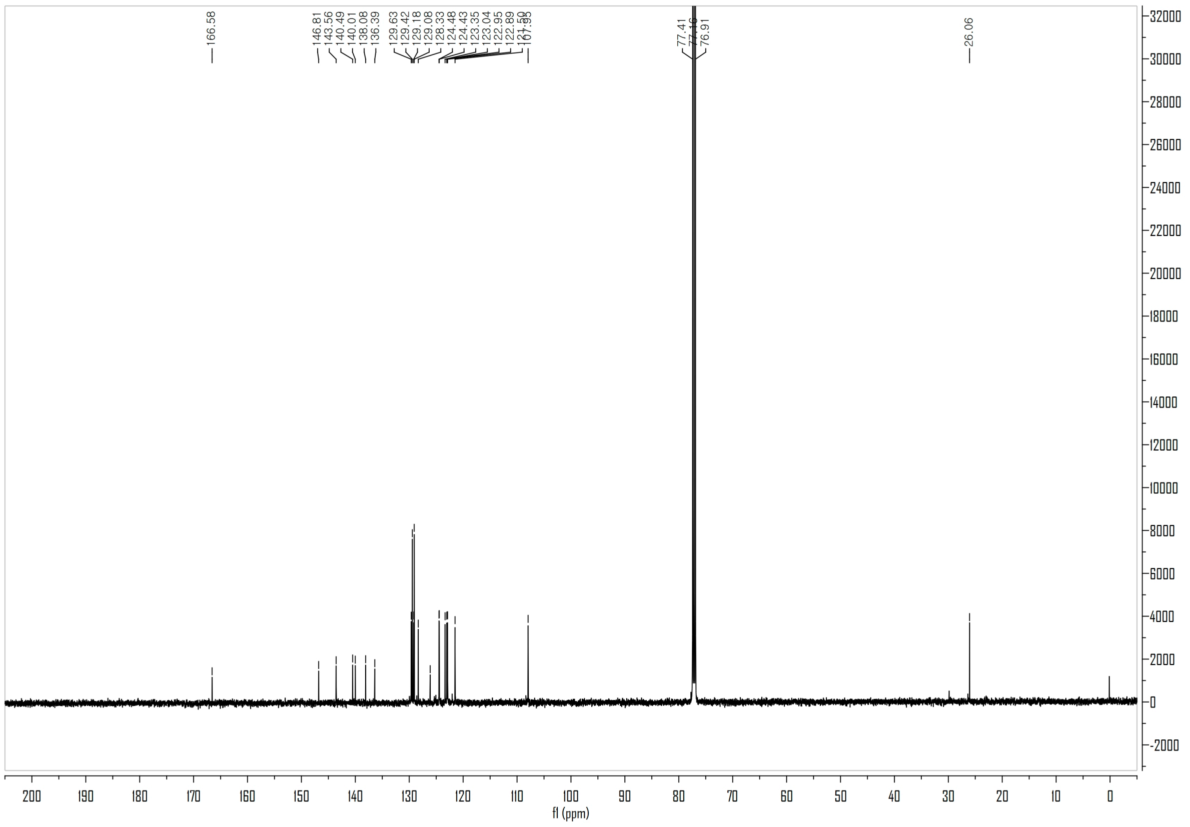

HSQC spectrum of (*Z*)-3q

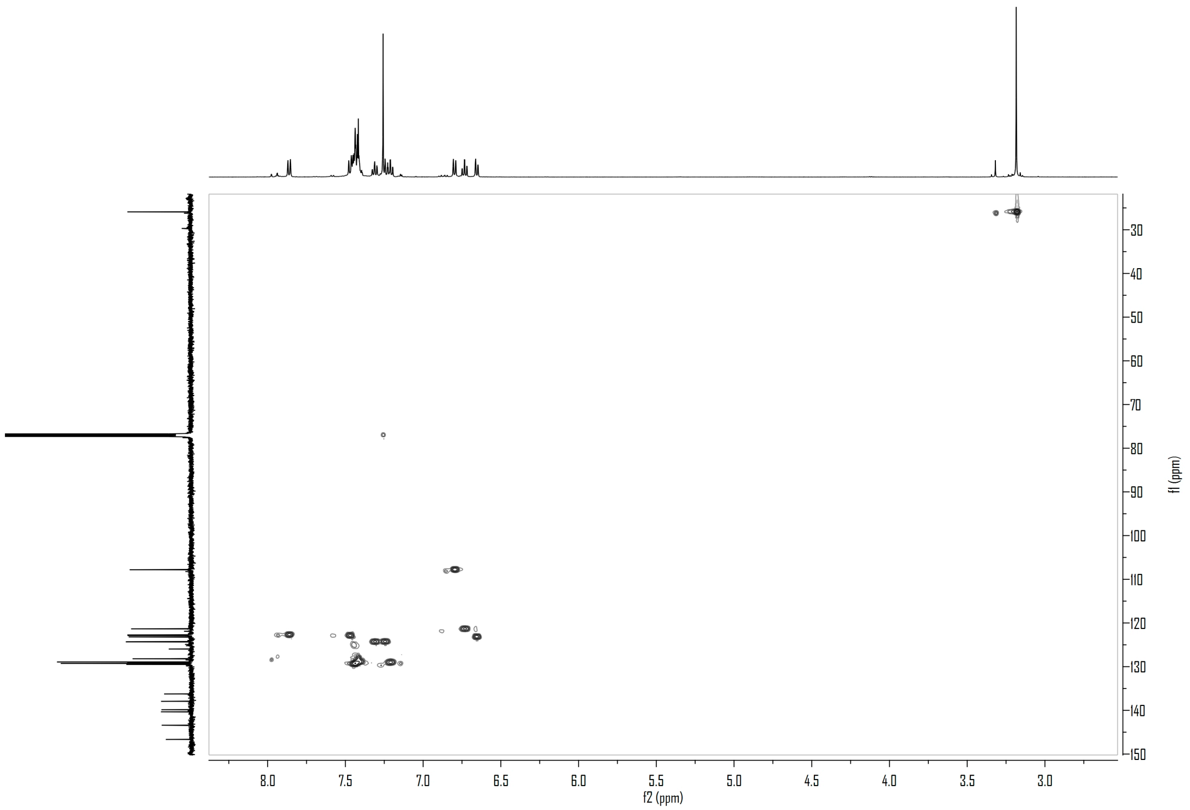

HMBC spectrum of (*Z*)-3q

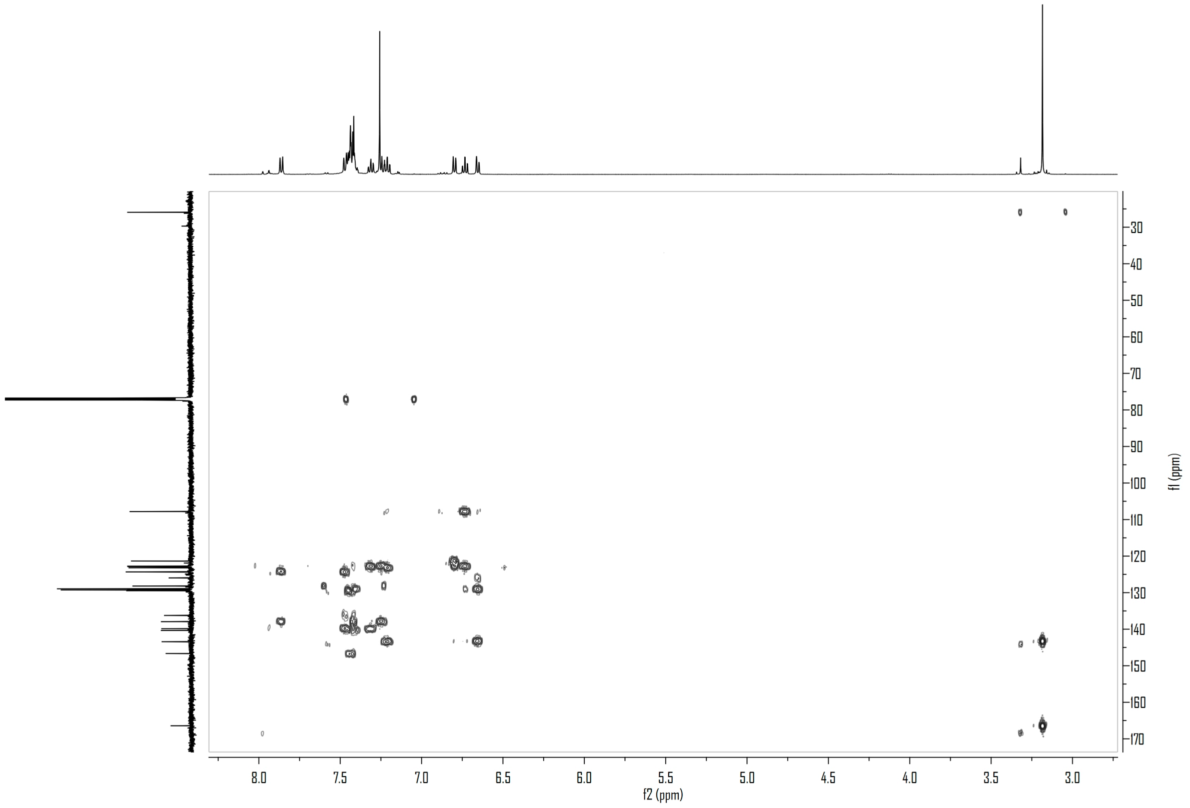

COSY spectrum of (*Z*)-3q

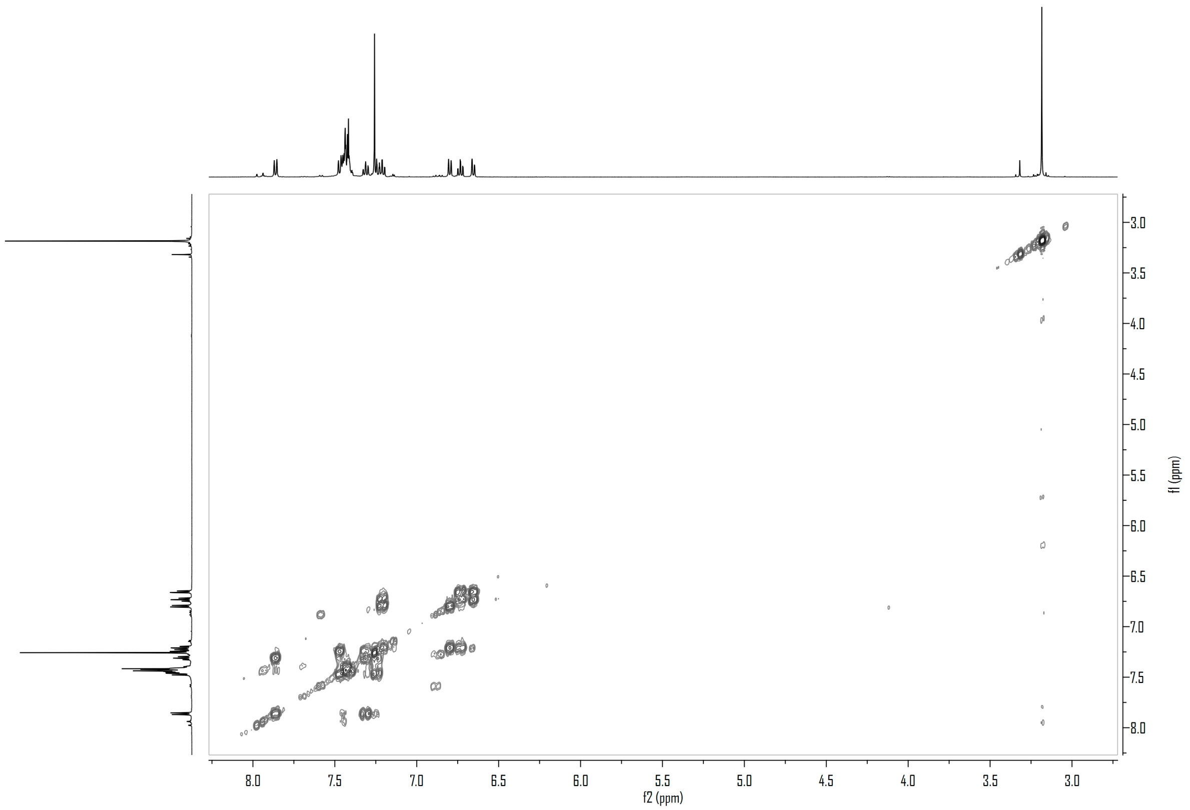

ROESY spectrum of (*Z*)-3q

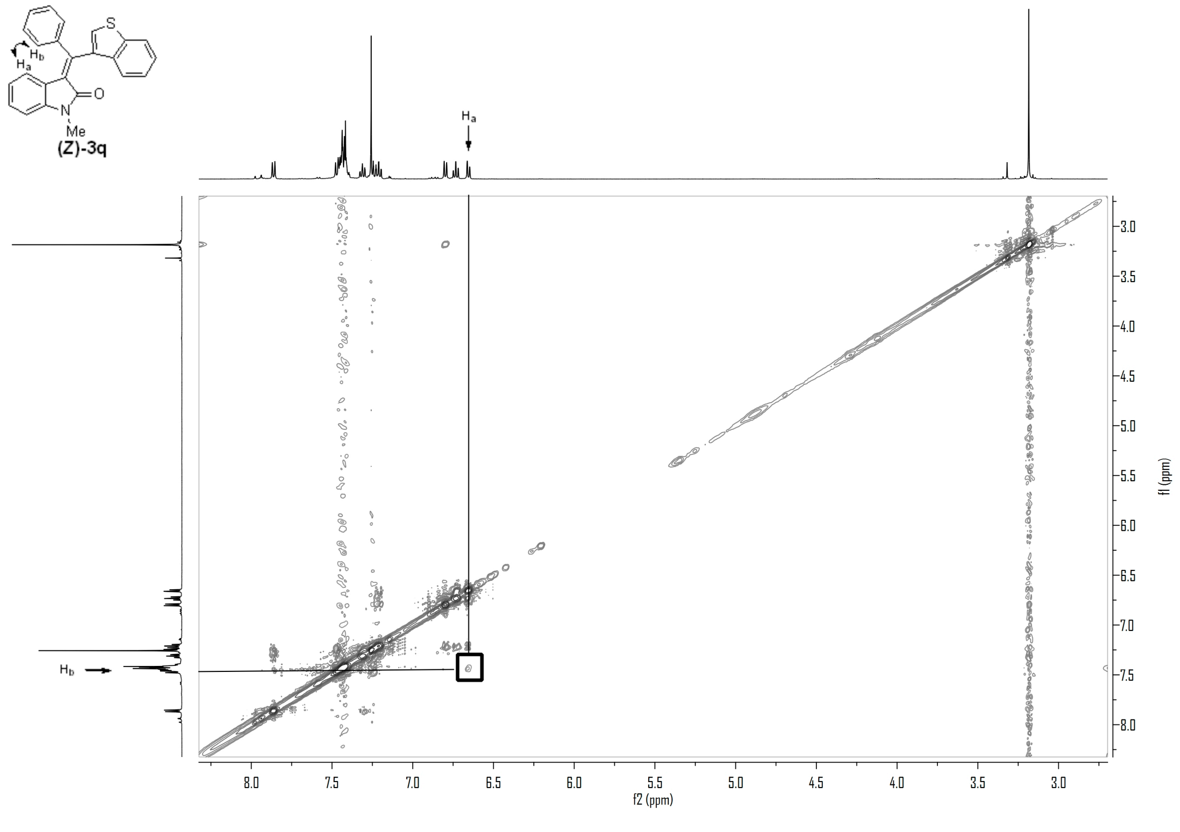

(*E*)-3-(benzo[b]thiophen-2-yl(phenyl)methylene)-1-methylindolin-2-one ((*E*)-3r)

^{1}H NMR spectrum of (*E*)-3r

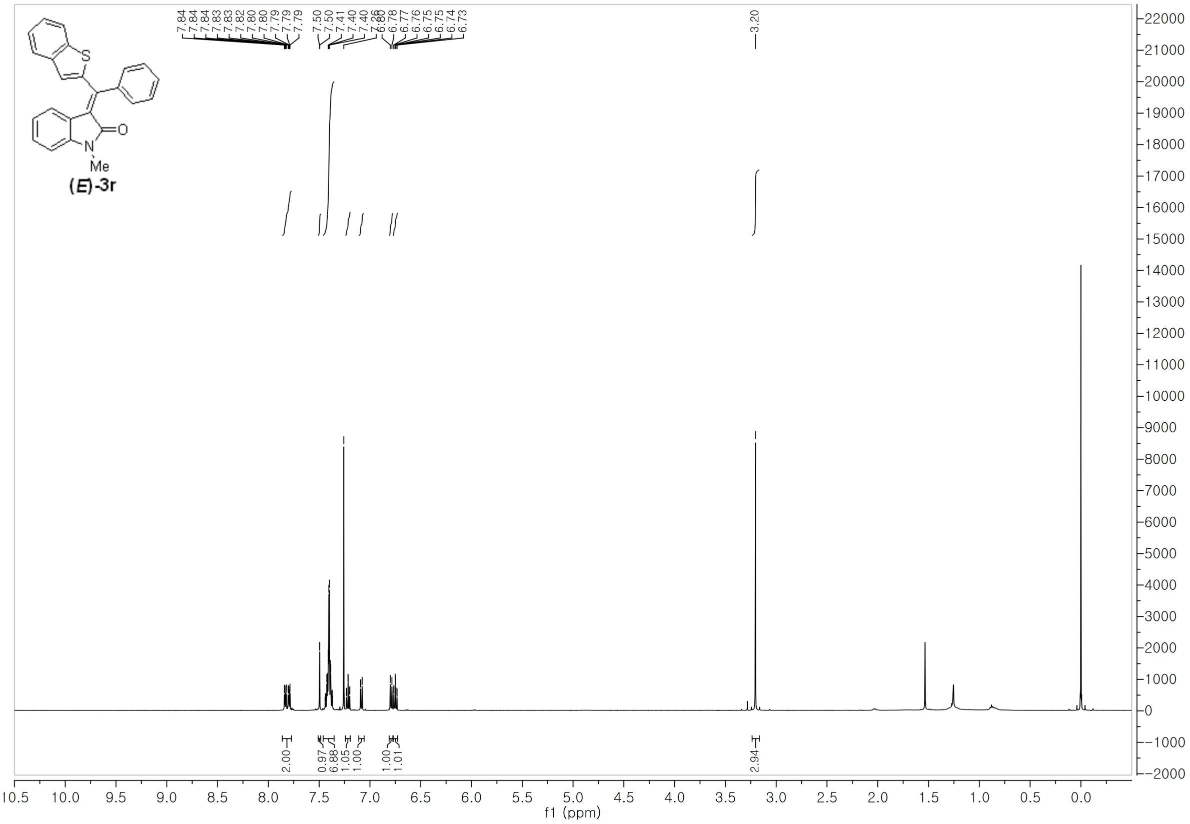

^{13}C NMR spectrum of (*E*)-3r

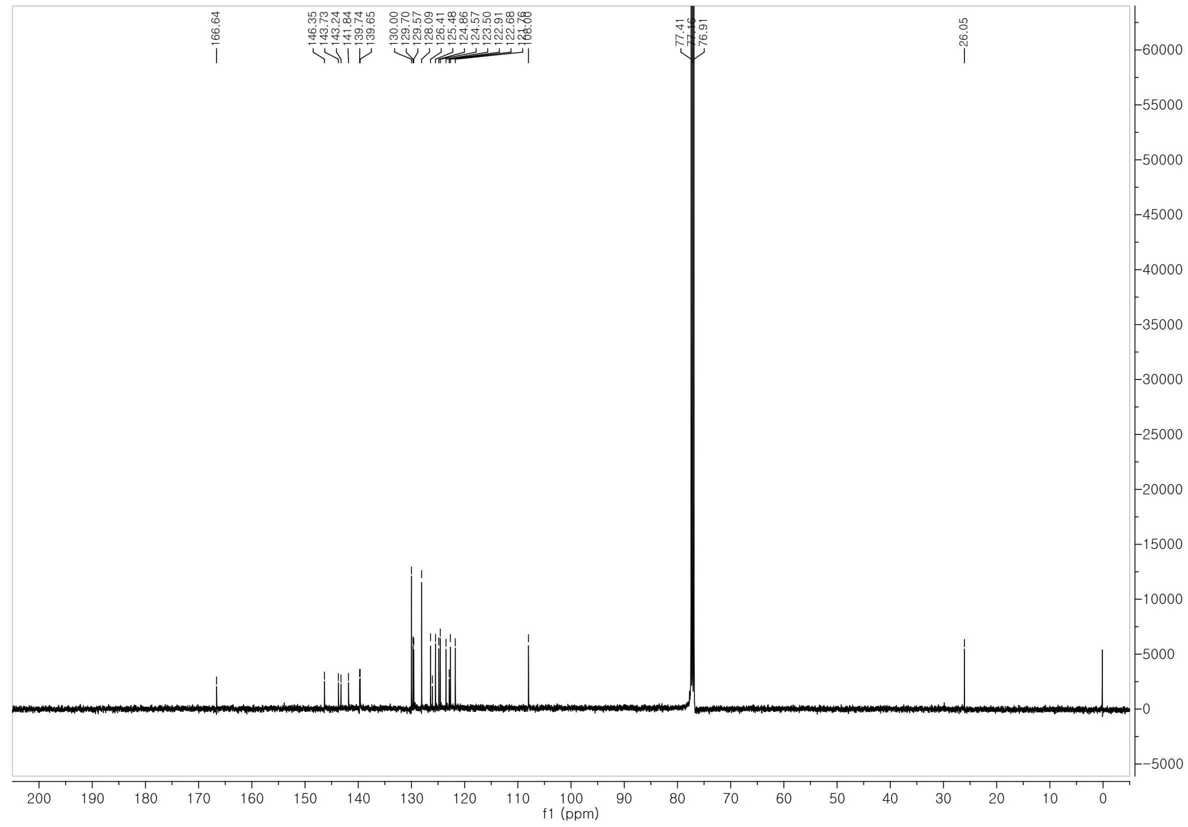

HSQC spectrum of (*E*)-3r

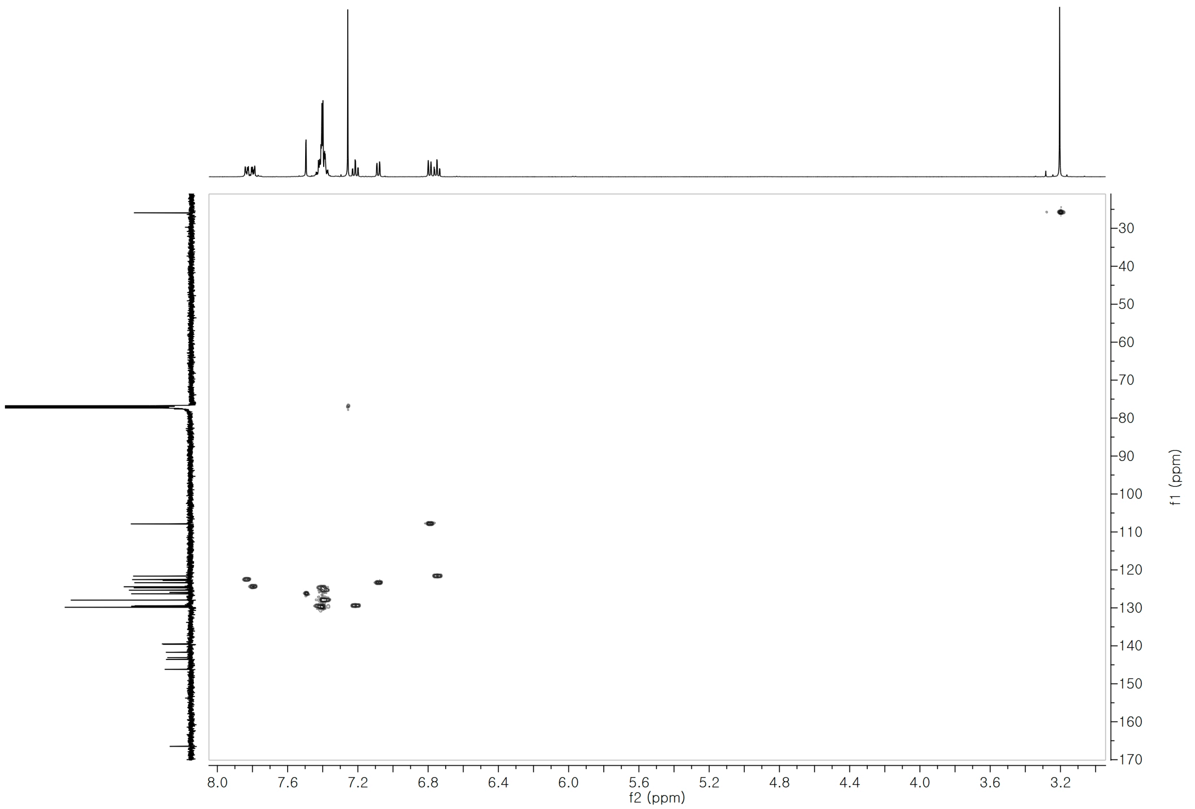

HMBC spectrum of (*E*)-3r

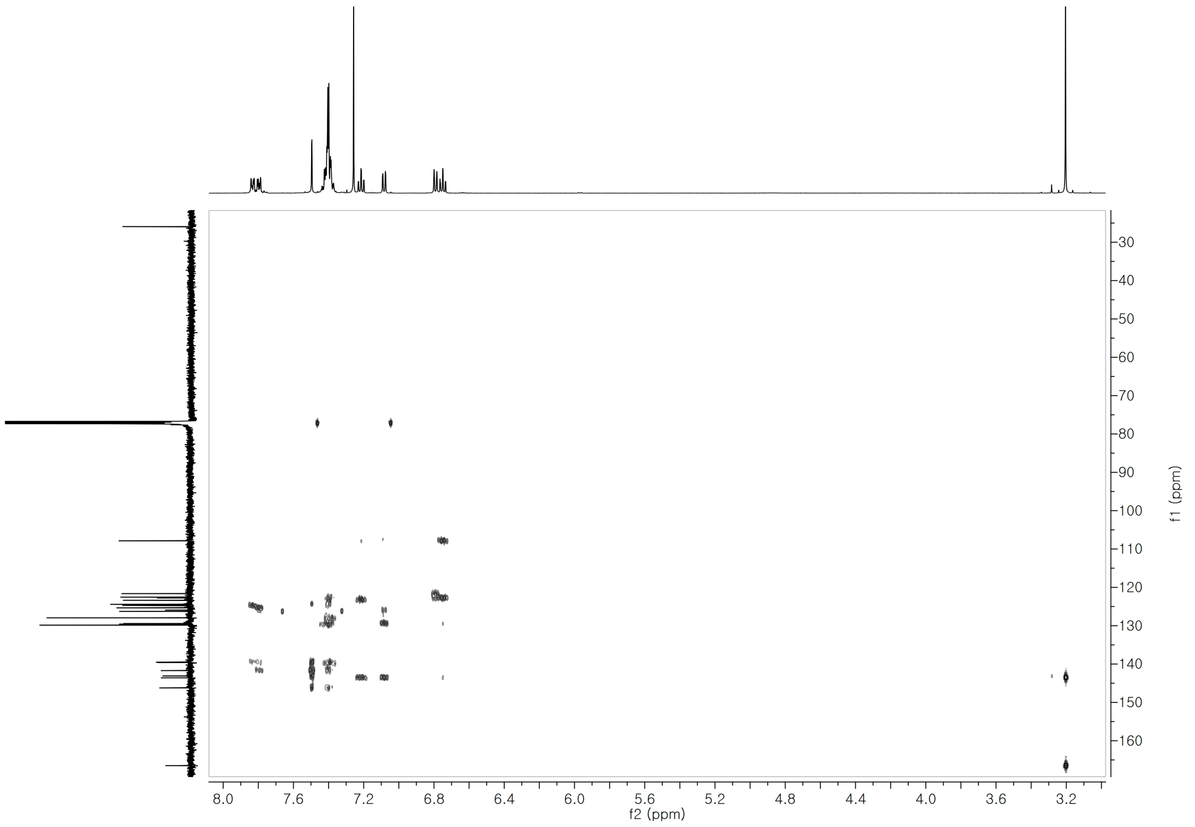

COSY spectrum of (*E*)-3r

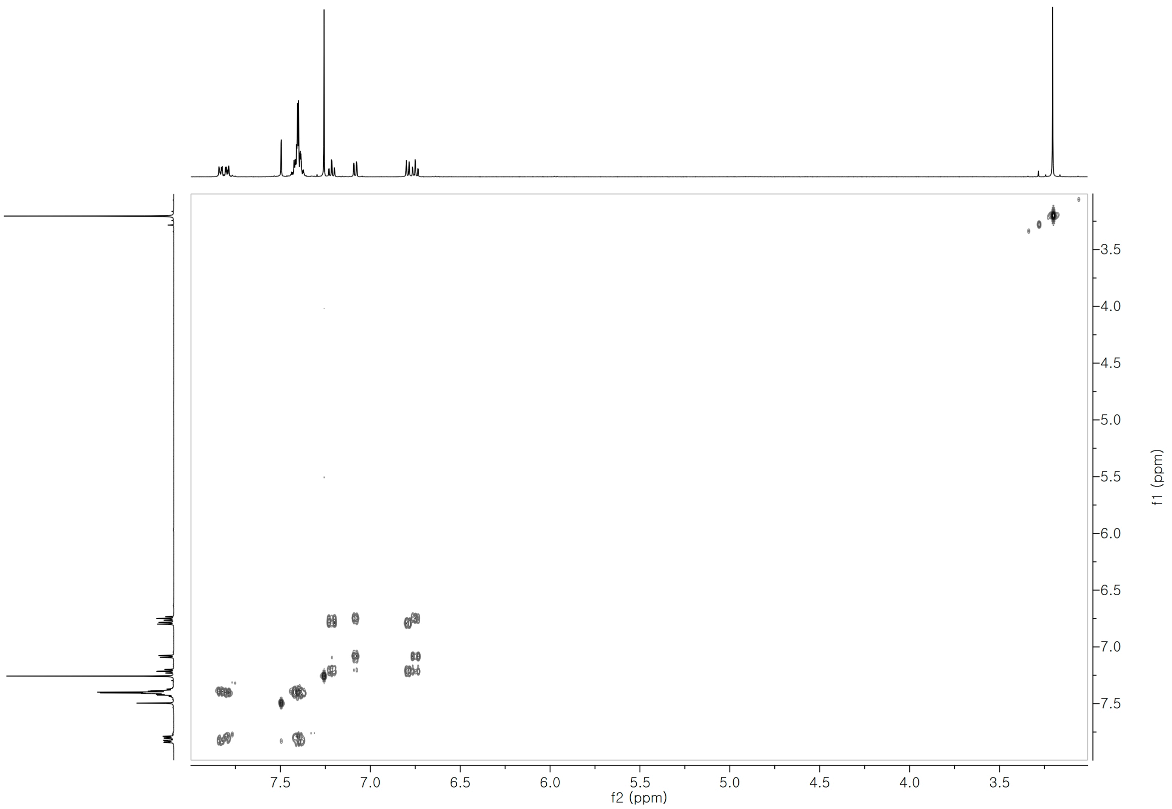

ROESY spectrum of (*E*)-3r

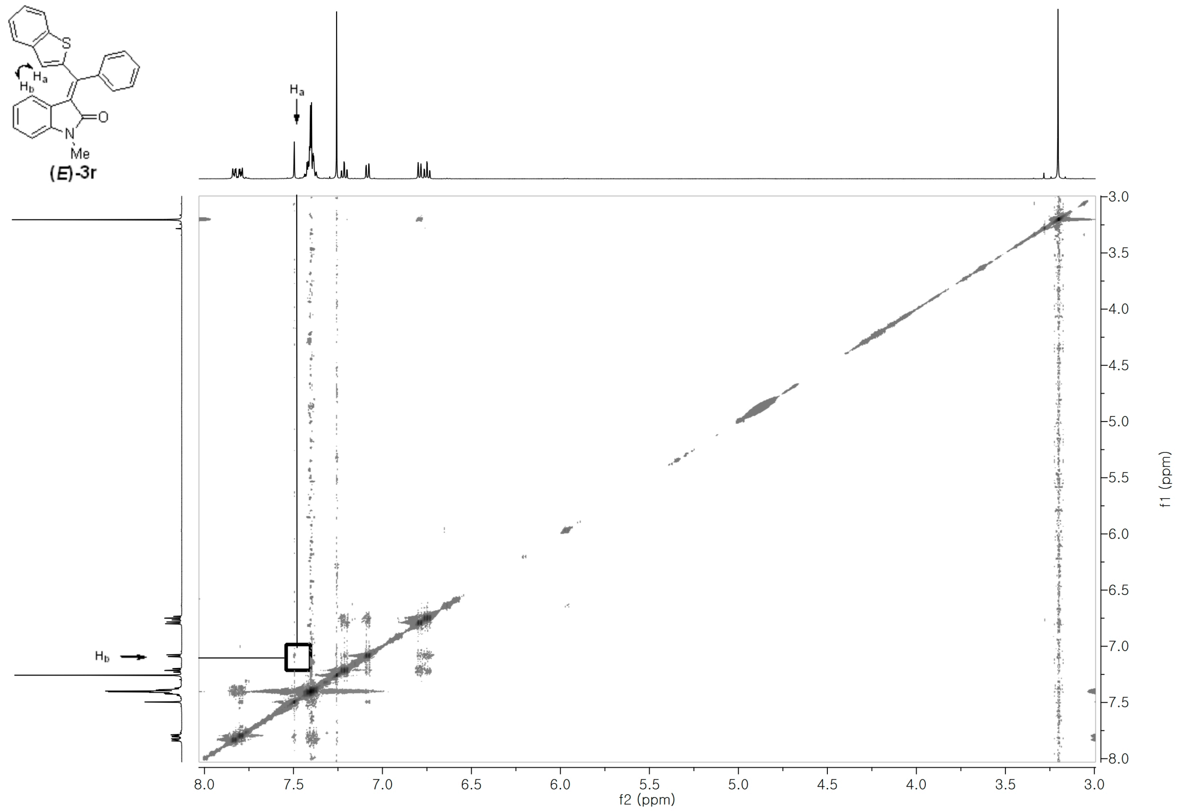

(*Z*)-3-(benzo[b]thiophen-2-yl(phenyl)methylene)-1-methylindolin-2-one ((*Z*)-3r)

^{1}H NMR spectrum of (*Z*)-3r

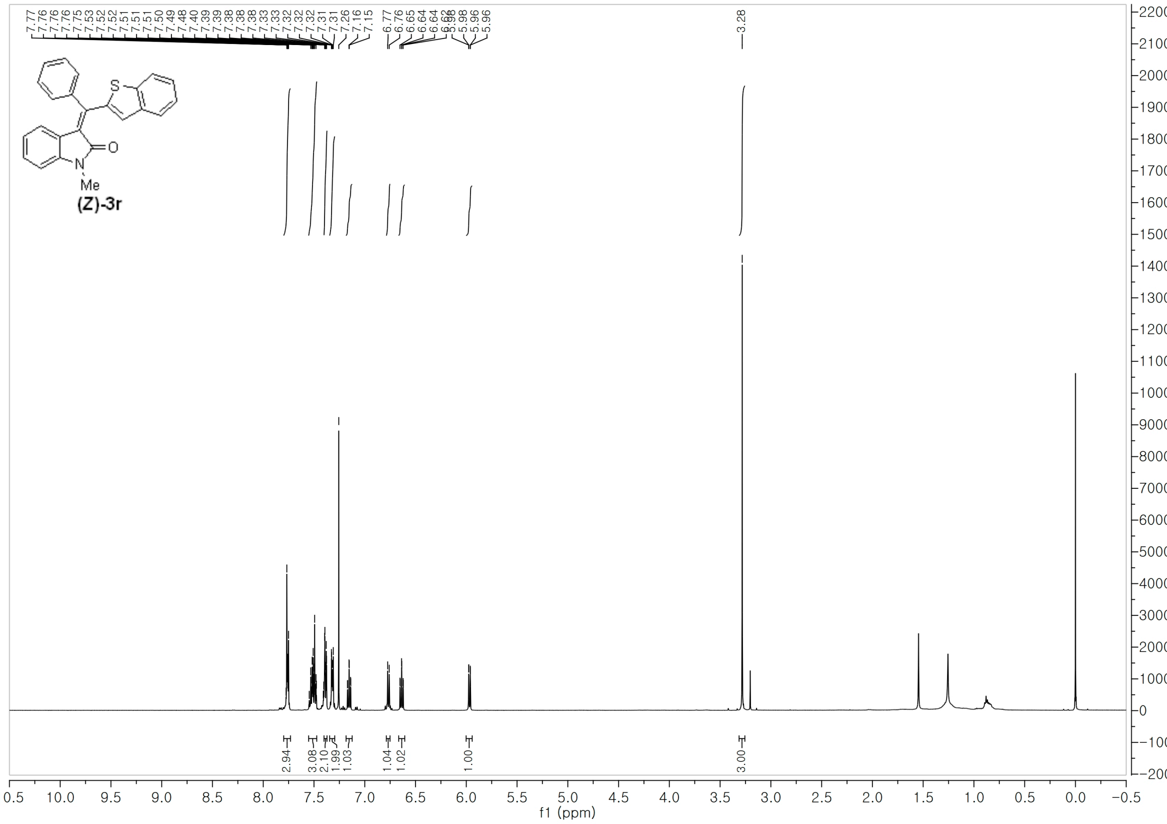

^{13}C NMR spectrum of (*Z*)-3r

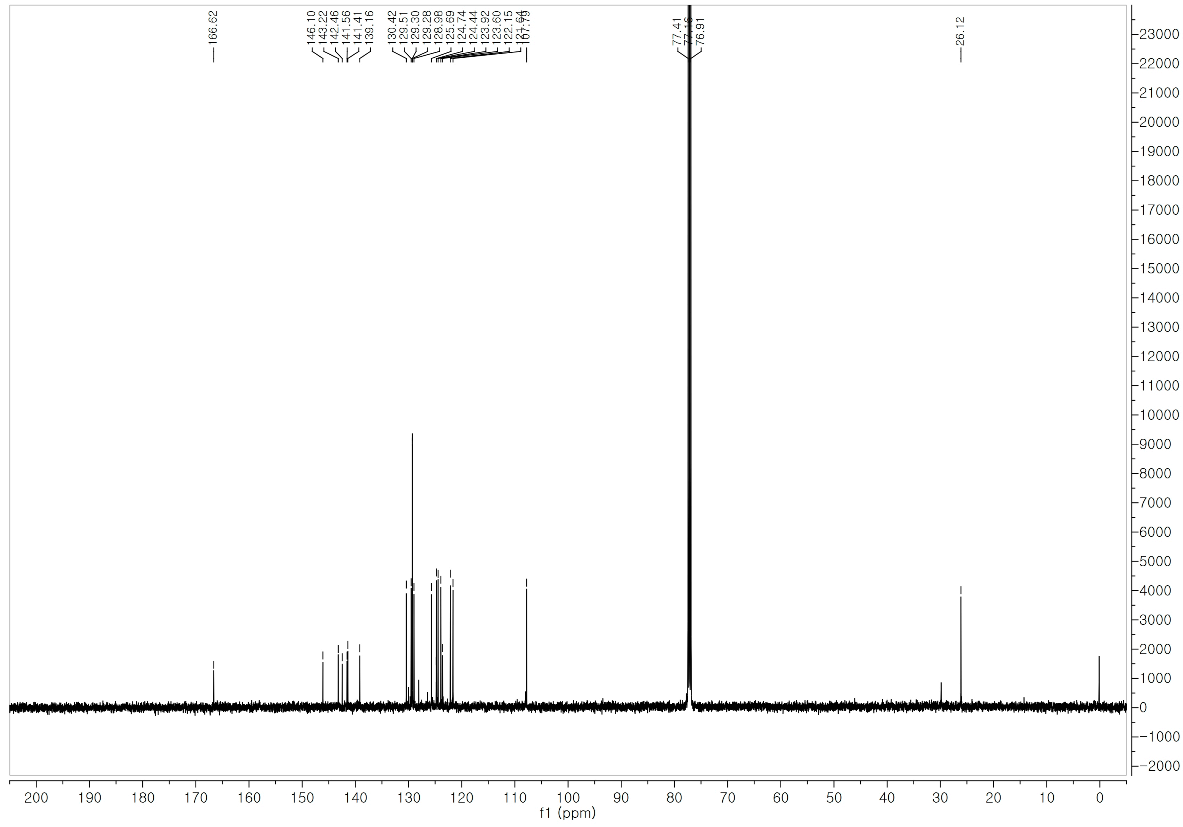

HSQC spectrum of (*Z*)-3r

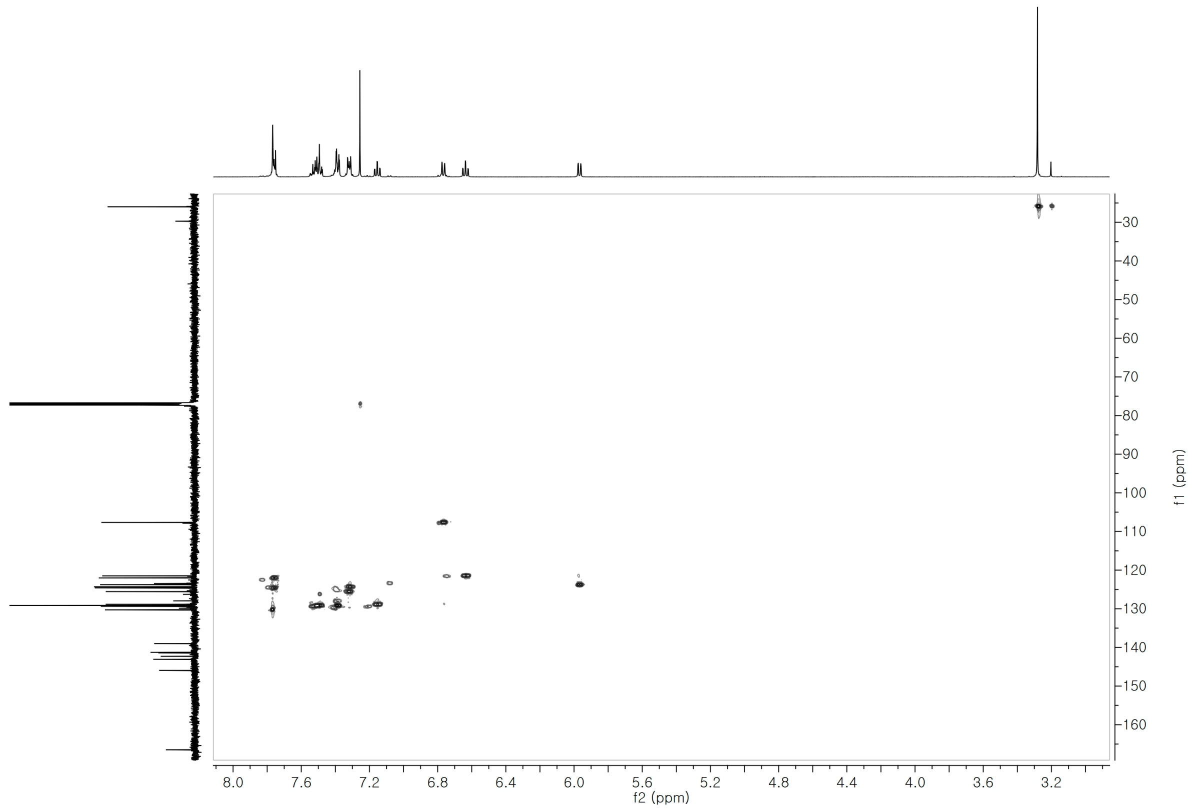

HMBC spectrum of (*Z*)-3r

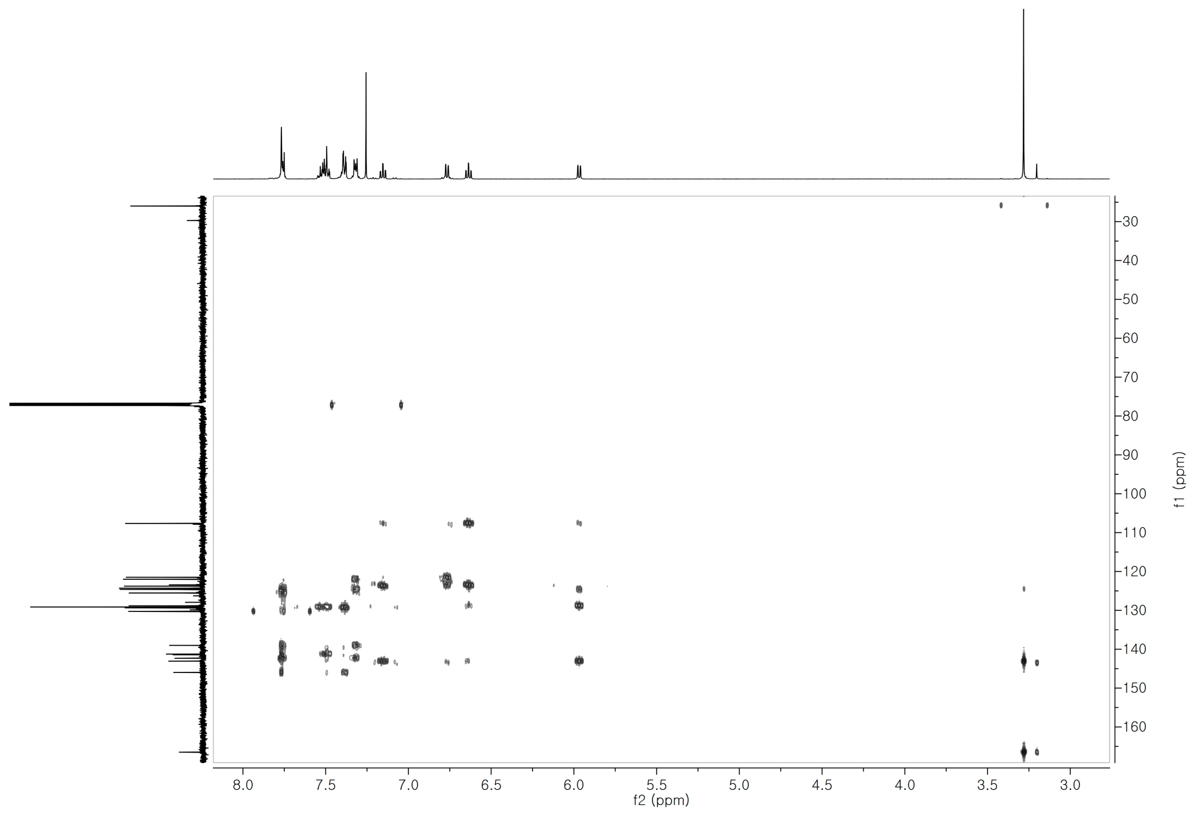

COSY spectrum of (*Z*)-3r

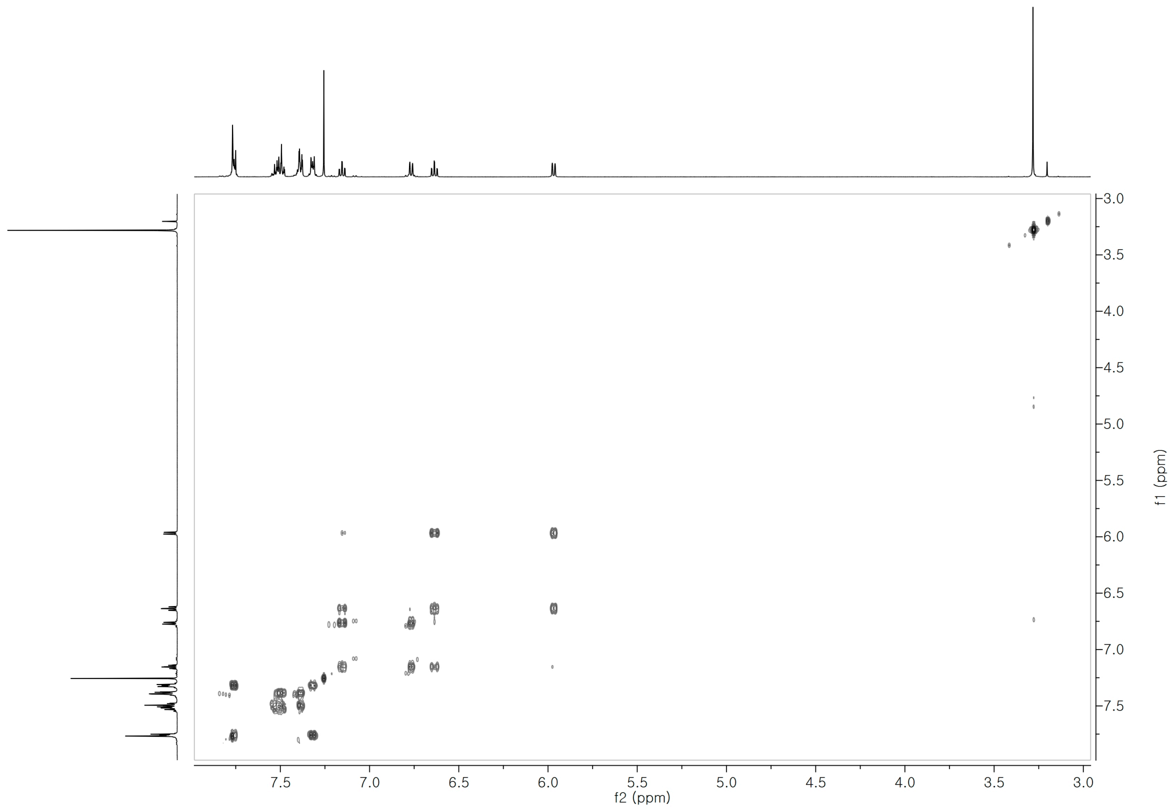

ROESY spectrum of (*Z*)-3r

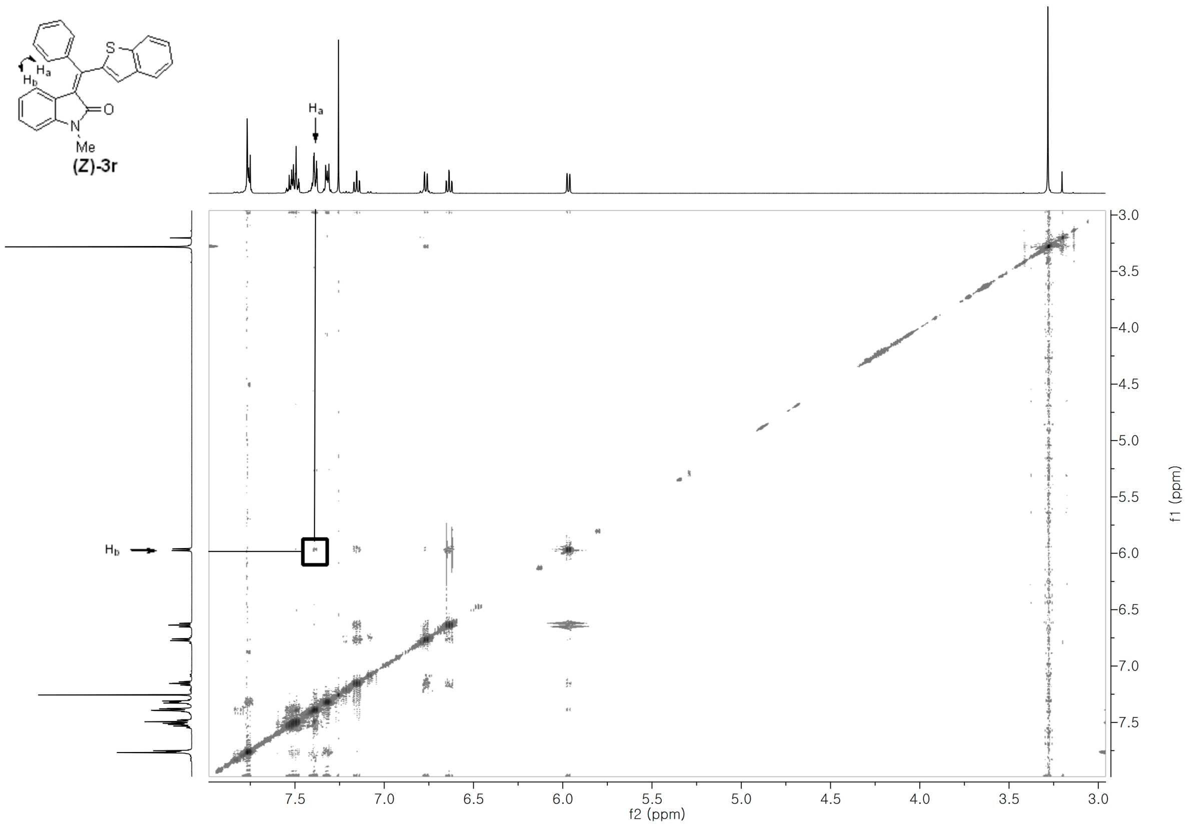

(*Z*)-3-(furan-3-yl(phenyl)methylene)-1-methylindolin-2-one ((*Z*)-3s)

^{1}H NMR spectrum of (*Z*)-3s

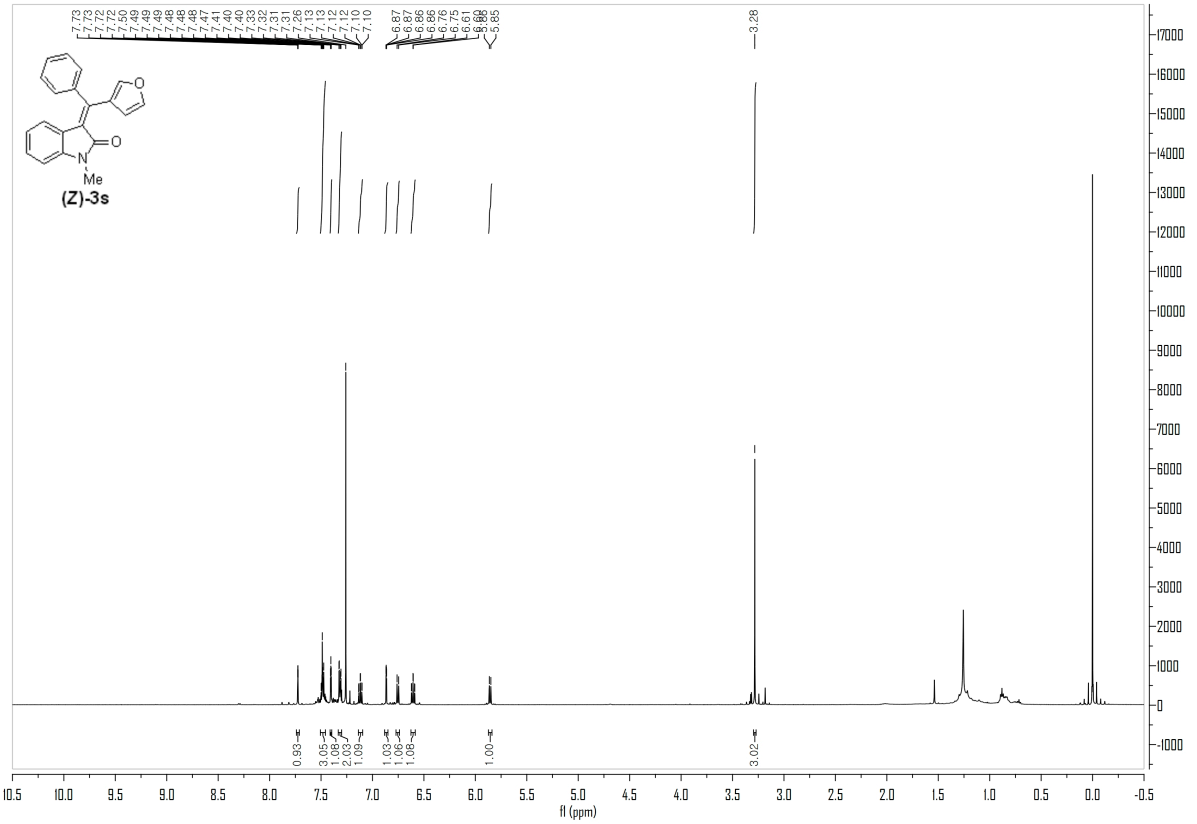

^{13}C NMR spectrum of (*Z*)-3s

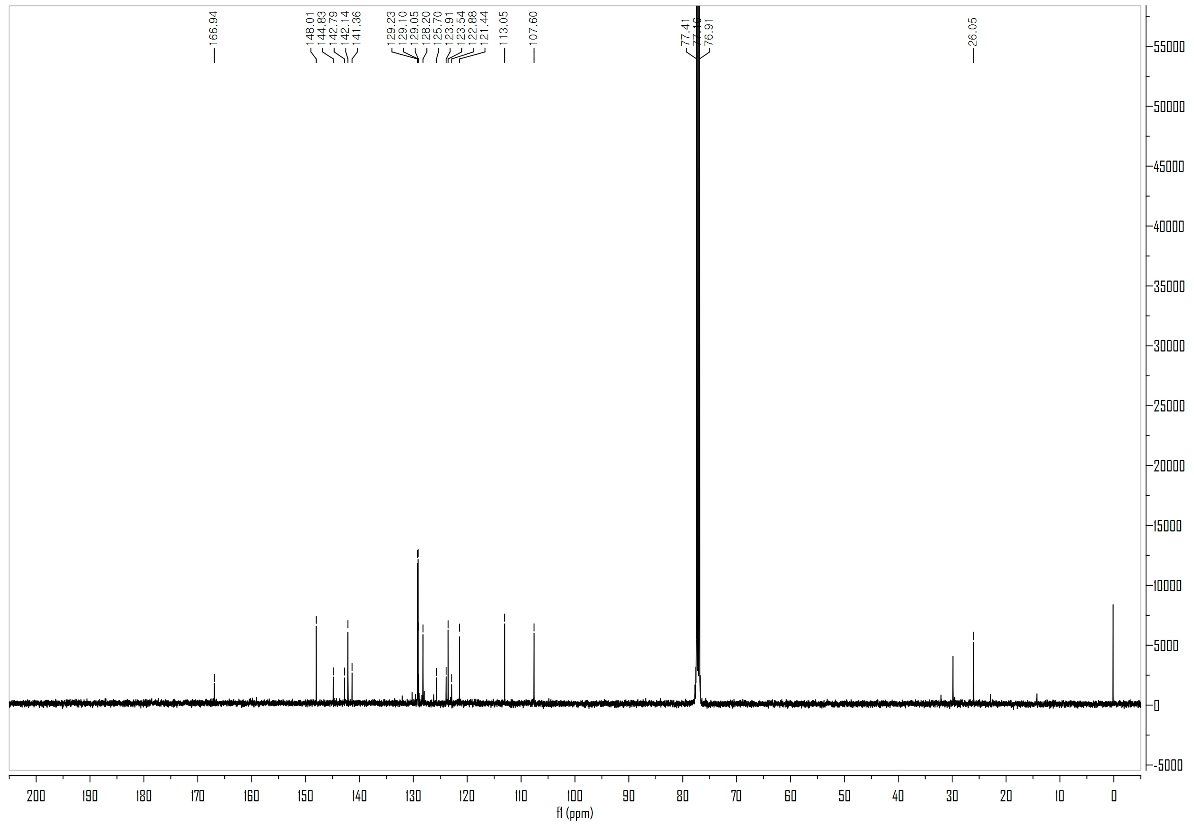

HSQC spectrum of (*Z*)-3s

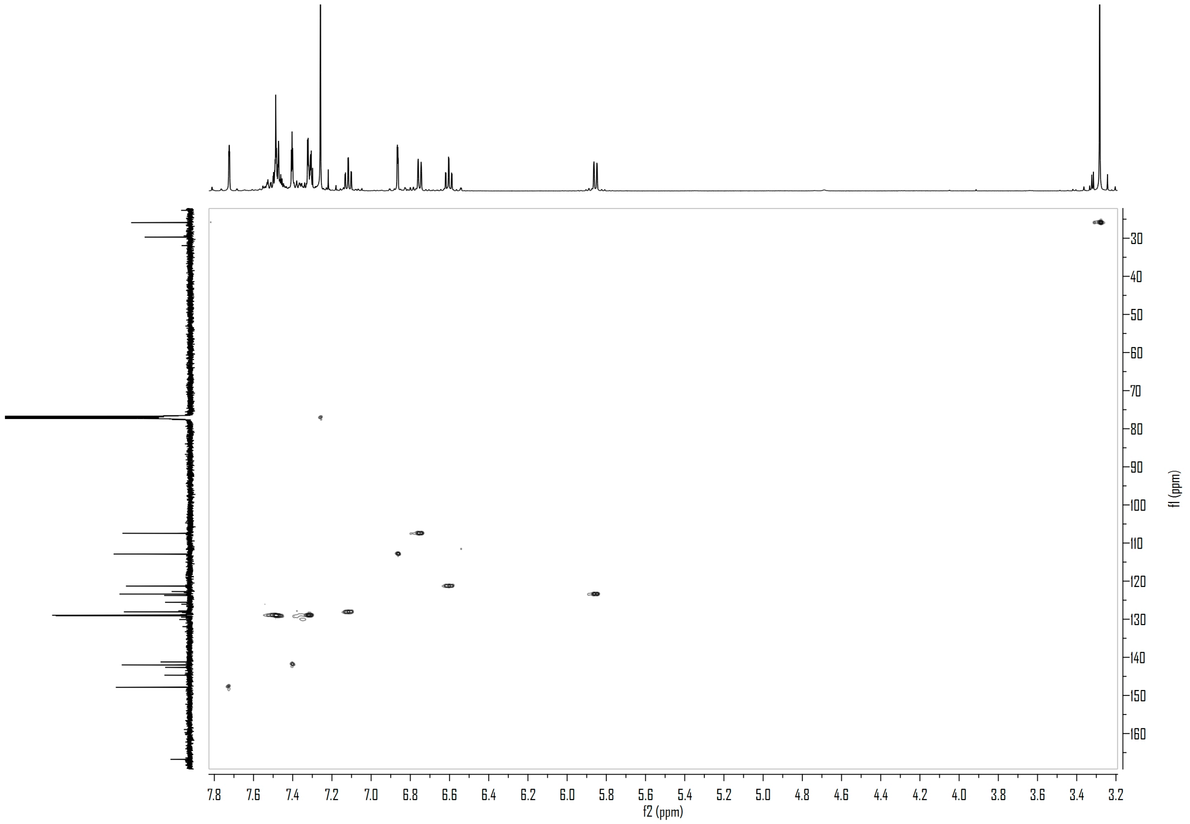

HMBC spectrum of (*Z*)-3s

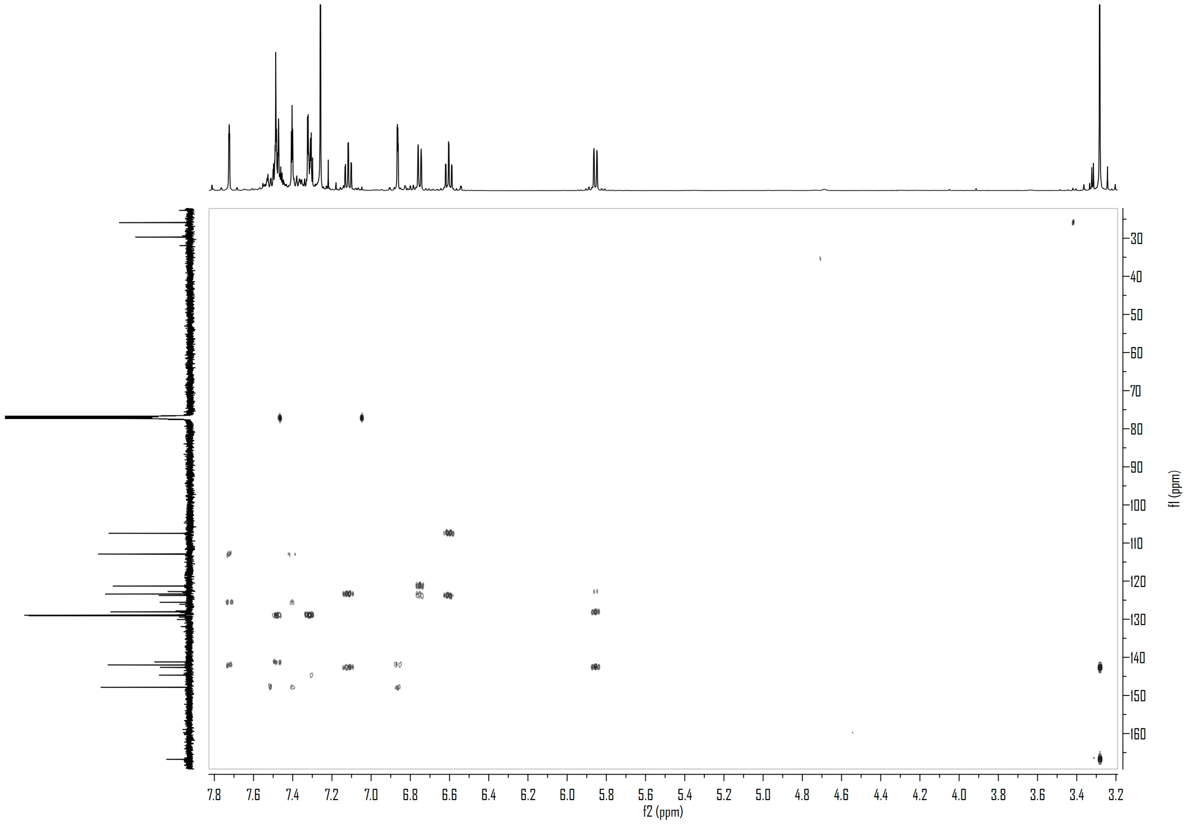

COSY spectrum of (*Z*)-3s

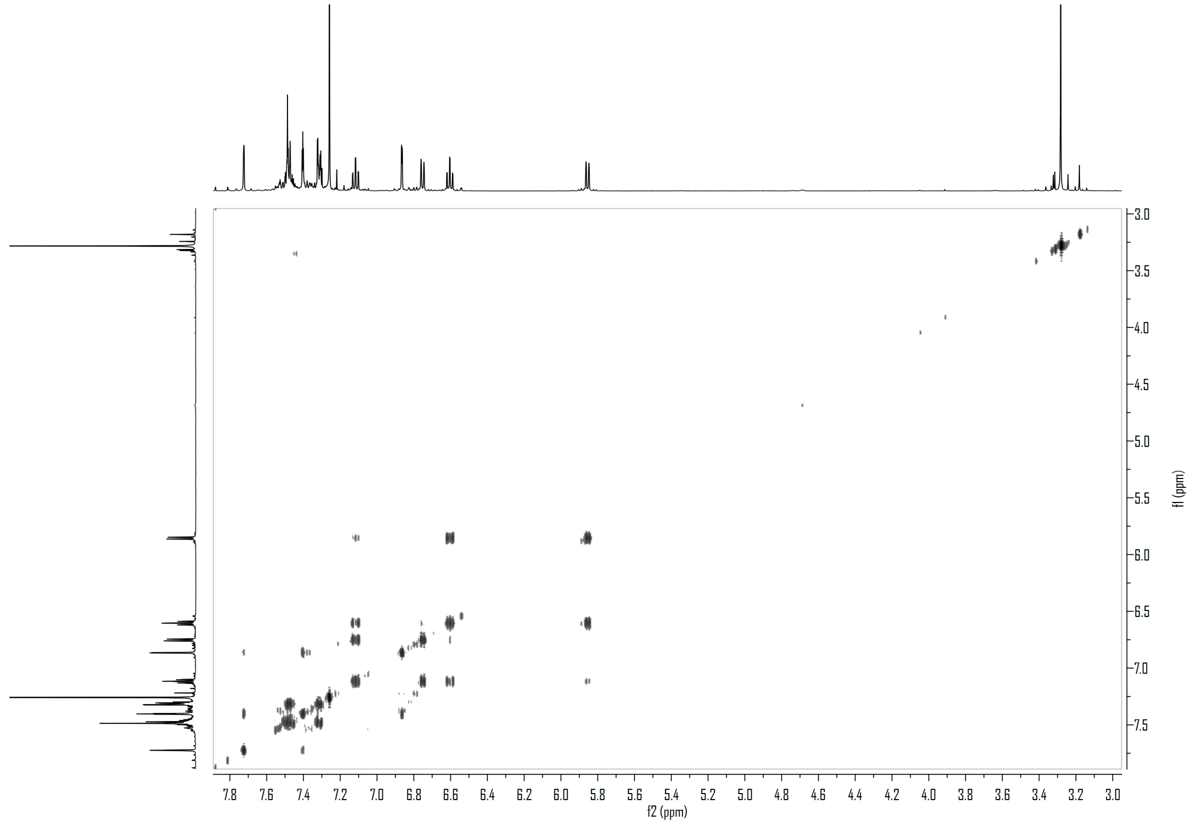

ROESY spectrum of (*Z*)-3s

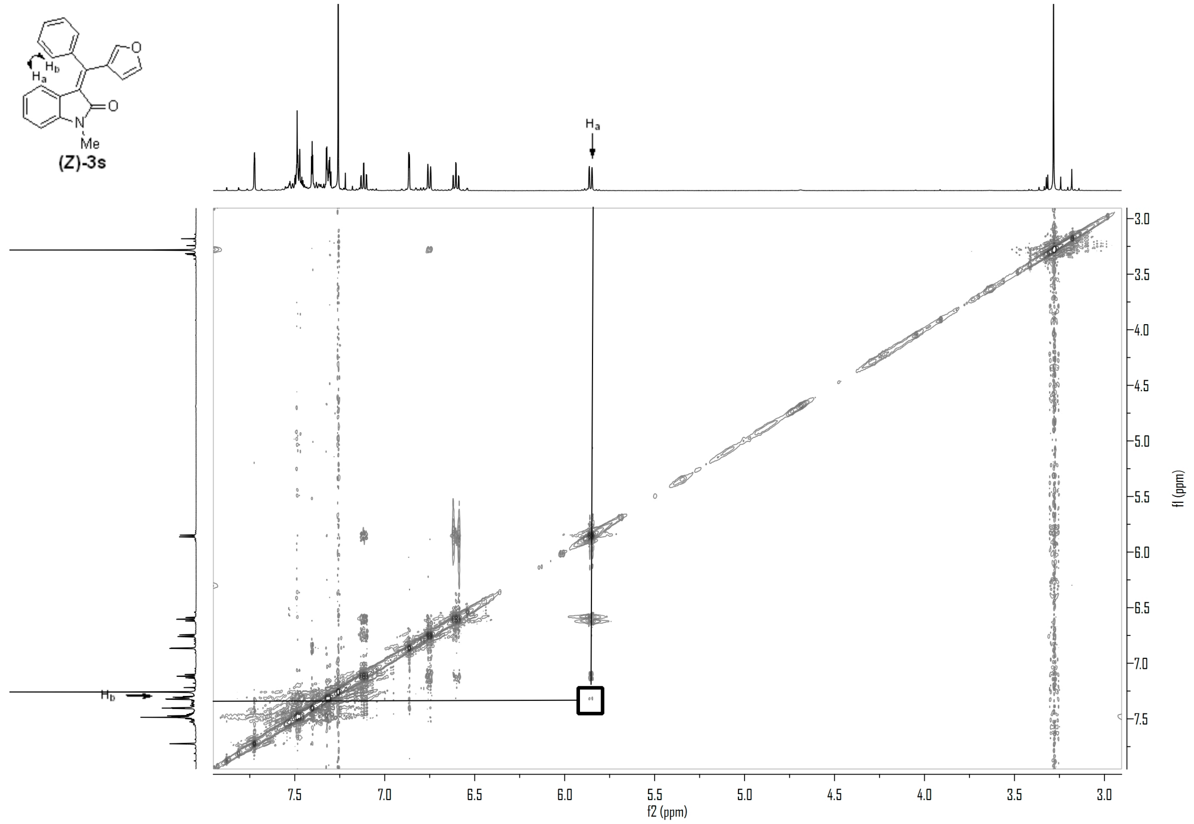

(*E*)-3-(furan-3-yl(phenyl)methylene)-1-methylindolin-2-one ((*E*)-3s)

^{1}H NMR spectrum of (*E*)-3s

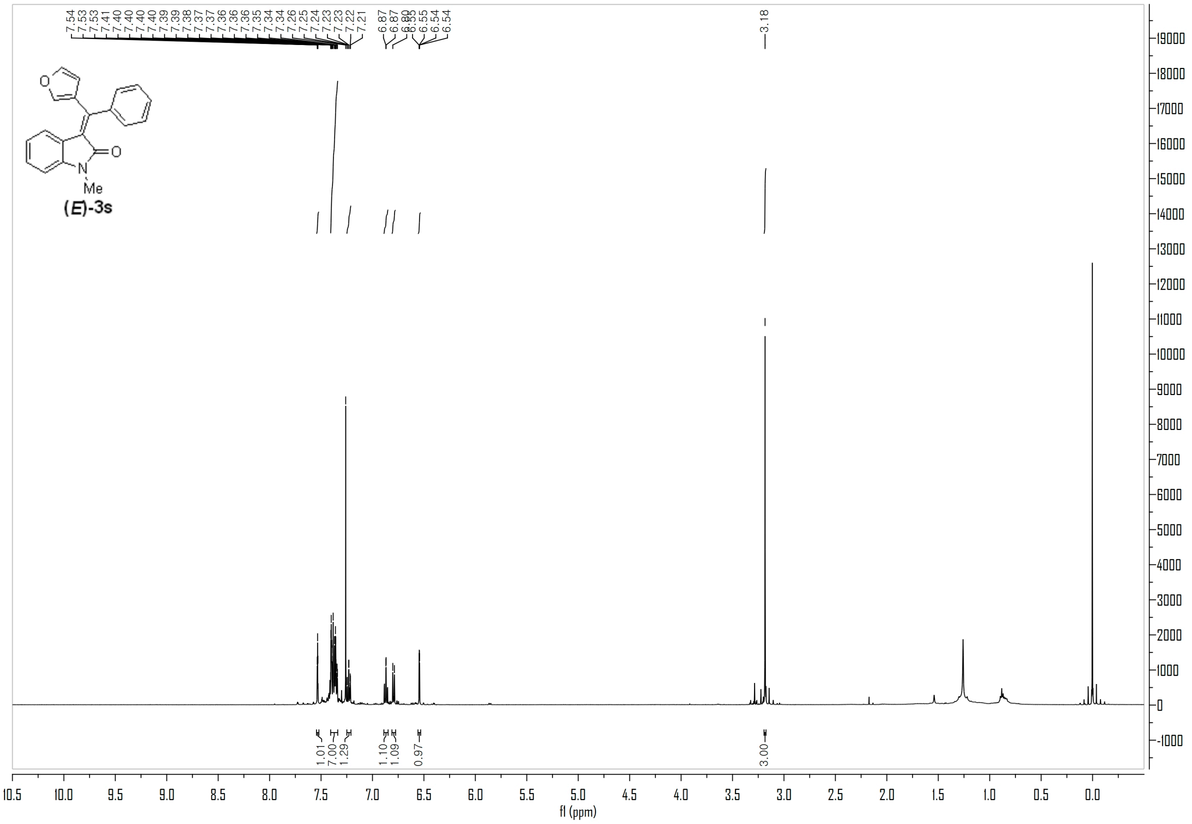

^{13}C NMR spectrum of (*E*)-3s

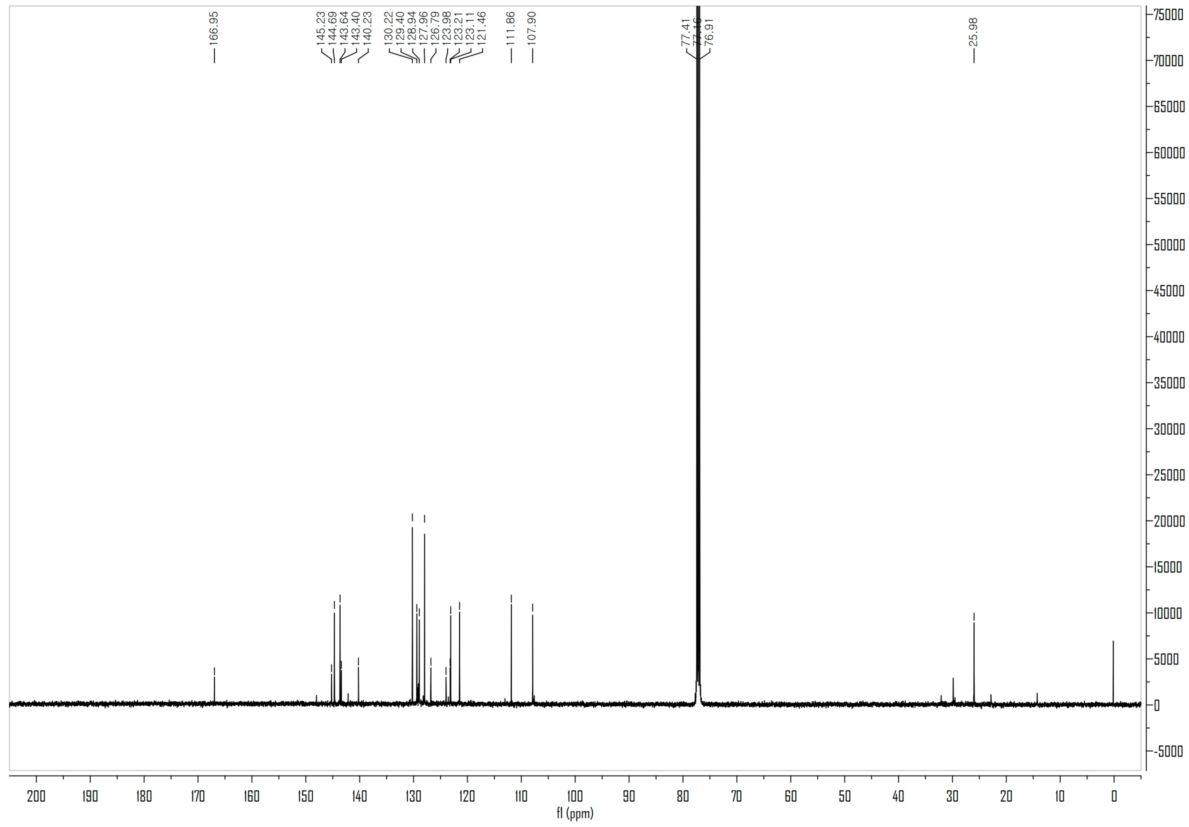

HSQC spectrum of (*E*)-3s

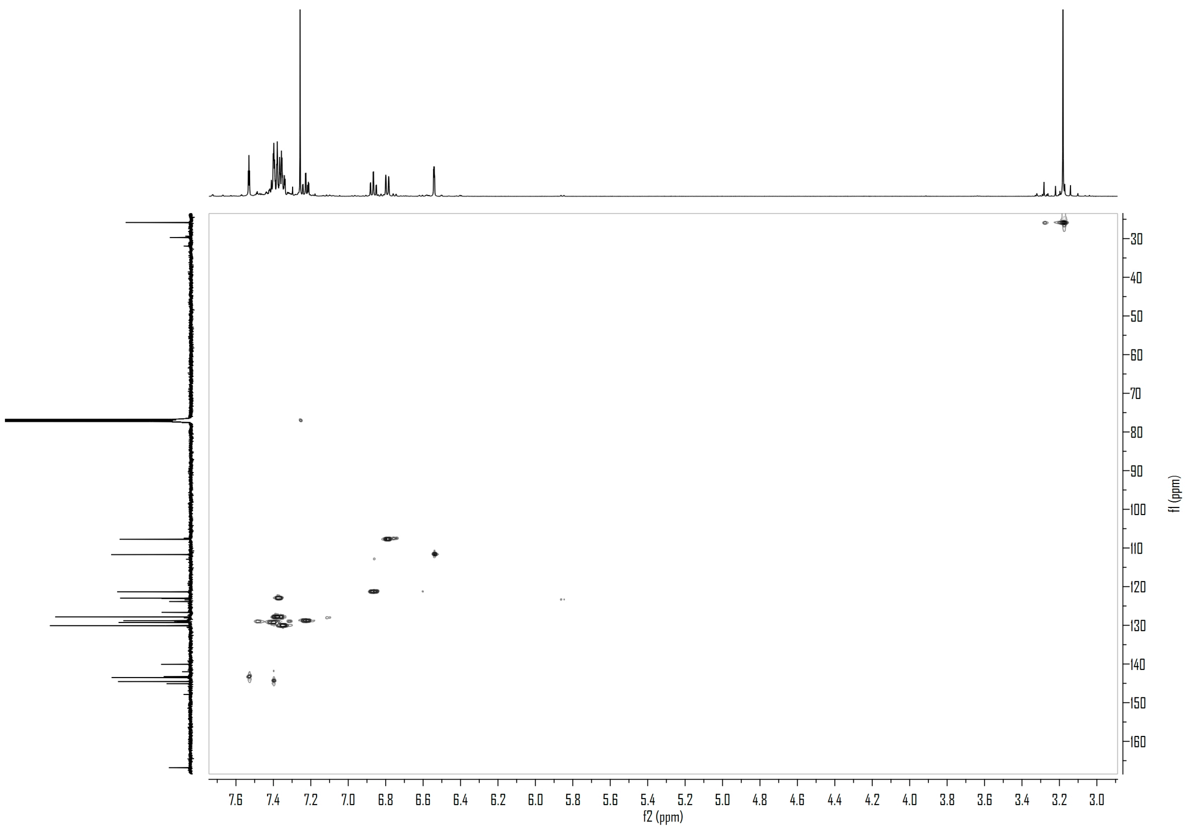

HMBC spectrum of (*E*)-3s

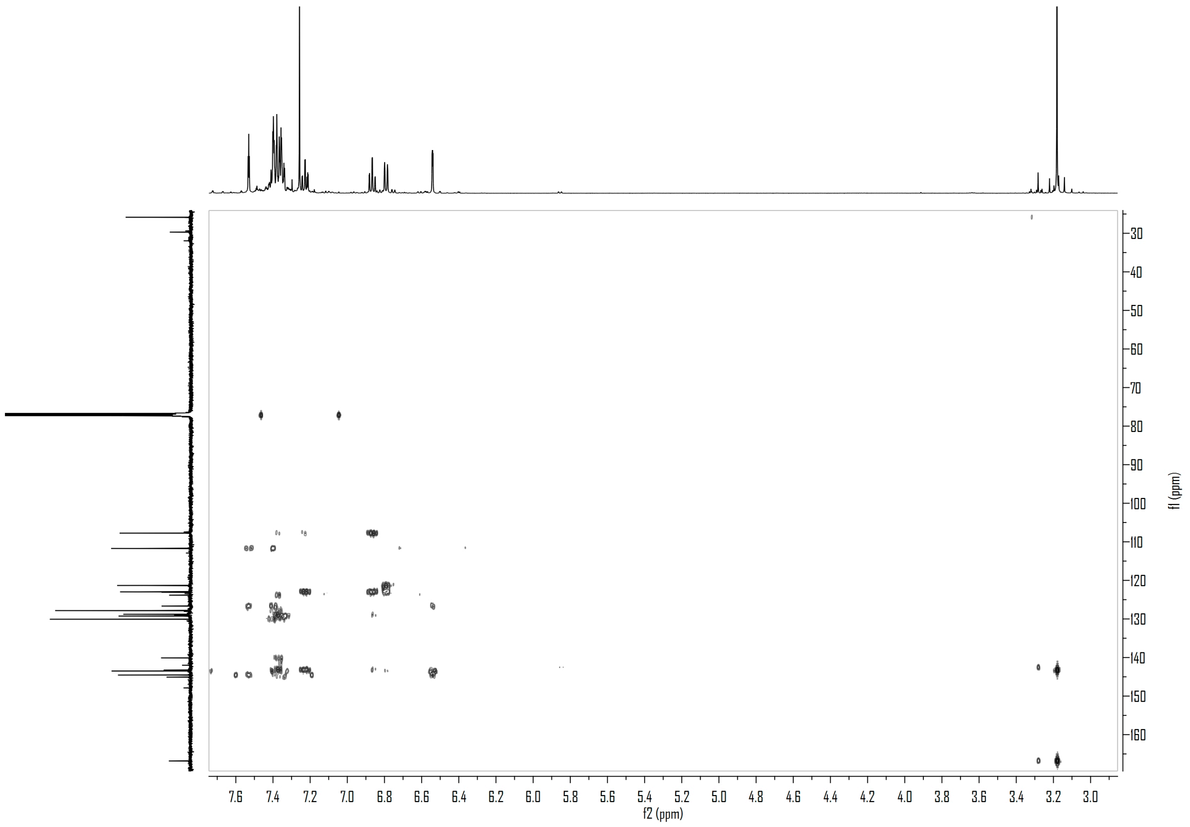

COSY spectrum of (*E*)-3s

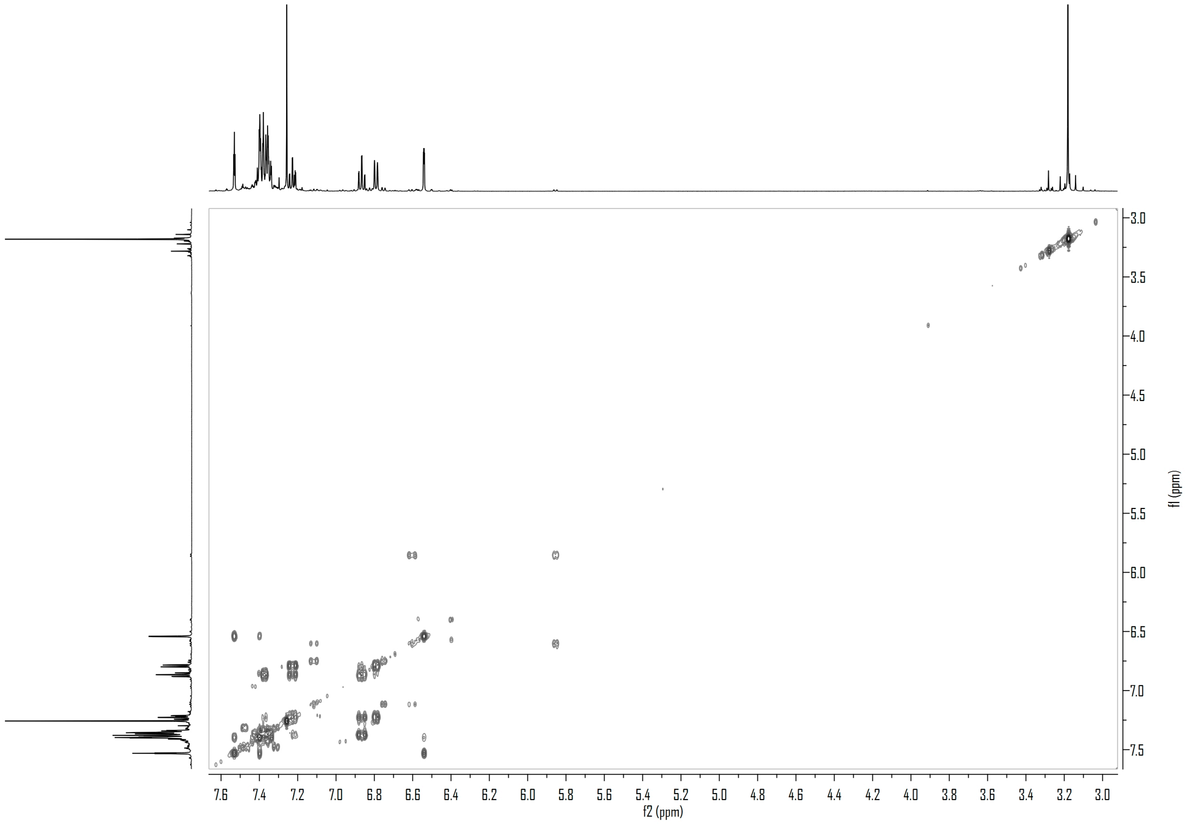

ROESY spectrum of (*E*)-3s

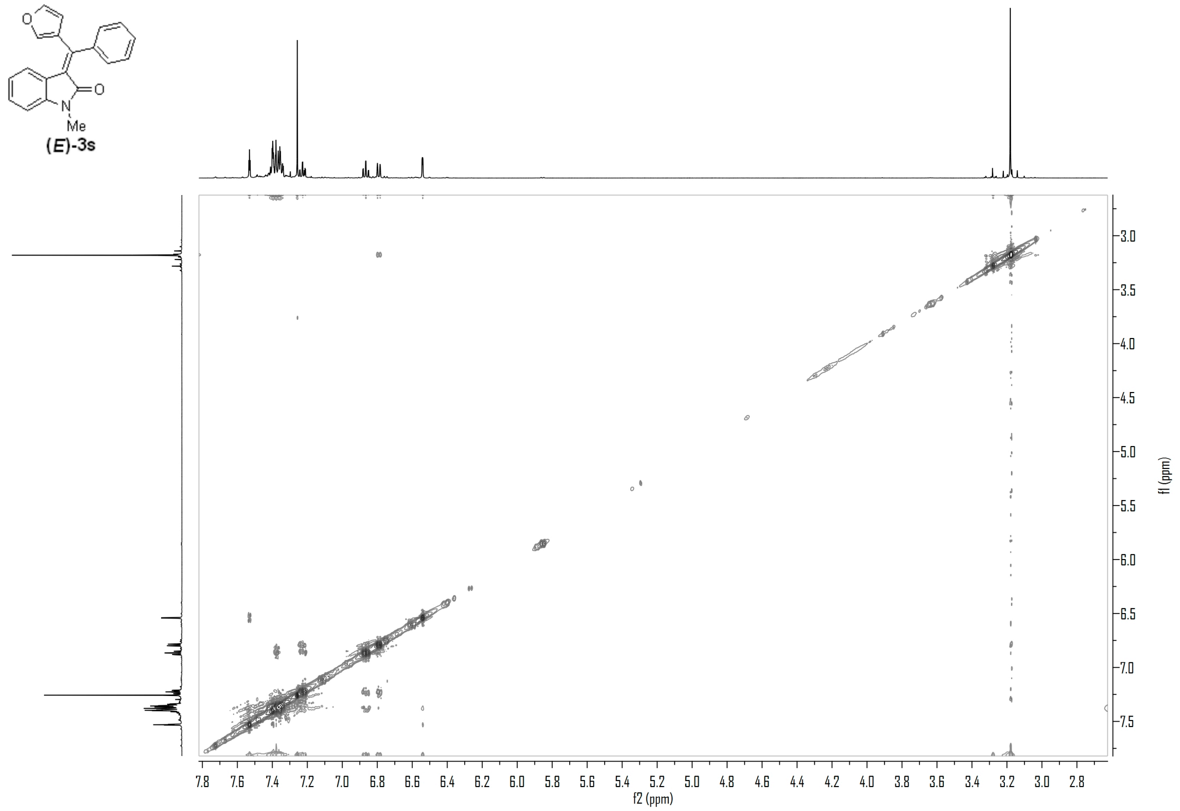

4,4'-dinitro-1,1'-biphenyl (4d)

^{1}H NMR spectrum of 4d

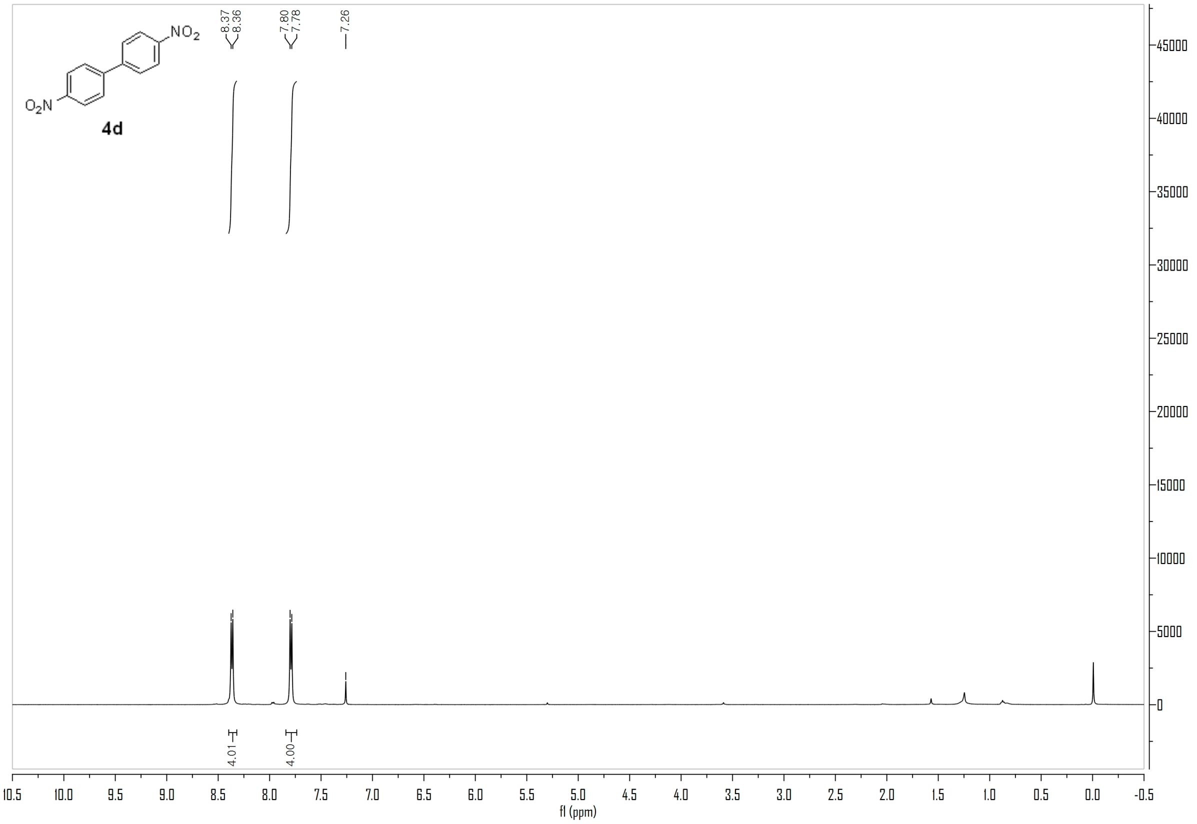

^{13}C NMR spectrum of 4d

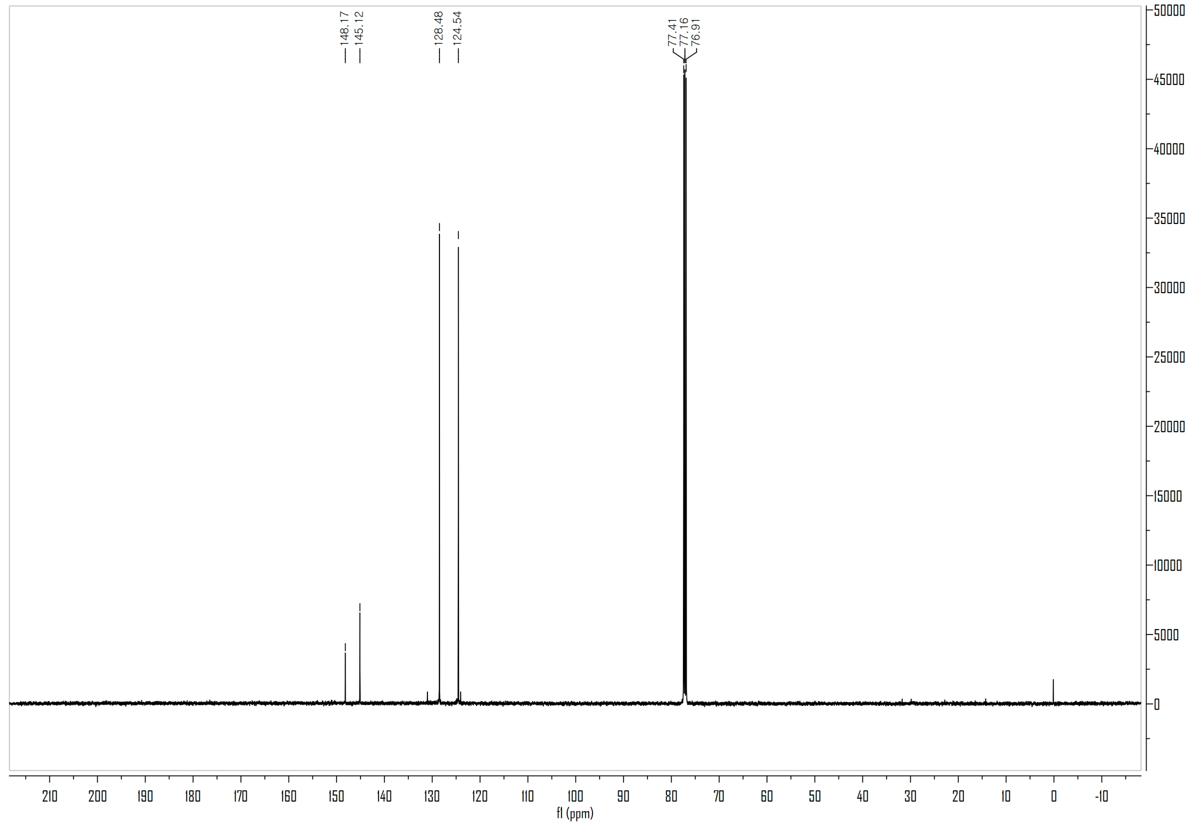

4-methoxy-1,1'-biphenyl (4e)

^{1}H NMR spectrum of 4e

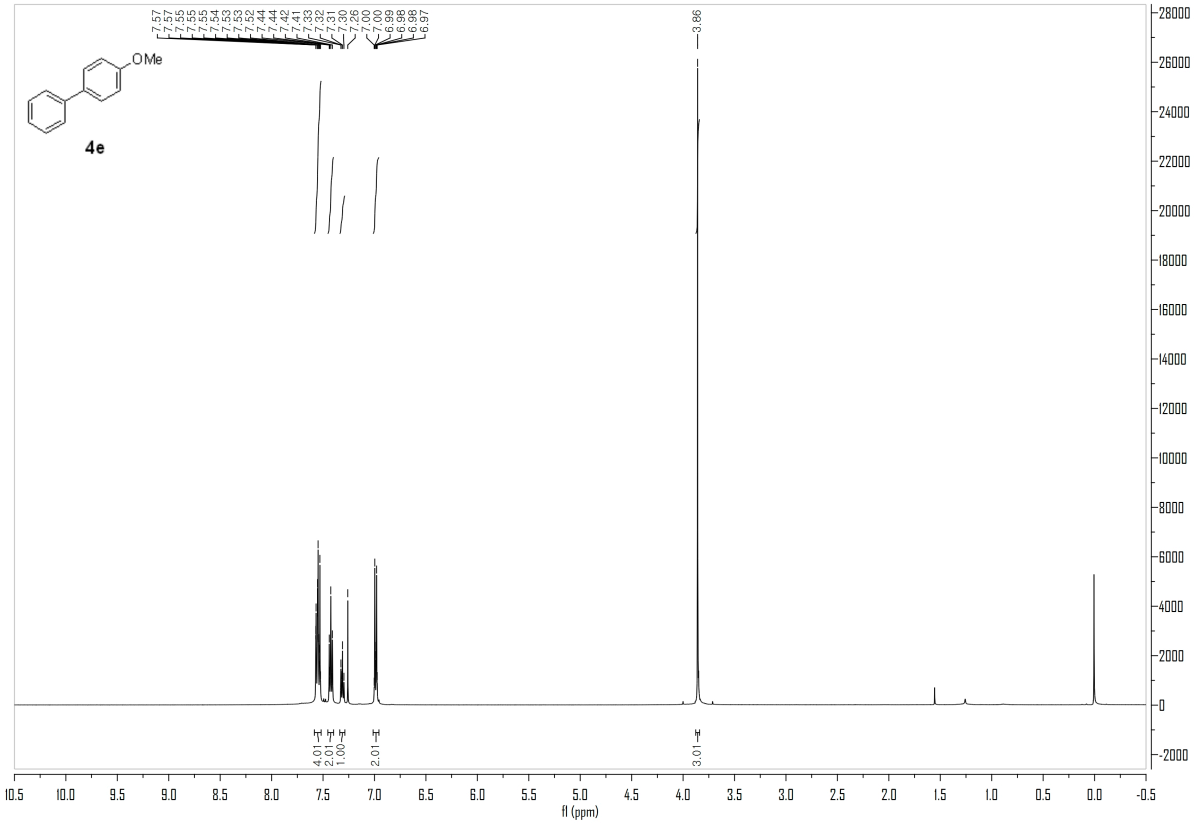

^{13}C NMR spectrum of 4e

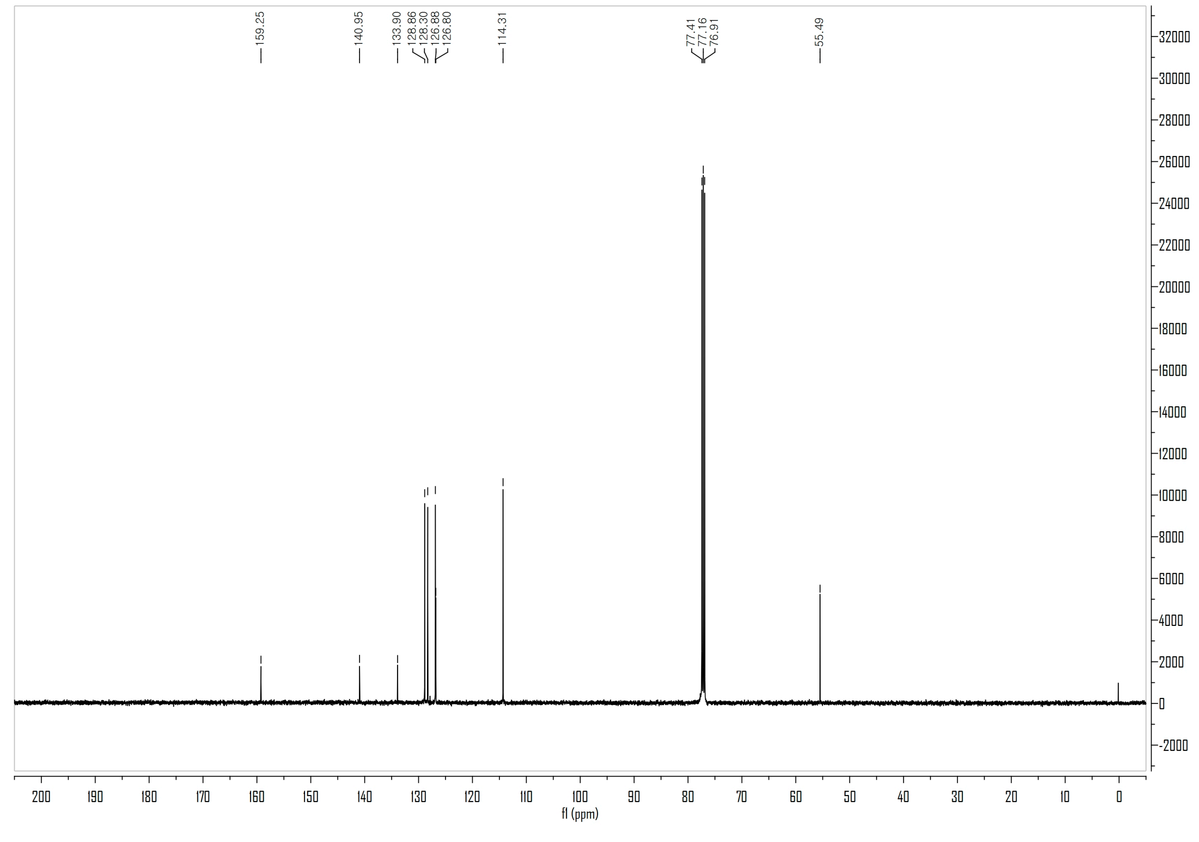

4-chloro-1,1'-biphenyl (4f)

^{1}H NMR spectrum of 4f

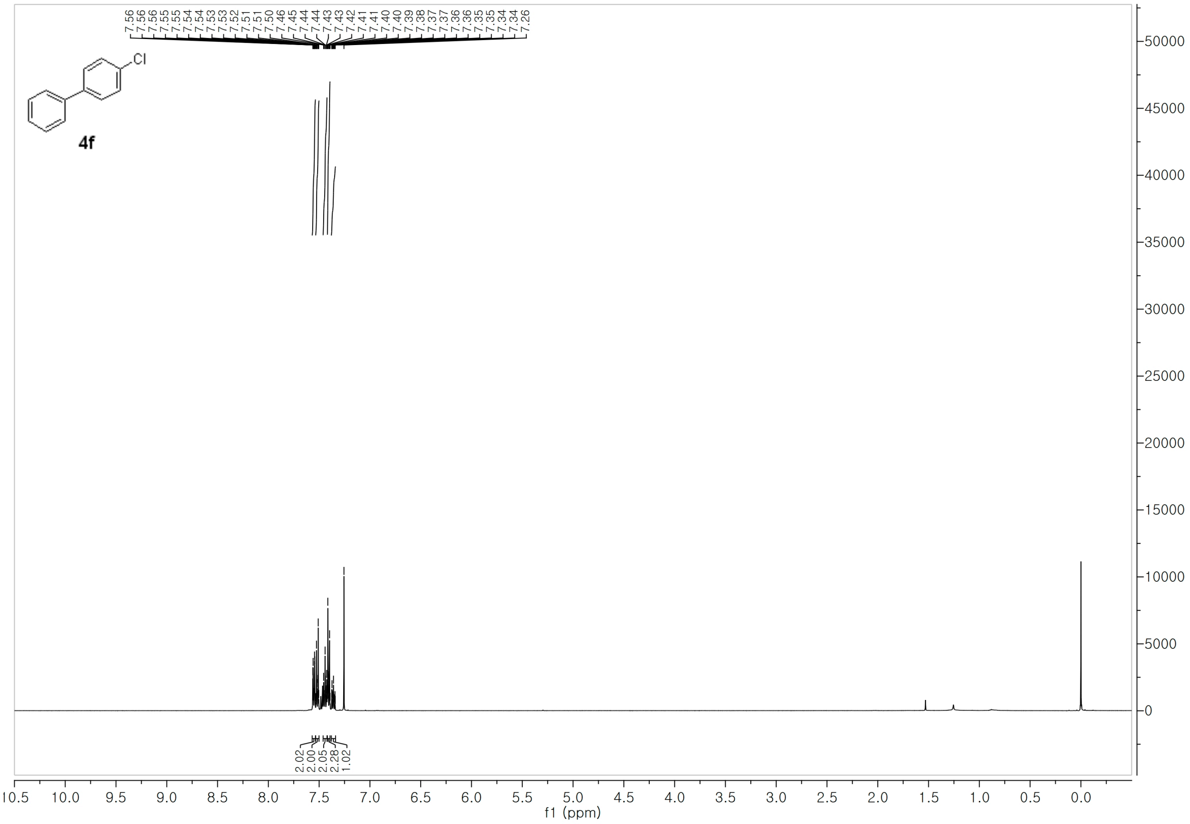

^{13}C NMR spectrum of 4f

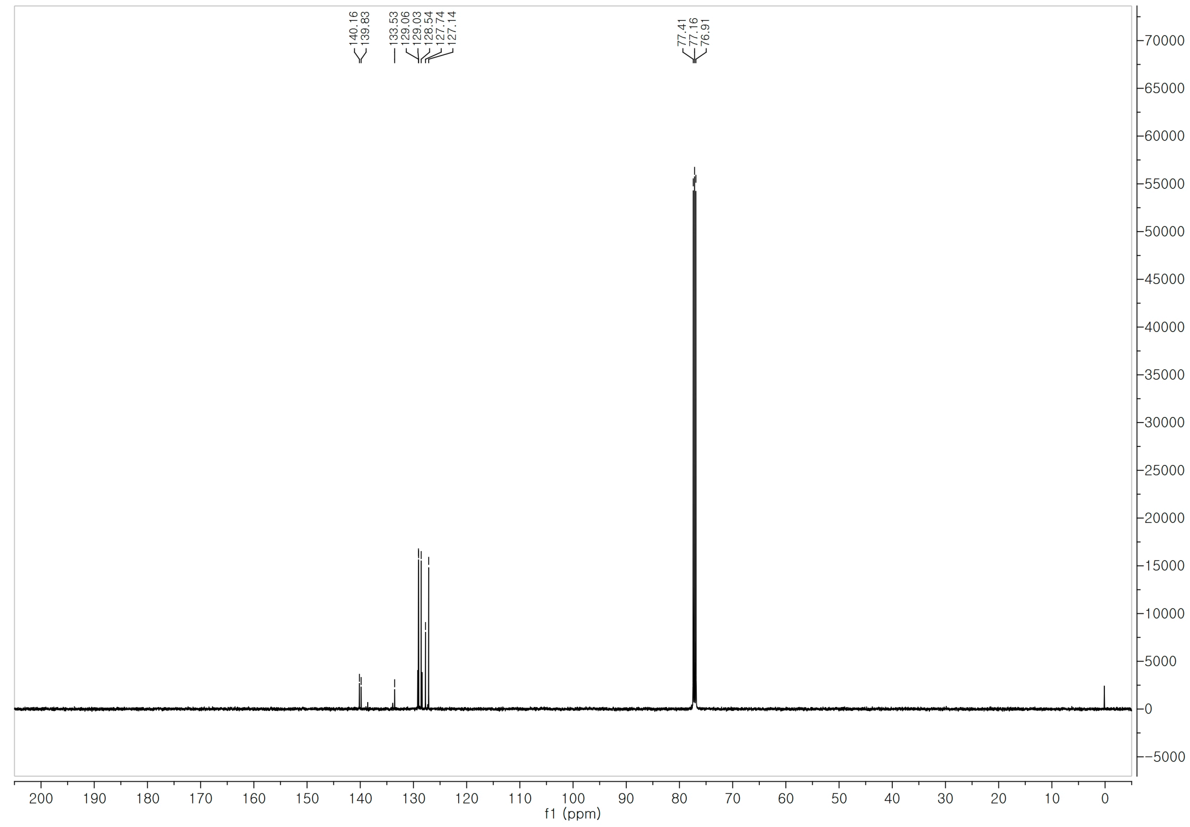

4-nitro-1,1'-biphenyl (4g)

^{1}H NMR spectrum of 4g

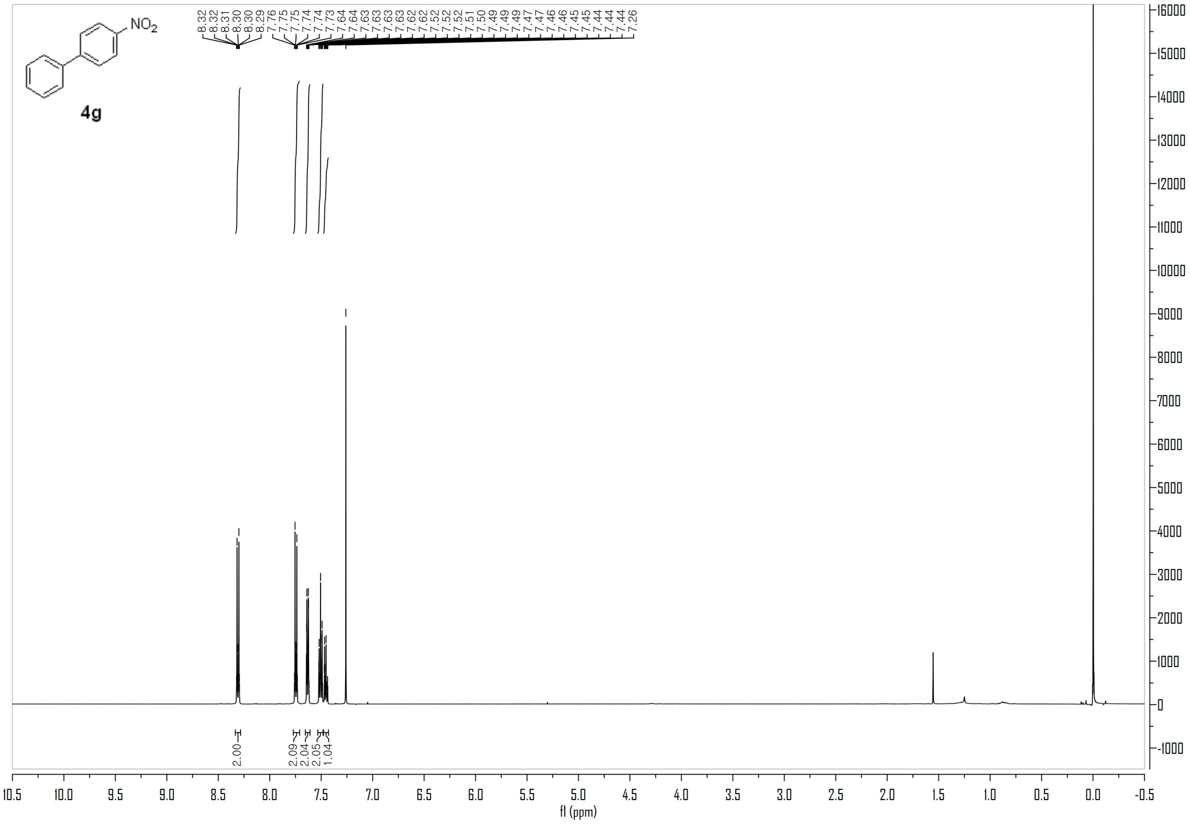

^{13}C NMR spectrum of 4g

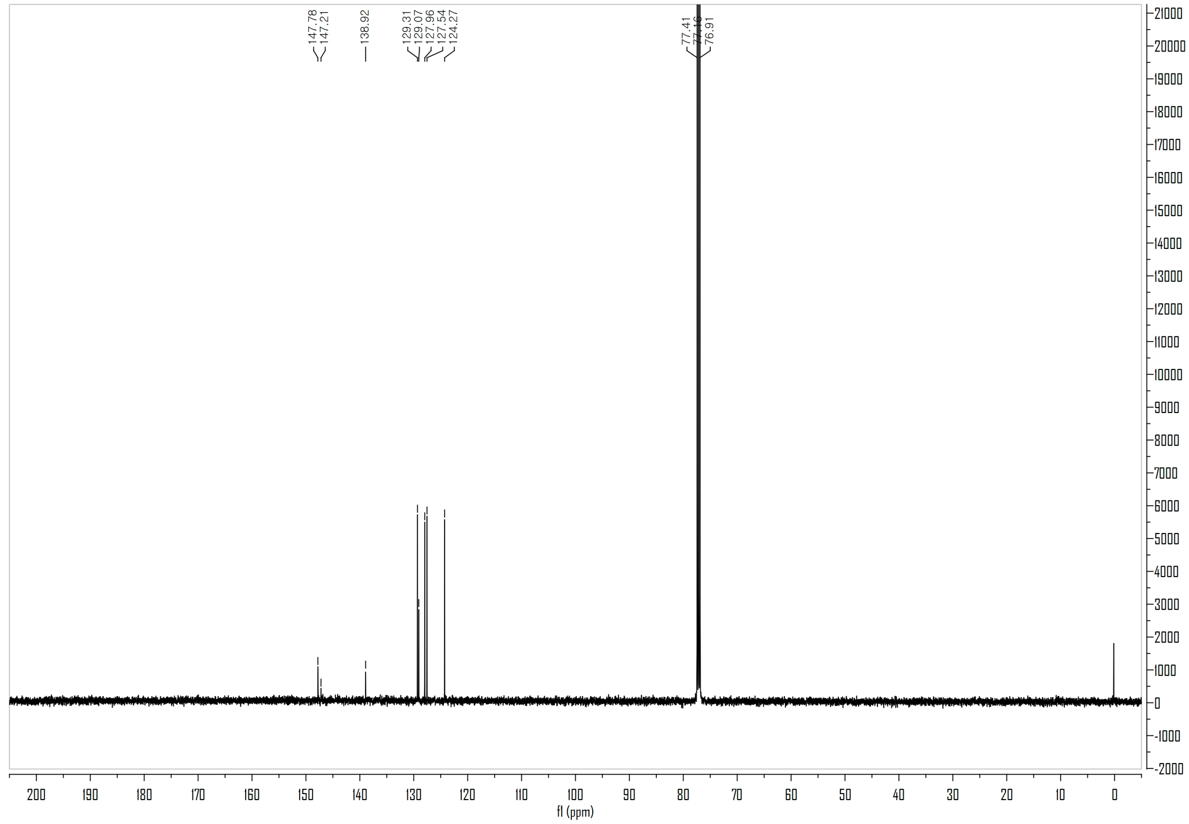

9-methyl-4-phenylpyrano[2,3-b]indol-2(9H)-one (5)

^{1}H NMR spectrum of 5

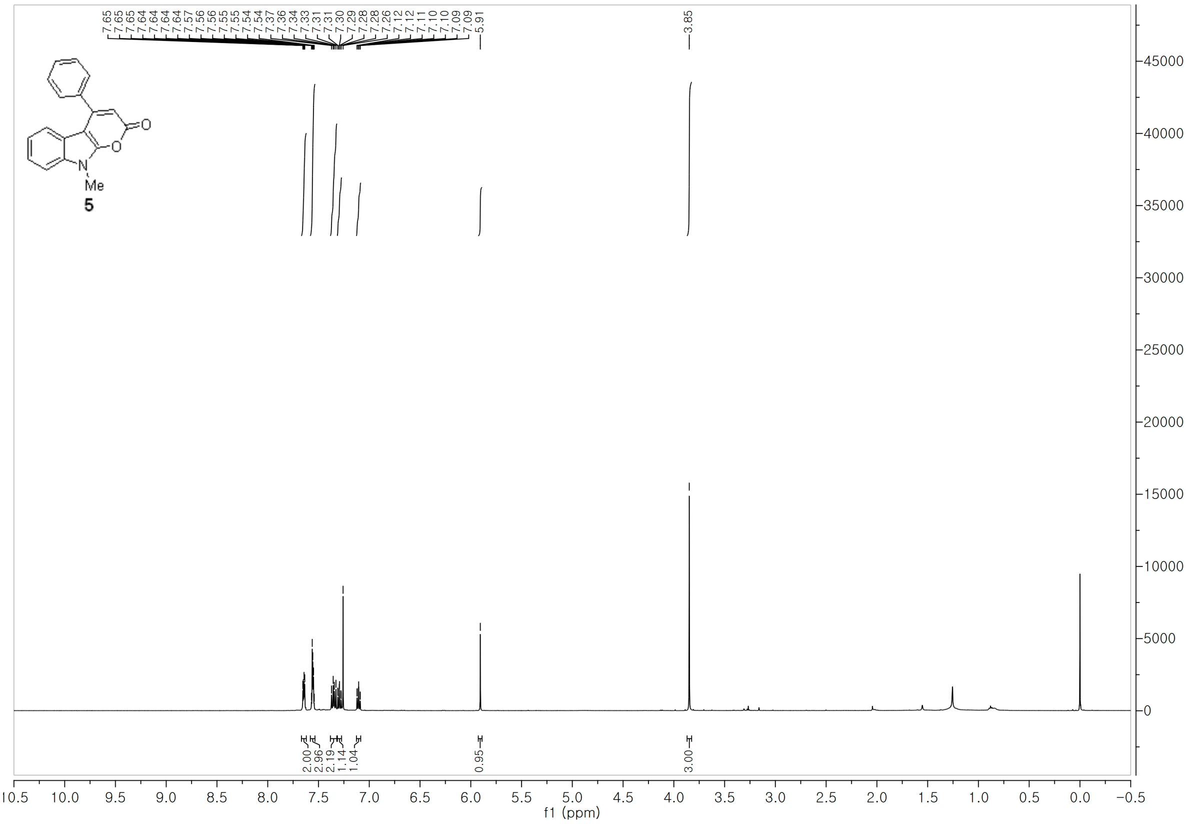

^{13}C NMR spectrum of 5

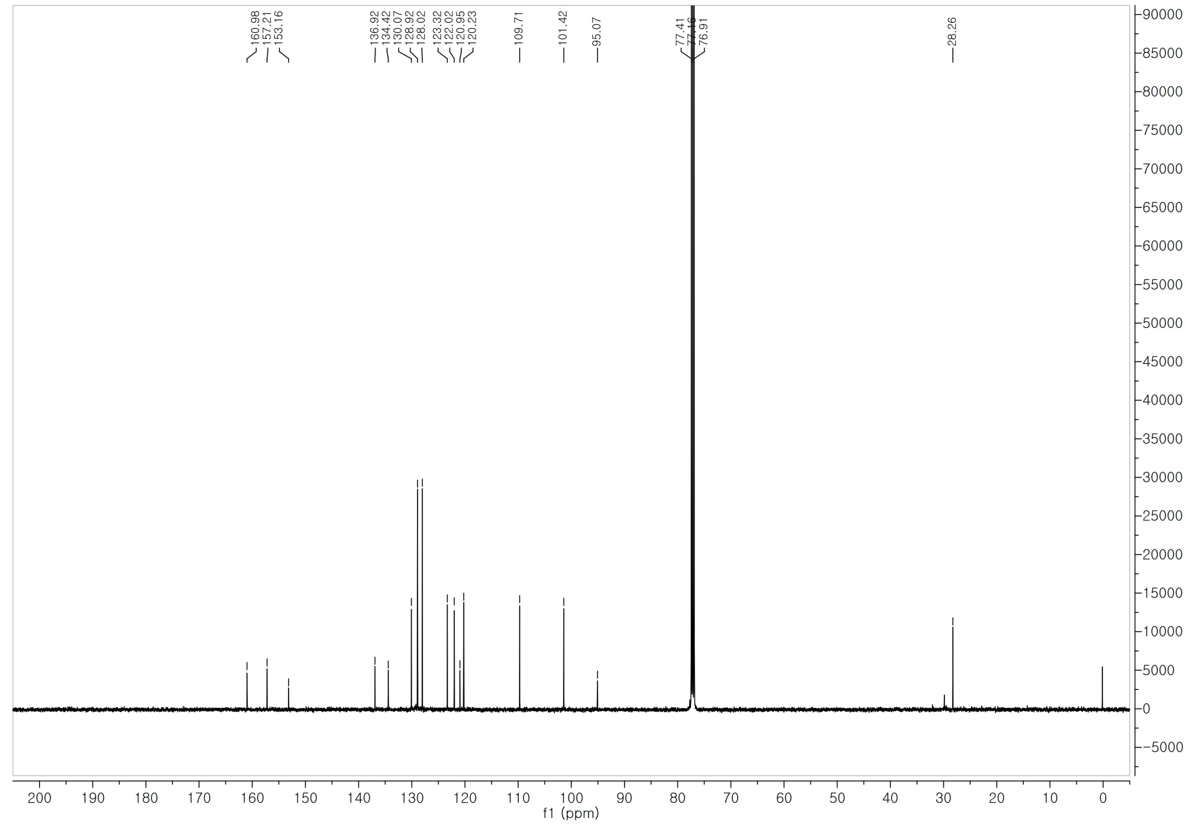

HSQC spectrum of 5

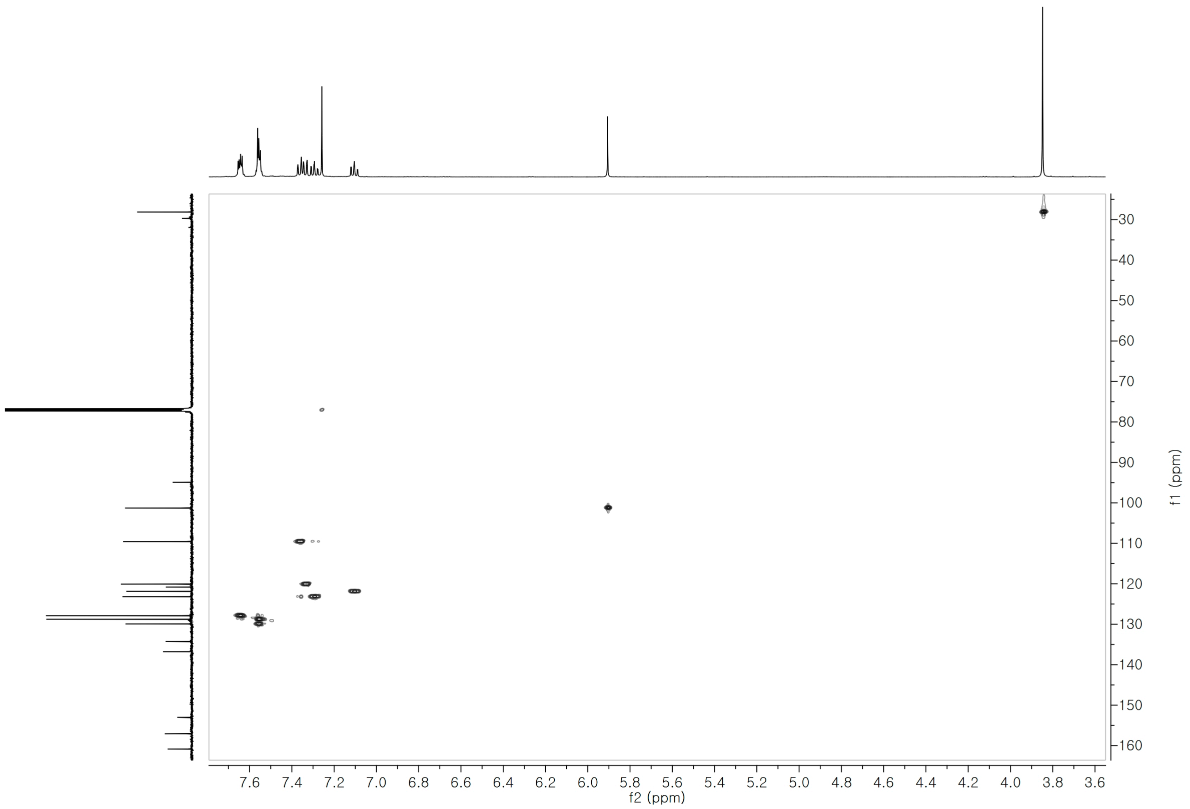

HMBC spectrum of 5

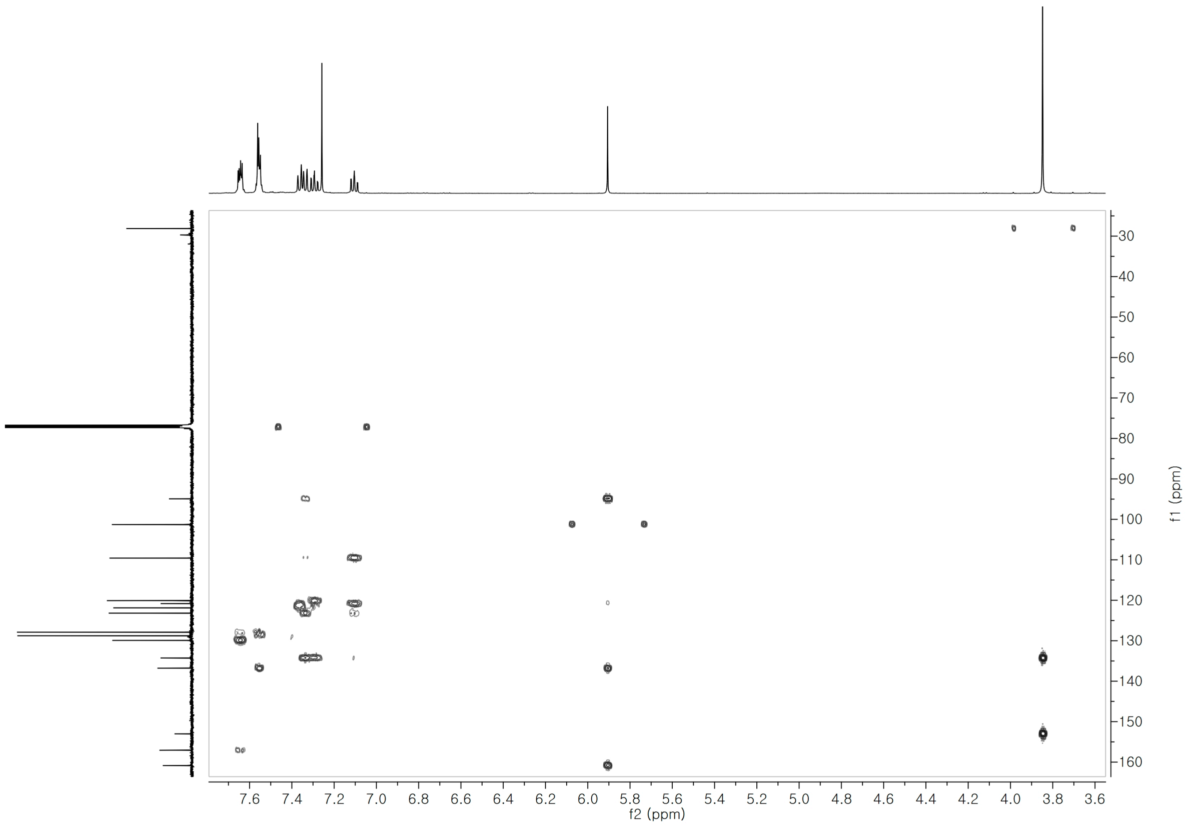

COSY spectrum of 5

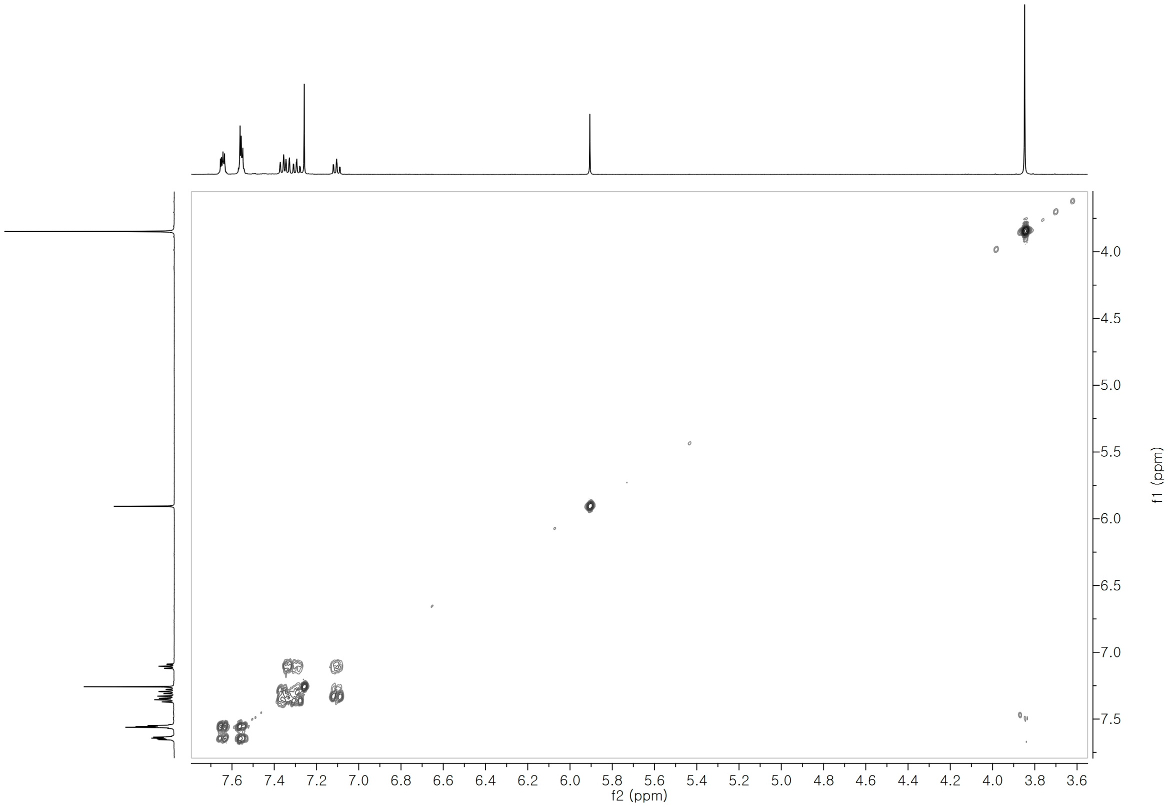

ROESY spectrum of 5

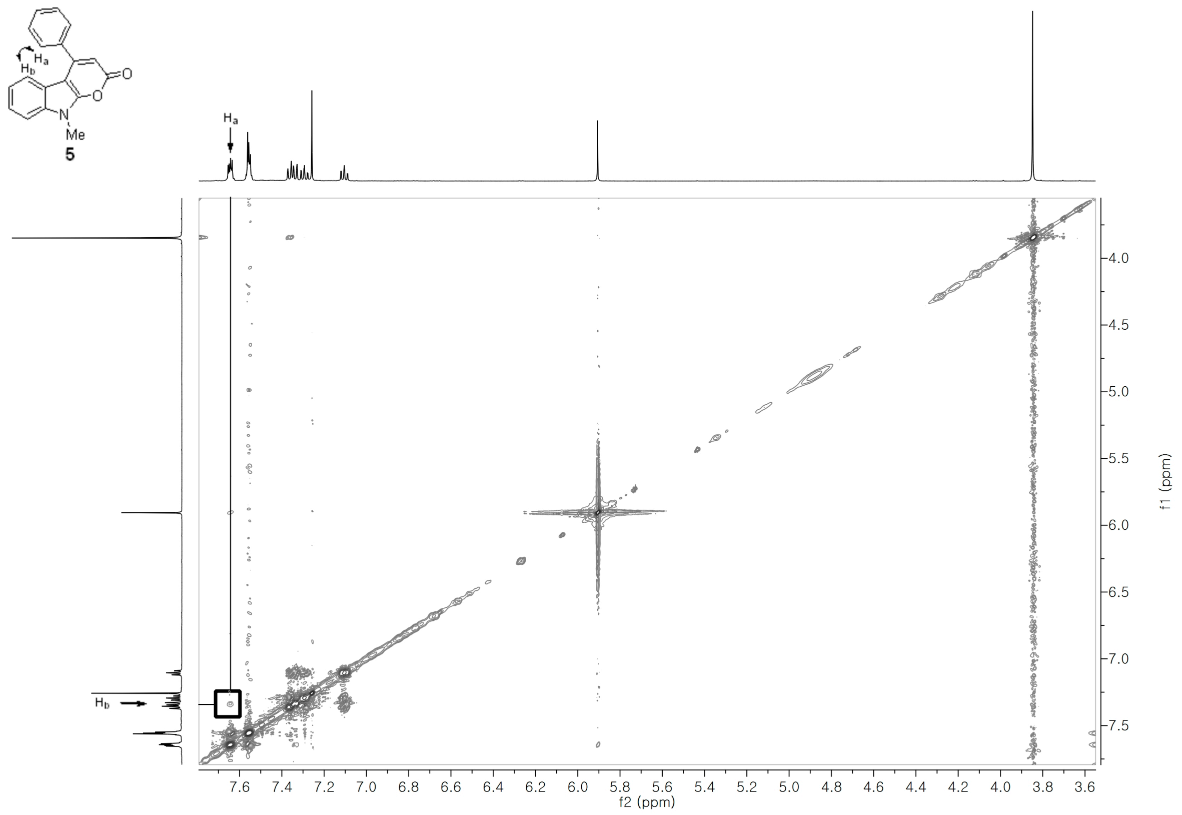

N-(2-bromophenyl)propiolamide (6a)

^{1}H NMR spectrum of 6a

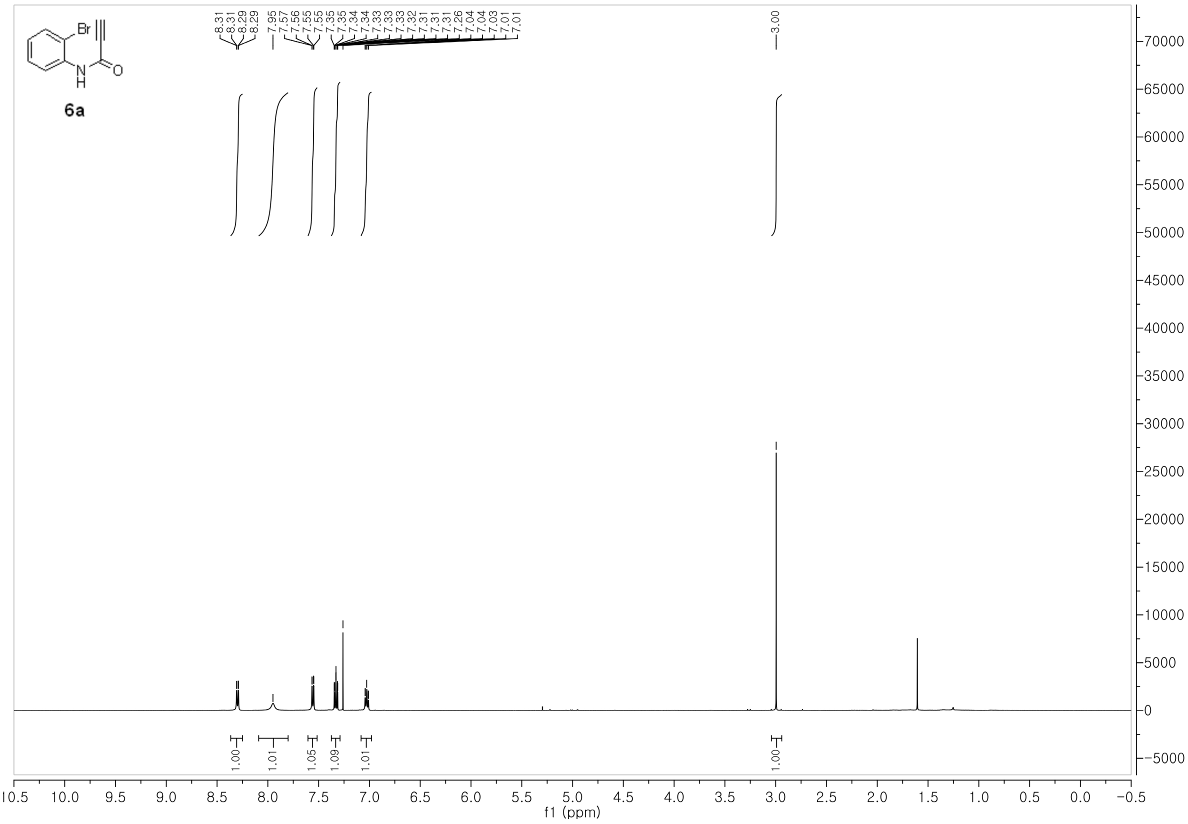

^{13}C NMR spectrum of 6a

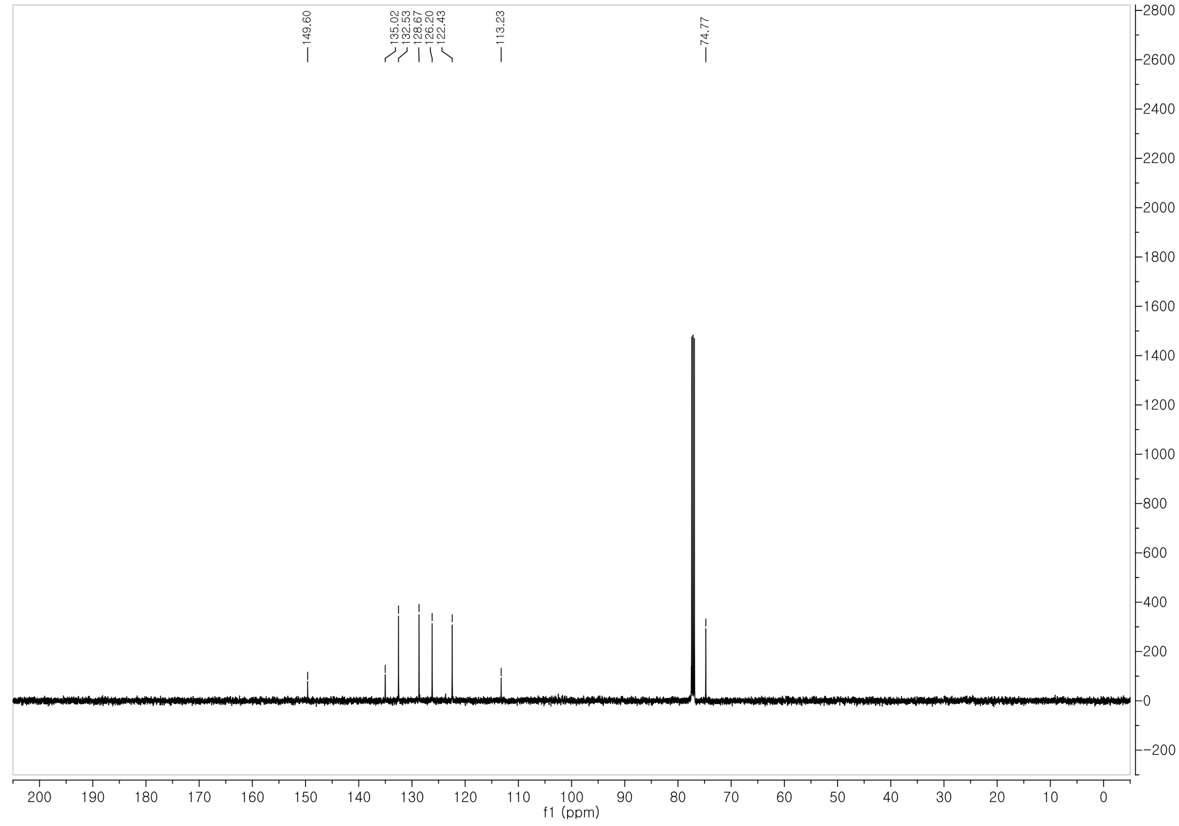

N-benzyl-*N*-(2-bromophenyl)propiolamide (6b)

^{1}H NMR spectrum of 6b

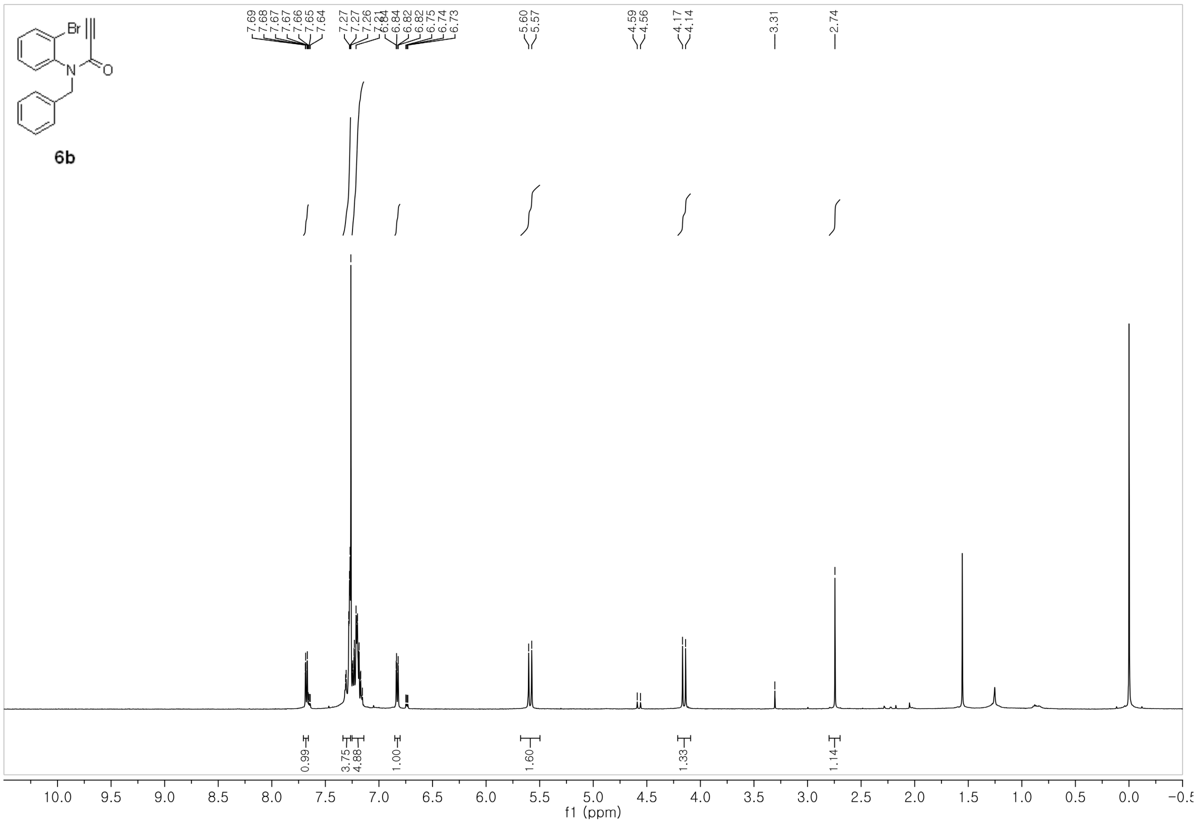

^{13}C NMR spectrum of 6b

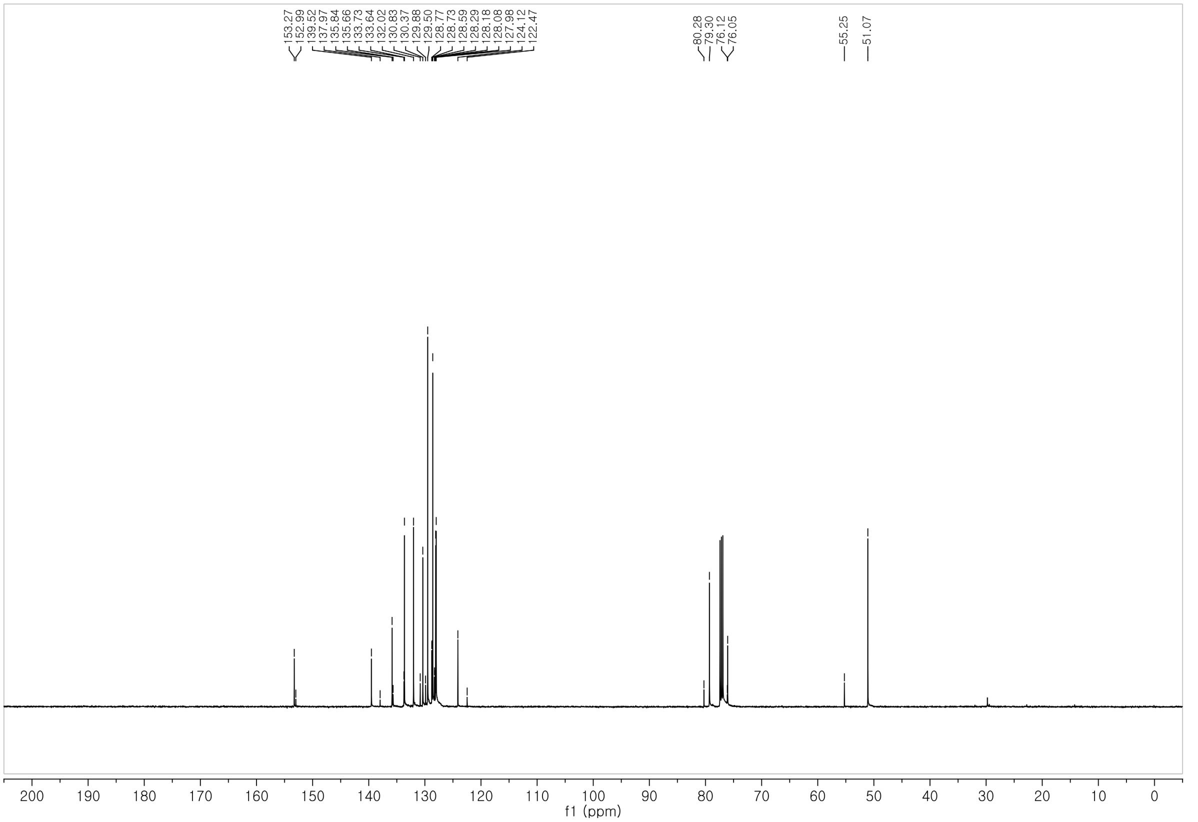

N-(2-bromobenzyl)-*N*-methylpropiolamide (6c)

^{1}H NMR spectrum of 6c

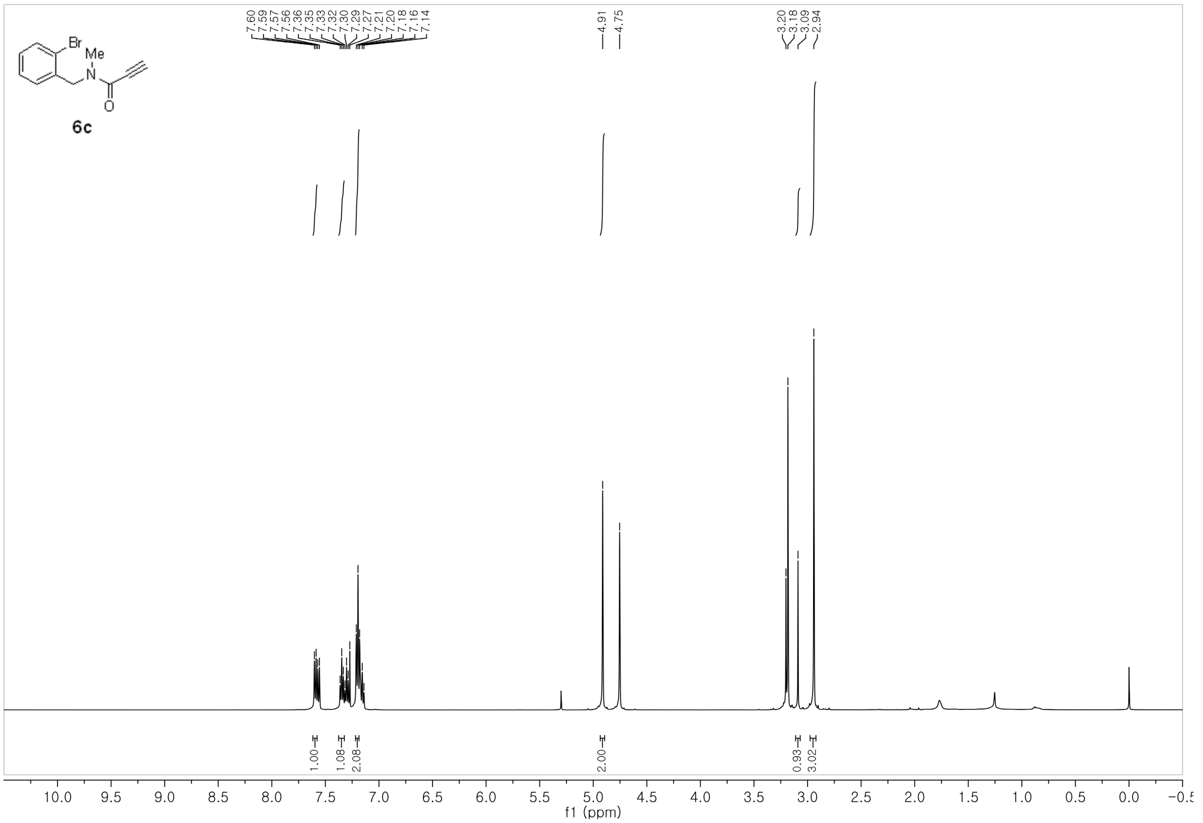

^{13}C NMR spectrum of 6c

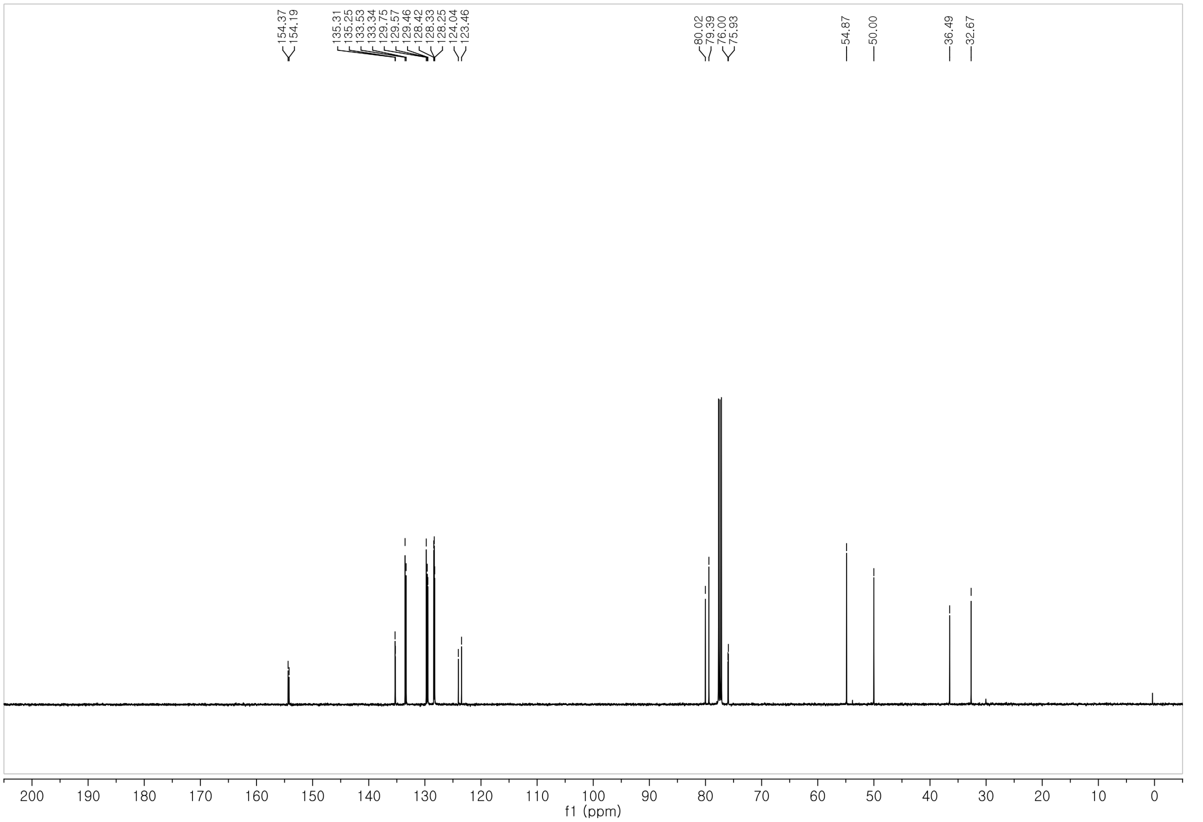

2-bromophenyl propiolate (6d)

^{1}H NMR spectrum of 6d

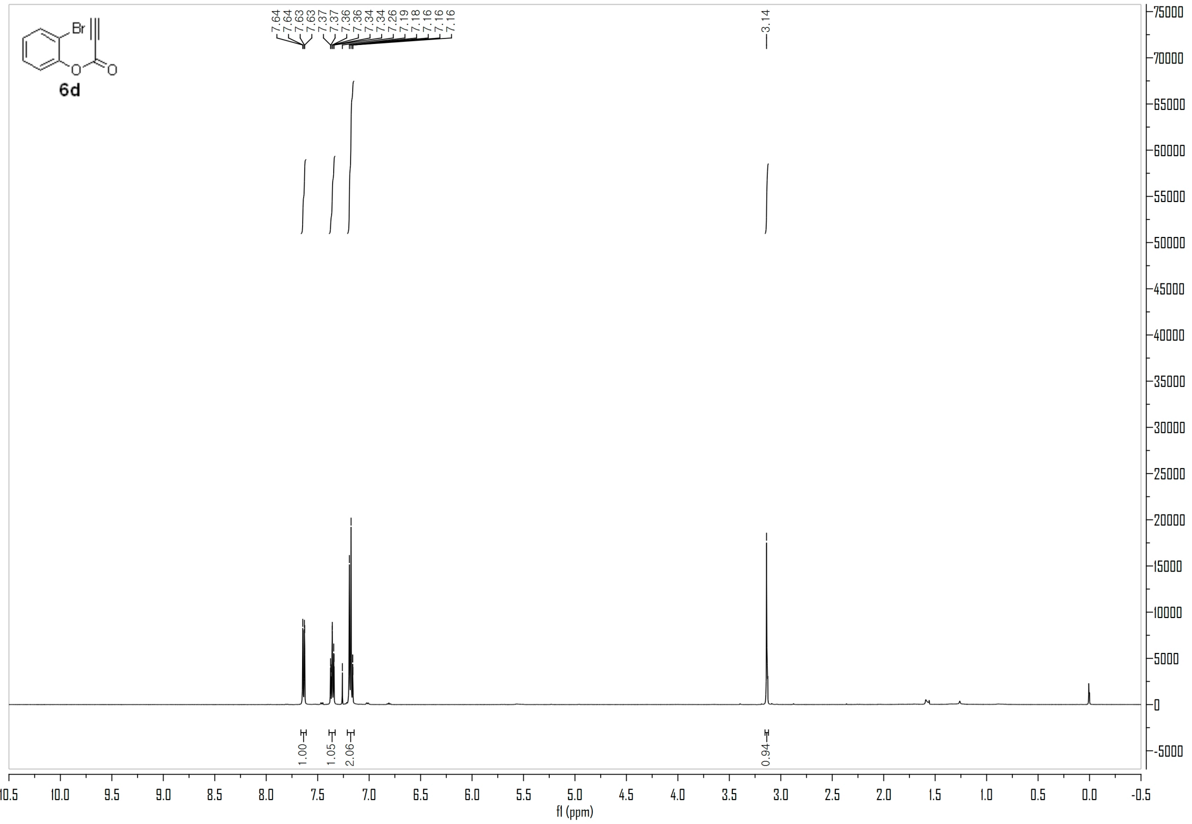

^{13}C NMR spectrum of 6d

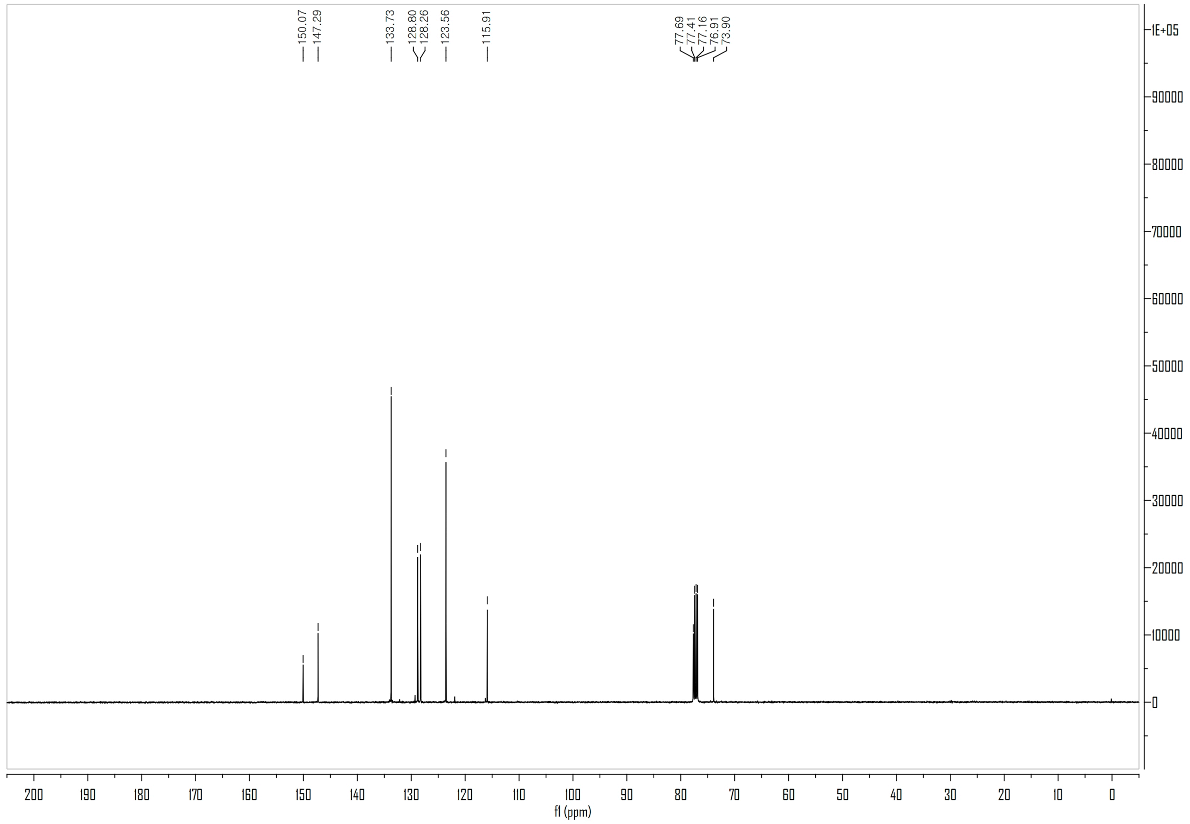

(*Z*)-3-((4-chlorophenyl)(phenyl)methylene)indolin-2-one one ((*Z*)-7a)

^{1}H NMR spectrum of (*Z*)-7a

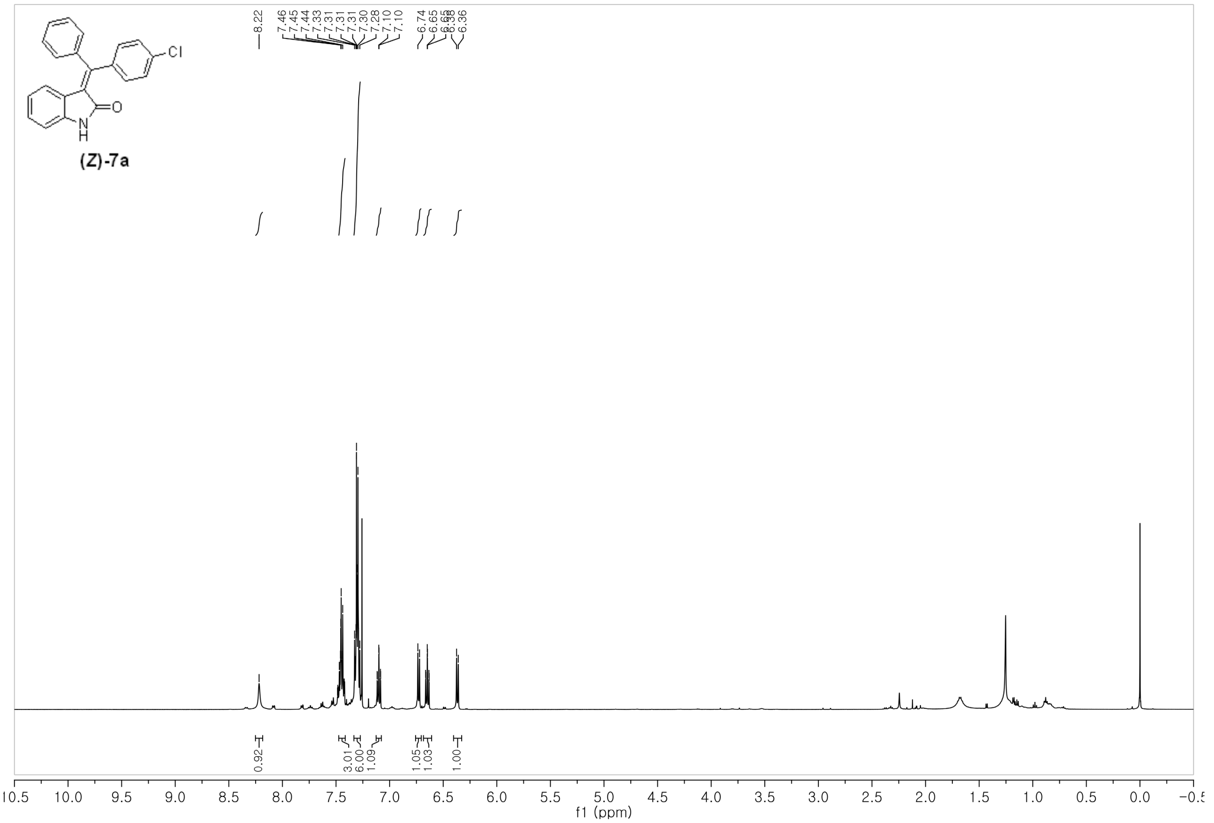

^{13}C NMR spectrum of (*Z*)-7a

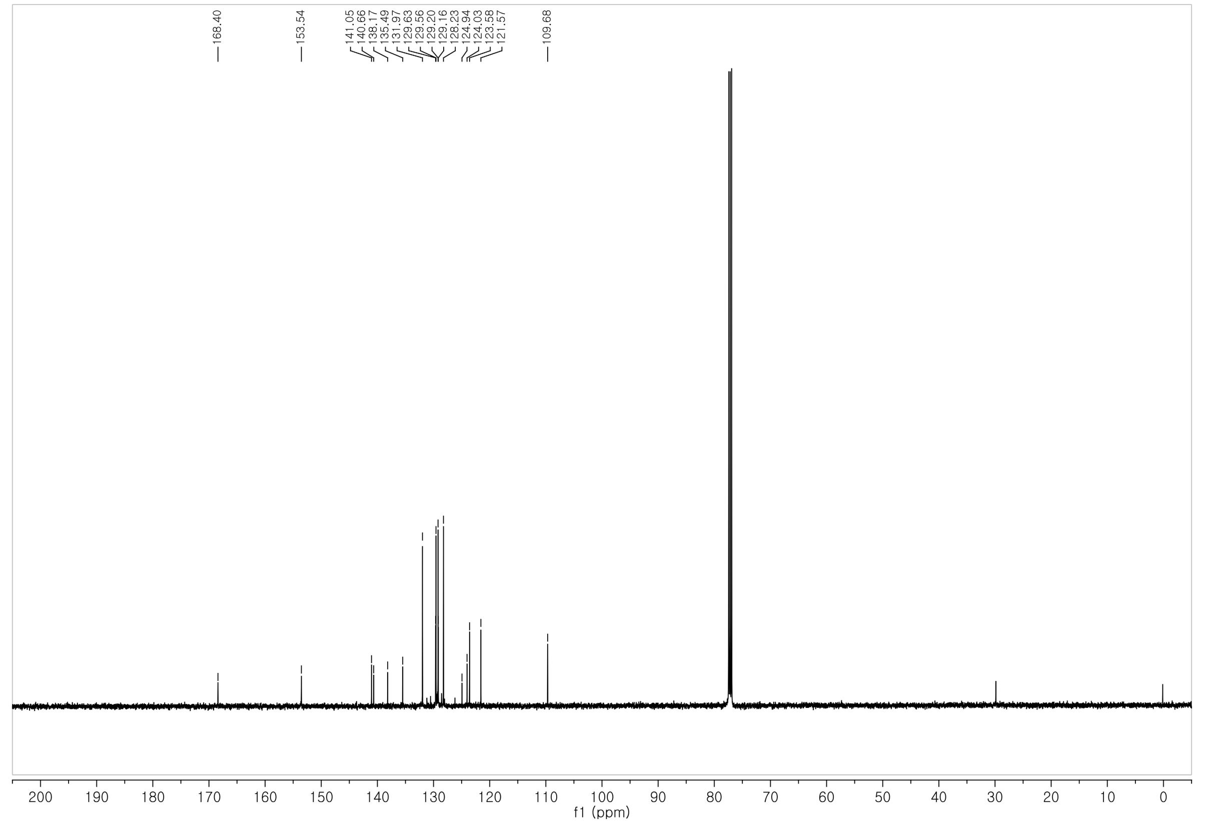

HSQC spectrum of (*Z*)-7a

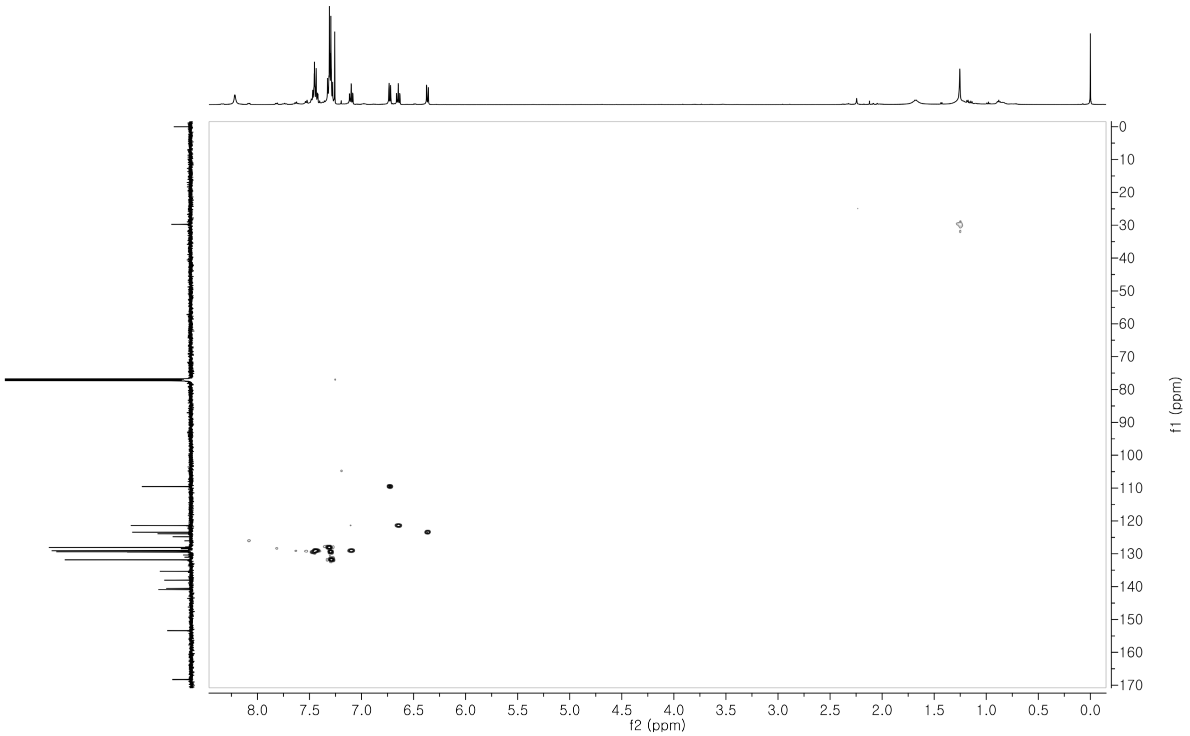

HMBC spectrum of (*Z*)-7a

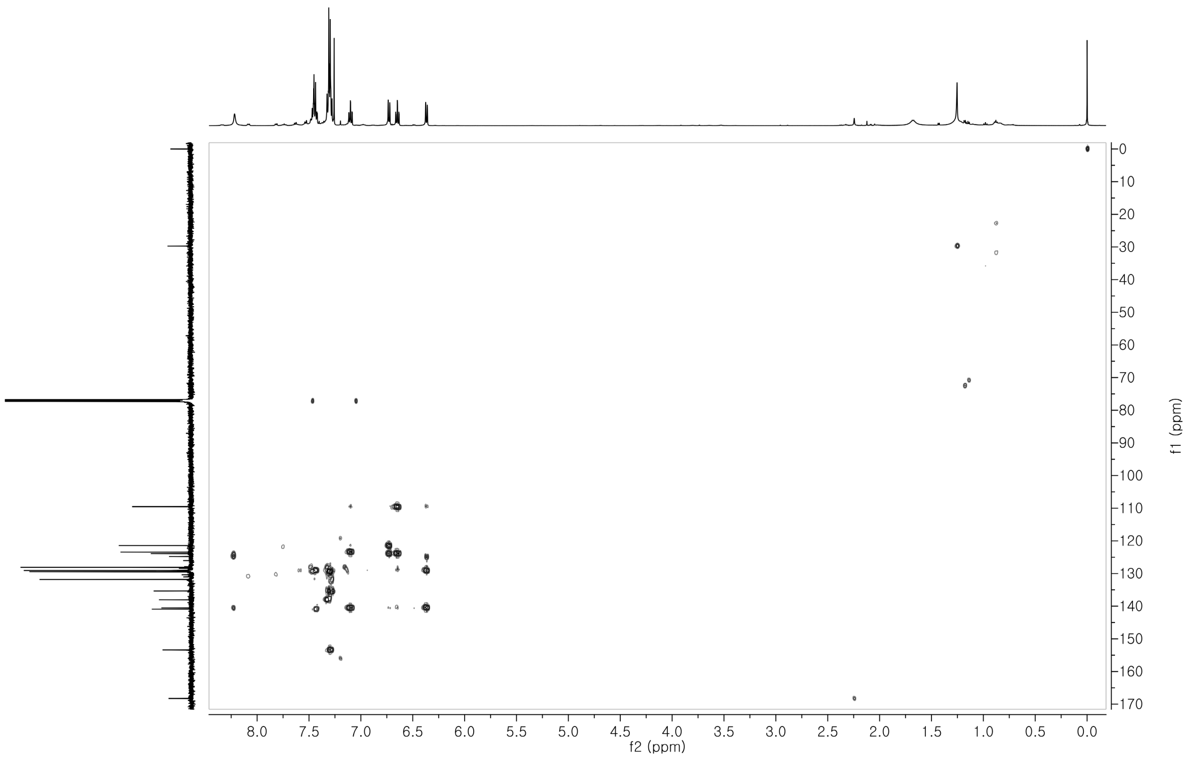

COSY spectrum of (*Z*)-7a

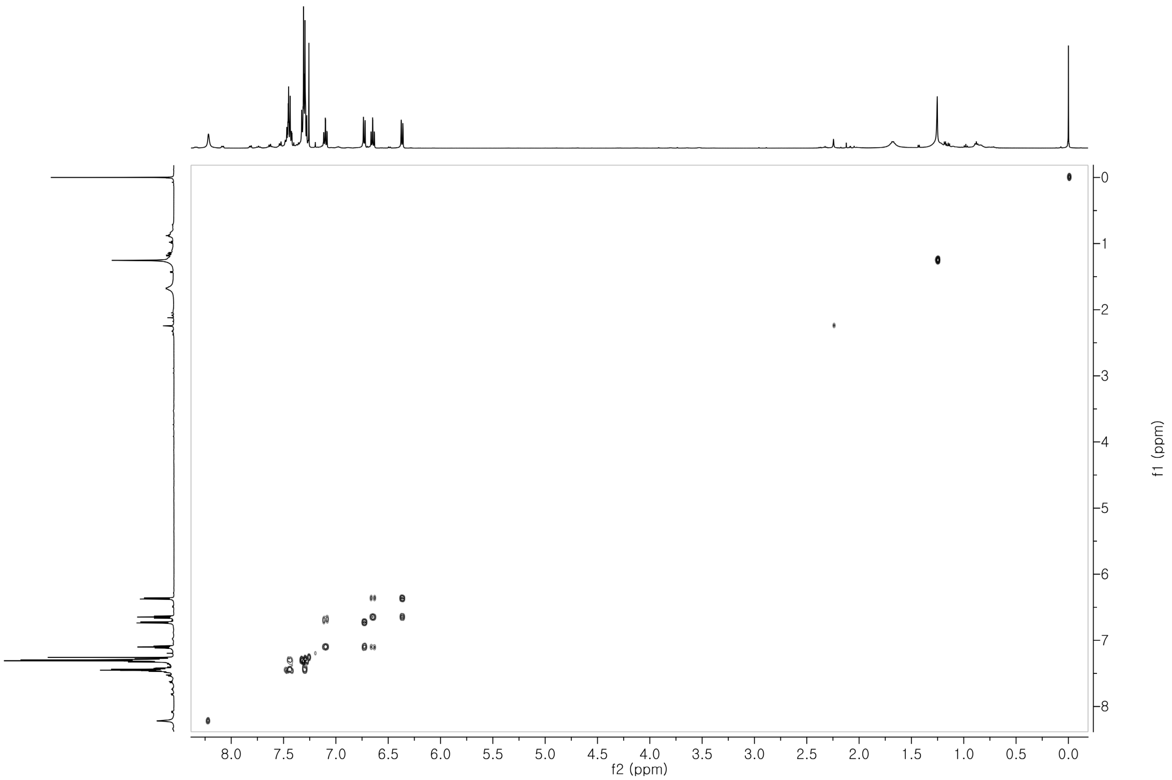

ROESY spectrum of (*Z*)-7a

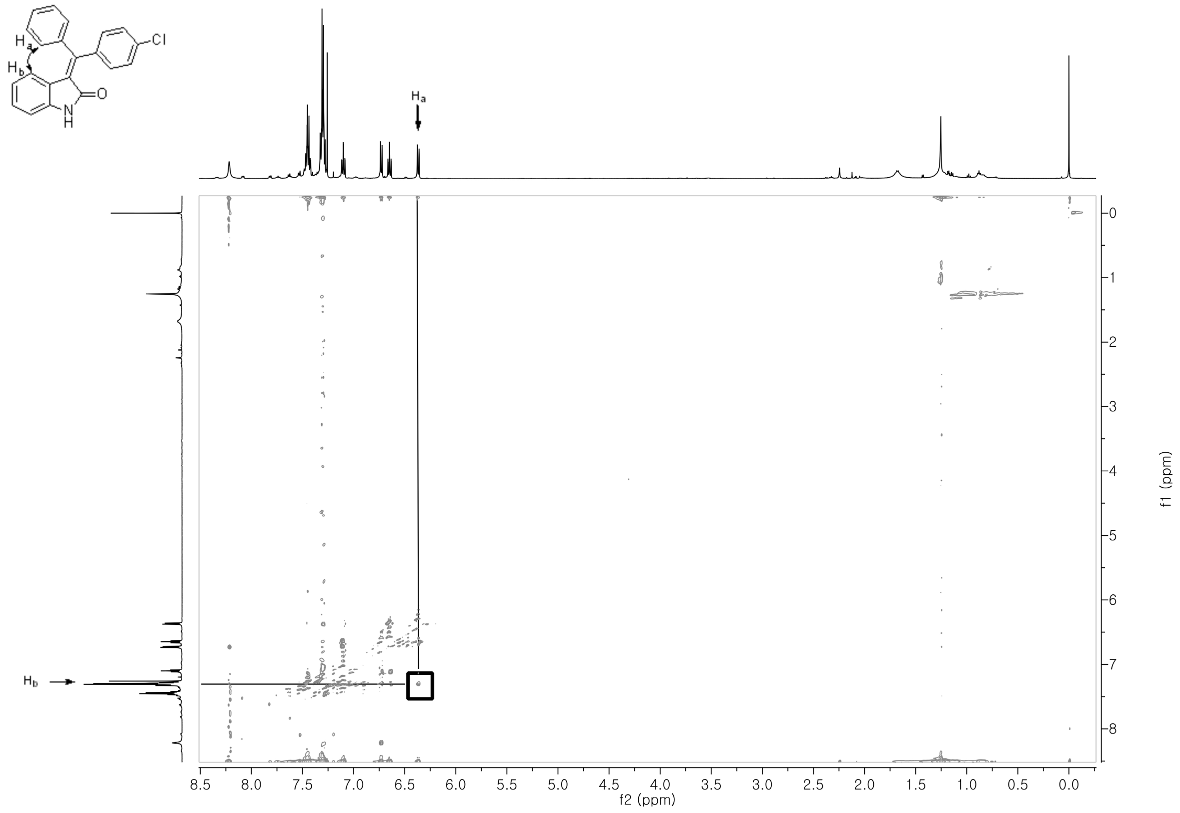

(*E*)-3-((4-chlorophenyl)(phenyl)methylene)indolin-2-one ((*E*)-7a)

^{1}H NMR spectrum of (*E*)-7a

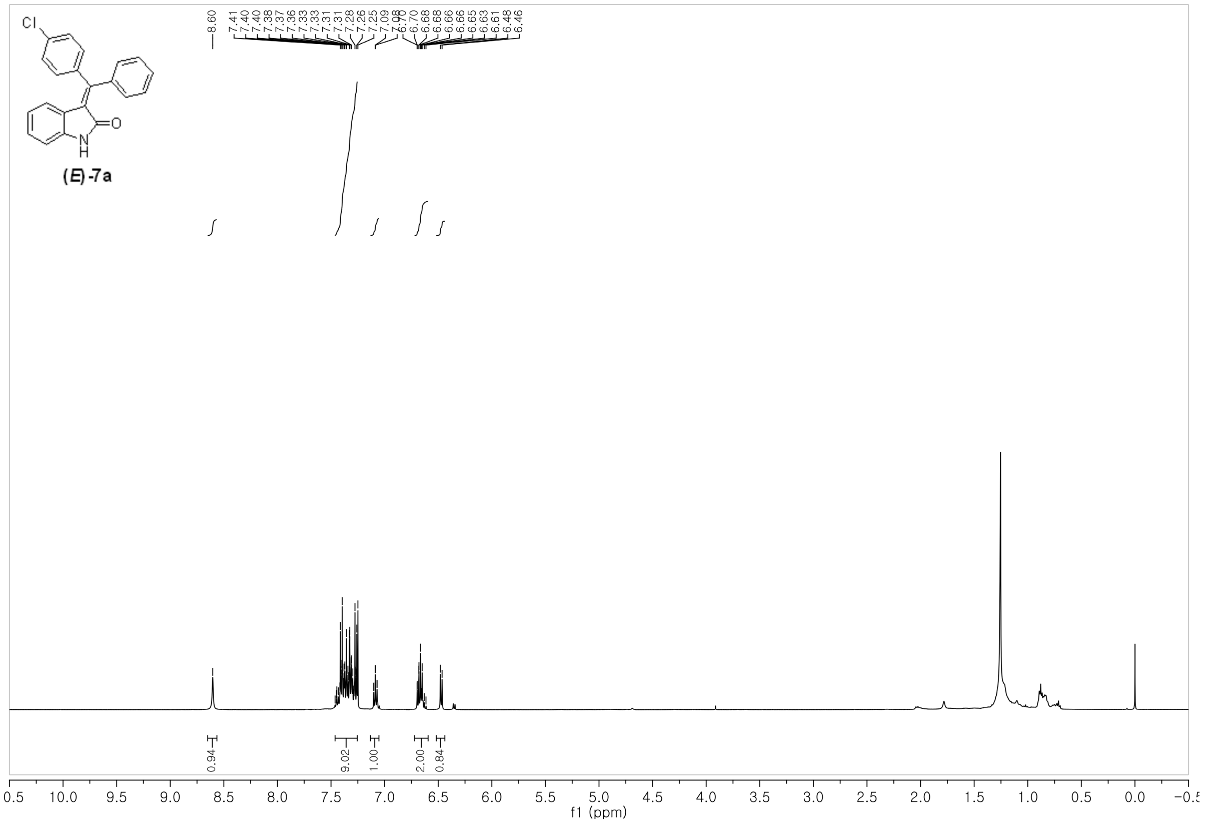

^{13}C NMR spectrum of (*E*)-7a

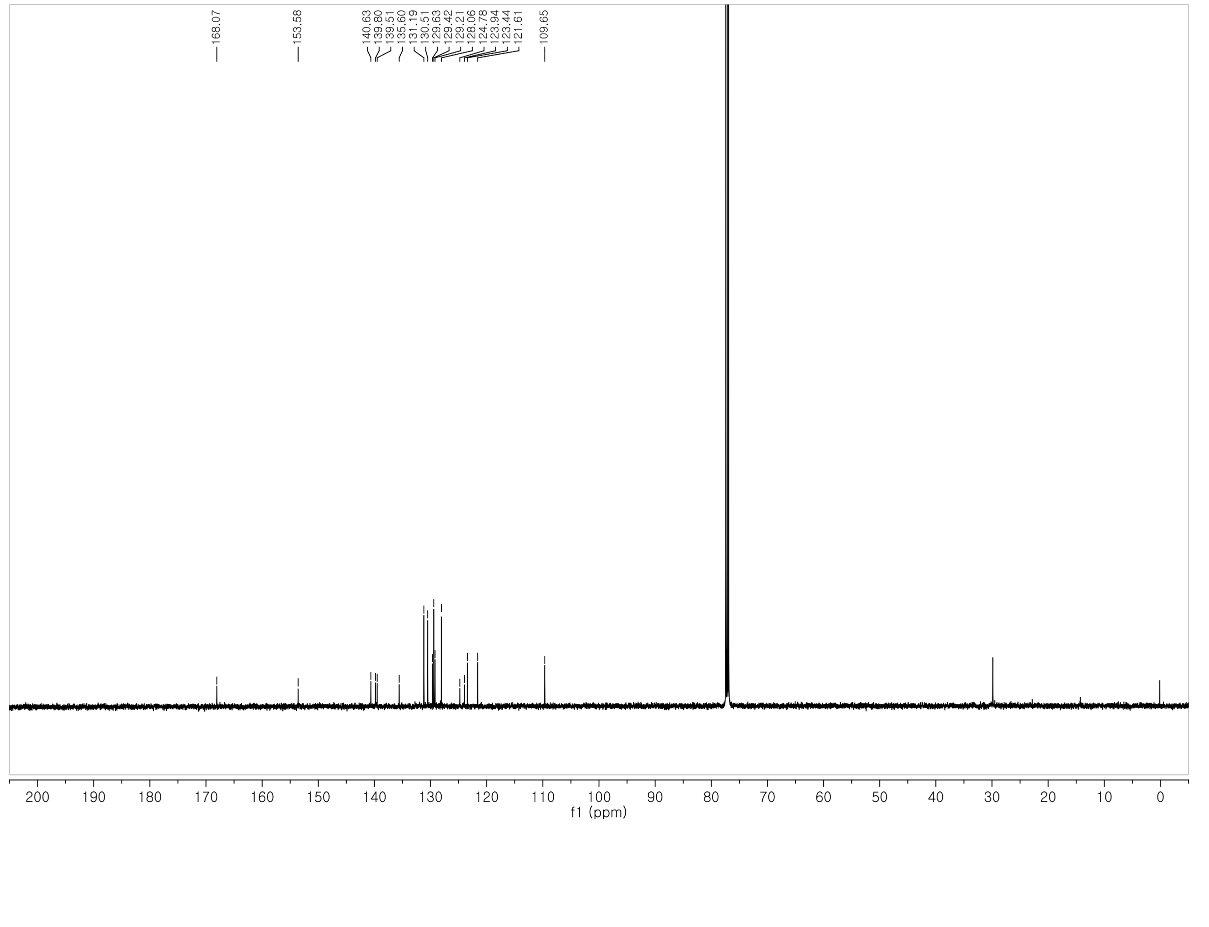

HSQC spectrum of (*E*)-7a

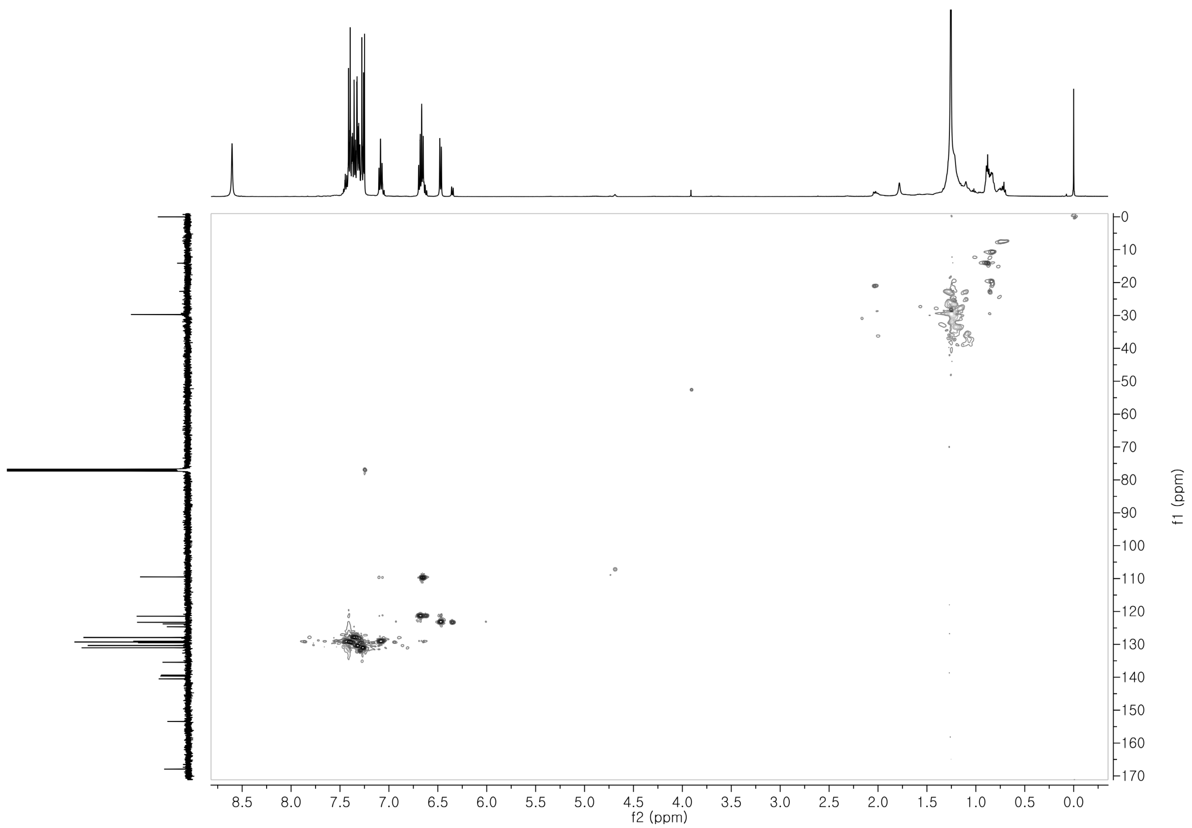

HMBC spectrum of (*E*)-7a

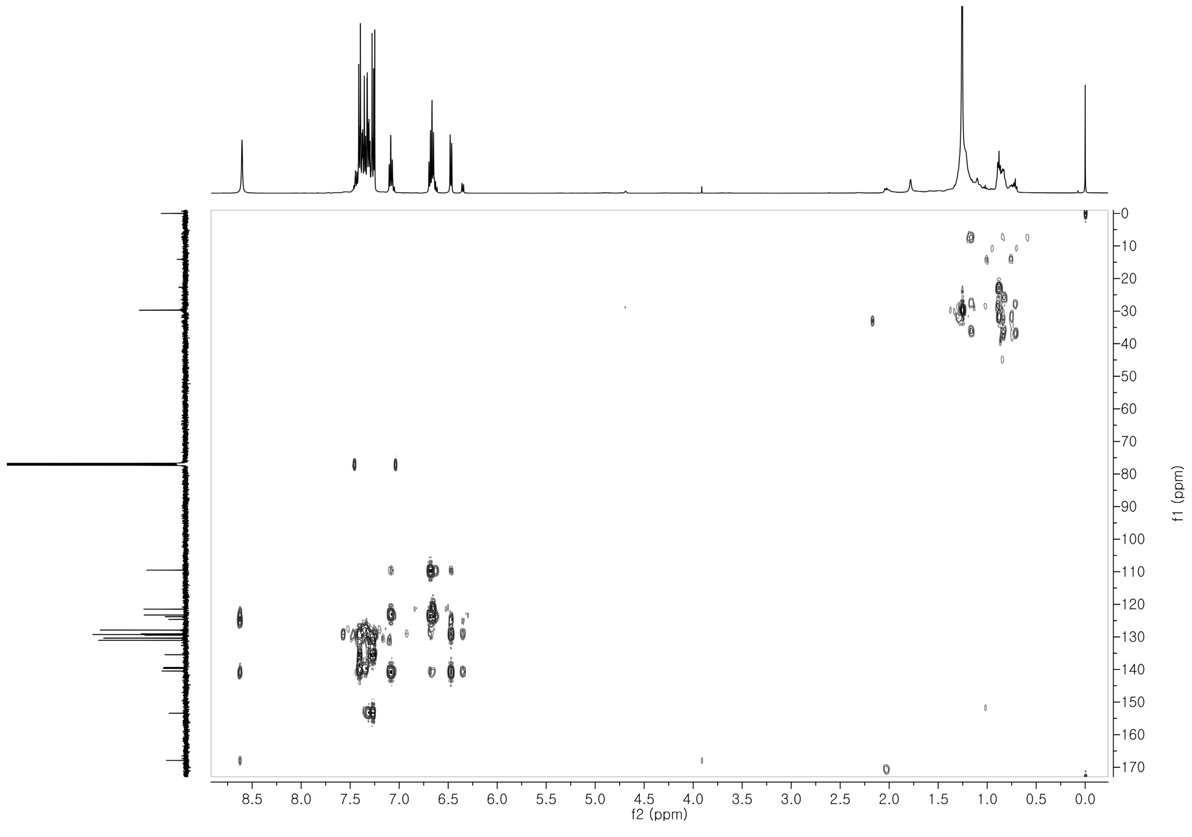

COSY spectrum of (*E*)-7a

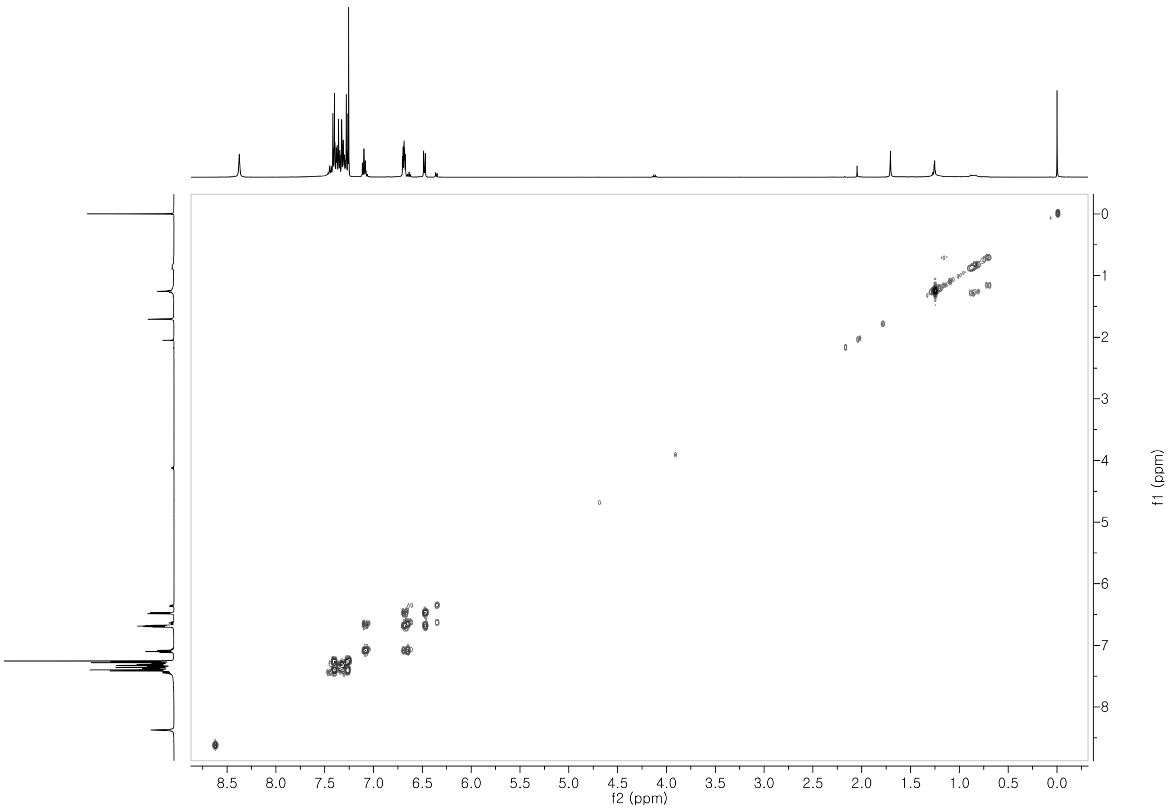

ROESY spectrum of (*E*)-7a

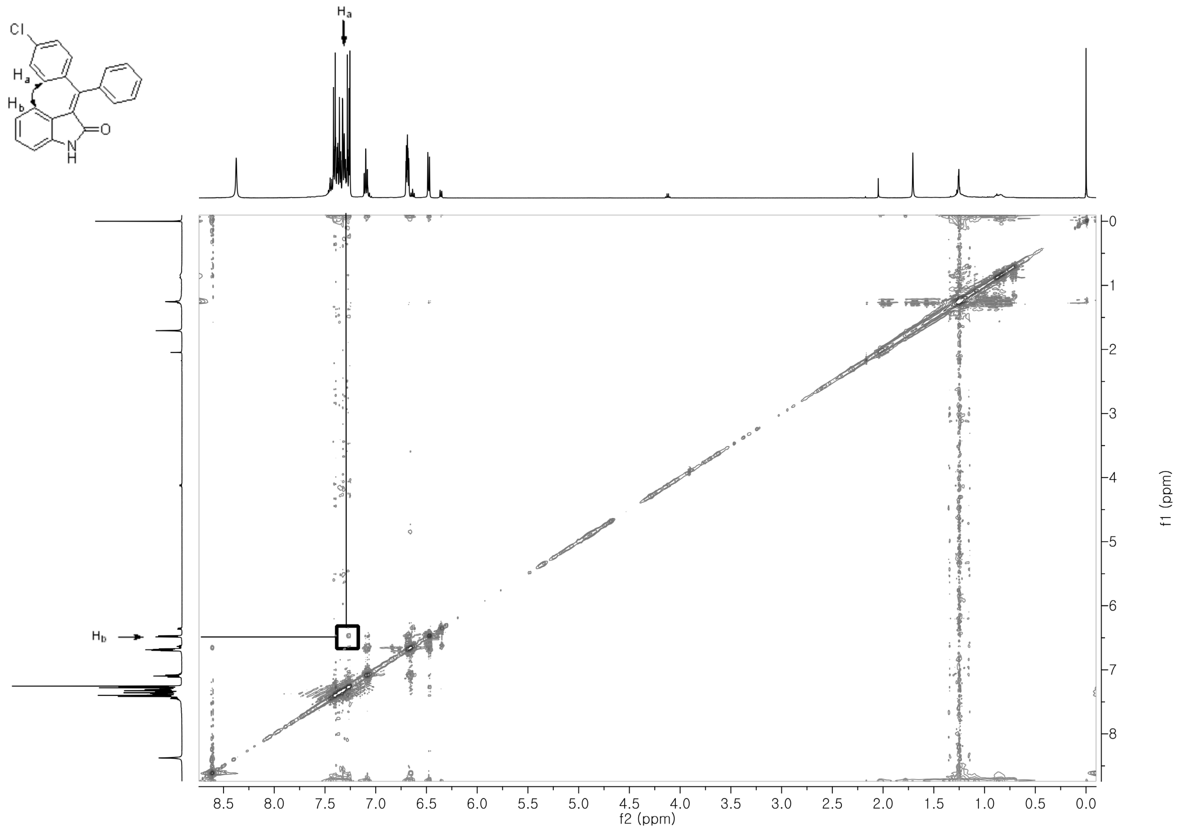

(*Z*)-1-benzyl-3-((4-chlorophenyl)(phenyl)methylene)indolin-2-one ((*Z*)-7b)

^{1}H NMR spectrum of (*Z*)-7b

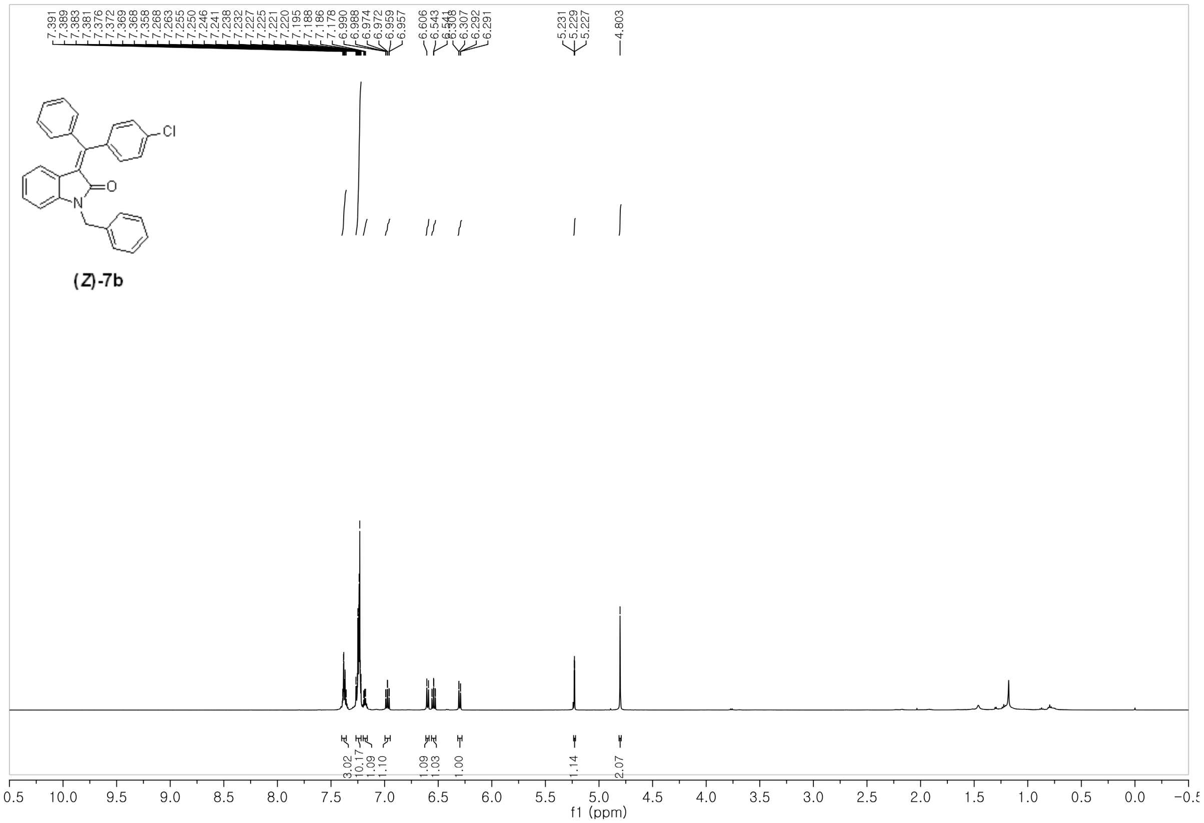

^{13}C NMR spectrum of (*Z*)-7b

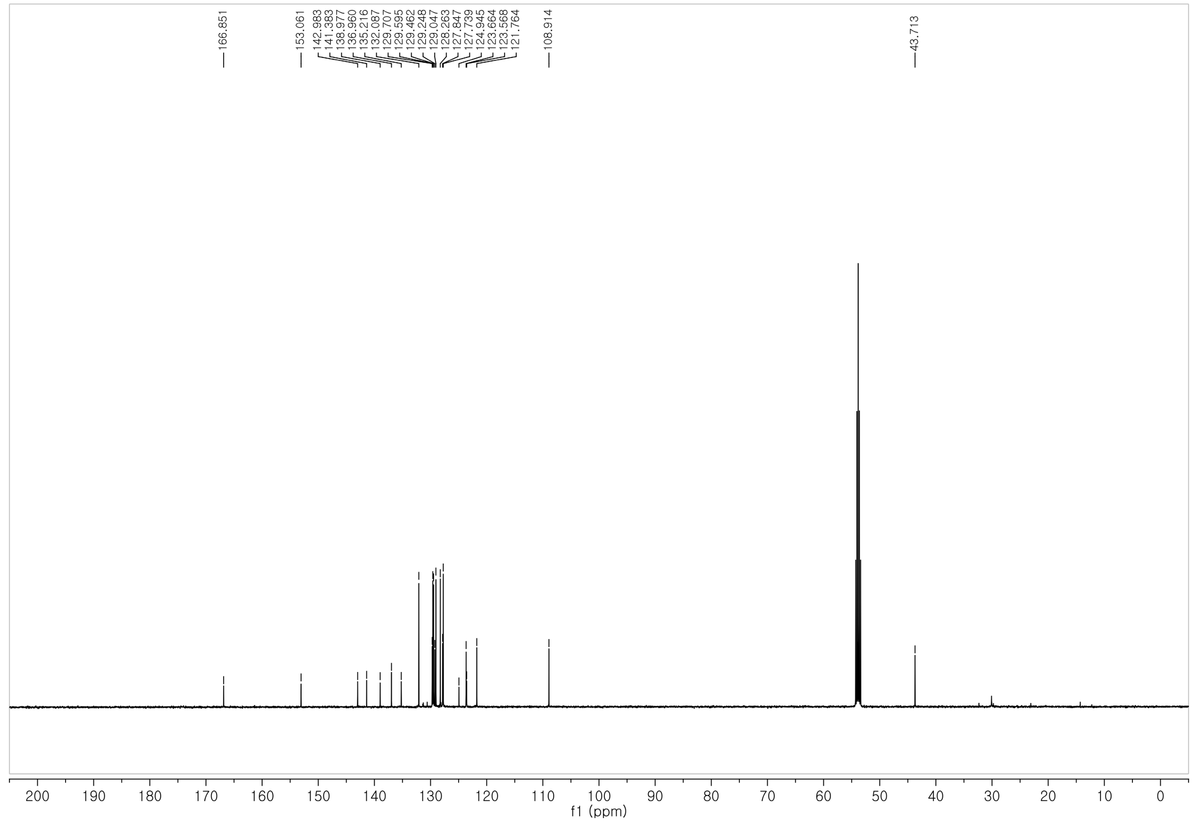

HSQC spectrum of (*Z*)-7b

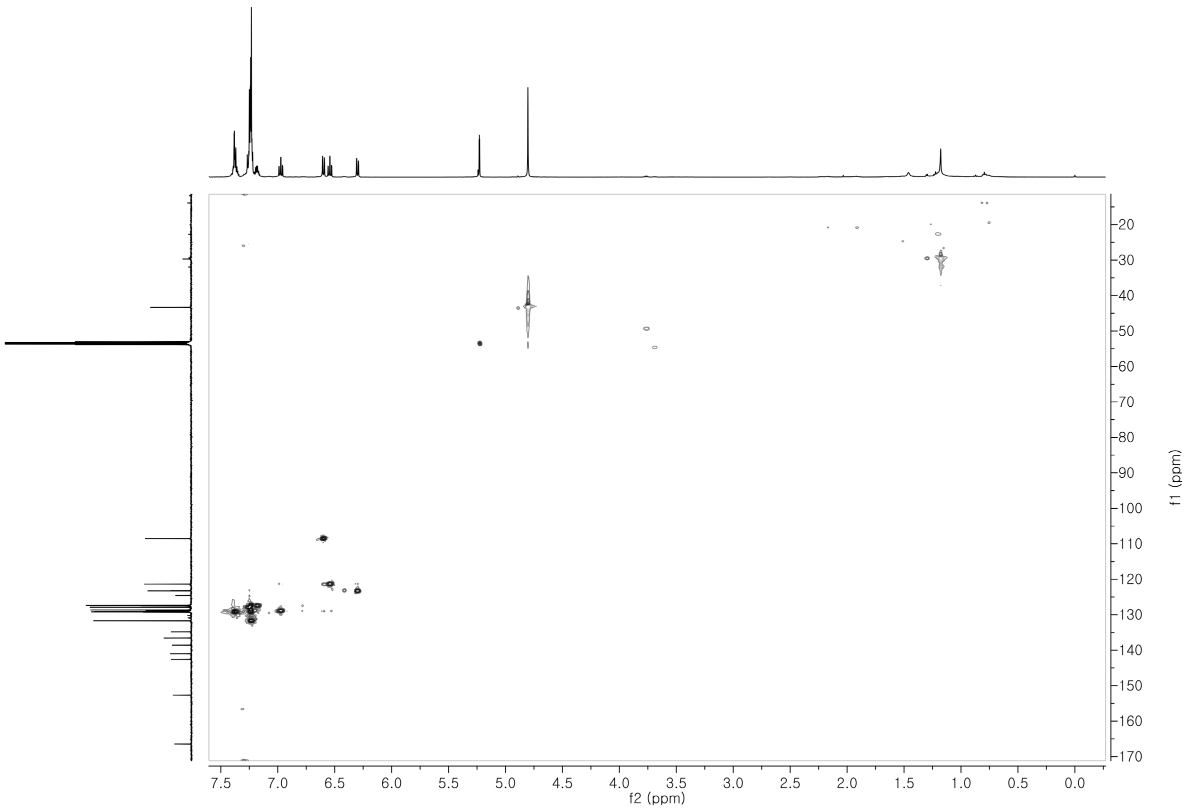

HMBC spectrum of (*Z*)-7b

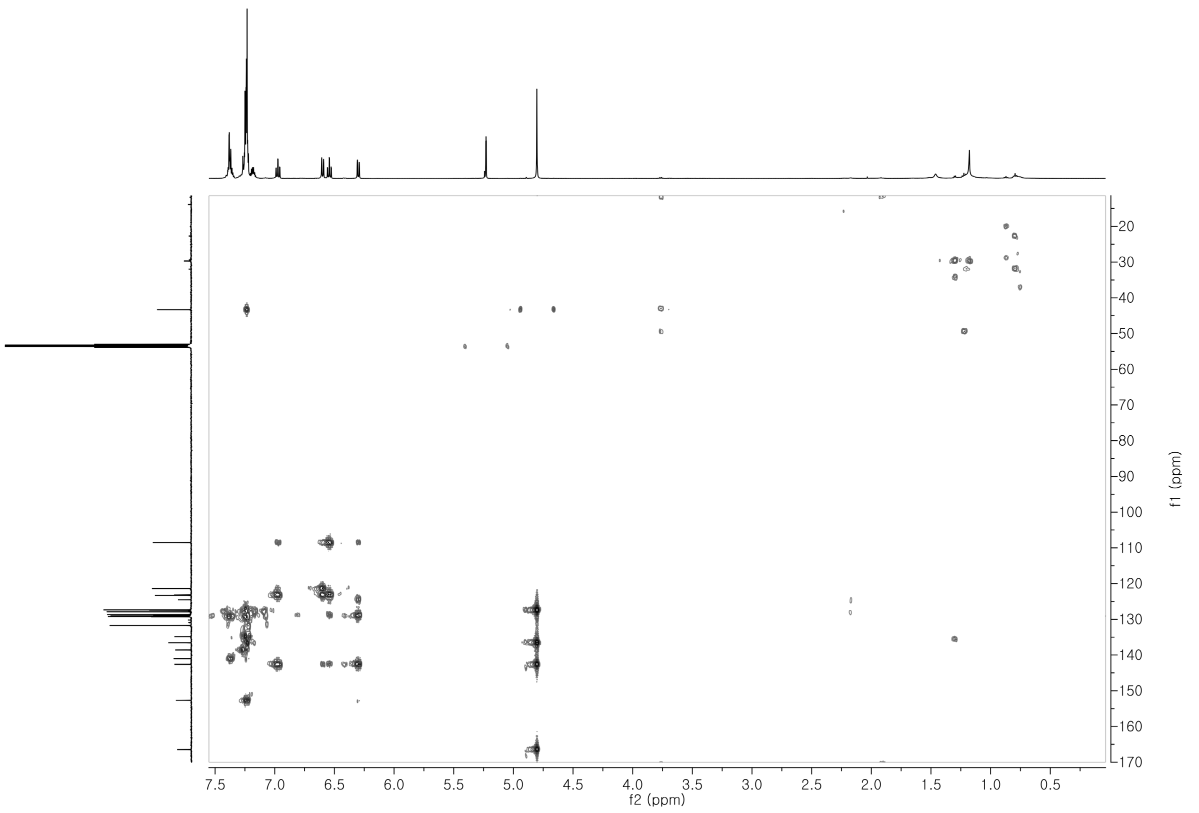

COSY spectrum of (*Z*)-7b

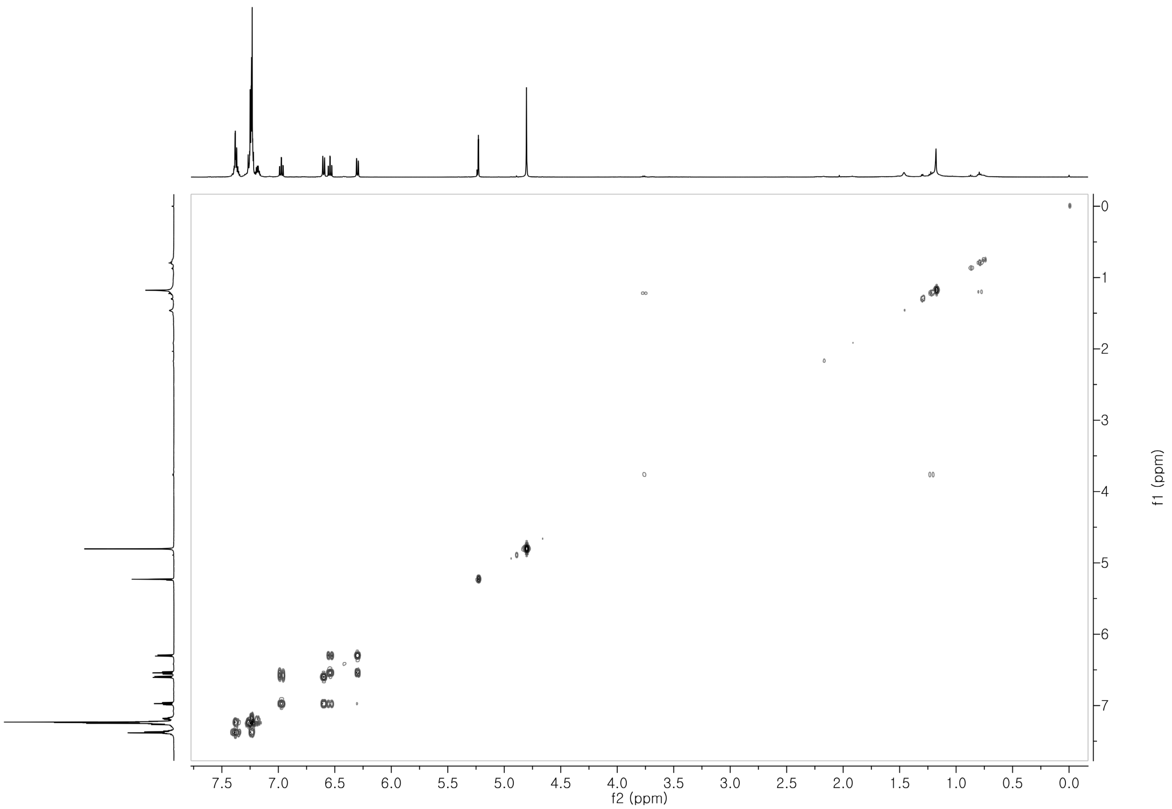

ROESY spectrum of (*Z*)-7b

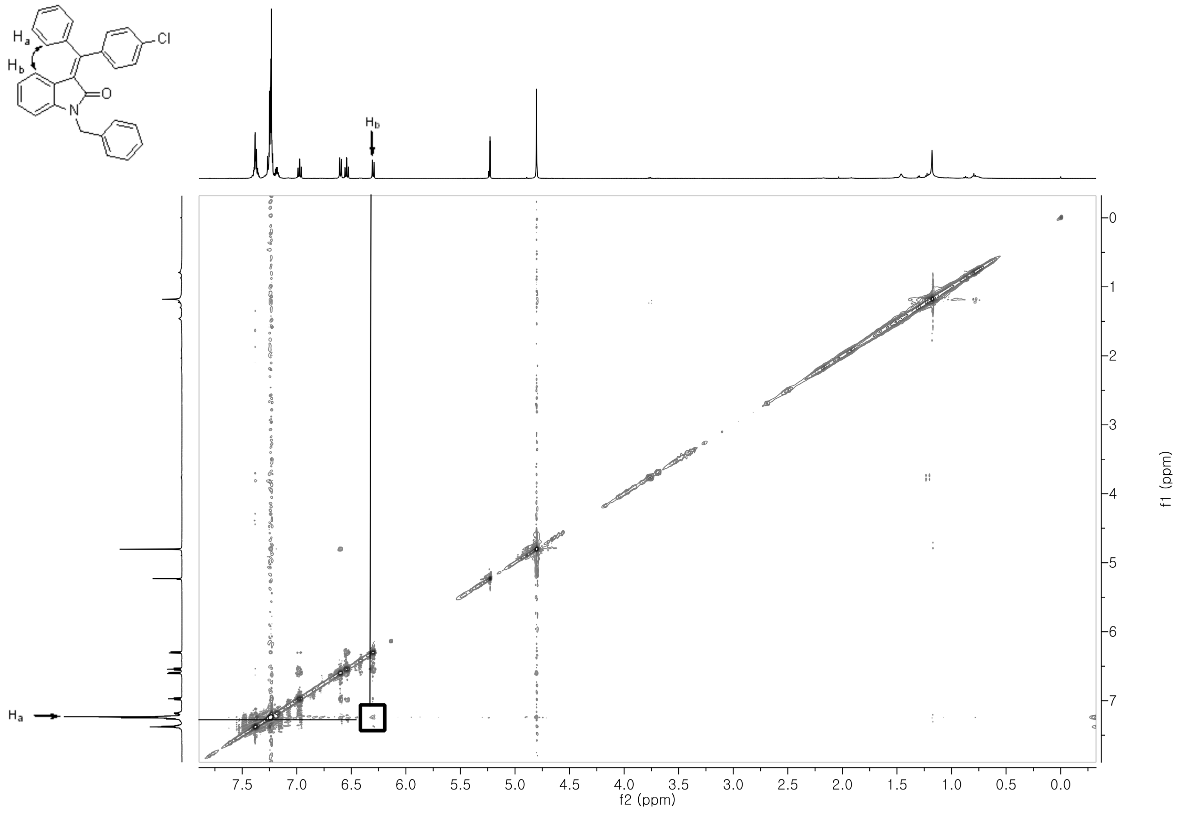

(*E*)-1-benzyl-3-((4-chlorophenyl)(phenyl)methylene)indolin-2-one ((*E*)-7b)

^{1}H NMR spectrum of (*E*)-7b

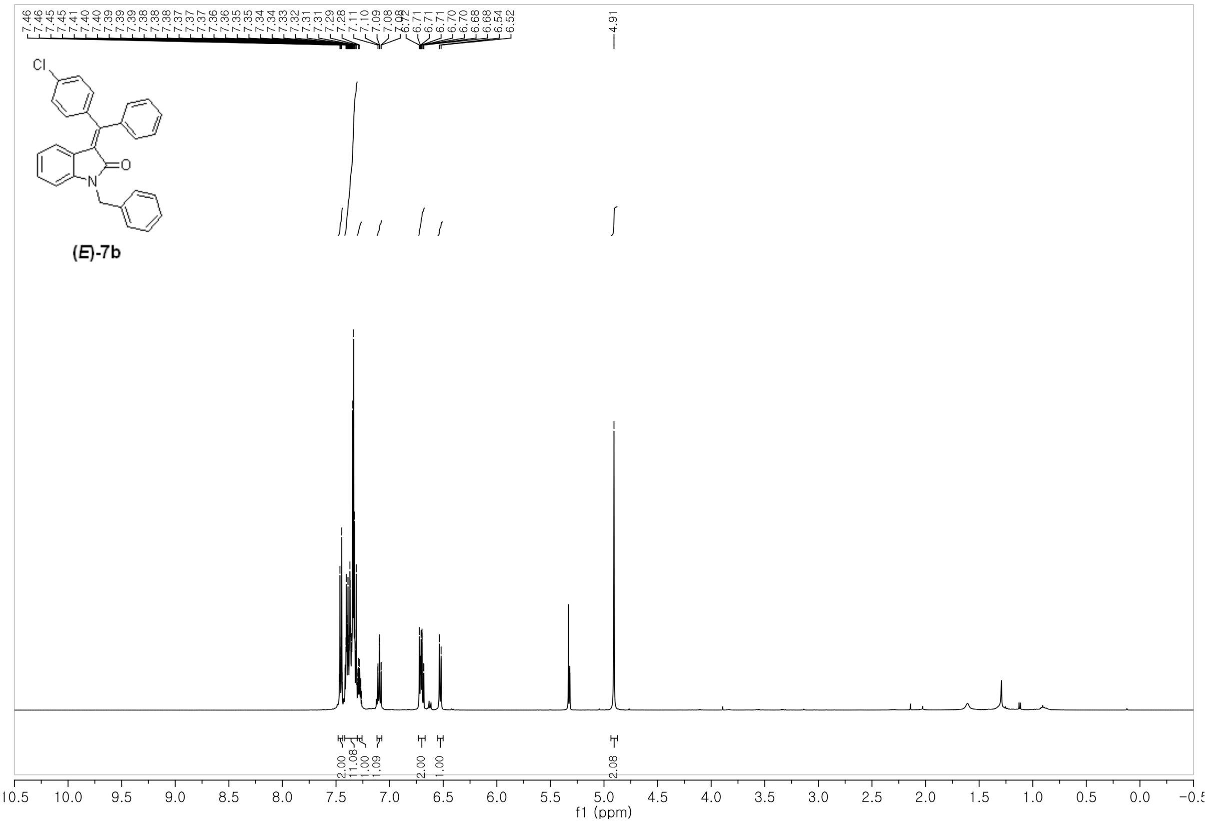

^{13}C NMR spectrum of (*E*)-7b

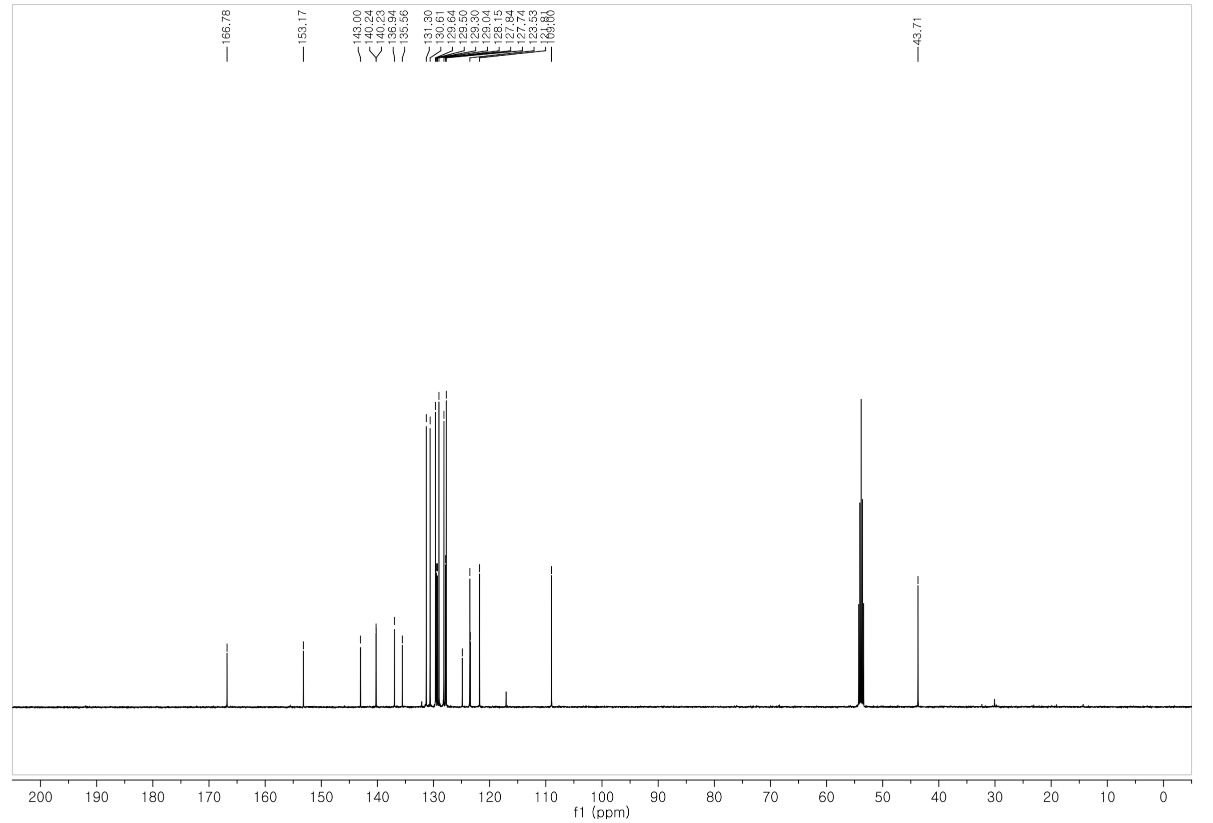

HSQC spectrum of (*E*)-7b

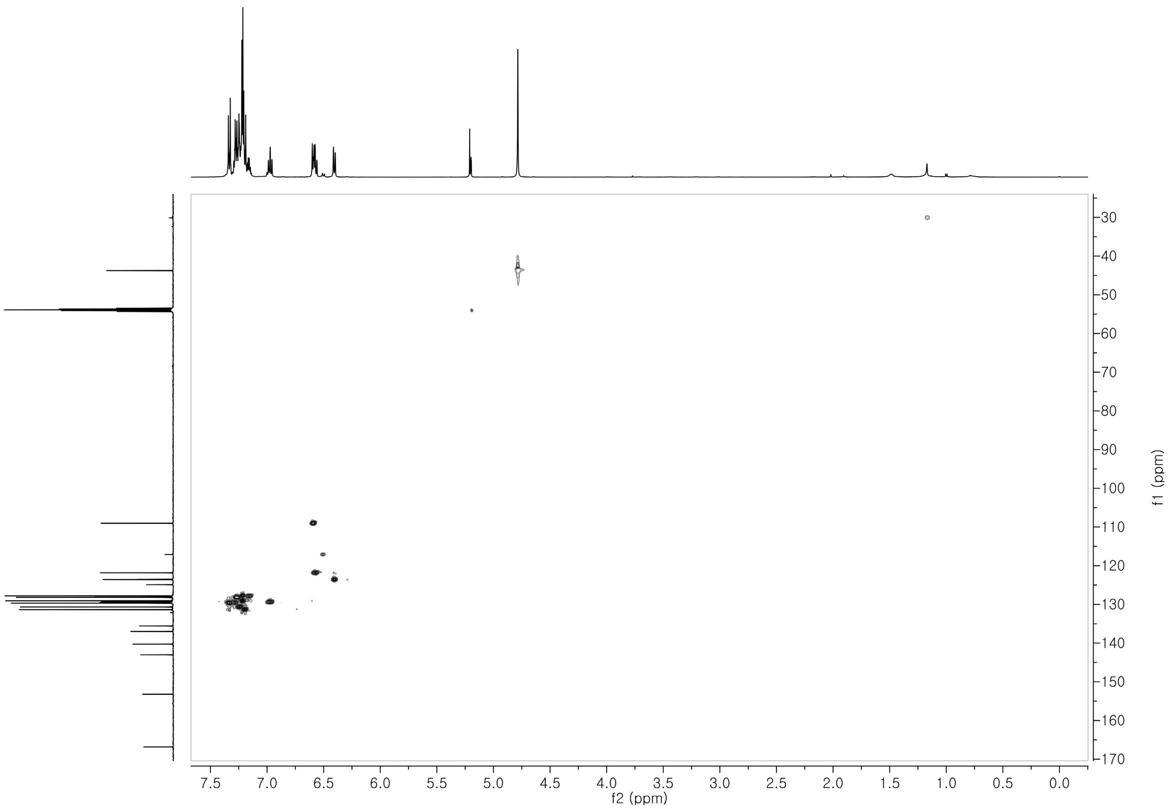

HMBC spectrum of (*E*)-7b

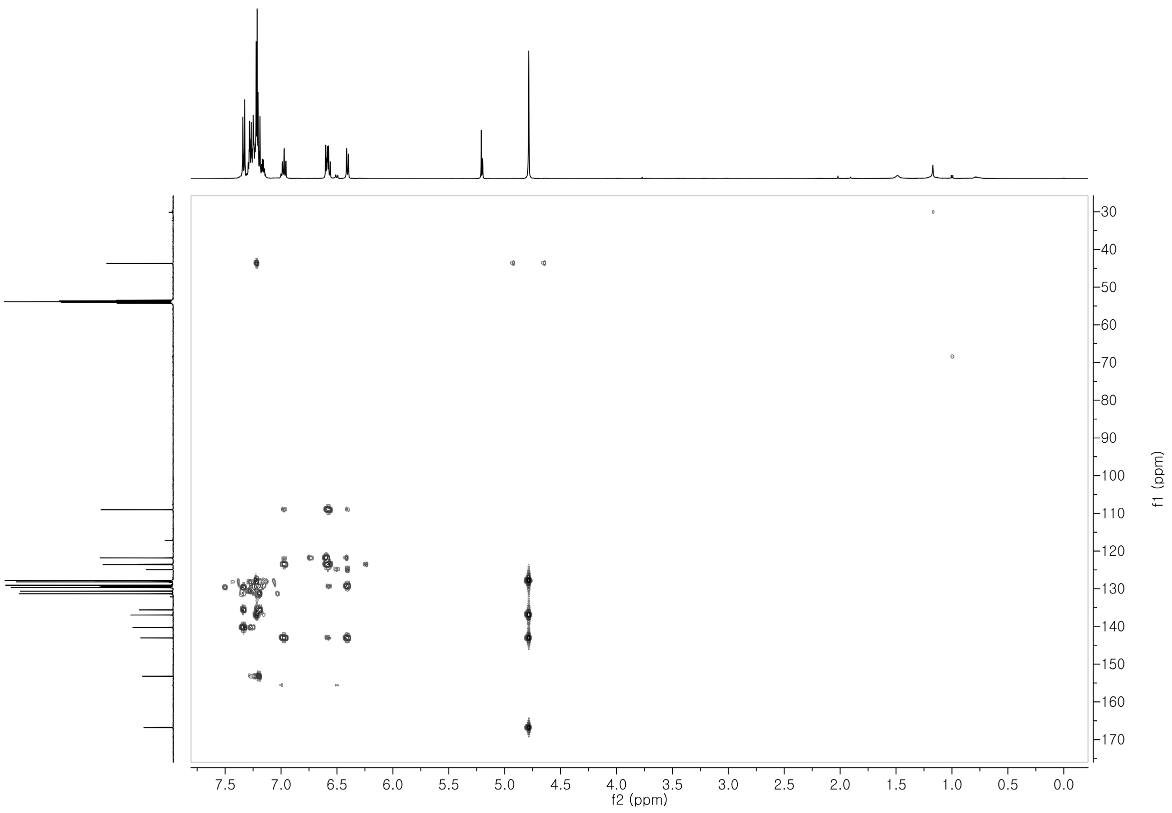

COSY spectrum of (*E*)-7b

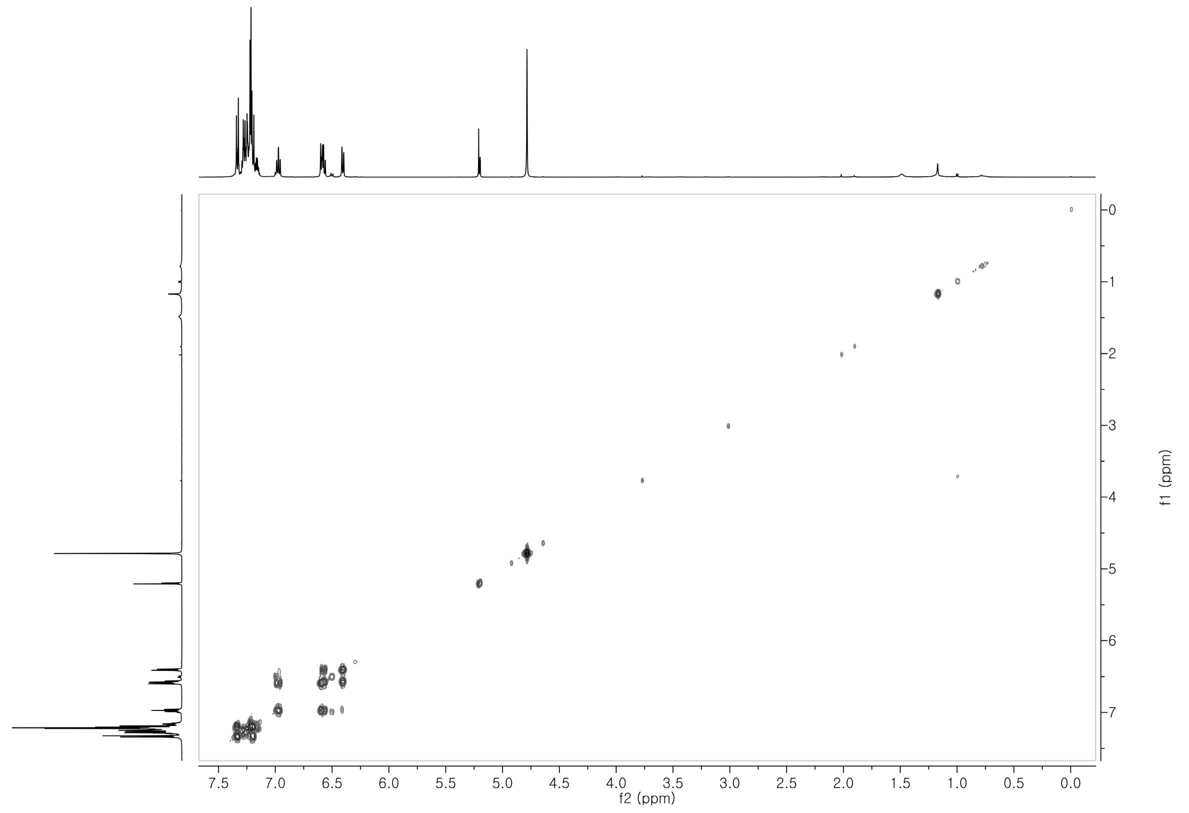

ROESY spectrum of (*E*)-7b

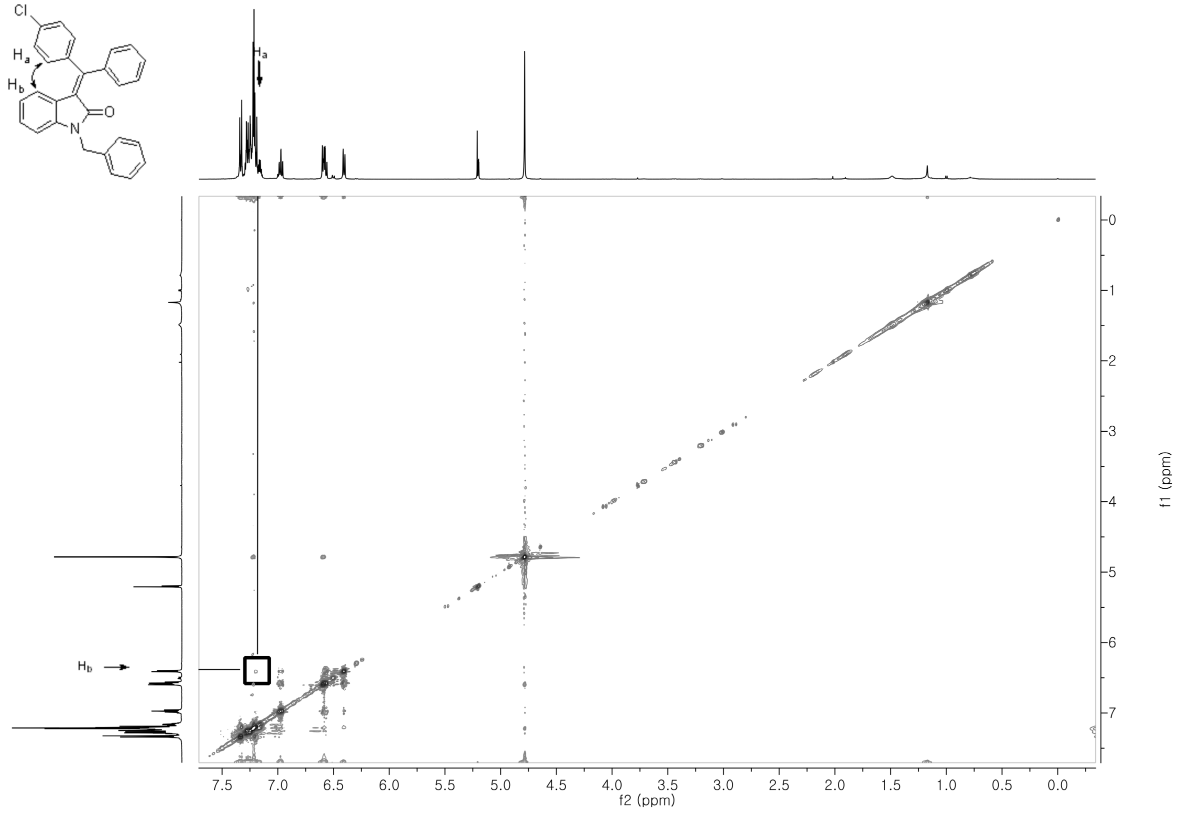

(*E*)-4-((4-chlorophenyl)(phenyl)methylene)-2-methyl-1,2-dihydroisoquinolin-3(4*H*)-one ((*E*)-7c)

^{1}H NMR spectrum of (*E*)-7c

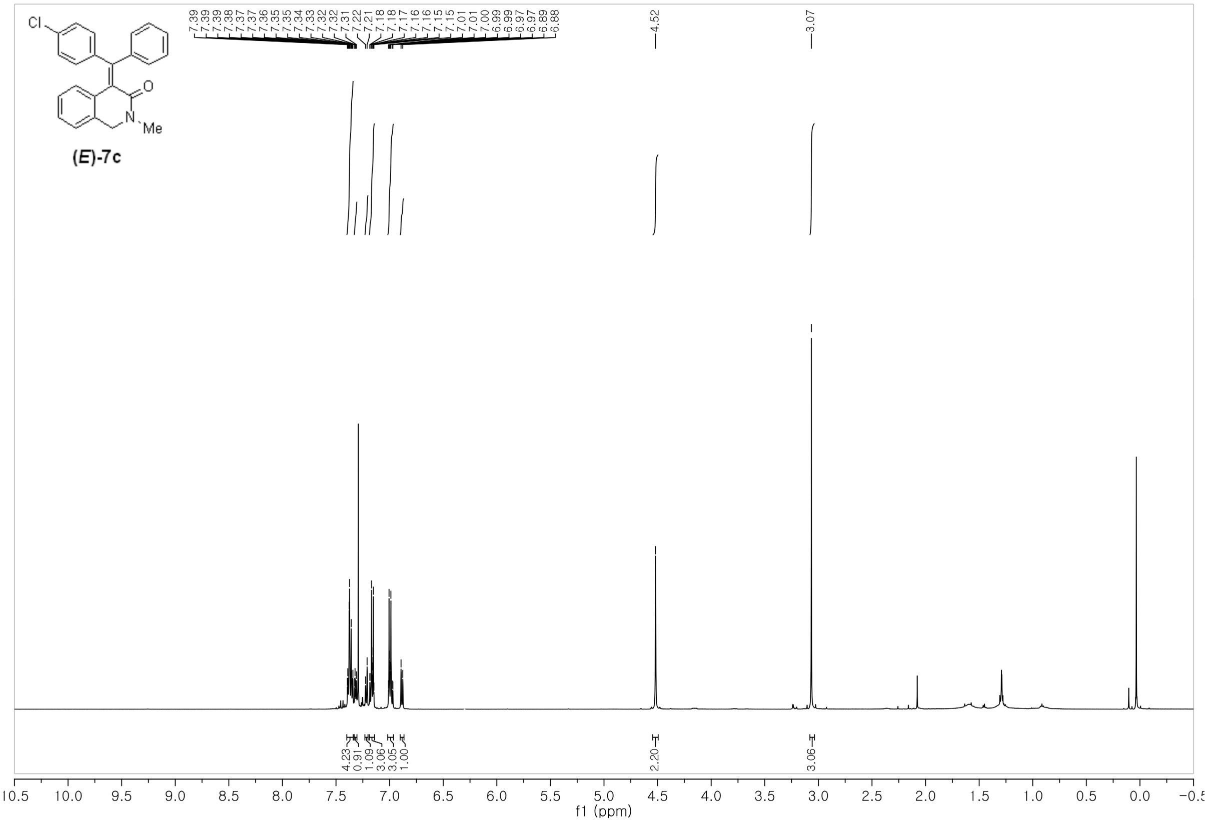

^{13}C NMR spectrum of (*E*)-7c

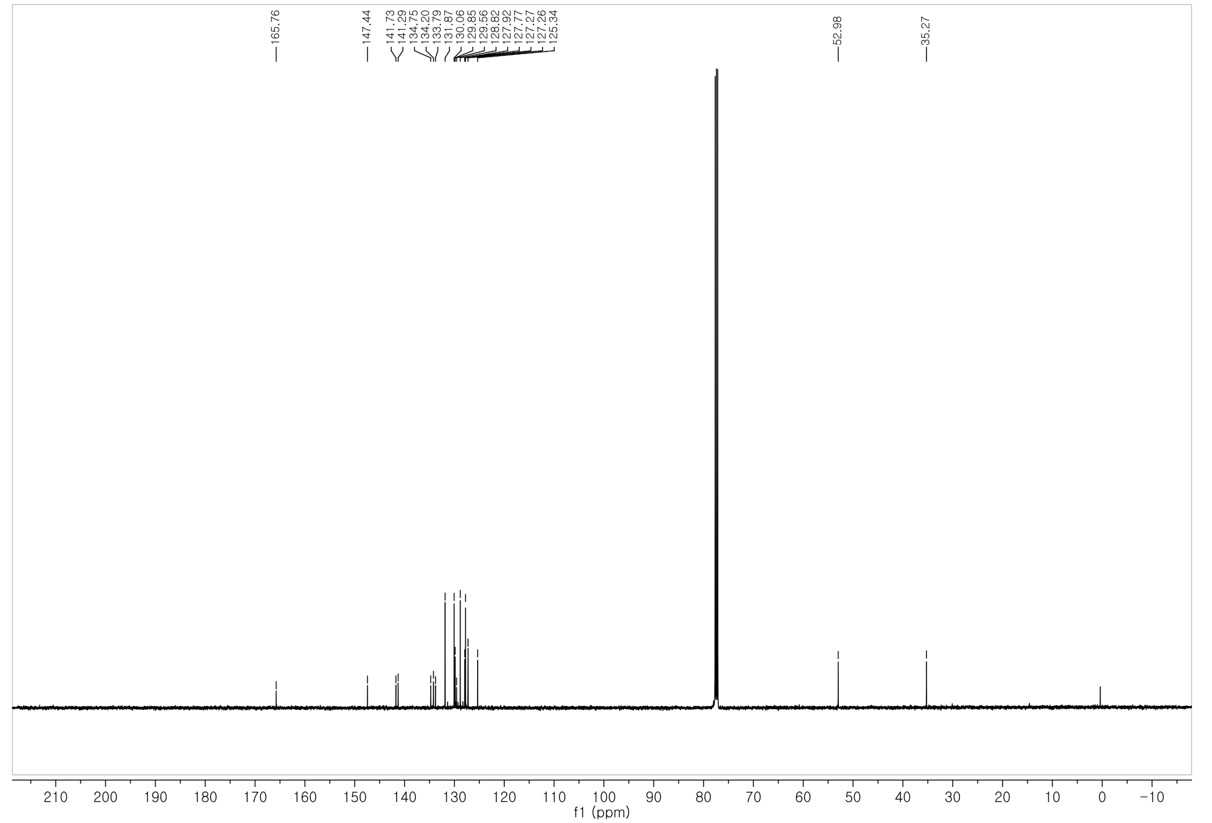

HSQC spectrum of (*E*)-7c

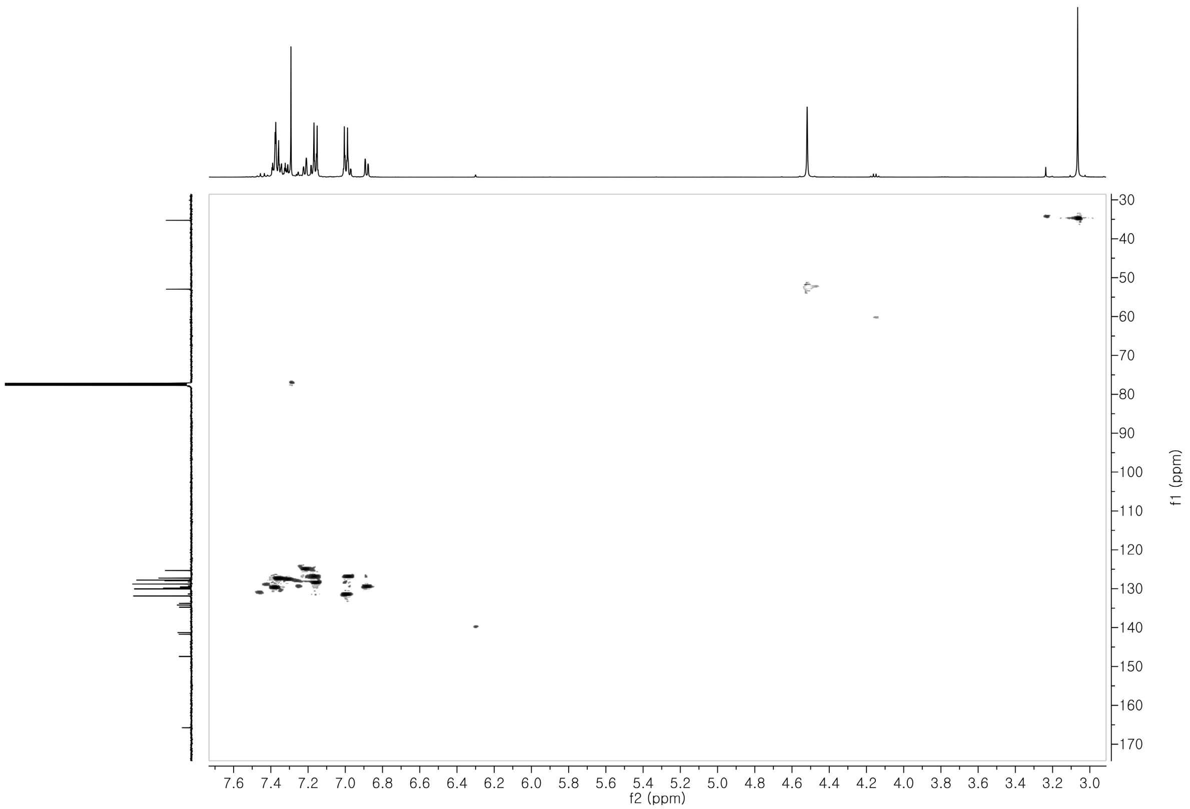

HMBC spectrum of (*E*)-7c

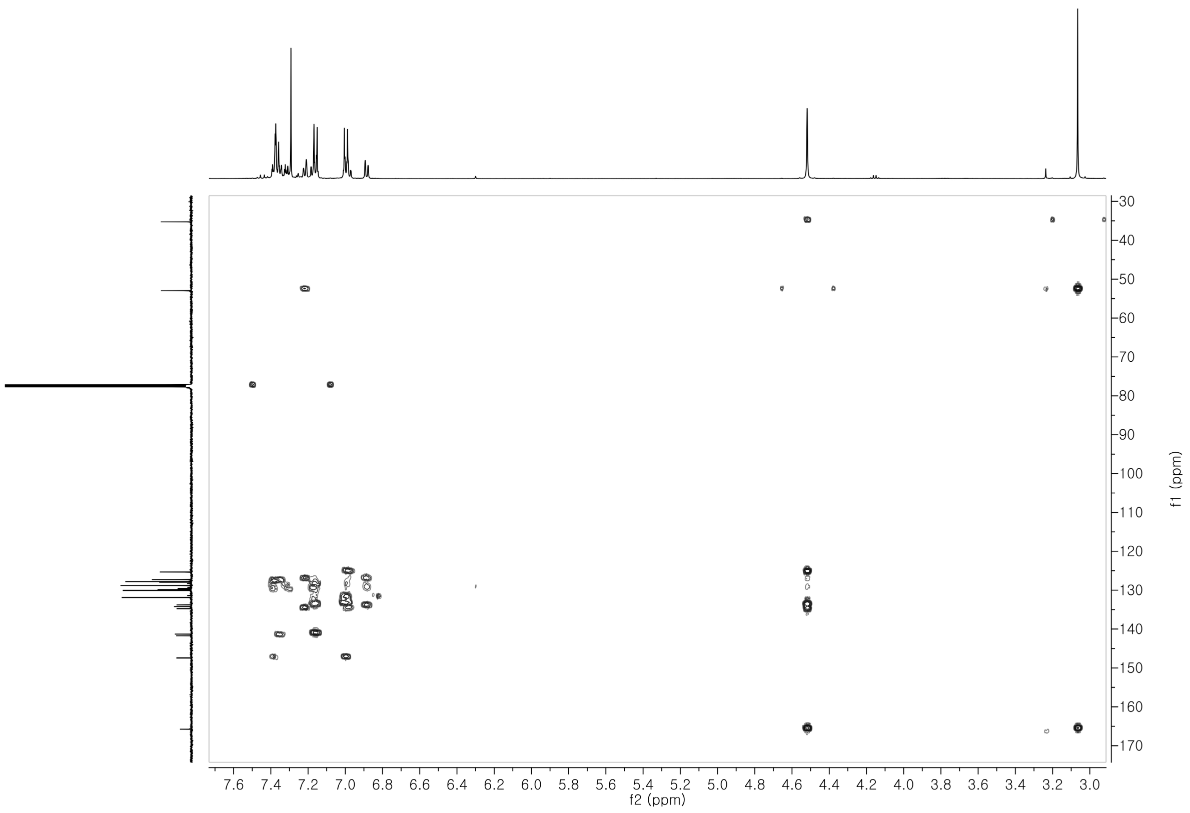

COSY spectrum of (*E*)-7c

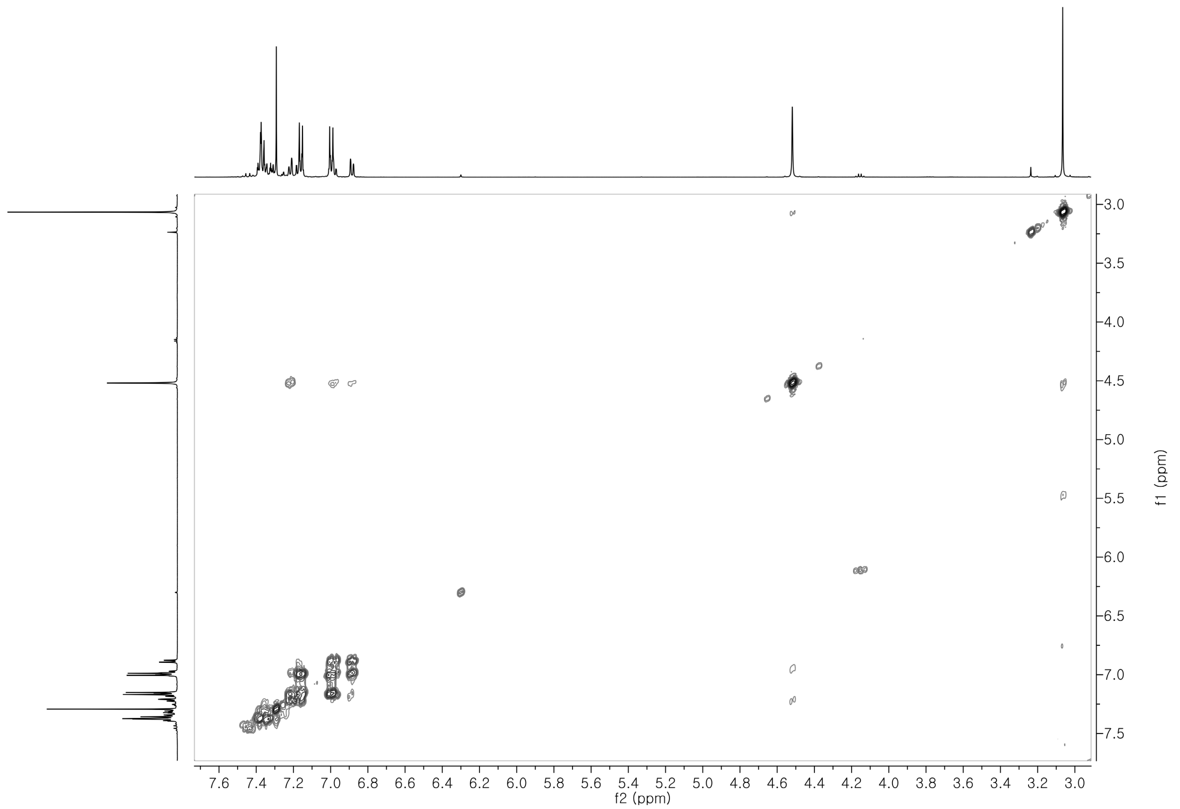

ROESY spectrum of (*E*)-7c

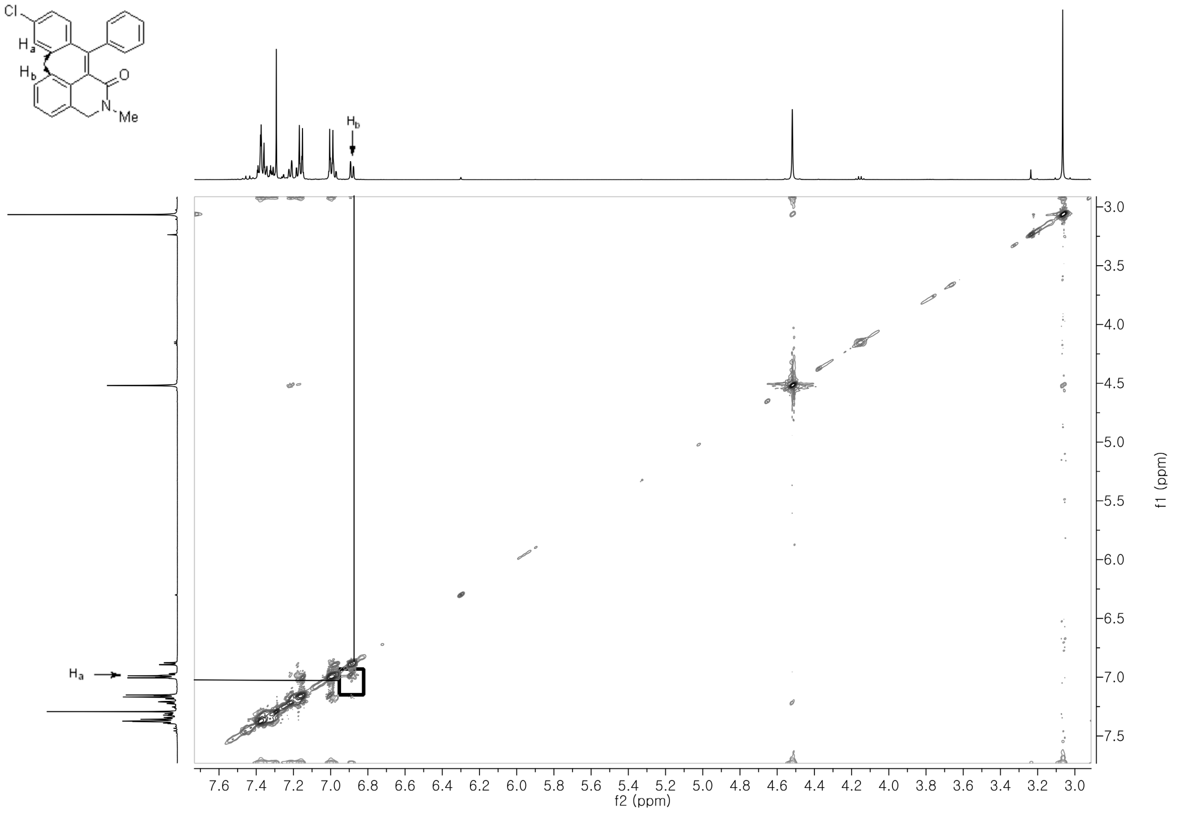

(*Z*)-4-((4-chlorophenyl)(phenyl)methylene)-2-methyl-1,2-dihydroisoquinolin-3(4*H*)-one ((*Z*)-7c)

^{1}H NMR spectrum of (*Z*)-7c

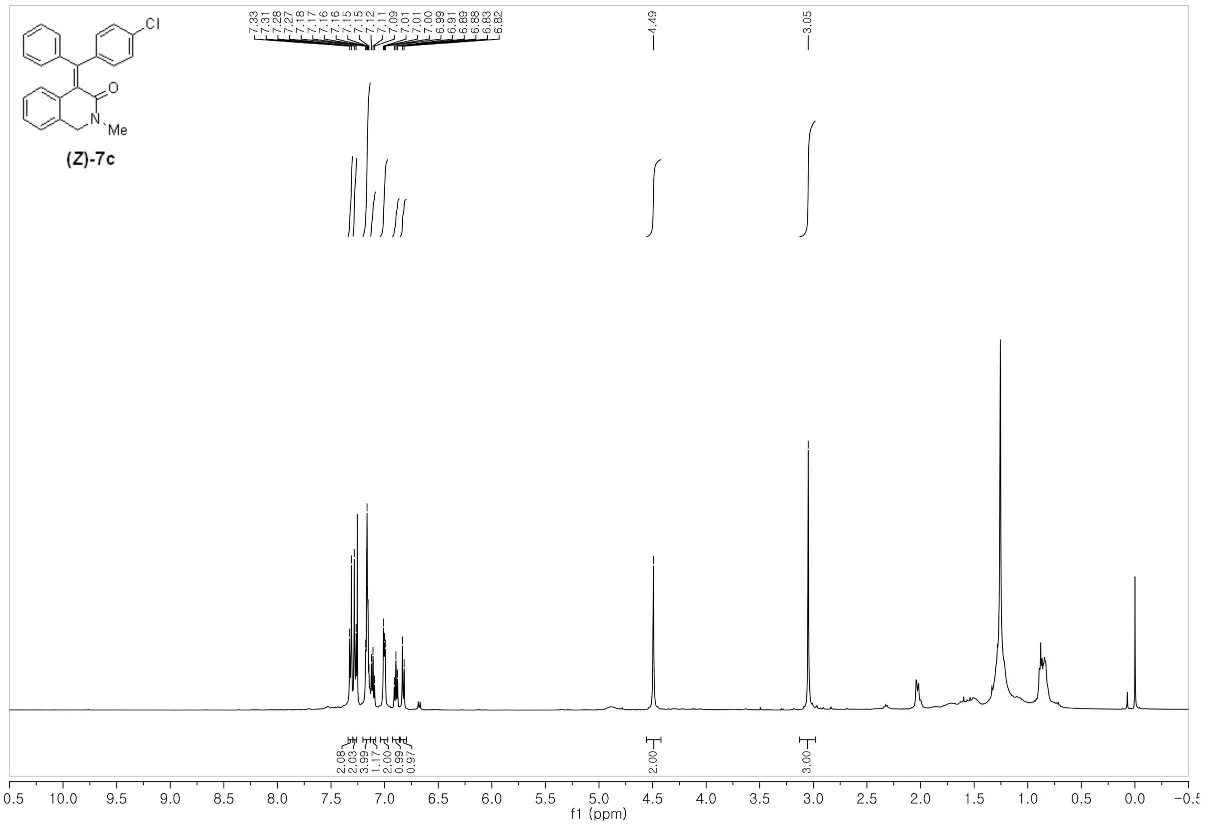

^{13}C NMR spectrum of (*Z*)-7c

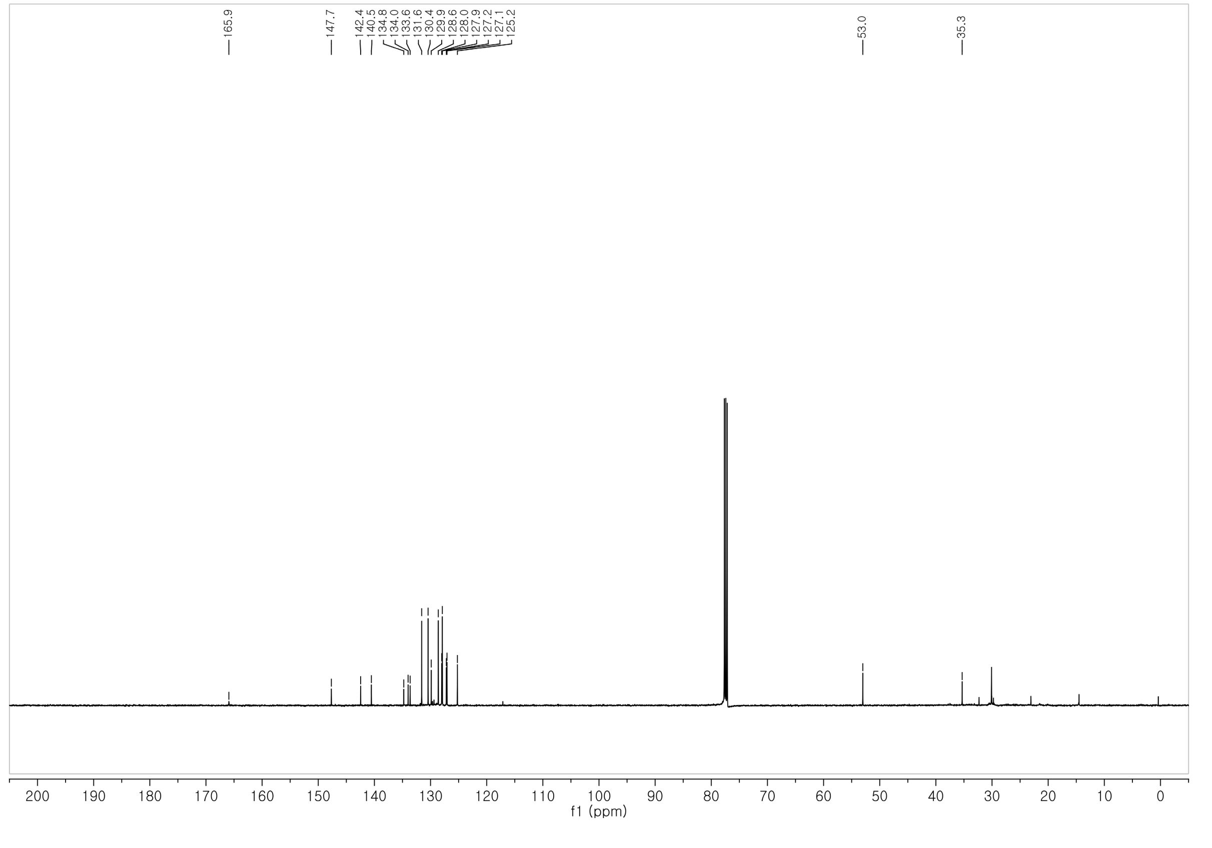

HSQC spectrum of (*Z*)-7c

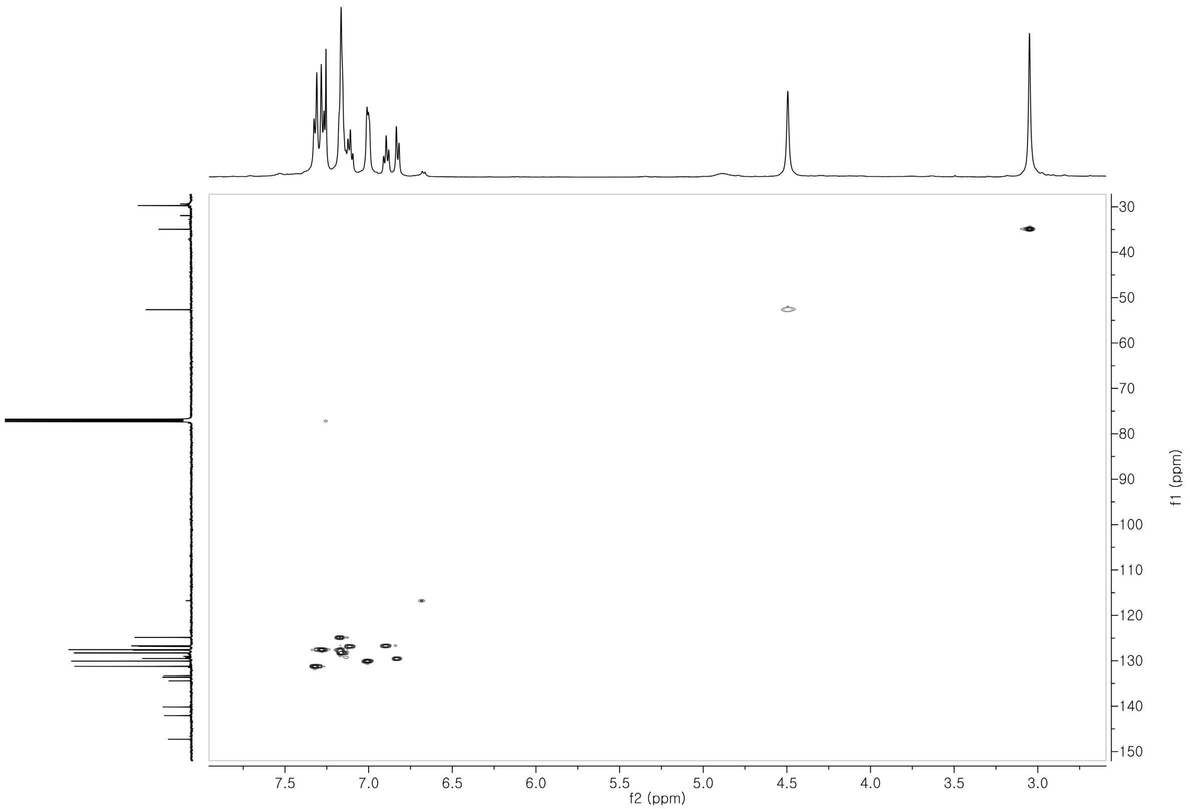

HMBC spectrum of (*Z*)-7c

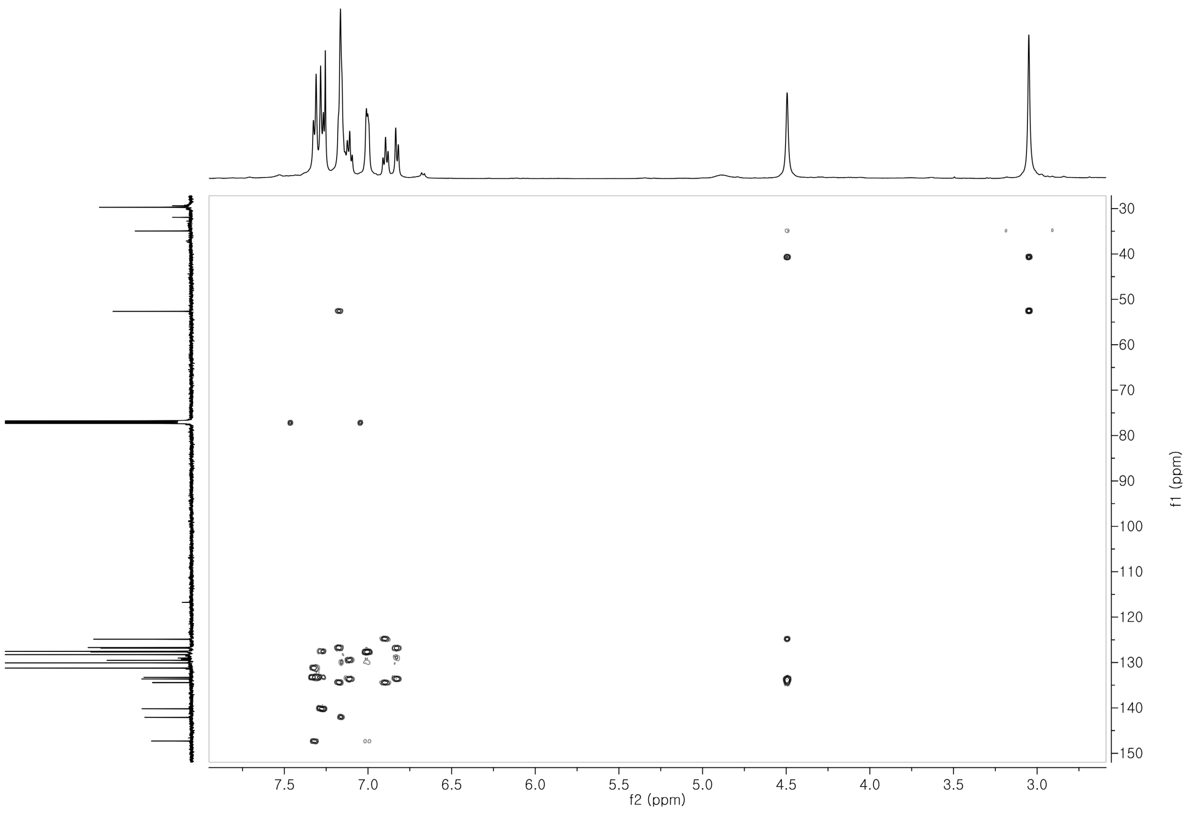

COSY spectrum of (*Z*)-7c

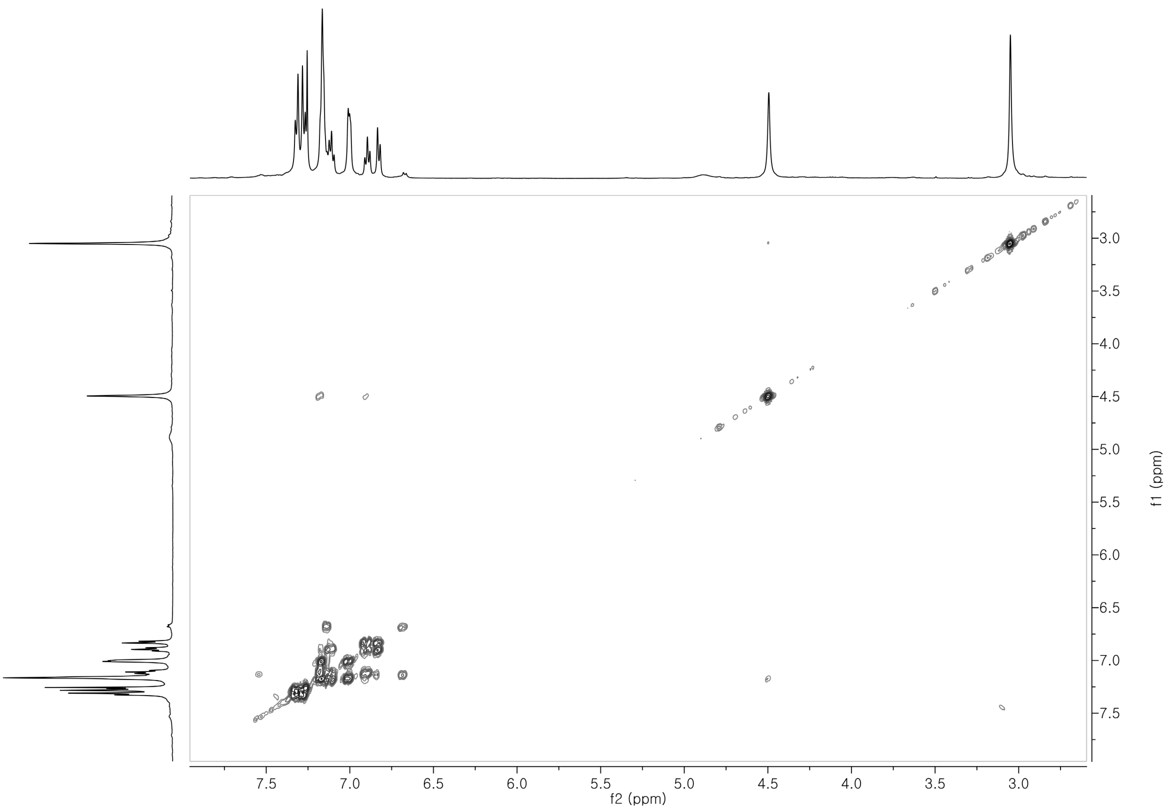

ROESY spectrum of (*Z*)-7c

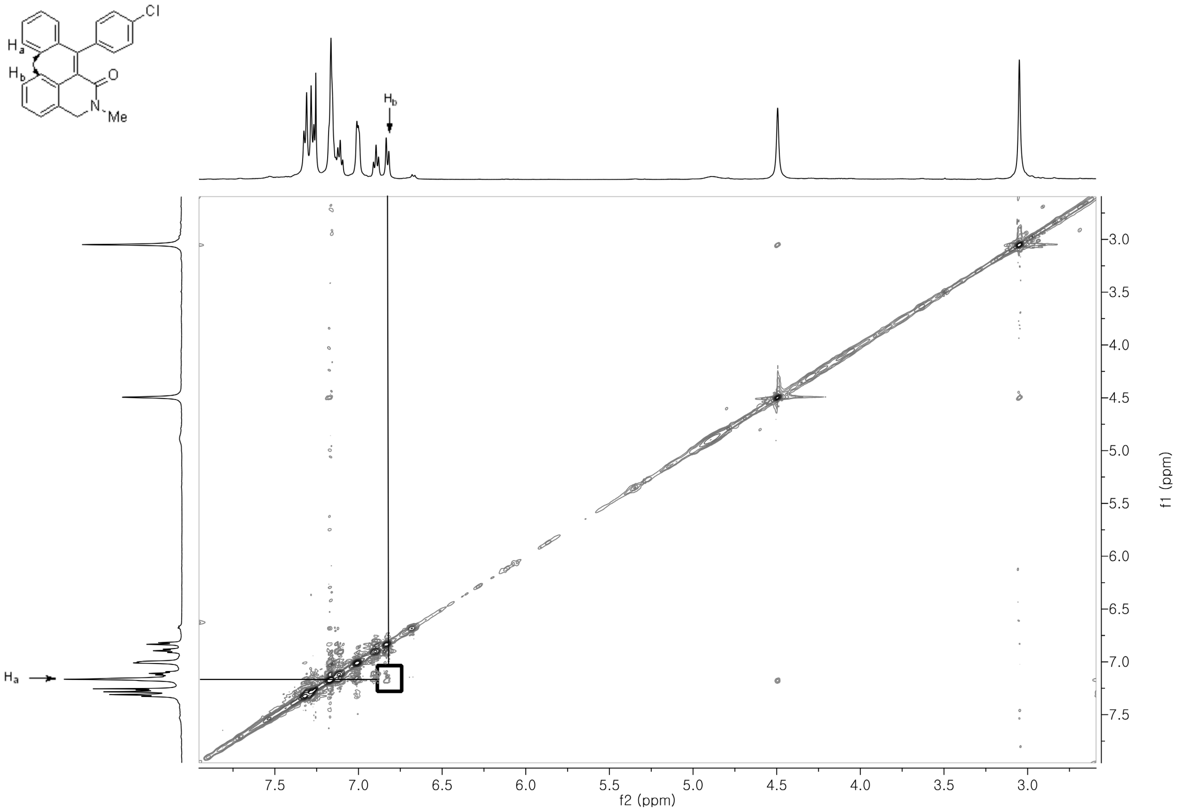

(*Z*)-3-((4-chlorophenyl)(phenyl)methylene)benzofuran-2(3H)-one ((*Z*)-7d)

^{1}H NMR spectrum of (*Z*)-7d

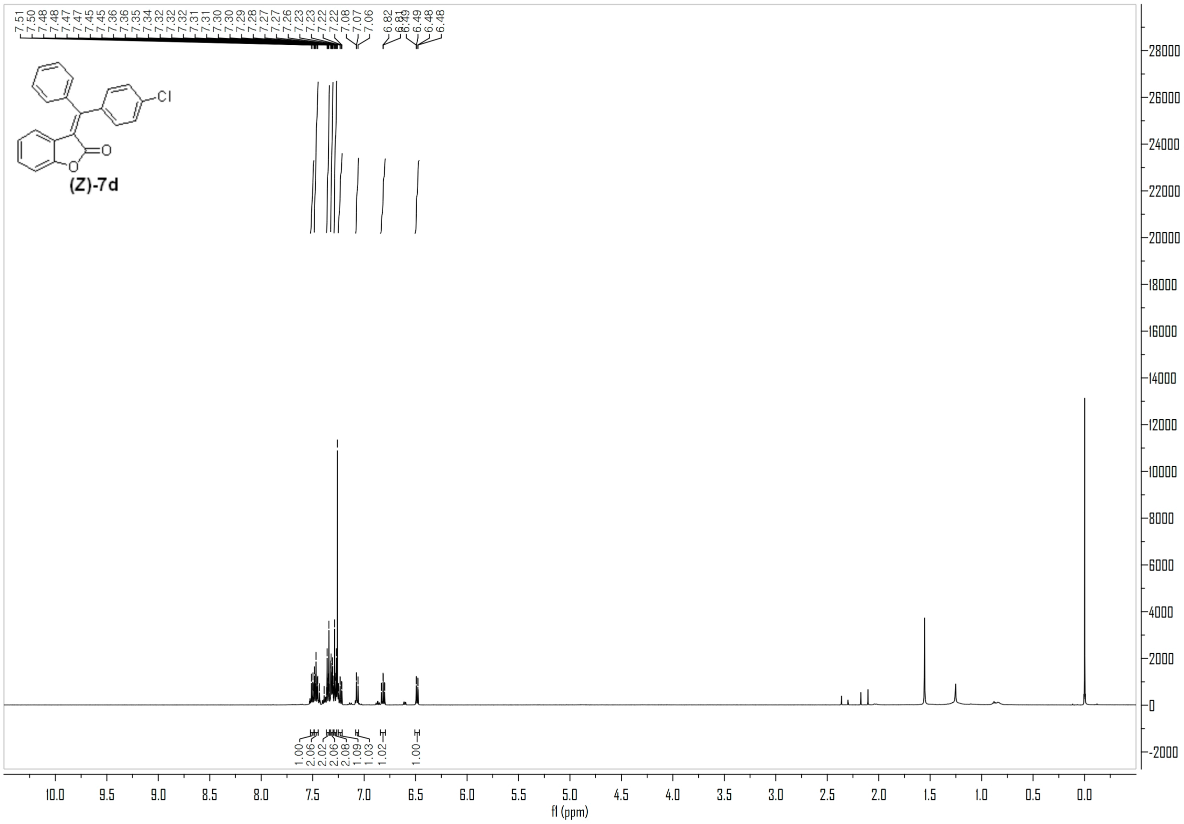

^{13}C NMR spectrum of (*Z*)-7d

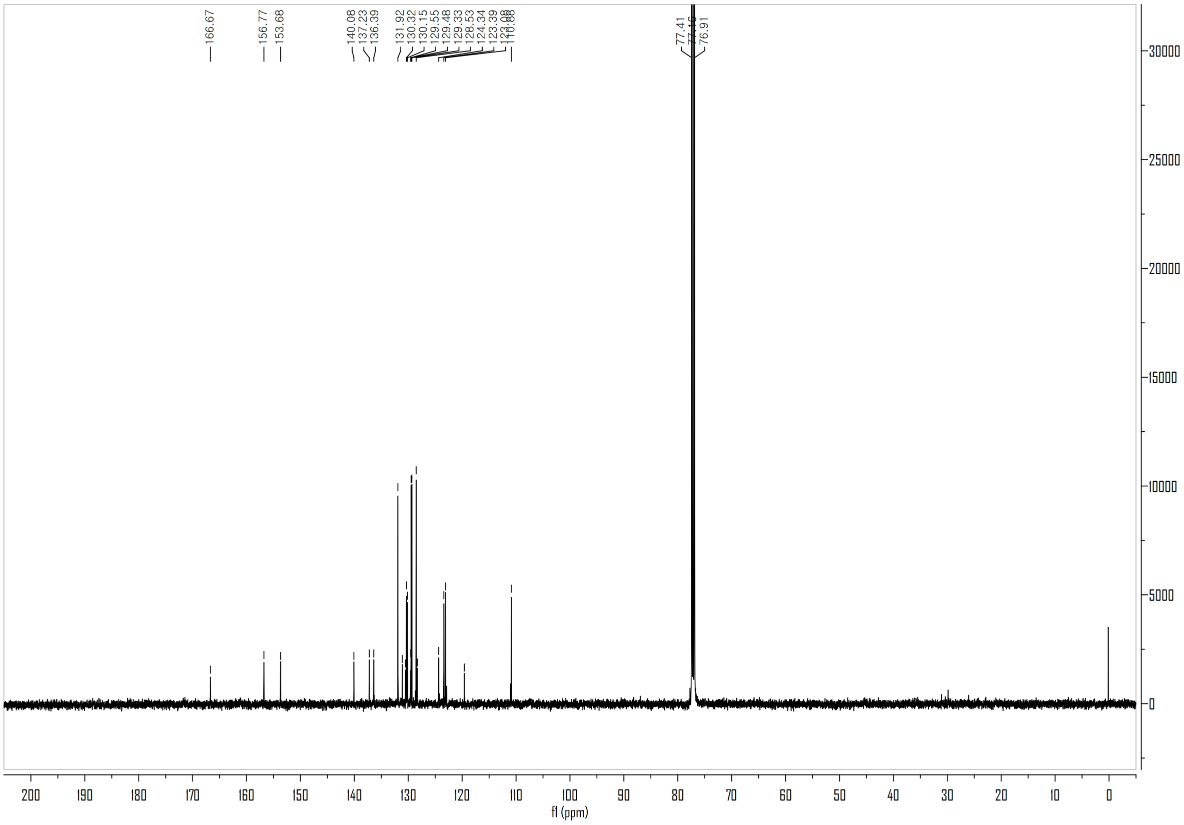

HSQC spectrum of (*Z*)-7d

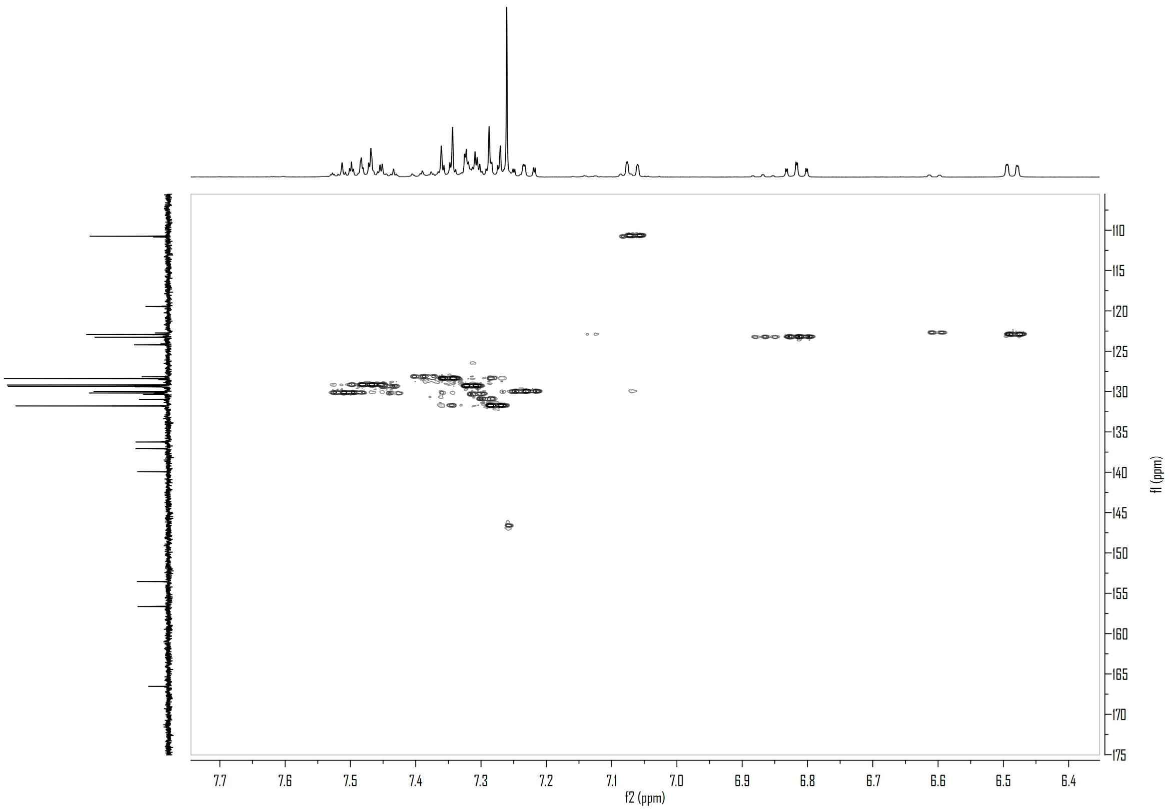

HMBC spectrum of (*Z*)-7d

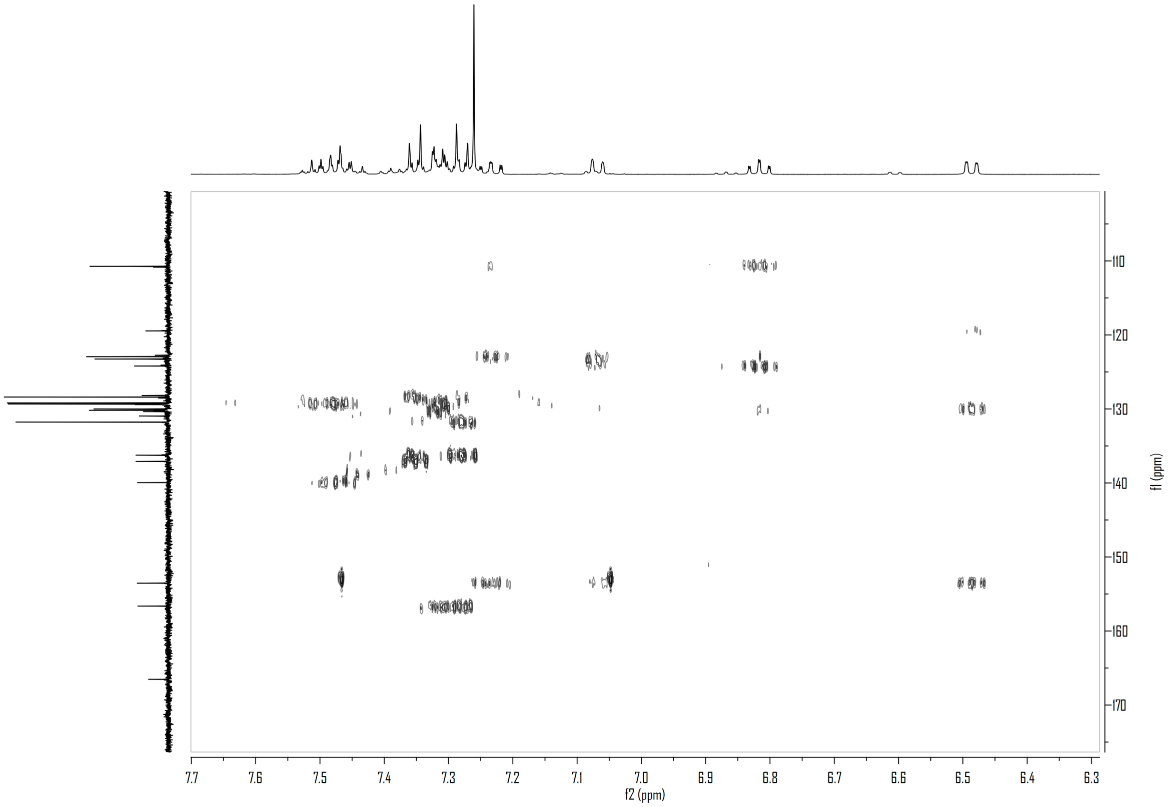

COSY spectrum of (*Z*)-7d

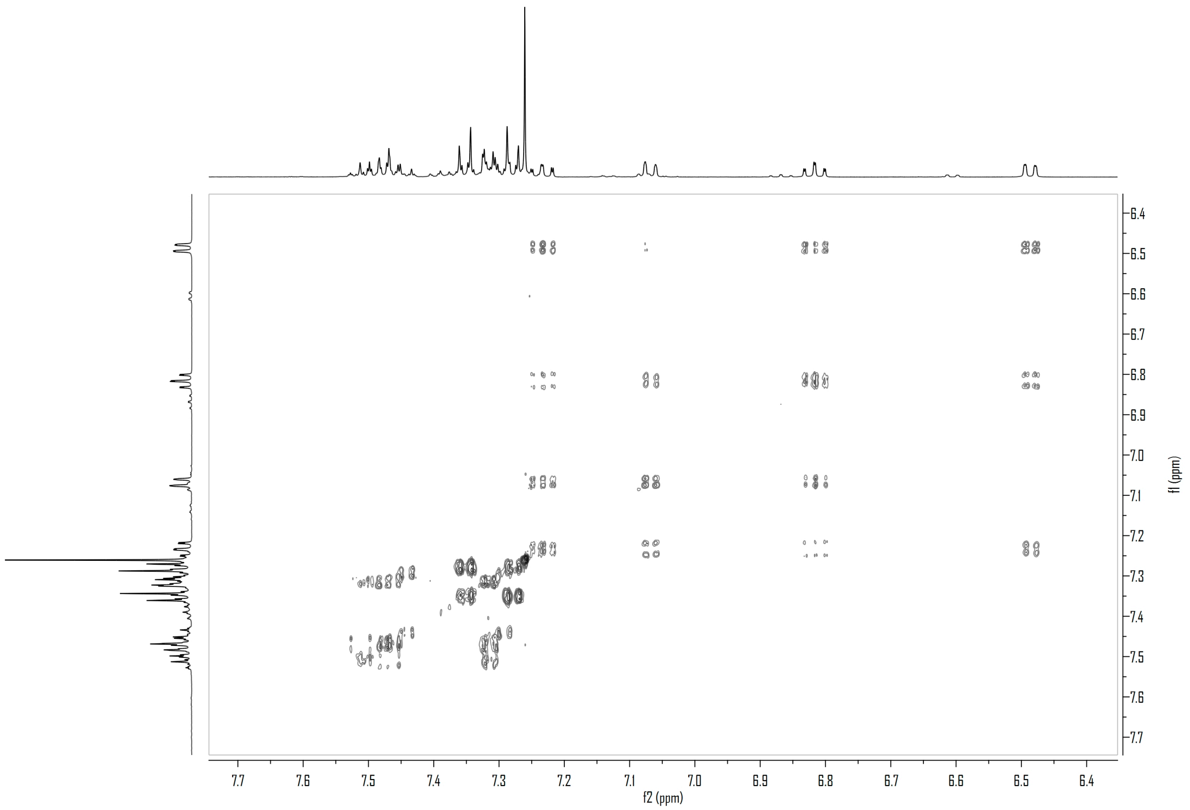

ROESY spectrum of (*Z*)-7d

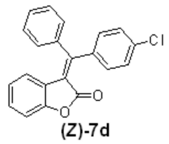

(Z)-7d

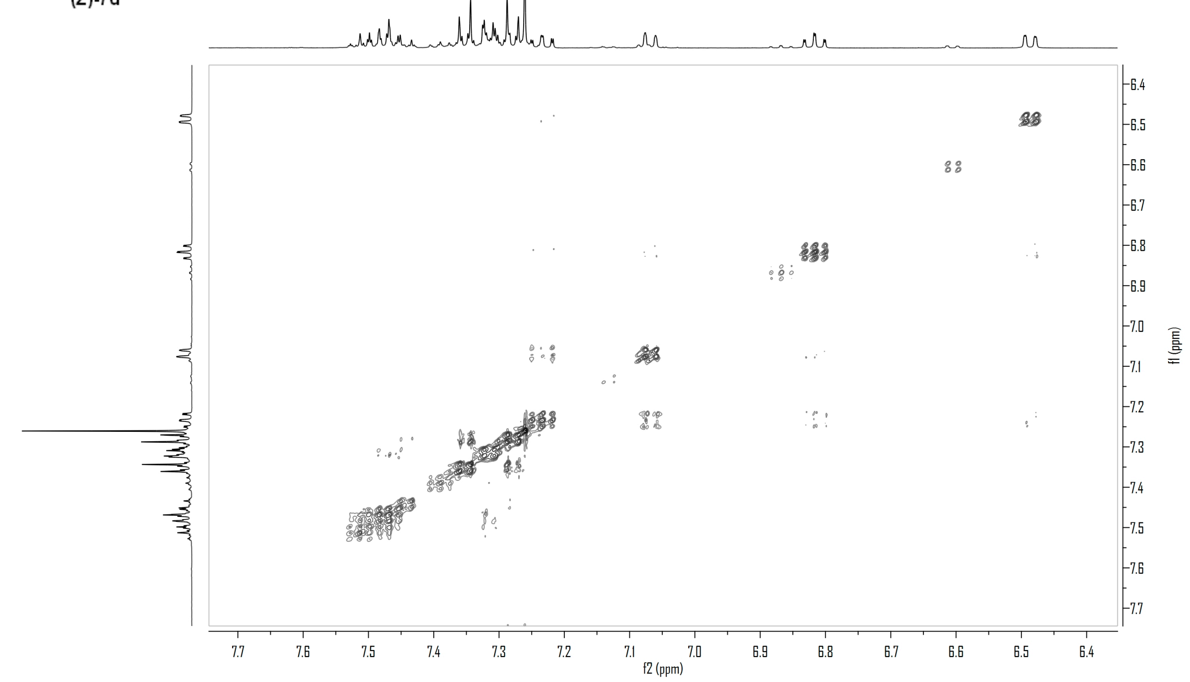

(*E*)-3-((4-chlorophenyl)(phenyl)methylene)benzofuran-2(3H)-one ((*E*)-7d)

^{1}H NMR spectrum of (*E*)-7d

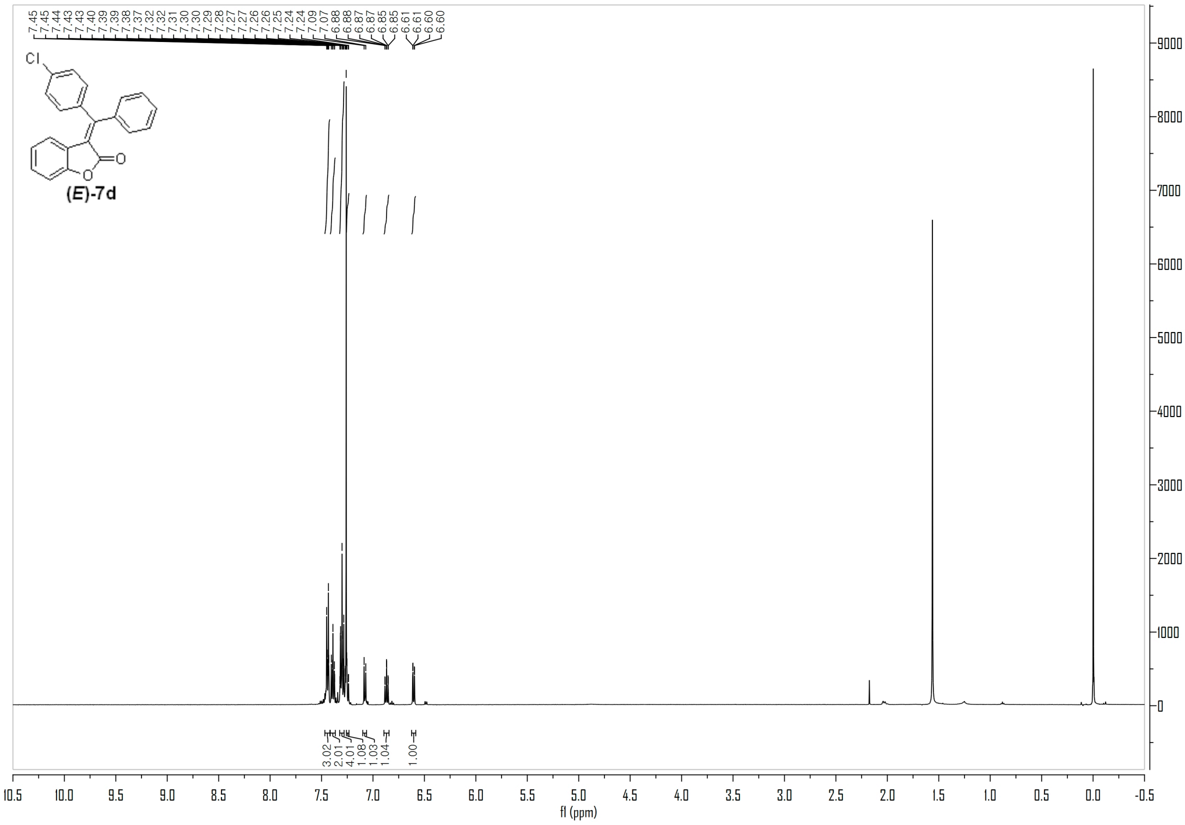

^{13}C NMR spectrum of (*E*)-7d

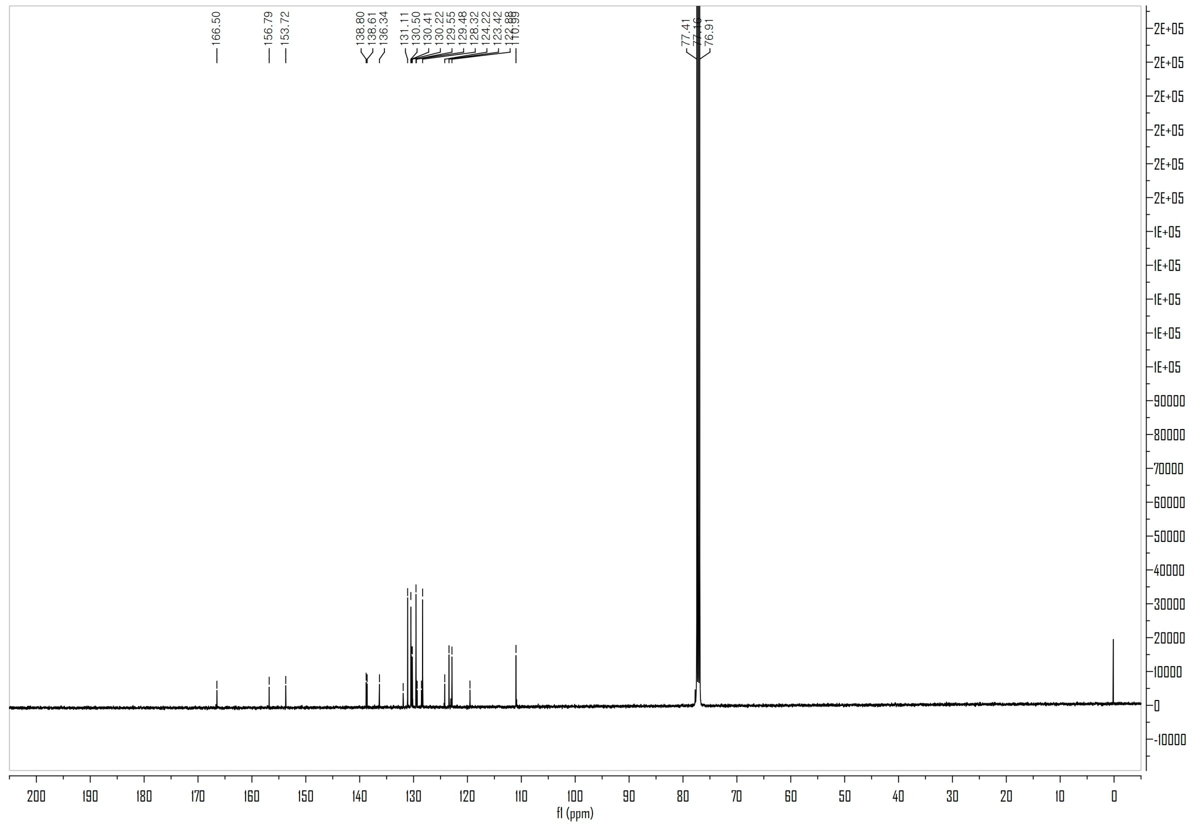

HSQC spectrum of (*E*)-7d

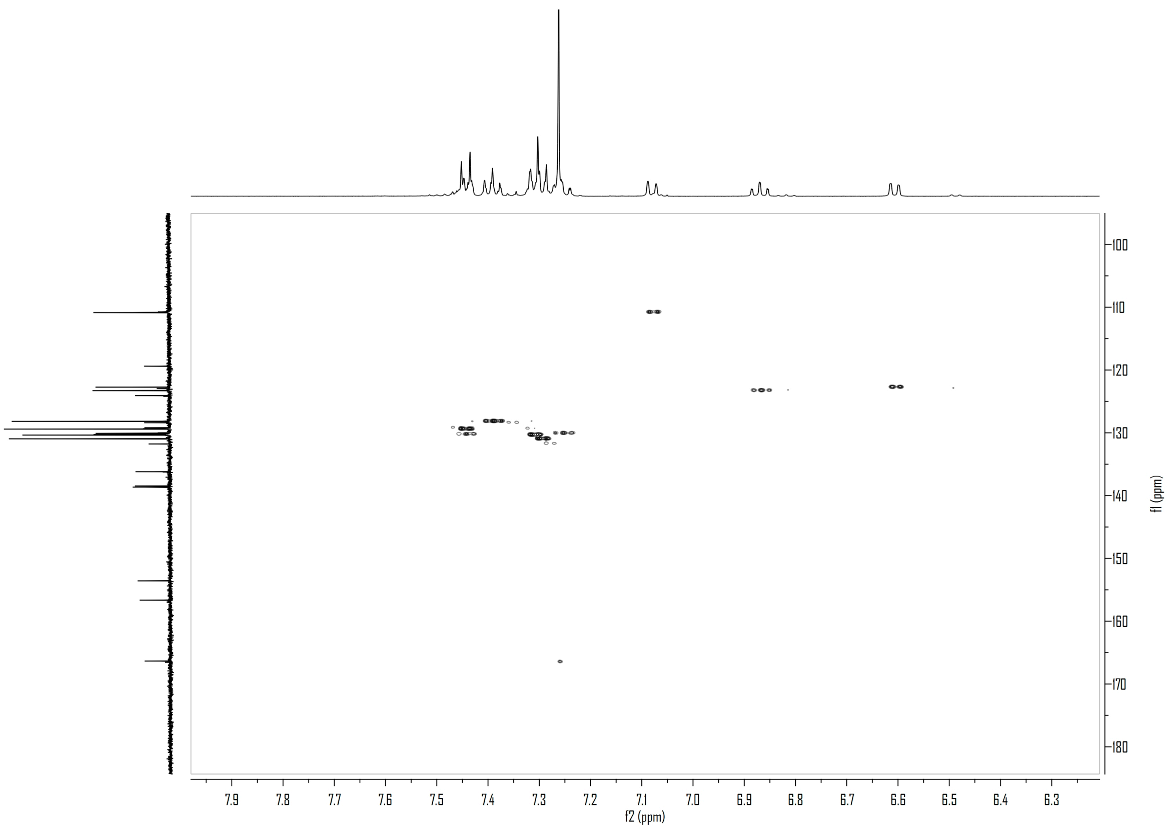

HMBC spectrum of (*E*)-7d

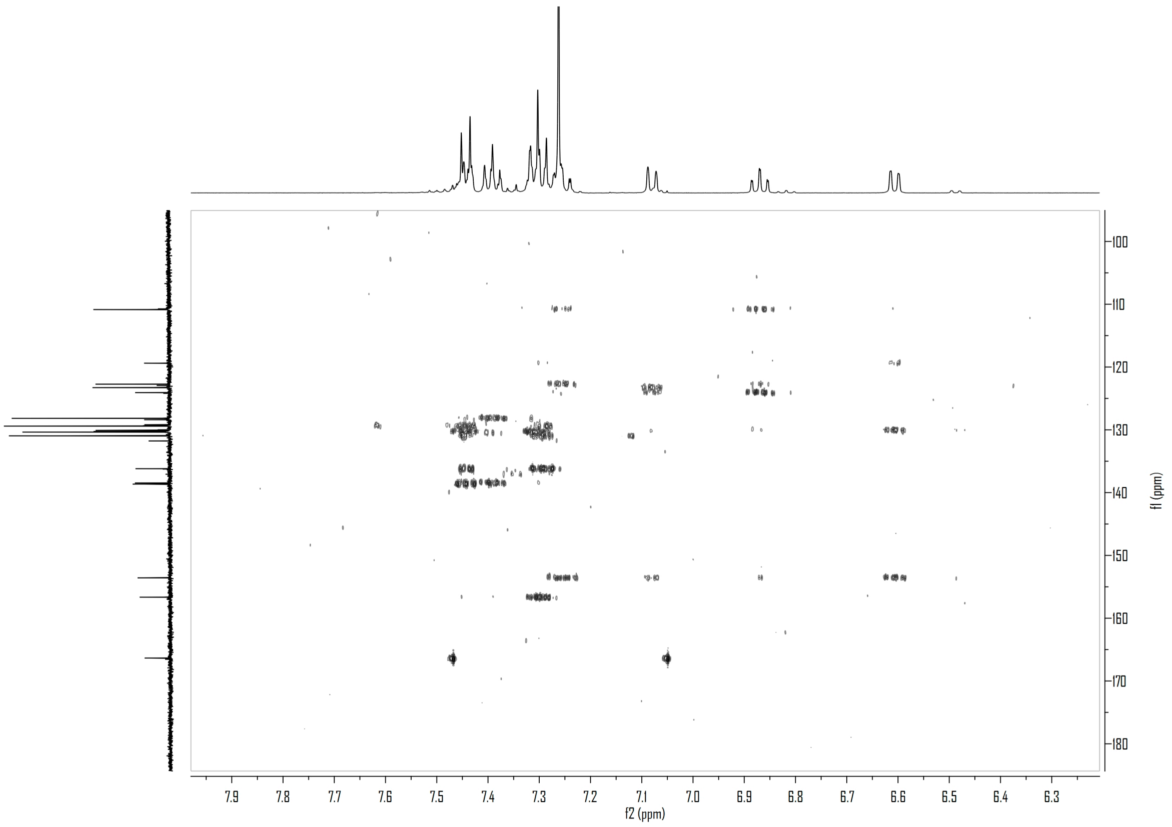

COSY spectrum of (*E*)-7d

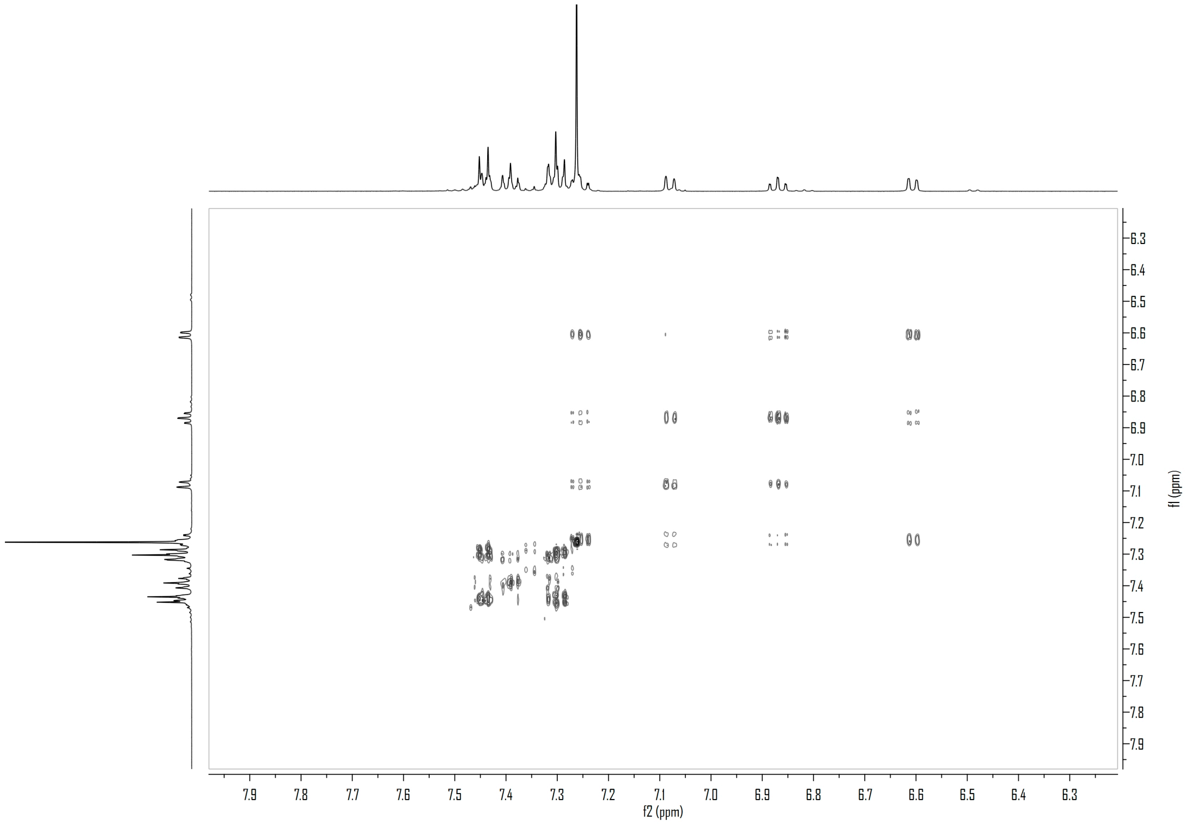

ROESY spectrum of (*E*)-7d

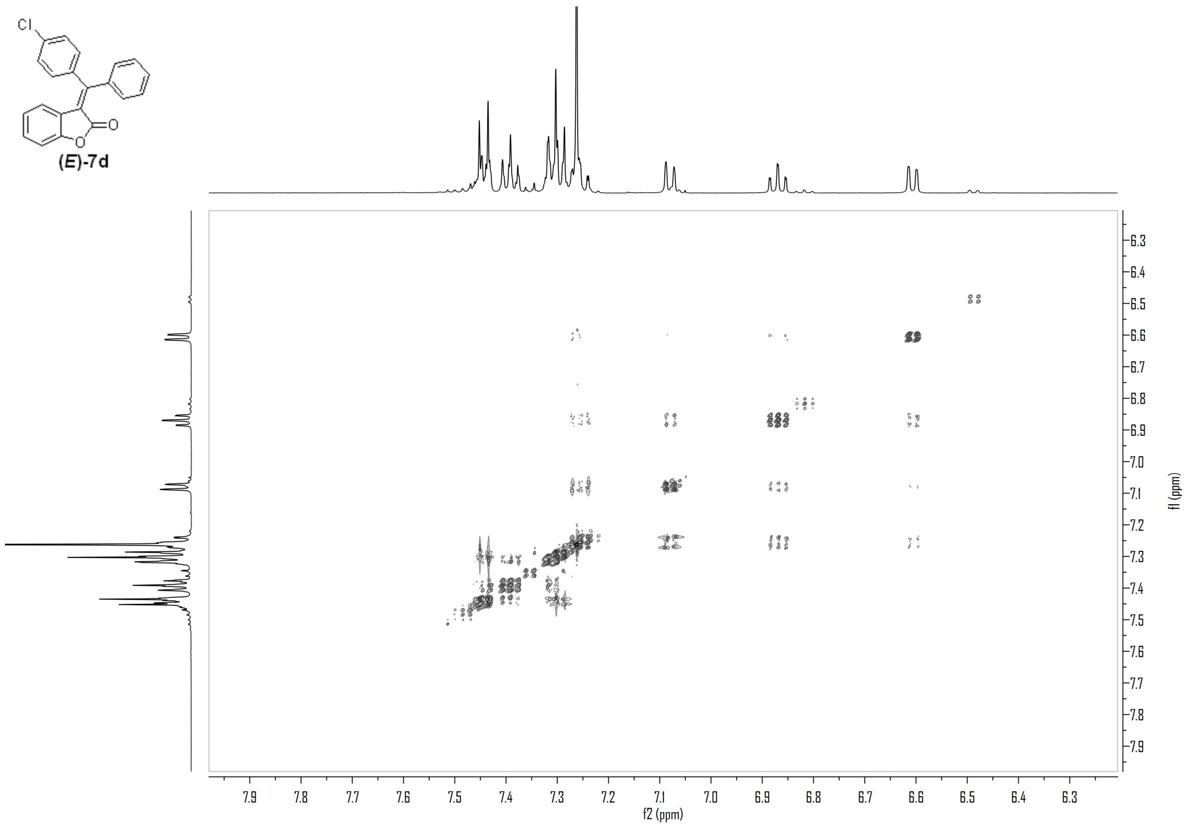

Supplement: Supplementary file 1 [file molecules-22-00503-s001.pdf]
